# Spatial confidence regions for combinations of excursion sets in image analysis

**DOI:** 10.1093/jrsssb/qkad104

**Published:** 2023-09-21

**Authors:** Thomas Maullin-Sapey, Armin Schwartzman, Thomas E Nichols

**Affiliations:** Nuffield Department of Population Health, Big Data Institute, Li Ka Shing Centre for Health Information and Discovery, University of Oxford, Oxford, UK; Division of Biostatistics, University of California, San Diego, CA, USA; Halicioğlu Data Science Institute, University of California, San Diego, CA, USA; Nuffield Department of Population Health, Big Data Institute, Li Ka Shing Centre for Health Information and Discovery, University of Oxford, Oxford, UK

**Keywords:** confidence regions, excursion sets, linear model, spatial statistics

## Abstract

The analysis of excursion sets in imaging data is essential to a wide range of scientific disciplines such as neuroimaging, climatology, and cosmology. Despite growing literature, there is little published concerning the comparison of processes that have been sampled across the same spatial region but which reflect different study conditions. Given a set of asymptotically Gaussian random fields, each corresponding to a sample acquired for a different study condition, this work aims to provide confidence statements about the intersection, or union, of the excursion sets across all fields. Such spatial regions are of natural interest as they directly correspond to the questions ‘Where do *all* random fields exceed a predetermined threshold?’, or ‘Where does *at least one* random field exceed a predetermined threshold?’. To assess the degree of spatial variability present, our method provides, with a desired confidence, subsets and supersets of spatial regions defined by logical conjunctions (i.e. set intersections) or disjunctions (i.e. set unions), without any assumption on the dependence between the different fields. The method is verified by extensive simulations and demonstrated using task-fMRI data to identify brain regions with activation common to four variants of a working memory task.

## 1 Introduction

The collection and analysis of imaging data, modelled as a random field regularly sampled on a spatial domain, is central to a broad range of scientific disciplines such as neuroimaging, climatology, and cosmology. By combining spatial data from *n* observations of a random field, researchers can estimate a spatially varying target function μ:S→R, where the spatial region *S* is a closed subset of $R^{N}$. Examples of such outcomes include cosmic microwave background in astronomy, temperature data for climate, and changes in blood oxygenation in the brain. Empirical interest often lies in identifying locations where the target function exceeds a certain value, *c*; for example ‘Where has a significant change in temperature occurred?’, or ‘Which part of the brain demonstrated a strong response to stimuli?’. Such questions are addressed by the study of excursion sets, sets of the form {s∈S:μ(s)≥c}.

Previous literature has documented many geometric and topological properties of excursion sets of random functions (c.f. Adler, 1981; Adler & Taylor, 2009; Azas & Wschebor, 2009; Cao, 1999; Torres, 1994). These include the Euler characteristic (a measure of topological structure), the Hausdorff dimension (indicating how fractal the set may be), Lipschitz-Killing curvatures (describing high-dimensional volumes and areas), and Betti numbers (describing how many stationary points appear above the threshold, *c*) (Adler, 1977; Adler et al., 2017; Pranav et al., 2019; Worsley, 1996). However, much of this work is limited to homogeneous stationary processes, i.e. those with zero mean and a spatial covariance structure dependent upon only distance. Only the most recent literature, including the present paper, concerns processes with a non-zero mean function and a potentially heterogeneous covariance function.

At present, little is published concerning how excursion sets may be compared to one another. Often researchers collect data from two or more study conditions and contrast the results to draw some meaningful inference. For example, comparing temperature changes in winter vs. summer, heat measurements gathered using different imaging modalities, or brain activity in healthy subjects across a range of tasks. Such settings are modelled as *M* spatially aligned, potentially correlated, estimates, ${\hat{\mu }}_{n}^{1},{\hat{\mu }}_{n}^{2},\ldots {\hat{\mu }}_{n}^{M}\;:\;S\to R$, of *M* target functions, $\mu^{1},\mu^{2},\ldots \mu^{M}$.

In such settings, research questions are often expressed as logical statements involving logical conjunctions, negations, and disjunctions. For example, a climatologist may ask where significant changes in temperature were observed during either winter *or* summer. Alternatively, a neuroscientist may ask where was cognitive behaviour observed in the brain during one task *and not* another. Formally, these questions may be expressed as ‘Where does $\mu^{1}$*or*$\mu^{2}$ exceed *c*?’ and ‘Where does $\mu^{1}$, *and not*$\mu^{2}$, exceed *c*?’, respectively.

**Figure 1.**
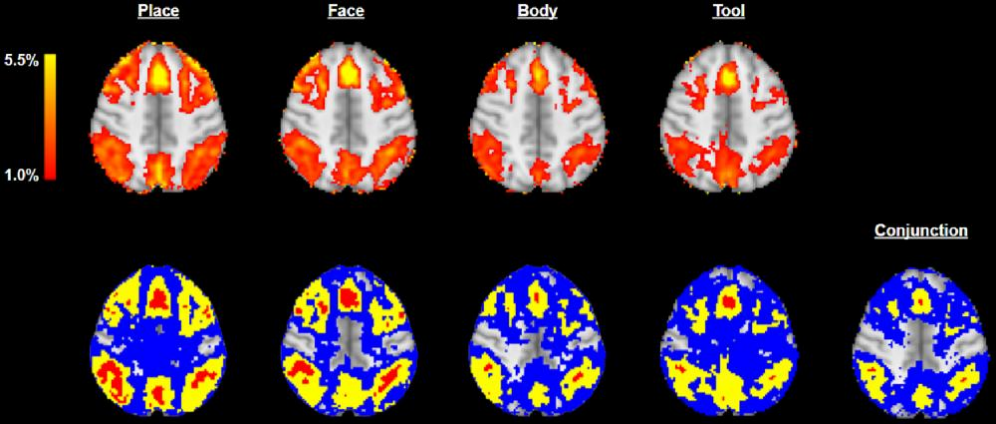
Functional magnetic resonance imaging (fMRI) % blood oxygenation level dependent (BOLD) images (top) and 95% confidence regions (CRs) (bottom left) for the Human Connectome Project working memory task for each of the four visual stimuli types, alongside conjunction CRs assessing the overlap of the four thresholded %BOLD images (bottom right). The thresholds employed were c=1% BOLD change, for all images, and α=0.05, for the CRs. Upper and lower CRs are displayed in red and blue, respectively. Across the bottom row, the yellow sets are the point estimates ${\hat{A}}_{c}^{1},\ldots {\hat{A}}_{c}^{4}$ and ${\hat{F}}_{c}$ respectively. The red conjunction set has localised regions in the superior frontal gyrus (top) and the angular gyri (left/right), for which we can assert with 95% confidence that, for all four study conditions, there was (at least) a 1% change in BOLD response. Displayed is axial slice z=46 mm.

Images corresponding to different study conditions are often compared via rudimentary visual inspection (see Dijkstra et al., 2021; Ferreira et al., 2021; Zhang et al., 2021 for recent examples of qualitative assessment of ‘overlap’ and ‘differences’ in neuroimaging). Such a subjective practice may lead to biased or misleading results. To formalise these inferences, recent work has proposed confidence regions (CRs), subsets, and supersets that bound the excursion set of a single target function with a given confidence (Bowring et al., 2019, 2021; Cuevas et al., 2006; French & Sain, 2013; Ren et al., 2022; Sommerfeld et al., 2018). However, current CR theory does not offer a means for investigating logical conjunctions and disjunctions of exceedance statements, i.e. intersections and unions of excursion sets. In this work, we provide a new method for generating CRs for ‘conjunction inference’ in image analysis, and then discuss how the proposed method may be adapted to allow the investigation of statements containing disjunctions and negations. An example application is provided in Figure 1, which shows CRs generated for the region of the brain that was active in all four variants of a working memory task (see Section 5 for further detail).

**Figure 2.**
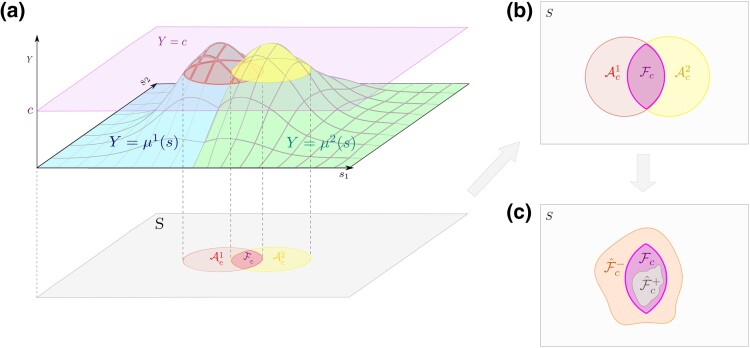
Illustration of the set $F_{c}$ for a setting in which M=2. Shown in (a) are two spatially varying target functions, $\mu^{1}(s)$ (blue) and $\mu^{2}(s)$ (green), overlaid and thresholded at the level *c*. In (b), the regions at which $\mu^{1}(s)\geq c$ and $\mu^{2}(s)\geq c$ are displayed as red and yellow circles, respectively, with the intersection set, $F_{c}$, illustrated in purple. In (c), potential CRs, ${\hat{F}}_{c}^{+}$ (grey) and ${\hat{F}}_{c}^{-}$ (orange), for $F_{c}$ are illustrated.

Formally, we primarily focus on the intersection of *M* excursion sets, over which the conjunction statement ‘$\mu^{1}(s)\geq c$*and*$\mu^{2}(s)\geq c$*and*⋯*and*$\mu^{M}(s)\geq c$’ holds. We denote M={1,…,M}, and for each i∈M define the *i*^*th*^ true and estimated excursion sets as


$$A_{c}^{i}=\{s\in S\;:\;\mu^{i}(s)\geq c\}\;\text{and}\;{\hat{A}}_{c}^{i}=\{s\in S\;:\;{\hat{\mu }}_{n}^{i}(s)\geq c\},$$

respectively. Next, we define $F_{c}\;:=\cap_{i}\{A_{c}^{i}{\}}_{i\in M}$ and ${\hat{F}}_{c}\;:=\cap_{i}\{{\hat{A}}_{c}^{i}{\}}_{i\in M}$. The spatial region we are interested in, and our estimate of that region, are now given by


$$F_{c}=\{s\in S\;:\;\min_{i\in M}\mu^{i}(s)\geq c\}\;\text{and}\;{\hat{F}}_{c}=\{s\in S\;:\;\min_{i\in M}{\hat{\mu }}_{n}^{i}(s)\geq c\},$$

respectively. An illustration of $F_{c}$ in a setting in which M=2 is provided in Figure 2. We note that the above construction can be adjusted to allow *c* to vary across study conditions (i.e. ‘$\mu^{1}(s)\geq c_{1}$*and*⋯*and*$\mu^{M}(s)\geq c_{M}$’ for $c_{1},\ldots ,c_{M}\in R$) by substituting each $\{\mu^{i}{\}}_{i\in M}$ for $\{\mu^{i}-c+c_{i}{\}}_{i\in M}$). For ease, we assume *c* is equal for all study conditions. In addition, although the results we shall present theoretically hold in arbitrary dimensions, we shall focus primarily on two-dimensional simulations and applications to investigate the method’s performance.

Our goal is to obtain CRs ${\hat{F}}_{c}^{+}$ and ${\hat{F}}_{c}^{-}$ such that the below holds as *n* tends to ∞:


(1) $$P[{\hat{F}}_{c}^{+}\subseteq F_{c}\subseteq {\hat{F}}_{c}^{-}]=1-\alpha$$

for a predefined tolerance level *α* (say, α=0.05). Pictorially, the above states that the sets ${\hat{F}}_{c}^{-}$, $F_{c}$, and ${\hat{F}}_{c}^{+}$ are nested within one another as shown in Figure 2c with probability (1−α). To generate such regions, we build on the theory of Sommerfeld et al. (2018) to show that if ${\hat{F}}_{c}^{+}$ and ${\hat{F}}_{c}^{-}$ are defined as appropriately chosen level sets of a standardised minimum process, the above probability may be approximated using quantiles of a well-defined random variable. Using a wild *t*-bootstrapping procedure, under the assumption of a Gaussian central limit theorem, we will then demonstrate that the relationship between this random variable and the above probability can be used to generate ${\hat{F}}_{c}^{+}$ and ${\hat{F}}_{c}^{-}$ for any desired value of *α*. Unlike previous literature on random excursion sets, in this work no model is assumed for the processes’ means or spatial covariance structure, and we do not make any assumption of between-‘study condition’ independence (i.e. $\{{\hat{\mu }}_{n}^{i}{\}}_{i\in M}$ may be correlated with one another).

If the CRs ${\hat{F}}_{c}^{+}$ and ${\hat{F}}_{c}^{-}$ closely resemble one another, this may be interpreted as signifying that ${\hat{F}}_{c}$ is a reliable estimate of $F_{c}$. Alternatively, little resemblance may suggest that there is a high degree of spatial variability present in the data and that the estimated shape, size, and locale of ${\hat{F}}_{c}$ are not particularly reliable. Such statements are of particular value in imaging applications where the aim of a statistical analysis is typically to assess how reliably some form of activity may be localised to a particular spatial region. For instance, in the fMRI example of Figure 1, ${\hat{F}}_{c}$ is the yellow conjunction region displayed in the bottom right panel, which represents an estimate of the brain regions which were active in response to *all four* variants of a working memory task. Here, the poor resemblance between the blue and red CRs may be interpreted as suggesting that more data is required for ${\hat{F}}_{c}$ to be reliable as an estimate of $F_{c}$.

Conjunction and disjunction inference is an active area of research in the broader statistical literature. A typical approach for such conjunction or disjunction inference is to employ null-hypothesis testing. A hypothesis test of a disjunction of nulls to assess evidence for a conjunction of alternative hypotheses can be conducted with an ‘intersection-union’ test. An intersection-union test rejects at level *α* when all of the individual nulls are rejected at level *α*. This approach has proven useful for tests of bioequivalence (Berger, 1997; Berger & Hsu, 1996), but does not consider the spatial aspect that is our focus here.

This work focuses on the quantification of *spatial uncertainty* present in the locale of intersections and unions of random excursion sets, which can be visualised using CRs. CR methodology has previously been developed for individual excursion sets in a wide range of contexts. For instance, Davenport et al. (2022) derive CRs for local extrema in Gaussian fields, whilst Craigmile et al. (2005) quantify uncertainty in extrema estimates within a Bayesian framework. Another active area of research concerns kernel density estimation, in which many authors have proposed CRs for excursion sets of kernel estimates (Chen et al., 2017; Mammen & Polonik, 2013; Qiao & Polonik, 2019). Further, in the setting of hotspot identification for geospatial data, CRs have been proposed to bound a target functions derived from Bayesian spatial mixed effects models (French & Hoeting, 2015; Hazra & Huser, 2021), as well as more general latent Gaussian models (Bolin & Lindgren, 2015; Mejia et al., 2020). Despite this, to our knowledge, no prior work exists that explicitly considers the generation of CRs for conjunction or disjunction inference. Moreover, such inference concerns spatial regions with piece-wise continuous boundaries, which are not typically considered in the CRs literature. The combinatoric nature of the boundary decompositions considered in this work adds an additional layer of complexity to the results and proofs provided, and has not previously been considered in the CRs literature.

In the following sections, we first describe the notation and assumptions upon which our theory relies. Following this, we provide the central theoretical result of this work, which relates equation (1) to an exceedance statement for a well-defined random variable. Next, via the use of the wild *t*-bootstrap, we detail how this result may be employed to obtain CRs for conjunction inference in the setting of linear regression modelling under the assumption of a Gaussian CLT. Finally, we validate our results with simulations and a real data example based on fMRI data taken from the Human Connectome Project (HCP) (Van Essen et al., 2013). Proofs of the theory presented in this work, alongside further illustration and extensive simulation results, are provided as online supplementary material.

## 2 Confidence regions for excursion set combinations

### 2.1 Notation

In the following sections, we shall need to denote the numerous possible low-dimensional sub-manifolds which can arise from intersecting the boundaries of *M* excursion sets. To do so, we first denote the power set of a finite set, *A*, as P(A), and define $P^{+}(A)$ as $P^{+}(A)=P(A)\setminus \{\emptyset \}$. For each i∈M, we define $\partial A_{c}^{i}$ as the level set $\left\{s\in S\;:\;\mu^{i}(s)=c\right\}$. Similarly, we define $\partial F_{c}$ as the level set $\left\{s\in S\;:\;\min_{i\in M}\mu^{i}(s)=c\right\}$. For a spatial set, B⊆S, its complement in *S* is denoted as $B^{c}=S\setminus B$, its topological closure is denoted as $\bar{B}$, its interior is denoted as $B^{\circ }$, and its boundary is denoted as ∂B. We note here that the notation $\partial A_{c}^{i}$ and ∂B may conflict with one another. This conflict is resolved, however, by the assumptions of Section 2.2 which ensure that the boundaries of $\{A_{c}^{i}{\}}_{i\in M}$ and $F_{c}$ are equal to the level sets $\{\partial A_{c}^{i}{\}}_{i\in M}$ and $\partial F_{c}$ (at least locally, in the vicinity of $\partial F_{c}$, which is sufficient for our purposes).

We can now partition the level set $\partial F_{c}$ into sub-manifolds using the set of possible intersections of the *M* excursion set boundaries. For all $\phi \in P^{+}(M)$, we define the boundary segment $\partial^{\phi }F_{c}$ as follows:


$$\partial^{\phi }F_{c}=\partial F_{c}\cap \left(\underset{i\in \phi }{⋂}\partial A_{c}^{i}\right)\cap \left(\underset{\;j\in M\setminus \phi }{⋂}(\partial A_{c}^{j}{)}^{c}\right).$$

**Figure 3.**
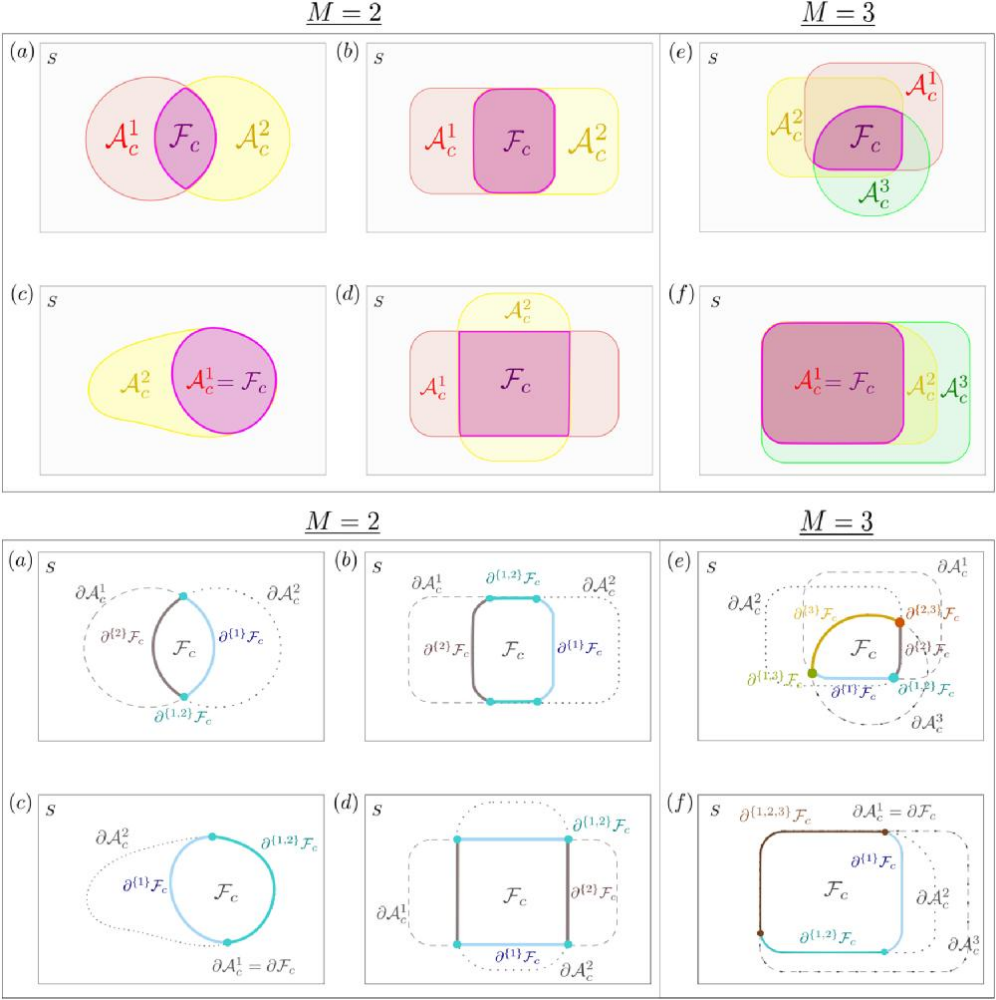
Annotated images of ‘overlapping’ excursion sets (top) with corresponding illustrations of boundary partitions (bottom) for settings in which the number of study conditions, *M*, is equal to 2 (left) or 3 (right). Shown in Panels (a)-(d) are two overlapping excursion sets $A_{c}^{1}$ and $A_{c}^{2}$. In Panels (e) and (f), a third set has also been included. Panels (b) and (f) illustrate settings in which the excursion sets share boundaries, Panels (a), (d) and (e) exemplify instances where $F_{c}$ has sharp corners, and Panels (c) and (f) show that the excursion sets may be nested.

We note it is possible that, for some $\phi \in P^{+}(M)$, the boundary segment $\partial^{\phi }F_{c}$ is empty (in fact, this is often the case in practice). This notation is crucial to the statement of Theorem 2.1 and is illustrated in Figure 3.

In the fMRI example presented in Section 1, M={1,2,3,4} is a set of indexes representing the four working memory conditions and $F_{c}$ represents the brain regions that are active in response to all four conditions. In this setting, an example of the notation $\partial^{\phi }F_{c}$ is given by the boundary segment $\partial^{\left\{1\right\}}F_{c}$, which represents the edges of $F_{c}$ which arose solely due to the first working memory condition.

### 2.2 Assumptions

In this section, we outline and discuss the assumptions upon which our theory relies. Further detailed discussion of Assumption 2.2.3, alongside illustration, is given in online Supplementary Theory Section S2.

Assumption 2.2.1 For each i∈M, there exists a bounded function $\sigma^{i}\;:\;S\to R^{+}$ and positive sequence $\tau_{n}\to 0$, such that the below joint central limit theorem (CLT) holds:

{μ^ni(s)−μi(s)τnσi(s)}s∈S,i∈Md→{Gi(s)}s∈S,i∈M,

where $\{G^{i}(s){\}}_{s\in S,i\in M}$ is a well-defined multivariate random field with continuous sample paths in *S* and d→ represents convergence in distribution.

Remark This assumption introduces the notation $\sigma^{i}(s)$ and $\tau_{n}$. In typical applications, $\sigma^{i}(s)$ represents standard deviation across observations (for study condition *i*, at spatial location *s*) and $\tau_{n}$ is a decreasing function of sample size. If ${\hat{\mu }}_{n}^{i}$ were derived as the mean of *n* i.i.d. observed Gaussian random fields $\left\{X_{1}^{i},\ldots ,X_{n}^{i}\right\}$, then Assumption 2.2.1 would reduce to a standard Gaussian functional CLT with $\mu^{i}=E[X_{1}^{i}],\sigma^{i}=\sqrt{\text{Var}(X_{1}^{i})}$, $\tau_{n}=n^{-\frac{1}{2}}$ and $G^{i}$ being a Gaussian random field with unit variance.

For conciseness in the following text, we now define $\{g^{i}{\}}_{i\in M}$ and $\{{\hat{g}}_{n}^{i}{\}}_{i\in M}$ as

$$g^{i}(s)=\frac{\mu^{i}(s)-c}{\sigma^{i}(s)}\;\text{and}\;{\hat{g}}_{n}^{i}(s)=\frac{{\hat{\mu }}_{n}^{i}(s)-c}{\sigma^{i}(s)}.$$

Pictorially, each $\left\{g^{i}\right\}$ may be visualised as a transformation of the surfaces depicted in Figure 2. Such a transformation would first vertically translate the entire image so that the purple plane, {Y=c}, coincides with the plane formed by the $s_{1}$ and $s_{2}$ axis. It would then stretch, or shrink, the resulting function away from, or towards, this plane. From this, it can be seen that the excursion sets of $\left\{\mu^{i}\right\}$ and $\left\{{\hat{\mu }}^{i}\right\}$ at the level *c* are equal to those of $\left\{g^{i}\right\}$ and $\left\{{\hat{g}}_{n}^{i}\right\}$ at the level 0 (i.e. $A_{c}^{i}=\{s\in S\;:\;g^{i}(s)\geq 0\}$ and ${\hat{A}}_{c}^{i}=\{s\in S\;:\;{\hat{g}}_{n}^{i}(s)\geq 0\}$).

Assumption 2.2.2 We assume that:

- The functions $\{g^{i}{\}}_{i\in M}$ are continuous on *S*.
- For *n* large enough, the functions $\{{\hat{g}}_{n}^{i}{\}}_{i\in M}$ are continuous on *S*.

Remark For the practical settings we consider, such as fMRI neuroimaging, astronomy and climate, the above assumptions of continuity are ubiquitous in the literature. Common reasons for this include: (1) the underlying estimator is assumed to be drawn from a smooth process and (2) spatial smoothing is often applied to the data during preprocessing to improve the signal-to-noise ratio (SNR) (see Adler & Taylor, 2009; Brett et al., 2004; Poldrack et al., 2011).

Assumption 2.2.2 ensures that all limits taken across space in the proofs in online Supplementary Theory Section S4 are well defined. However, it does not guarantee that the sets $\{A_{c}^{i}{\}}_{i\in M}$ will resemble the well-defined ‘blobs’ which may be expected. Without further assumptions, it is still possible for $\{A_{c}^{i}{\}}_{i\in M}$ to appear extremely erratic, possessing jagged edges and displaying fractal-like behaviour. A well-known example of a random field that satisfies Assumption 2.2.2 but which is not well-behaved in the sense we desire is the highly fractal Wiener process (also known as Brownian motion, c.f. Adler, 1981). To restrict our attention to situations encountered in imaging studies, the below assumption is required.

Assumption 2.2.3 We assume that:

- Every open ball around a point on the boundary $\partial F_{c}$ has a non-empty intersection with $(F_{c}{)}^{\circ }$. In addition, for all $\phi \in P^{+}(M)$ every open ball around a point in $\partial^{\phi }F_{c}$ has a non-empty intersection with the set:
  $$J_{c}^{\phi }\;:\;=\left(\underset{i\in \phi }{⋂}(A_{c}^{i}{)}^{c}\right)\cap \left(\underset{\;j\in M\setminus \phi }{⋂}(A_{c}^{\;j}{)}^{\circ }\right),$$
  where, if M∖ϕ is empty, the last term is defined to be equal to *S*.
- There is an open neighbourhood of $\partial F_{c}$ over which the functions $\{g^{i}{\}}_{i\in M}$ and, for *n* large enough, $\{{\hat{g}}_{n}^{i}{\}}_{i\in M}$ are $C^{1}$ with finite, non-zero, gradients. The same condition holds for the restrictions of each $\{g^{i}{\}}_{i\in M}$ and $\{{\hat{g}}_{n}^{i}{\}}_{i\in M}$ to ∂S.
- For every point $s\in \partial F_{c}$ and $\phi \in P^{+}(M)$, if $s\in \partial (\underset{i\in \phi }{⋂}A_{c}^{i})$ then the set $\partial (\underset{i\in \phi }{⋂}A_{c}^{i})$ partitions every sufficiently small open ball around *s* into exactly two components, each of which is path-connected.

Remark The above statements ensure that $F_{c}$ is non-empty and has a well-defined boundary which is equal to the level set $\partial F_{c}=\{s\in S\;:\;\min_{i\in M}g^{i}(s)=0\}$. All three statements ensure that no changes in topology occur at the level *c* by requiring that $\partial F_{c}$ does not contain plateaus, local minima or maxima. In addition, each statement handles pathological cases that the others do not. Statement (a) handles cases in which $\{\partial A_{c}^{i}{\}}_{i\in M}$ coincide exactly with ∂S, Statement (b) ensures that the notion of the gradient is well defined in a neighbourhood of $\partial F_{c}$, and Statement (c) handles unusual, potentially unintuitive, fractal-like behaviour which is not ruled out by the first two statements. Further nuanced discussion and illustrations of the degenerate fractal-like cases that are ruled out by Statement (c) are provided in online Supplementary Theory Section S2.

Each of the examples presented in Figure 3 satisfies Assumptions 2.2.2 and 2.2.3 and highlight several features worthy of note. Firstly, as shown in examples (d) and (e), we do not assume $\partial F_{c}$ is a $C^{1}$ curve, but rather piece-wise $C^{1}$. Secondly, the assumptions allow for the possibility that the excursion sets could be nested within one another (c.f. examples (c) and (f)). Thirdly, the assumptions permit the excursion sets to share common boundaries (see examples (b), (c), and (f)). The last two observations are of practical relevance as, in many applications, it may be expected that the target functions for different study conditions exhibit a strong similarity. This relevance is exemplified by the four bottom-left panels of Figure 1 in Section 1, in which the regions of the brain that displayed activation in response to four study conditions are displayed in yellow. It can be seen that these regions are notably similar in terms of anatomy. Further, we note that, throughout this work, there is no requirement that $F_{c}$ be (globally) path-connected. For instance, in the fMRI example shown in Figure 1, the estimated yellow set ${\hat{F}}_{c}$ in the bottom-right panel has many disconnected components.

### 2.3 Theory

We now present the central result of this work, Theorem 2.1. To do so, we now define the nested sets ${\hat{F}}_{c}^{-}$ and ${\hat{F}}_{c}^{+}$ by thresholding the statistic $\tau_{n}^{-1}\min_{i\in M}{\hat{g}}_{n}^{i}(s)$:


$${\hat{F}}_{c}^{\pm }\;:\;={\hat{F}}_{c}^{\pm }(a)=\{s\in S\;:\;\tau_{n}^{-1}\min_{i\in M}{\hat{g}}_{n}^{i}(s)\geq \pm a\},$$

using some constant $a\in R^{+}$. To find an appropriate value of *a*, such that equation (1) holds for a desired tolerance level (e.g. α=0.05), Theorem 2.1 is required.

Theorem 2.1 Under the assumptions of Section 2.2, the below holds:

(2) $$\lim_{n\to \infty }P[{\hat{F}}_{c}^{+}\subseteq F_{c}\subseteq {\hat{F}}_{c}^{-}]=P[H\leq a],$$

where the variable *H* is defined as follows:

$$H=\max_{\phi \in P^{+}(M)}(\underset{s\in \partial^{\phi }F_{c}}{\sup}|\min_{i\in \phi }(G^{i}(s))|).$$

The above theorem shows that violations of the inclusion statement, ${\hat{F}}_{c}^{+}\subseteq F_{c}\subseteq {\hat{F}}_{c}^{-}$, are dependent on the extreme values of limiting processes $\left\{G^{i}\right\}$ along the boundary $\partial F_{c}$. In the context of the fMRI example of Section 1, this means that the probability that the red and blue CRs in Figure 1 correctly bound the conjunction region for the four working memory conditions depends solely on the measurement noise distribution along the boundary of this region. The exact relationship between the measurement noise along a given boundary segment, and the probability of the inclusion failing in a vicinity of that segment, depends directly on which study conditions the segment was derived from. For instance, in the example of Figure 3c, it may be expected that the probability of observing a violation along the blue boundary segment, $\partial^{\left\{1\right\}}F_{c}$, will depend upon only the limiting noise distribution of the first study condition, $G^{1}$. However, for the green boundary segment, $\partial^{\left\{1,2\right\}}F_{c}$, violations of the subset condition may depend on the noise distributions of both study conditions.

From the above, it follows that if a∈R satisfies P[H≤a]=1−α then asymptotically, the inclusion probability, $P[{\hat{F}}_{c}^{+}\subseteq F_{c}\subseteq {\hat{F}}_{c}^{-}]$, will also equal 1−α. Thus, given the (1−α)th quantile of the distribution of *H*, CRs ${\hat{F}}_{c}^{-}$ and ${\hat{F}}_{c}^{+}$ may be constructed which satisfy equation (1). We shall take up the task of evaluating the quantiles of *H* in Section 3. Using De Morgan’s laws and direct algebraic substitution, Theorem 2.1 may also be employed to answer questions about logical disjunctions and negations. For brevity, the extraneous details are not given here but are instead discussed at length with worked examples in online Supplementary Theory Section S3.

## 3 Spatial conjunction inference for the linear model

In this section, we build upon the work of Sommerfeld et al. (2018) and Bowring et al. (2019), in which CRs were generated for a single target function, μ(s), derived from a linear regression model. In Section 3.1 we introduce the spatially varying linear model (LM) for *M* study conditions and, in Section 3.2, we apply the wild *t*-bootstrap to estimate quantiles of the variable *H*. Further implementation details, for when data are sampled from a discrete lattice rather than across a continuous space, alongside pseudocode, are provided in online Supplementary Results Section S2. This section focuses solely on conjunction inference. However, the methods described here may also be employed to perform inference on other logical statements via the corollaries of Section 2.3.

### 3.1 Model specification

For each study condition, study condition i∈M, we assume that the data follow a spatially varying LM defined at location s∈S as


(3) $$Y^{i}(s)=X^{i}\beta^{i}(s)+\epsilon^{i}(s),\;\epsilon^{i}(s)∼N(0,\Sigma^{i}(s)).$$

The known quantities in the model are: the (n×1) vector of responses, $Y^{i}(s)$, and the (n×p) design matrix, $X^{i}$. The unknown model parameters are: the (p×1) vector of regression coefficients, $\beta^{i}(s)$, and the (n×n) random error covariance matrix, $\Sigma^{i}(s)$. We emphasise that $\beta^{i}(s)$ and $\Sigma^{i}(s)$ model the signal observed at location s∈S, and do not incorporate any spatial information. In particular, $\Sigma^{i}(s)$ denotes the covariance between the *n* observed values at *s*, and not the spatial covariance of the field.

To illustrate the above notation in practice, we briefly detail its usage for the fMRI example of Section 1. Here, $Y^{i}$ represents the blood oxygenation level dependent (BOLD) signal, a proxy measure of brain function derived from the properties of blood oxygenation, for *n* subjects in response to study condition *i*. $X^{i}$ represents covariates of interest (e.g. subject age, gender, body mass index, etc.) which are assumed not to vary across the brain. $\beta^{i}$ models the relation between $X^{i}$ and $Y^{i}$, and $\epsilon^{i}$ represents random measurement noise.

For each spatial location, s∈S, the generalised least squares estimator of the parameter vector $\beta^{i}(s)$, ${\hat{\beta }}^{i}(s)$, is given by


$${\hat{\beta }}^{i}(s)=(X^{i^{\prime }}\Sigma^{i}{\left(s\right)}^{-1}X^{i}{)}^{-1}X^{i^{\prime }}\Sigma^{i}{\left(s\right)}^{-1}Y^{i}(s).$$

In this context, under the *i*^*th*^ study condition, at each spatial location *s*, our interest lies in assessing whether linear relationships hold between the elements of the parameter vector $\beta^{i}(s)$. Such relationships may be expressed using a contrast vector $L^{i}$ of dimension (p×1). In the notation of the previous sections, the target functions for the spatially varying LM (i.e. the surfaces depicted in Figure 2) are given as $\mu^{i}(s)=L^{i^{\prime }}\beta^{i}(s)$, with corresponding estimator functions ${\hat{\mu }}_{n}^{i}(s)=L^{i^{\prime }}{\hat{\beta }}^{i}(s)$. $\tau_{n}^{-1}\sigma^{i}(s)$ is the contrast standard error, given as


$$\tau_{n}^{-1}\sigma^{i}(s)=[L^{i^{\prime }}(X^{i^{\prime }}\Sigma^{i}{\left(s\right)}^{-1}X^{i}{)}^{-1}L^{i}{]}^{\frac{1}{2}}.$$

Although we have presented $\{{\hat{\beta }}^{i}(s){\}}_{i\in M}$ as being estimated separately using *M* different models, there is nothing in our theory that prevent the same model being used for all *M* study conditions. For example, it is possible for the model matrices, $\{Y^{i}(s){\}}_{i\in M}$ and $\{X^{i}{\}}_{i\in M}$, and parameter vector, $\{\beta^{i}(s){\}}_{i\in M}$, to be equal for all i∈M, with the only distinction between study conditions being represented by the contrast vector $L^{i}$. This observation is noteworthy as it allows researchers to compile all study conditions into a single LM to obtain a heightened statistical power.

In the settings we are motivated by, such as neuroscience and geospatial data, it is common to assume that $Y^{i}(s)$, $\Sigma^{i}(s)$ and $\beta^{i}(s)$ are smooth functions over space. Such assumptions are reasonable due to the point spread function of imaging devices or spatial smoothing applied during data preprocessing. As a result, we assume that derivative measures, such as contrast estimates and statistical values, are also spatially smooth. In the fMRI example of Section 1, the yellow excursion sets in the four bottom-left panels of Figure 1 were derived from a linear regression as excursion sets of the form ${\hat{A}}_{c}^{i}\;:\;=\{s\in S\;:\;L{\hat{\beta }}^{i}(s)\geq c\}$, and each represent the BOLD response to the *i*th working memory stimuli type, respectively.

To apply Theorem 2.1 to the above LM, we now assume that Assumptions 2.2.1–2.2.3 hold. Such assumptions are commonly satisfied by standard conditions placed on the continuity and boundedness of the increments and moments of $\{\beta^{i}(s){\}}_{s\in S}$ and $\{\epsilon^{i}(s){\}}_{s\in S}$. A sufficient list of such conditions is provided in online Supplementary Theory Section S5 and further detail may be found in Sommerfeld et al. (2018). We note here that the covariance matrix, $\Sigma^{i}(s)$, is usually unknown in practice, meaning the function $\tau_{n}^{-1}\sigma^{i}(s)$ cannot be calculated. As demonstrated in Sommerfeld et al. (2018), however, $\Sigma^{i}(s)$ can be replaced by any consistent estimator ${\hat{\Sigma }}^{i}(s)$ and the assumptions given in Section 2.2 are still satisfied. Such estimation is common in the statistics literature and is typically achieved by assuming that some fixed correlation structure applies to $\Sigma^{i}(s)$ (e.g. diagonal independence, auto-regressive, etc.) so that the number of independent variance parameters which must be estimated is small.

### 3.2 The wild *t*-bootstrap

In practice, the distribution of *H* is unknown and must be estimated. In this section, for the model specification described in Section 3.1, we employ a wild *t*-bootstrap resampling scheme to obtain the quantile, *a*, of *H* which satisfies P[H≤a]=1−α.

To do so, we first define the (n×1)-dimensional decorrelated residual vector, $R^{i}$, for the LM of the *i*^*th*^ study condition (i∈M) as


$$[R_{1}^{i}(s),\ldots ,R_{n}^{i}(s)]\prime =R^{i}(s)=[{\hat{\Sigma }}^{i}(s){]}^{-\frac{1}{2}}(Y^{i}(s)-X^{i}{\hat{\beta }}^{i}(s)).$$

The wild *t*-bootstrap proceeds by, in each bootstrap instance, generating *n* i.i.d. Rademacher random variables, $\left\{r_{1},\ldots ,r_{n}\right\}$ (random variables which take the values −1 and +1 with equal probability) independently of the data. For the *i*^*th*^ study condition, at spatial location *s*, a new bootstrap sample is then obtained by multiplying the elements of the decorrelated residual vector by the Rademacher variables (i.e. the new sample is given by $\left\{r_{1}R_{1}^{i}(s),\ldots ,r_{n}R_{n}^{i}(s)\right\}$). Once the boostrap sample has been generated, the standard deviation of the bootstrap sample, ${\hat{\sigma }}^{*,i}(s)$, is calculated. A bootstrap instance of the variable $G^{i}$, ${\tilde{G}}^{i}$, is now generated as follows:


$${\tilde{G}}^{i}(s)=n^{-\frac{1}{2}}\sum_{l=1}^{n}\frac{r_{l}R_{l}^{i}(s)}{{\hat{\sigma }}^{*,i}(s)}.$$

A bootstrap instance of the variable *H*, $\tilde{H}$, may then be computed using the bootstrap variables $\{{\tilde{G}}^{i}(s){\}}_{s\in S,i\in M}$ as follows:


(4) $$\tilde{H}=\max_{\phi \in P^{+}(M)}(\underset{s\in \partial^{\phi }{\hat{F}}_{c}}{\sup}|\min_{i\in \phi }({\tilde{G}}^{i}(s))|).$$

Quantiles of the distribution of *H* may now be estimated empirically from the observed sampling distribution of $\tilde{H}$. Discussion of how the above supremum, taken across continuous space, is evaluated in practice is deferred to online Supplementary Results Section S2.1. We note here that, as the locations of the boundary segments $\left\{\partial^{\phi }F_{c}\right\}$, depicted in Figure 3, are in practice unknown, the boundary segments $\left\{\partial^{\phi }F_{c}\right\}$ have been substituted for estimates $\left\{\partial^{\phi }{\hat{F}}_{c}\right\}$. The performance of substitutions of this form is discussed and verified extensively in the works of Sommerfeld et al. (2018), Bowring et al. (2019), and Bowring et al. (2021). In keeping with these publications, all simulations in this work have been replicated using both the true and estimated boundary segments, $\left\{\partial^{\phi }F_{c}\right\}$ and $\left\{\partial^{\phi }{\hat{F}}_{c}\right\}$, with a full comparison being provided in online Supplementary Results Sections S5 and S6. We note that, due to this substitution, our proposed method may not be applied when ${\hat{F}}_{c}$ is empty.

Telschow and Schwartzman (2021) show that the above procedure provides asymptotically consistent estimates of the distribution of *H* if each $\{G^{i}{\}}_{i\in M}$ satisfies the below.

Assumption 3.2.1 For each $\{G^{i}{\}}_{i\in M}$, we assume:

- $\{G^{i}{\}}_{i\in M}$ is $L^{2}$ Lipschitz with respect to the Euclidean distance metric. In other words, there exists a random variable *A* satisfying $E[|A{|}^{2}]<\infty$ such that $|G^{i}(s)-G^{i}(s^{\prime })|\leq A|s-s^{\prime }|\text{ for all }s,s^{\prime }\in S$.
- $\{G^{i}{\}}_{i\in M}$ has finite $l_{\infty }$ second moment. That is, $E[|G^{i}(s){|}_{\infty }^{2}]<\infty$.

The above procedure is reliant on the Gaussianity of each $G^{i}$. However, it has been suggested that this approach may also be suitable for estimating quantiles of maxima distributions for general symmetric $G^{i}$ (see Efron & Tibshirani, 1994; Hesterberg, 2015; Wu, 1986).

In order to utilise the results of Section 2 when the random variables $\{G^{i}{\}}_{i\in M}$ are not symmetric, the wild *t*-bootstrap must be adjusted to account for skewness. One approach commonly adopted for this purpose is to replace the Rademacher variables in the above with Mammen variables (variables which take values $-(\sqrt{5}-1)/2$ and $(\sqrt{5}+1)/2$ with probabilities $\left(\sqrt{5}+1)/(2\sqrt{5}\right)$ and $\left(\sqrt{5}-1)/(2\sqrt{5}\right)$) (c.f. Mammen, 1993). This procedure preserves the third moment of the bootstrap distribution but at the expense of an inflated kurtosis which may negatively impact the estimation of the distribution tail probabilities. For this reason, alternative resampling procedures, such as, for instance, the generalised Jackknife (c.f. Gomes et al., 2015), may provide improved performance over the Mammen-based wild *t*-bootstrap. As a comparison of such resampling methodology is beyond the scope of this work, in the following sections, we make the common simplifying assumption that $\{G^{i}{\}}_{i\in M}$ are Gaussian, with unknown spatial and between-‘study condition’ covariance structure, and refer the reader to Efron and Tibshirani (1994) for further discussion of resampling techniques. Here we stress that, whilst the theory presented in Section 2 places no restrictions on the distribution of $\{G^{i}{\}}_{i\in M}$, throughout Section 3$\{G^{i}{\}}_{i\in M}$ are assumed to be Gaussian.

## 4 Simulations

### 4.1 Simulation settings

To assess the correctness and performance of the method, a range of simulations were conducted using synthetic data. Each simulation was designed to investigate how the empirical coverage (i.e. the proportion of simulation instances in which the inclusion ${\hat{F}}_{c}^{+}\subseteq F_{c}\subseteq {\hat{F}}_{c}^{-}$ held) was affected by various secondary factors of practical interest. In this section, we describe three simulations designed to investigate how the methods’ performance was influenced by: (1) the degree of overlap between excursion sets, (2) the presence of correlation between the noise fields for different study conditions, and (3) the magnitude of the spatial rate of change of the signal.

In all simulations, the model used to generate the synthetic data took the form:


(5) $$Y^{i}(s)=\mu^{i}(s)+\epsilon^{i}(s),\;\epsilon^{i}(s)∼N(0,\sigma^{i}(s)I_{n}),\;\text{ for each}\;i\in M,$$

where *n* was allowed to vary from 40 to 500 in increments of 20. To induce spatial correlation between observations, $\epsilon^{i}(s)$ was smoothed using an isotropic Gaussian filter with a full-width half maximum (FWHM) of 3 pixels. The aim, across all simulations, was to assess the performance of the method when employed for conjunction inference on the true signal $\{\mu^{i}{\}}_{i\in M}$. This setting corresponds to asking research questions of the form ‘Where do *all* of the $\{\mu^{i}{\}}_{i\in M}$ exceed *c*?’.

**Figure 4.**
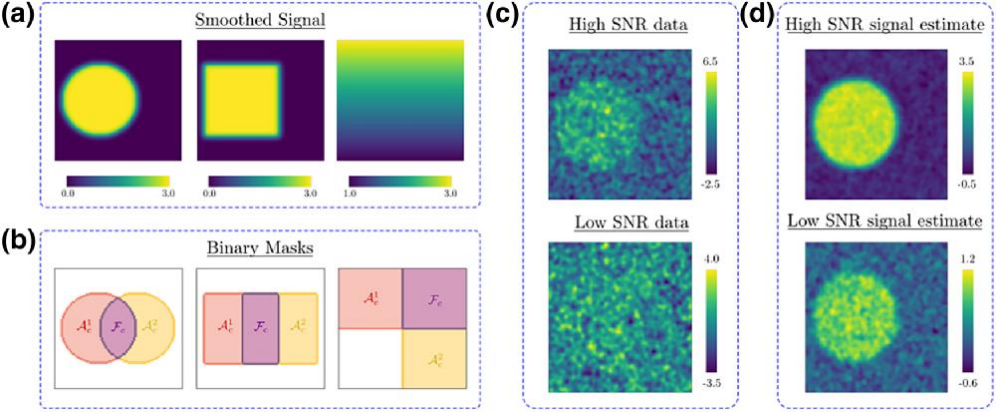
(a) The circular, square, and ramp high-SNR signals used for $\mu^{1}$ in Simulations 1, 2, and 3, respectively. (b) Binary images for $A_{c}^{1},A_{c}^{2}$, and $F_{c}$, for Simulations 1, 2, and 3. (c) Instances of the high- and low-SNR data generated for study condition 1 in Simulation 1. (d) The estimated mean signal for study condition 1, ${\hat{\mu }}^{1}$, in Simulation 1, generated using n=120 high-SNR, and low-SNR, simulated data instances.

The mechanism used to generate the true signal, $\{\mu^{i}{\}}_{i\in M}$, varied across simulations. In Simulations 1 and 2, $\{\mu^{i}{\}}_{i\in M}$ were generated using two binary images of circles and squares, respectively, positioned in a ‘Venn diagram’ arrangement. Each binary image was scaled by a predefined amount and then smoothed using an isotropic Gaussian filter with an FWHM of 5 pixels. The resulting signal for Simulation 1 strongly resembled that depicted in Figure 2, whilst Simulation 2’s signal resembled Figure 3b in order to test the effect of $\partial^{\left\{1,2\right\}}F_{c}$ having non-zero length. In Simulation 3, $\mu^{1}$ and $\mu^{2}$ were simulated as a horizontal and vertical linear ramp, respectively, with predefined gradients. For each simulation, $\mu^{1}$ is depicted in Figure 4a, with corresponding binary images for $A_{c}^{1}$, $A_{c}^{2}$, and $F_{c}$ shown in Figure 4b.

Each simulation was performed twice, once using noisier ‘low SNR’ synthetic data and again using less noisy ‘high-SNR’ synthetic data. To generate the high-SNR synthetic data for Simulations 1 and 2, all simulated $\{\mu^{i}{\}}_{i\in M}$ were scaled by a factor of 3 prior to smoothing. This data generation process is identical to that employed in Bowring et al. (2019), in which it is noted that synthetic data generated using these parameters strongly resembles that of an fMRI analysis when voxels are of dimension $2\;{\mathrm{mm}}^{3}$. In Simulation 3, for the high-SNR data, the linear ramps were initially simulated with a gradient of 1 per 50 pixels. Across all simulations, the low-SNR data was generated by reducing the signal magnitude of the high-SNR data by a factor of 4. In all simulations, thresholds of c=1/2 and c=2 were employed for the low-SNR data and high-SNR data, respectively. Data realisations and signal estimates employed for Simulations 1–3 are illustrated in Figure 4c and d, respectively.

As Simulation 1 aimed to investigate the method’s performance as the overlap between excursion sets increased, in this simulation, the distance between the centre of the circles was varied, ranging from 0 pixels (full overlap) to 50 pixels (barely overlapping) in increments of 2 pixels. Similarly, as Simulation 2 aimed to assess the effect of correlation between $\epsilon^{1}$ and $\epsilon^{2}$, in this simulation, this correlation was varied between −1 and 1 in increments of 0.1 (in all other simulations, the noise fields were generated independently). As Simulation 3 served to assess how sensitive the empirical coverage was to the rate of change of $\mu^{1}$ and $\mu^{2}$ over space, in this simulation, the initial ramp gradients were divided by a factor of *k*, where *k* was varied from 0.25 to 1.75 in 0.05 increments.

All simulation results were obtained as averages taken across 2,500 simulation instances and compared to the nominal coverage using 95% binomial confidence intervals. In all simulation instances, the image dimensions were (100×100) pixels, CRs were computed for a tolerance level of α=0.05 using 5,000 bootstrap realisations, $\sigma^{i}(s)$ was computed using the ordinary least squares estimator and $\tau_{n}$ was computed as $\tau_{n}=n^{-0.5}$. In each simulation instance, the assessment of whether the inclusion statement, ${\hat{F}}_{c}^{+}\subseteq F_{c}\subseteq {\hat{F}}_{c}^{-}$, held was verified using the interpolation-based methods of Bowring et al. (2019). In this approach, the inclusion statement was deemed to hold if, and only if, the sets of pixels which were identified as ${\hat{F}}_{c}^{-}$, $F_{c}$ and ${\hat{F}}_{c}^{+}$ were appropriately nested and, in addition, interpolation along the boundary $\partial F_{c}$ confirmed that no violations of the inclusion statement had occurred.

A further 12 simulations which investigated the influence of other secondary factors of interest were also conducted. The secondary factors considered by these simulations include: the presence of spatial structure in the noise, the effect of $\partial^{\left\{1\right\}}F_{c}$ and $\partial^{\left\{2\right\}}F_{c}$ having differing lengths, the effect of $\epsilon^{1}$ having a much larger variance than $\epsilon^{2}$, spatial smoothing on $\left\{\mu^{i}\right\}$ and $\left\{\epsilon^{i}\right\}$, raising and lowering the threshold *c*, $\left\{\epsilon^{i}\right\}$ having heavy-tailed non-Gaussian symmetric distributions, and the impact of varying the value of *M*. Also considered was the effect of $\left\{A_{c}^{i}\right\}$ having higher order shared boundary segments of the form depicted in Figure 3f. A full discussion of these simulations is deferred to online Supplementary Results Section S4. In addition, in keeping with the previous literature, tolerance levels of α=0.1 and α=0.2, as well as the substitution of $\partial F_{c}$ for $\partial {\hat{F}}_{c}$ in equation (4), were also considered for simulation (c.f. Bowring et al., 2019, 2021; Sommerfeld et al., 2018). These simulation results may be found in online Supplementary Results Sections S5 and S6.

### 4.2 Simulation results

**Figure 5.**
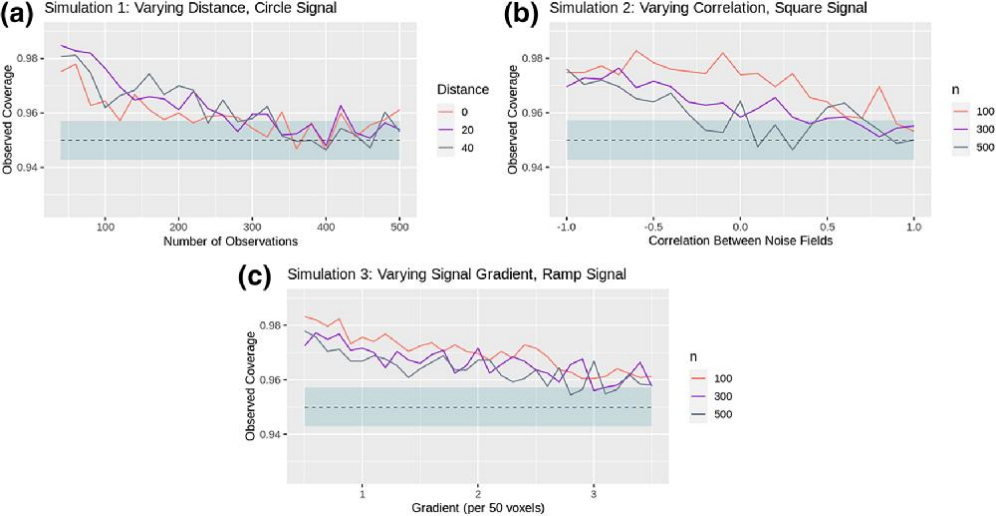
Simulation results generated using the low-SNR synthetic data. Nominal coverage is shown as a dashed line, with a corresponding binomial confidence interval also shaded. All data points are averages taken across 2,500 simulation instances.

For a majority of the simulations conducted, the empirical coverage estimates for the proposed method were tightly distributed around the expected nominal coverage. In particular, for the high-SNR data, across all simulations, the 95% binomial confidence intervals for the proposed method consistently captured the nominal coverage at the predicted rate. However, when the simulations employed the low-SNR data, the proposed CRs method was conservative under certain unfavourable conditions. The low-SNR results for Simulations 1–3 are shown in Figure 5.

For example, in Figure 5a, it can be seen that some over-coverage was observed for Simulation 1 when the data was noisy, and the number of subjects was low. This observation is corroborated by the remaining low-SNR synthetic data simulations (c.f. online Supplementary Results Section S5). However, in all cases, the degree of agreement between the empirical and nominal coverage improved as sample size increased, and no such over-coverage was observed for the equivalent high-SNR simulations. This observation matches expectation, as Theorem 2.1 holds only asymptotically, and is supported by the previous literature on CRs, in which similar results were observed for the single-‘study condition’ setting (c.f. Bowring et al., 2019; Sommerfeld et al., 2018).

The presence of strong negative correlation between the study conditions and the rate at which the signal varied across space both also appeared to influence the methods’ performance (c.f. Figure 5b and c, respectively). In both instances, it is likely that the observed over-coverage is due to difficulties in estimating the true location of the boundary $\partial F_{c}$, as each of these factors make it harder to identify the locations at which small changes in $\min_{i\in M}\mu^{i}$ occur. When high-SNR data was used in the place of low-SNR data, both of these factors had a substantially reduced impact on the empirical coverage.

Despite the above observations, the simulation results overwhelmingly supported the claim that the proposed method is robust to a range of secondary factors. Included in the list of such factors are: variation in the shape and size of $F_{c}$, spatial correlation in the noise, variation in the lengths of individual boundary segments, between-‘study condition’ correlation, variation in the magnitude of the noise, high spatial smoothness of the signal and noise, raising and lowering the threshold, heavy-tailed noise distributions, large numbers of study conditions, and situations in which boundary segments are shared by many excursion sets. For further detail, see online Supplementary Results Sections S5 and S6.

In terms of time efficiency, the wild *t*-bootstrap provided extremely fast computational performance. For instance, using an Intel(R) Xeon(R) Gold 6126 2.60 GHz processor with 16 GB RAM, and averaging over the 2,500 simulation instances conducted for n=500 high-SNR observations, in Simulation 1, the time taken to generate CRs for a circle separation of 20 pixels was 5.98 s. For a comprehensive summary of computation times, see online Supplementary Results Section S7.

## 5 Real data application

In Section 1, an fMRI application based on using data from the HCP dataset (Van Essen et al., 2013) was presented. Here, we provide further detail for this application. In this example, task-fMRI data were collected as part of a block design from 80 healthy, unrelated, young adults as part of a working memory task that had four distinct components, each using a different stimuli type: pictures of places, tools, faces, and body parts. In online Supplementary Results Section S3, we provide a brief overview of the imaging acquisition protocol, task paradigm, preprocessing stages and first-level analysis employed for generating this dataset (for further detail, see Barch et al., 2013; Glasser et al., 2013). Following first-level analysis, the data consisted of 4 images for each of the 80 subjects, measuring the %BOLD response to each of the 4 stimuli types. In this example, our interest lies in identifying, at the group-level, which regions of the brain are associated with working memory regardless of stimuli type. In other words, ‘Which regions are active in response to *all* four working memory stimuli types?’.

As this work has focussed on two-dimensional excursion sets, a single slice of the brain was chosen for analysis (z=46mm, covering portions of the frontal gyrus involved in working memory). For each stimuli type, an n=80 group-level LM was constructed. Using the proposed method, CRs were then generated at the 5% confidence level to assess where the group-level percentage BOLD change exceeded 1% for *all* four stimuli types. To compare the proposed method to standard fMRI inference procedures, a group-level contrast was also generated using the Big LM toolbox for each of the four stimuli types. In addition, single-‘study condition’ CRs were also generated using the methods of Sommerfeld et al. (2018) for each of the four stimuli types.

The results are those shown in Figure 1. The conjunction inference identified several localised regions, including the superior frontal gyrus, which is well known for its involvement in working memory (Alagapan et al., 2019; Boisgueheneuc et al., 2006; Vogel et al., 2016), and the angular gyri, which are well known to be involved in memory retrieval, attention, and spatial cognition (Bréchet et al., 2018; Seghier, 2013).

Whilst the CRs for conjunction inference illustrated in Figure 1 exhibit a clear resemblance to the four contrast images, we note that the red set, ${\hat{F}}_{c}^{+}$, is very small in comparison to the blue set, ${\hat{F}}_{c}^{-}$, which is large and diffuse. This observation conveys important information about the spatial variability of the regions identified. In particular, it can be seen that, in the regions immediately surrounding the superior frontal gyrus and angular gyri, there is a much higher resemblance between the blue (${\hat{F}}_{c}^{-}$) and yellow (${\hat{F}}_{c}$) sets than is seen across the rest of the brain. This resemblance provides an insight into the degree of spatial variation present surrounding these regions. In this case, the strength and localisation of this resemblance suggest that the spatial variability of ${\hat{F}}_{c}$ is less severe in and around the aforementioned anatomical regions than it is across the rest of the brain. It may be concluded that the estimated yellow ‘blobs’ corresponding to the superior frontal gyrus and the angular gyri have been more reliably localised than the other yellow ‘blobs’ appearing in the image.

In general, the single-‘study condition’ CRs can be seen to be much larger than those observed for conjunction inference. This observation matches expectation as the overlap of the four excursion sets is smaller than each set individually. In addition, in this example, the conjunction method employed the entire experimental design for analysis, as opposed to the single-‘study condition’ method which employed only the data concerning the stimuli of interest. It is therefore expected to have a higher statistical power and, thus, tighter confidence bounds.

## 6 Conclusion and discussion

In this work, we have produced a method for generating CRs for intersections and unions of multiple excursion sets. The CRs generated by the method generalise the notion of confidence intervals to arbitrary spatial dimensions and possess the usual frequentist interpretation that, were the 0.95 procedure repeated on numerous samples, the proportion of CRs, ${\hat{F}}_{c}^{+}$ and ${\hat{F}}_{c}^{-}$, that correctly enclose $F_{c}$ in the manner depicted in Figure 2c, would tend to be 0.95. Such CRs serve as an indicator of the reliability of ${\hat{F}}_{c}$ as an estimate of $F_{c}$.

We stress here that, whilst ${\hat{F}}_{c}$ is equal to the intersection of $\{{\hat{A}}_{c}^{i}{\}}_{i\in M}$, the CRs ${\hat{F}}_{c}^{\pm }$ are not the intersections of the single-‘study condition’ CRs $\{{\hat{A}}_{c}^{\pm ,i}{\}}_{i\in M}$ which would be obtained using the methods of Sommerfeld et al. (2018). In other words, the intersection of the yellow sets in the four bottom-left panels of Figure 1 is equal to the yellow set in the fifth, but the corresponding statement does not hold for the blue or red sets. This claim follows from the fact that $\{{\hat{A}}_{c}^{\pm ,i}{\}}_{i\in M}$ are defined using separate quantile estimates from different bootstraps and can be heavily influenced by the behaviour of $\{\partial A_{c}^{i}{\}}_{i\in M}$ in spatial regions far from $F_{c}$. In fact, treating the naive intersection of the (1−α) CRs $\{{\hat{A}}_{c}^{\pm ,i}{\}}_{i\in M}$ as (1−α) CRs for $F_{c}$ is not a valid method in general and can result in undesirable asymptotic coverage lying anywhere inside the range [1−Mα,1]. This claim is further discussed in online Supplementary Theory Section S6 and verified in Simulations 14 and 15 of the online Supplementary Results document, which show that naive intersections can provide over- and under-coverage, respectively. To our knowledge, the only valid approach for generating CRs for conjunction inference is that outlined in this work.

One potential limitation of the specific methods of Section 3 is that they allow for CRs to be generated only when the target functions, $\{\mu^{i}{\}}_{i\in M}$, are linear combinations of regression parameter estimates. As noted by Bowring et al. (2021), it is often preferable for an analysis to consider standardised effect sizes, such as Cohen’s *d* (Cohen, 2013) or Hedges’ *G* (Hedges, 1981), instead of regression parameter estimates, as standardised effect sizes allow for information about statistical power to be incorporated into the analysis. To this end, Bowring et al. (2021) outlined an approach for generating CRs (in the single-‘study condition’ setting) for images of Cohen’s *d* effect sizes. Here, we suggest that a potential avenue for future work is to combine the methods of Bowring et al. (2021) and those proposed in this work to allow for conjunction, or disjunction, inference to be performed on standardised effect sizes rather than regression parameter estimates.

It must also be noted that the wild *t*-bootstrap has only been shown to consistently estimate quantiles of the supremum in equation (2) when the $\left\{G^{i}\right\}$ are Gaussian random fields satisfying Assumption 3.2.1 (c.f. Telschow & Schwartzman, 2021). As discussed in Section 3.2, it has been suggested that this approach may also be used for estimating suprema quantiles for symmetric non-Gaussian random fields, and further adapted to non-symmetric cases by substituting the Rademacher variables for Mammen variables. However, to our knowledge, the consistency of such approaches has not yet been shown to hold theoretically. Our preliminary simulations have indicated that consistency may hold in the symmetric case (c.f. online Supplementary Results Simulation 13), and thus we suggest theoretical exploration of this topic may provide a basis for future investigation.

Another limitation noted here is that, whilst our proposed method theoretically works for data of arbitrary dimensions, the simulations of Section 4 verify the performance of our method only for two-dimensional data. Whilst it is typical for many practical applications to focus on two-dimensional data (e.g. climate maps, surface images), higher dimensional datasets are abundant in a wealth of disciplines such as brain imaging and astrological mapping. For this reason, we intend to investigate further the performance of the method in higher dimensions in future work.

A possible direction for future exploration is to investigate the applicability of the method within the framework of latent Gaussian models. Such models seek to explain non-Gaussian outcomes as a combination of Gaussian processes transformed via a link function. By using the proposed method, it may be possible to assess the regions of space in which a combination of latent processes predicted the outcome. If successful, such an approach would have high utility for the analysis of spatial data with a count or binary structure, making it a promising direction for further research.

## Supplementary Material

qkad104_Supplementary_Data

## Data Availability

Data were provided by the Human Connectome Project, WU-Minn Consortium (Principal Investigators: David Van Essen and Kamil Ugurbil; 1U54MH091657) funded by the 16 NIH Institutes and Centers that support the NIH Blueprint for Neuroscience Research; and by the McDonnell Center for Systems Neuroscience at Washington University. The code used to obtain the results of Sections 4 and 5 is freely available and may be found at: https://github.com/TomMaullin/ConfSets
