# Supplementary material for: Spatial confidence regions for combinations of excursion sets in image analysis
Source: J R Stat Soc Series B Stat Methodol. 2023 Sep 21;86(1):177–93. doi: 10.1093/jrsssb/qkad104 (PMC10852994; doi:10.1093/jrsssb/qkad104)
Supplement: qkad104_Supplementary_Data [file qkad104_supplementary_data.zip › Supplementary_Results.pdf]

Spatial Confidence Regions for Combinations of Excursion Sets in Image Analysis

Supplementary Results

Thomas Maullin-Sapey[1,*], Armin Schwartzman[2,3], and Thomas E. Nichols[1]

[1] Big Data Institute, Li Ka Shing Centre for Health Information and Discovery, Oxford, UK

[2] Division of Biostatistics, University of California, San Diego, CA, USA

[3] Halicioğlu Data Science Institute, University of California, San Diego, USA

[*]Corresponding Author: Thomas, TM, Maullin-Sapey, Thomas.Maullin-Sapey@bdi.ox.ac.uk

Submission for Journal of the Royal Statistical Society Series B (Statistical Methodology)

S1 Overview

This document contains supplementary results for the paper 'Spatial Confidence Regions for Combinations of Excursion Sets in Image Analysis'. The following sections provide a full account of the simulations and real data example described in the main text. However, unlike in the main text, here we report empirical coverage simulation results for 3 significance levels ($\alpha = 0.05, 0.1$ and 0.2), 15 simulations and 2 different approaches to computing realisations of H during the Wild $t-$bootstrap.

In particular, as detailed in Section 3.2 of the main text, bootstrap samples for H are obtained using the below expression;

$$\tilde{H} = \max_{\phi \in \mathcal{P}^+(\mathcal{M})} \left(\sup_{s \in \partial^\phi \mathcal{F}_c} \big| \min_{i \in \phi} (\tilde{G}^i(s)) \big| \right). \tag{S1}$$

The simulation results detailed in this document consider 2 approaches to evaluating the above supremum taken over $\partial^\phi \mathcal{F}_c$. In the first approach, the location of $\partial^\phi \mathcal{F}_c$ is evaluated using the underlying mechanism which was employed for synthetic data generation. In the second approach, the location of $\partial^\phi \mathcal{F}_c$ is estimated from the synthetic data itself. In the following sections, the former approach is referred to as using the 'true boundary', whilst the latter is referred to as using the 'estimated boundary'. The simulation results in the main text were generated using $\alpha = 0.05$ with suprema evaluated using $\partial \hat{\mathcal{F}}_c$ (i.e. the 'estimated boundary'). These settings were chosen for inclusion in the main text as, in practice, $\partial \mathcal{F}_c$ (i.e. the 'true boundary') is unknown, and $\alpha = 0.05$ is a popular choice of tolerance level.

This document is organized as follows. Section S2 provides practical details for implementation of the method, alongside pseudocode. Next, Section S3 provides an overview of the process used to obtain the real data employed in Section 5 of the main text. After this, Section S4 describes the 12 simulations that were not outlined in the main text. Section S5 provides empirical coverage results obtained by using the estimated boundary to evaluate Equation (S1), with Sections S5.1, S5.2 and S5.3 providing results for $\alpha = 0.2, 0.1$ and 0.05, respectively. Similarly, Section S6 provides empirical coverage results obtained by using the true boundary to evaluate Equation (S1) with Sections S6.1, S6.2 and S6.3 providing results for $\alpha = 0.2$, 0.1 and 0.05, respectively. Following this, Section S7 provides an overview of the computation times observed for each of the simulations.

S2 Implementation Details

S2.1 Modelling on a Discrete Lattice

Throughout the main text, we assumed that the target and estimator functions are defined on a compact region of continuous space, S. In practice, however, observations are sampled on a discrete subset of S, typically a lattice. As noted by Bowring et al. [2019], great care must be exhibited when data are sampled on a lattice, as interpolation must be performed to estimate the location of the boundary $\partial\hat{\mathcal{F}}_c$.

The interpolation process begins by first identifying pairs of neighbouring gridpoints, $s_{\mathcal{I}}$ and $s_{\mathcal{O}}$, which lie inside and outside $\hat{\mathcal{F}}_c$, respectively. For each pair of neighbouring gridpoints, interpolation weights, which represent the location of $\partial\hat{\mathcal{F}}_c$, are then obtained for each study condition using the estimators $\{\hat{\mu}^i_n\}_{i\in\mathcal{M}}$. Following this, in each bootstrap instance, the interpolation weights are used to obtain interpolated bootstrap samples $\{\tilde{G}^i\}_{i\in\mathcal{M}}$ for each pair of gridpoints, $(s_{\mathcal{I}}, s_{\mathcal{O}})$. Using the interpolated bootstrap samples $\{\tilde{G}^i\}_{i\in\mathcal{M}}$, interpolated bootstrap samples of H, $\tilde{H}$, are obtained using Equation (S1). Further detail of this approach may be found in Bowring et al. [2019]. We note here that, to obtain $\tilde{H}$ using Equation (S1), it is also necessary to identify the boundary segments $\{\partial^\phi\hat{\mathcal{F}}_c\}_{\phi\in\mathcal{P}^+(\mathcal{M})}$. In practice, this may be achieved via careful consideration of the values of $\{\hat{\mu}^i(s_{\mathcal{O}})\}_{i\in\mathcal{M}}$ for each pair of neighbouring gridpoints, $(s_{\mathcal{I}}, s_{\mathcal{O}})$, in order to identify which of the boundaries $\{\partial\mathcal{A}^i_c\}_{i\in\mathcal{M}}$ lie between $s_{\mathcal{O}}$ and $s_{\mathcal{I}}$. This process was employed to obtain the results of the following sections.

S2.2 Pseudocode

Algorithm 1: Pseudocode to generate CRs for LM Conjunction Inference

for *each study condition, $i \in \mathcal{M}$, and every gridpoint, s* **do**

  Compute estimator function, $L^{i'}\hat{\beta}^i(s) = L^{i'}(X^{i'}\Sigma^i(s)^{-1}X^i)^{-1}X^{i'}\Sigma^i(s)^{-1}Y^i(s)$.

  Compute decorrelated residual vector, $R^i(s) = [\Sigma^i(s)]^{-\frac{1}{2}}(Y^i(s) - X^i\hat{\beta}^i(s))$.

end

Compute intersection set $\hat{\mathcal{F}}_c$ as the set of gridpoints where $\min_{i\in\mathcal{M}} L^{i'}\hat{\beta}^i(s) \geq c$ and identify boundary segments $\{\partial^\phi\hat{\mathcal{F}}_c\}_{\phi\in\mathcal{P}^+(\mathcal{M})}$.

for *each bootstrap instance, instance b* **do**

  Generate n Rademacher variables, $\{r_l\}_{l\in\{1,\dots,n\}}$.

  for *each boundary segment, $\partial^\phi\hat{\mathcal{F}}_c$* **do**

    for *every neighbouring gridpoint of $\partial^\phi\hat{\mathcal{F}}_c$, s, and each study condition, $i \in \phi$* **do**

      Compute bootstrap residual mean, $\overline{R^i}(s) = \sum_{l=1}^n \frac{r_l R^i_l(s)}{n}$.

      Compute bootstrap residual standard error, $\hat{\sigma}^{*,i}(s) = \sqrt{\frac{\sum_{l=1}^n (r_l R^i_l(s) - \overline{R^i}(s))^2}{n-1}}$.

      Compute bootstrap instance of $G^i(s)$, $\tilde{G}^i(s) = n^{-\frac{1}{2}}\sum_{l=1}^n r_l \frac{R^i_l(s)}{\hat{\sigma}^{*,i}(s)}$.

    end

    for *every neighbouring pair of gridpoints lying inside and outside of $\partial^\phi\hat{\mathcal{F}}_c$, $(s_\mathcal{I}, s_\mathcal{O})$, and each study condition, $i \in \phi$* **do**

      Interpolate $\tilde{G}^i(s_\mathcal{I})$ and $\tilde{G}^i(s_\mathcal{O})$ to obtain bootstrap instance $\tilde{G}^i(s_\mathcal{I}, s_\mathcal{O})$.

    end

  end

  Compute bootstrap instance of H, $\tilde{H}_b = \max_{\phi\in\mathcal{P}^+(\mathcal{M})}(\max_{\{(s_\mathcal{I},s_\mathcal{O})\}} |\min_{i\in\phi}\tilde{G}^i(s_\mathcal{I}, s_\mathcal{O})|)$.

end

Estimate a as the $(1-\alpha)^{th}$ quantile of the bootstrap instances $\{\tilde{H}_b\}$.

Construct $\hat{\mathcal{F}}_c^+$ and $\hat{\mathcal{F}}_c^-$ using the estimate of a.

S3 Real Data Methods

In this section, we detail the imaging acquisition protocol, task paradigm, preprocessing stages and first-level analysis used to generate the dataset employed in Section 5 of the main text. In the experiment, each subject performed two tasks spread across two runs where each run contained four blocks (one for each stimuli type, e.g. faces, places, tools and body parts). During each block, the subject undertook either a 2-back memory task or a 0-back control task. The experimental design was arranged such that, in each run, two blocks were designated to the 2-back memory task, and two blocks were designated to the 0-back control task. In total, there were eight blocks across the two runs, one for every combination of task and stimuli type (e.g. '0-back and faces', '2-back and places',...). All image acquisitions were obtained using a 32 channel head coil on a modified $3T$ Siemans Skyra scanner via a gradient-echo EPI sequence with TR= 720ms, TE= 33.1ms, 2.0mm slice thickness, 72 slices, 2.0mm isotropic voxels, 208mm$\times$180mm FOV, and a multi-band acceleration factor of 8.

Using FSL's 'FMRIB's Improved Linear Model' (FILM) toolbox (Woolrich et al. [2001]), for each of the 80 subjects, a subject-level analysis which included observations for all four stimuli types across both tasks was conducted. In each subject-level analysis, eight explanatory variables (one for each task-stimuli combination), alongside temporal derivative confound terms, were regressed onto Blood Oxygenation Level Dependent (BOLD) response. Each of the eight task-stimuli explanatory variables was convolved with FSL's default double-gamma haemodynamic response function and temporally filtered using a high pass frequency cutoff of 200s. The functional data were subjected to identical temporal filtering, as well as prewhitening to remove autocorrelation.

Following the subject-level regression, a Contrast Parameter Estimate (CoPE) map was generated for each of the four stimuli types. Each CoPE map represented the average difference in BOLD response between a subject performing the $2-$back task for a given stimuli type and the corresponding $0-$back task. The 320 resultant CoPE maps (one for each of the four stimuli types across 80 subjects) were then registered to MNI space. The resultant 320 registered CoPE maps formed the dataset employed in the analysis of Section 5 of the main text.

S4 Simulation Settings

In Section 4.1 of the main text, three simulations were described in full detail, and reference was made to a further 12. Here, we describe the remaining 12 simulations (Simulations 4-15), the results of which are presented in Sections S5 and S6.

Simulation 4 was conducted to ensure that the method was not affected by the degree to which two sets shared a common boundary (e.g. the length of $\partial^{\{1,2\}}\mathcal{F}_c$). For this reason, Simulation 4 was similar to Simulation 1 but employed squares instead of circles for data generation. This ensured that $\mathcal{A}_c^1$ and $\mathcal{A}_c^2$ shared boundary segments at the top and bottom of $\mathcal{F}_c$ (c.f. Fig. S1 (a)). Simulation 5 aimed to assess how robust the method was when spatial structure was present in the noise. In this simulation, following the example of Bowring et al. [2019], ϵ^2 was simulated using a heterogeneous linear ramp (c.f. Fig. S1 (b)).

Simulation 6 aimed to assess how robust the empirical coverage was to the boundary segments $\partial^{\{1\}}\mathcal{F}_c$ and $\partial^{\{2\}}\mathcal{F}_c$ having differing lengths. This simulation was similar to Simulation 4, except the height and width of $\mathcal{A}_c^2$ were reduced by a factor of 2/3. Reducing the height and width of $\mathcal{A}_c^2$ in this manner ensured that $\partial^{\{2\}}\mathcal{F}_c$ increased in length as the squares were moved closer together whilst the length of $\partial^{\{1\}}\mathcal{F}_c$ remained constant. Simulation 7 aimed to assess how increasing the variance of ϵ^2 affected the empirical coverage. This simulation employed squares for generating μ^1 and μ^2. In this simulation, the standard deviation of ϵ^1 was held fixed at 1 whilst the standard deviation of ϵ^2 varied from 1 to 3 in 0.2 increments.

The first seven simulations consider only the case in which $M = 2$. To assess the method's performance for higher values of M, Simulation 8 allowed M to vary from 2 to 5. For this simulation, the functions $\mu^1, ..., \mu^M$ were generated using overlapping arrangements of circles similar to that employed by Simulation 1 (c.f. Fig. S1 (c)). In Simulation 8, all non-empty boundary segments, $\partial^{\phi}\mathcal{F}_c$, satisfied $|\phi| \leq 2$. To validate our method in the situation in which the boundary segments result from higher-order combinations of excursion set boundaries (i.e. $|\phi| > 2$), Simulation 9 was run. In this simulation, M was held fixed at $M = 3$, and data was generated using two large squares with a smaller square nested perfectly within their intersection (see Fig. S1 (d)). This setup ensured that $\partial^{\{1,2,3\}}\mathcal{F}_c$ was non-empty. Unlike in the other simulations, only the number of observations was varied for Simulation 9.

Simulations 10 and 11 were designed to assess how the method was impacted by varying

the spatial smoothness of $\{\mu^i\}$ and $\{\epsilon^i\}$ respectively. In both settings, square binary masks were used to generate the signal and the respective smoothness was varied from 0 to 8 in 0.2 increments. Notably, both simulations included the case in which the smoothness was 0, thus violating Assumption 2.2.2 in the main text. It should be noted that we are not asserting that the method will function in this case; rather, it was included in the simulations for purely empirical considerations.

Simulation 12 aimed to assess the impact of varying the threshold c. To do so, square binary masks were employed for the signal, and c was varied from 0 to 0.8 in increments of 0.05 in the low-SNR setting, and 0 to 3.2 in increments of 0.2 in the high-SNR setting. In both settings, the signal had a plateau at the level $c = 0$ with signal peaks occurring at $c = 0.75$ and $c = 2.8$ for the low and high-SNR settings, respectively. As Assumption 2.2.3 was violated at these thresholds, it follows that the method is expected to fail for such cases.

Simulation 13 was designed to assess the performance of the method when the noise distribution is symmetric, but not Gaussian. This simulation employed circular binary masks for the signal and, to ensure that the noise distribution had non-Gaussian tails, the $\{\epsilon^i\}$ were generated as a $50 : 50$ Gaussian noise mixture of $N(0, 1)$ and $N(0, \sigma^2)$ for σ values ranging from 0.8 to 4.2 in increments of 0.4. As discussed in Section 3.2 of the main text, the wild t-bootstrap is expected to perform well in such instances, but its asymptotic consistency has only been shown under Assumption 3.2.1 and Gaussianity.

Simulation 14 replicated Simulation 1, but with a different objective. It aimed to evaluate the observed coverage when CRs were obtained by naively intersecting the "single-study condition" CRs generated using the methods of Sommerfeld et al. [2018]. Simulation 15 also aimed to evaluate the performance of such "naively intersected" CRs. To do so, it employed two horizontal ramps for μ^1 and μ^2. The ramps were generated in opposing directions with gradients 1 and -1 per 50 pixels, respectively, and were raised and lowered to ensure that the distance between the boundaries $\partial^{\{1\}}\mathcal{F}_c$ and $\partial^{\{2\}}\mathcal{F}_c$ varied from 10 to 100 pixels in increments of 5 pixels. In Supplementary Theory Section S6, it is argued that the naive intersections approach is not expected to provide nominal coverage, but instead can give arbitrary coverage anywhere within the range $[1 - M\alpha, 1]$. Simulations 14 and 15 were specifically designed to demonstrate that this approach could result in over-coverage in the range $(1 - \alpha, 1]$ and under-coverage in the range $[1 - M\alpha, 1 - \alpha)$, respectively.

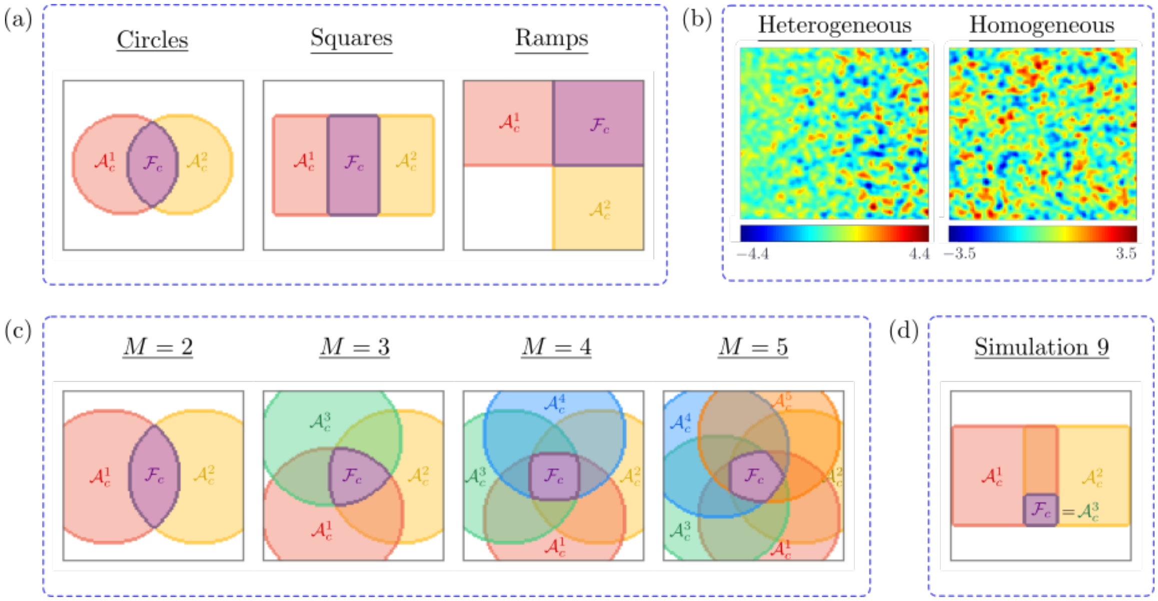

Figure S1: Binary images, alongside example noise realizations, employed for generating synthetic data. (a) The circular binary images, employed for Simulations 1, 13 and 14, the square binary images, employed for Simulations 2, 4, 5, 7 and 10 − 12, and the binary images that correspond to the ramp signal, employed for Simulation 3 (Simulation 15 also employed similar horizontal ramps). (b) A realization of ϵ^2 generated using the heterogeneous ramp employed in Simulation 5, compared to the homogeneous noise generated for Simulations 1-4 and 6-15. (c) The circular binary images employed for varying the value of M in Simulation 8. (d) The nested square binary images employed for Simulation 9.

S5 Empirical Coverage Along the Estimated Boundary

In this section, we provide the simulation results that were obtained by using the estimated boundary, $\partial\hat{\mathcal{F}}_c$, to evaluate the suprema which appears in Equation (S1). Broadly speaking, the results in the following sections concur that, when the suprema appearing in Equation (S1) was evaluated using $\partial\hat{\mathcal{F}}_c$, the proposed method was robust to a range of secondary factors. Such factors include; signal shape, degree of overlap between excursion sets, spatial structure in the noise, correlation between the error fields, the coinciding of multiple excursion set boundaries, the intersection of excursion sets with the boundary of S, the number of study conditions, the value of the threshold c and the smoothness of the signal and noise (provided that the assumptions of the main text are not violated). Notably, Simulation 13 also showed that the proposed method achieves nominal coverage not only under a Gaussian noise distribution, but also under symmetric Gaussian mixture distributions.

Notably, the simulations also showed that the method was sensitive to several factors of interest. These were; the lowering of the signals' spatial gradient, the severity of the noise in the data, the lowering of the number of observations, and the lowering of the tolerance level, α. Of these factors, only the latter was not discussed in the main text. Previous authors have argued that this observation may be due to violations of the inclusion statement, $\hat{\mathcal{F}}_c^+ \subseteq \mathcal{F}_c \subseteq \hat{\mathcal{F}}_c^-$, being miscounted as a result of resolution issues (c.f. Sommerfeld et al. [2018] and Bowring et al. [2019]). As might be expected, it can also be seen from Simulations 10 and 12 that the method failed to obtain nominal coverage if Assumptions 2.2.2 and 2.2.3 were not satisfied, respectively. Furthermore, as predicted by Supplementary Theory Section S6, niave intersection of the single-'study condition' CRs that are generated using the methods of Sommerfeld et al. [2018] failed to provide nominal coverage, and instead gave over- and under-coverage for Simulations 14 and 15, respectively.

Excluding the settings in which Assumptions 2.2.2 and 2.2.3 were violated, it can be seen that all of the factors which negatively impacted the method's performance tended to cause conservativeness in the generation of CRs (e.g. the inclusion statement, $\hat{\mathcal{F}}_c^+ \subseteq \mathcal{F}_c \subseteq \hat{\mathcal{F}}_c^-$, held "too often"). This observation matches the findings of Sommerfeld et al. [2018] and Bowring et al. [2019], and is likely explained by the fact that the estimated boundary segments, $\{\partial^\phi\hat{\mathcal{F}}_c\}_{\phi\in\mathcal{P}^+(\mathcal{M})}$, tend to be longer than the true boundary segments,

$\{\partial^\phi \mathcal{F}_c\}_{\phi\in\mathcal{P}^+(\mathcal{M})}$, for high thresholds. The increased length of $\{\partial^\phi \hat{\mathcal{F}}_c\}_{\phi\in\mathcal{P}^+(\mathcal{M})}$ can cause the estimation of $\sup_{s\in\partial^\phi\mathcal{F}_c} |\min_{i\in\phi}(G^i(s))|$ to be biased upwards, which in turn inflates the estimates of the quantiles of H. The consequential upward bias in the estimation of a can be seen to result in conservative estimates for the CRs $\hat{\mathcal{F}}_c^+$ and $\hat{\mathcal{F}}_c^-$.

This section is organized as follows. Section S5.1 provides empirical coverage results for $\alpha = 0.2$, Section S5.2 provides equivalent results for $\alpha = 0.1$ and Section S5.3 provides results for $\alpha = 0.05$. In all plots, nominal coverage is shown as a dotted gray line and a corresponding binomial confidence interval is shaded in blue. All data points displayed are averages taken across 2500 simulation instances, each using 5000 bootstrap realizations.

S5.1 Nominal Coverage: 80%

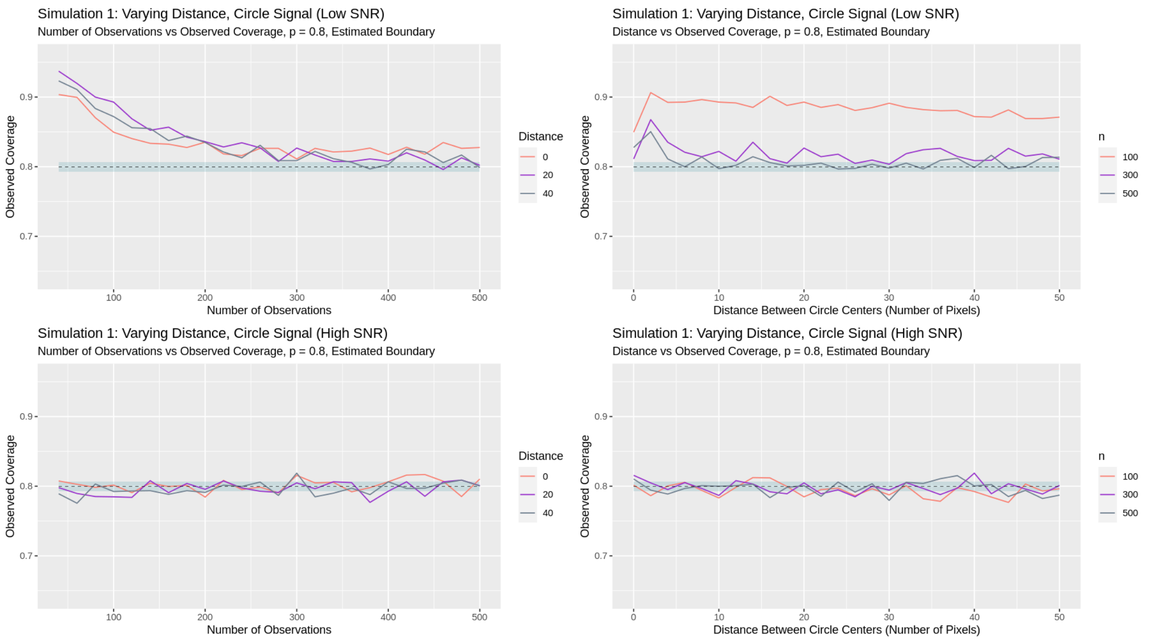

Figure S2: Empirical coverage for simulation 1. Top: Low-SNR synthetic data results. Bottom: High-SNR synthetic data results. Left: Number of observations vs observed coverage, shown for circle separations of $0, 20$ and 40 pixels. Right: Separation between circles vs observed coverage, shown for $n = 100, 300$ and 500.

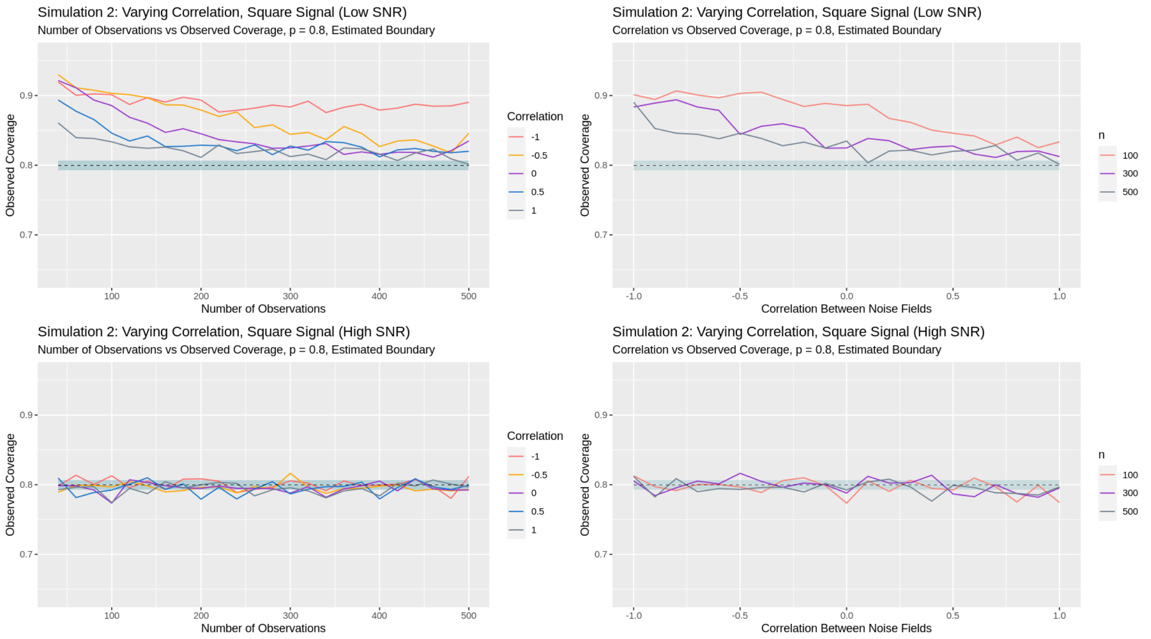

Figure S3: Empirical coverage for simulation 2. Top: Low-SNR synthetic data results. Bottom: High-SNR synthetic data results. Left: Number of observations vs observed coverage, shown for correlations of $-1, -0.5, 0, 0.5$ and 1. Right: Correlation between noise fields vs observed coverage, shown for $n = 100, 300$ and 500.

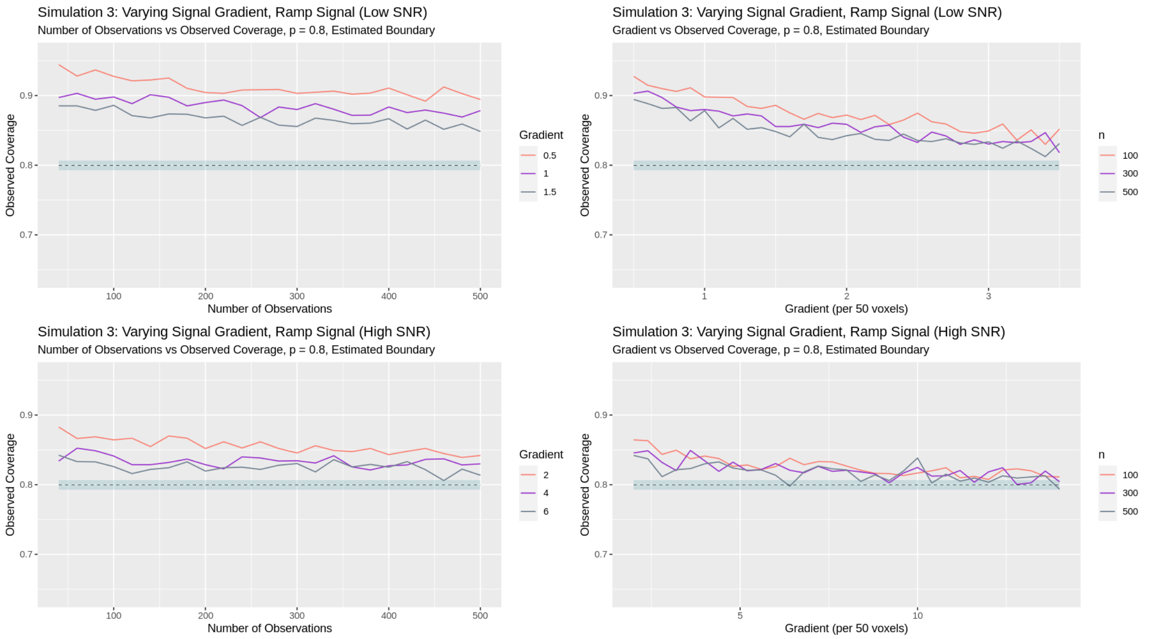

Figure S4: Empirical coverage for simulation 3. Top: Low-SNR synthetic data results. Bottom: High-SNR synthetic data results. Left: Number of observations vs observed coverage, shown for gradients of $0.5, 1$ and 1.5 per 50 pixels (top), and $2, 4$ and 6 per 50 pixels (bottom). Right: Ramp gradient per 50 pixels vs observed coverage, shown for $n = 100, 300$ and 500.

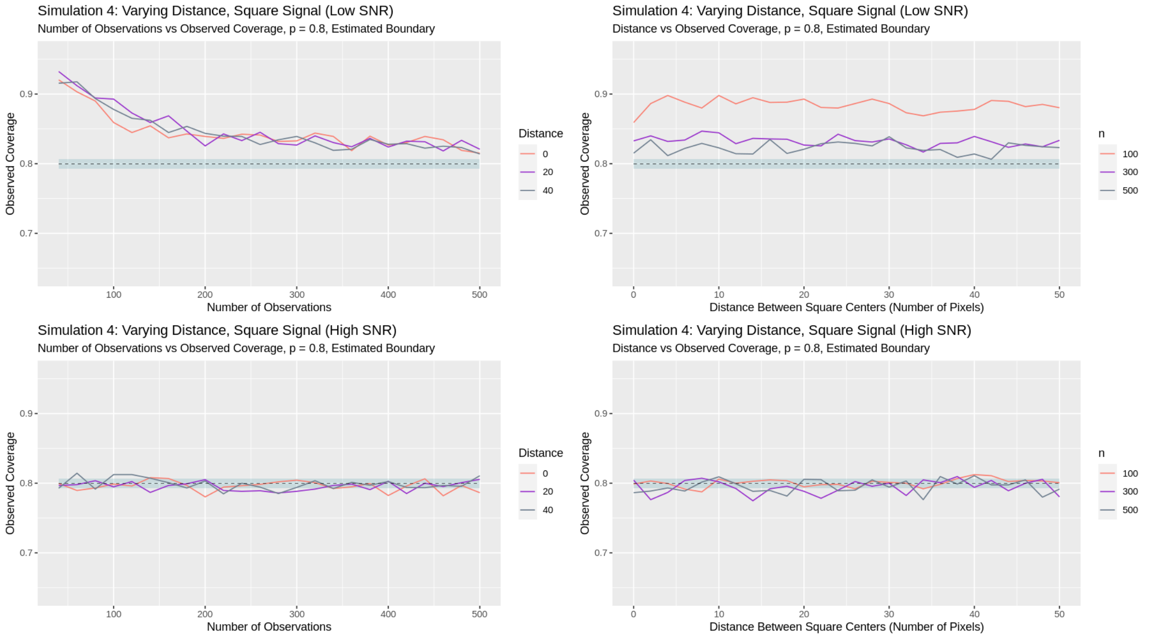

Figure S5: Empirical coverage for simulation 4. Top: Low-SNR synthetic data results. Bottom: High-SNR synthetic data results. Left: Number of observations vs observed coverage, shown for square separations of $0, 20$ and 40 pixels. Right: Separation between squares vs observed coverage, shown for $n = 100, 300$ and 500.

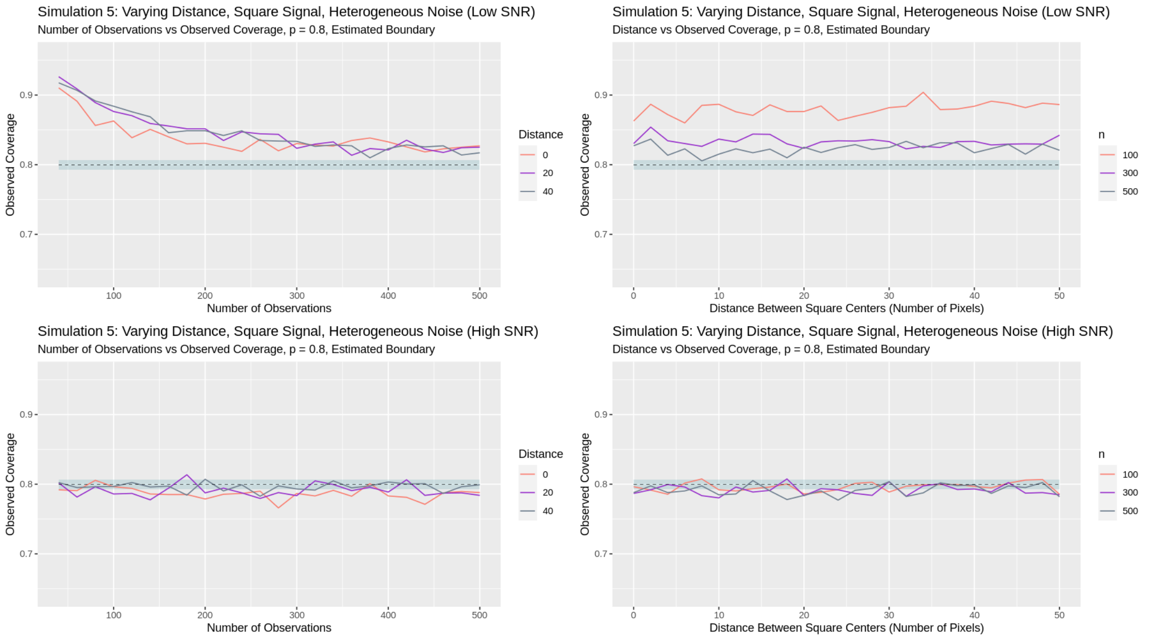

Figure S6: Empirical coverage for simulation 5. Top: Low-SNR synthetic data results. Bottom: High-SNR synthetic data results. Left: Number of observations vs observed coverage, shown for square separations of $0, 20$ and 40 pixels. Right: Separation between squares vs observed coverage, shown for $n = 100, 300$ and 500.

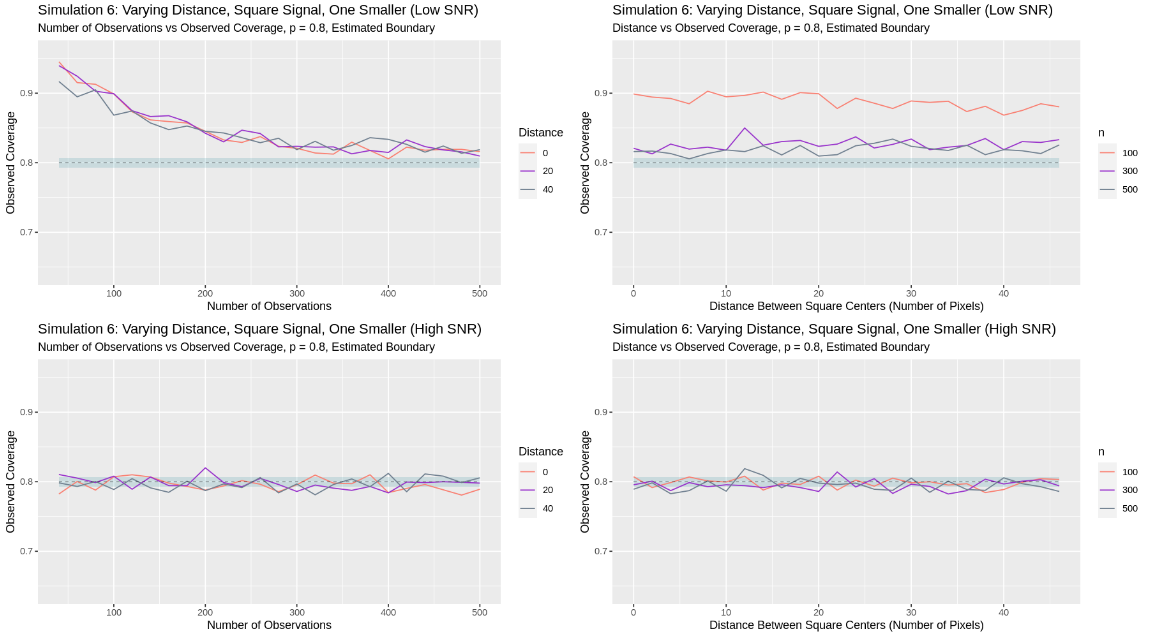

Figure S7: Empirical coverage for simulation 6. Top: Low-SNR synthetic data results. Bottom: High-SNR synthetic data results. Left: Number of observations vs observed coverage, shown for square separations of $0, 20$ and 40 pixels. Right: Separation between squares vs observed coverage, shown for $n = 100, 300$ and 500.

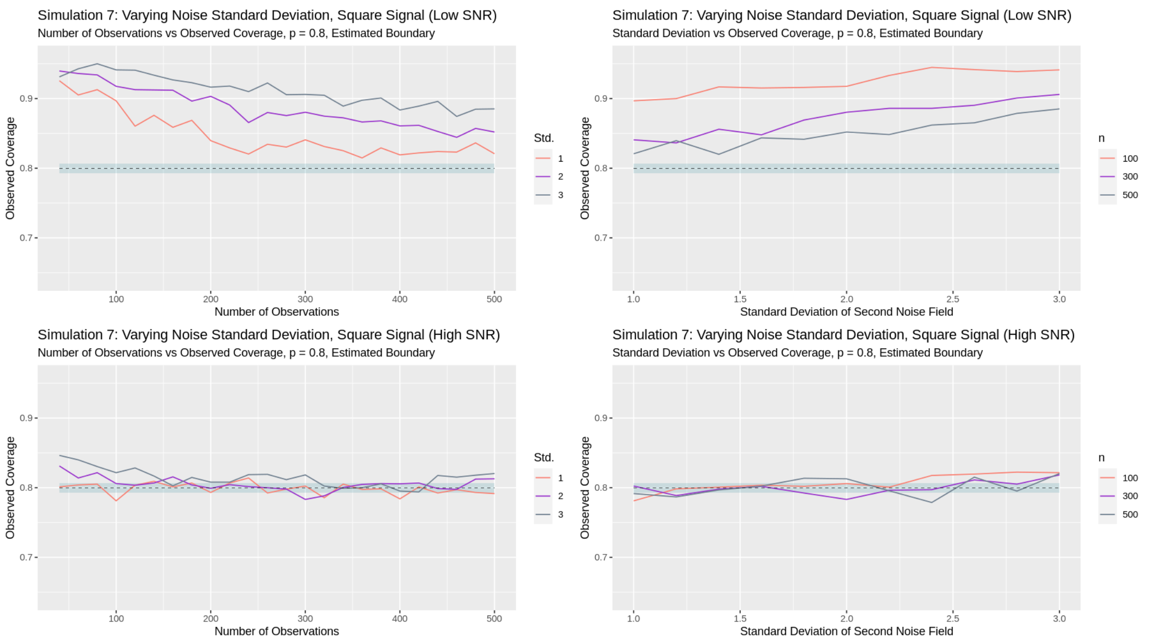

Figure S8: Empirical coverage for simulation 7. Top: Low-SNR synthetic data results. Bottom: High-SNR synthetic data results. Left: Number of observations vs observed coverage, shown for noise standard deviations of $1, 2$ and 3. Right: The standard deviation of the second noise field vs observed coverage, shown for $n = 100, 300$ and 500.

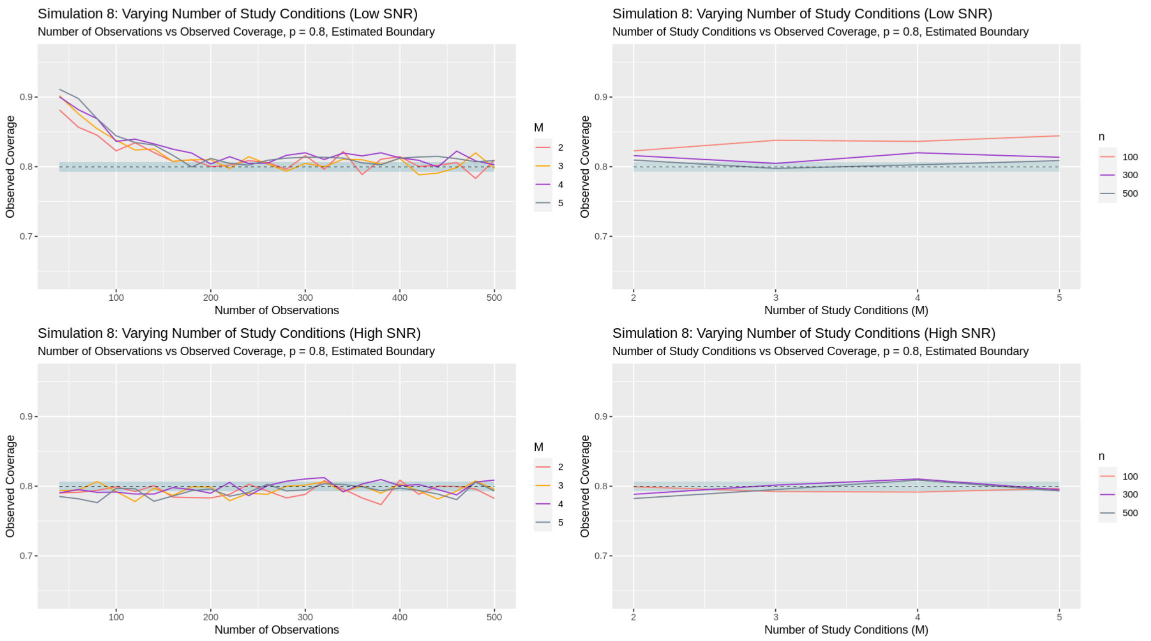

Figure S9: Empirical coverage for simulation 8. Top: Low-SNR synthetic data results. Bottom: High-SNR synthetic data results. Left: Number of observations vs observed coverage, shown for $M = 2, 3, 4$ and 5. Right: Number of study conditions (M) vs observed coverage, shown for $n = 100, 300$ and 500.

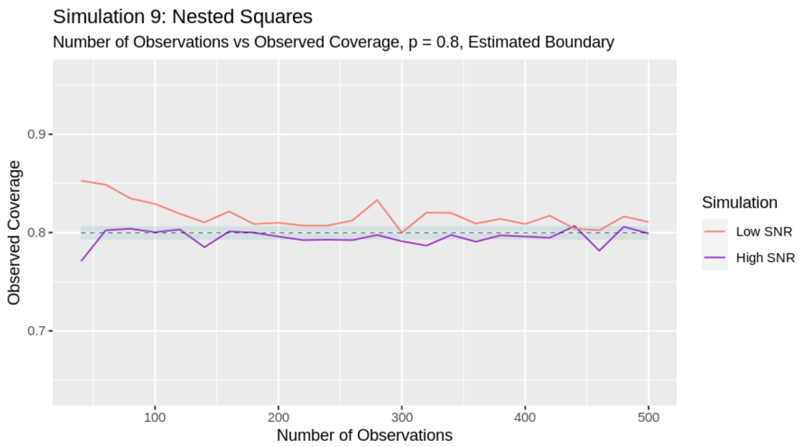

Figure S10: Empirical coverage for simulation 9. Results are shown for both low-SNR synthetic data and high-SNR synthetic data. Displayed is the number of observations vs observed coverage.

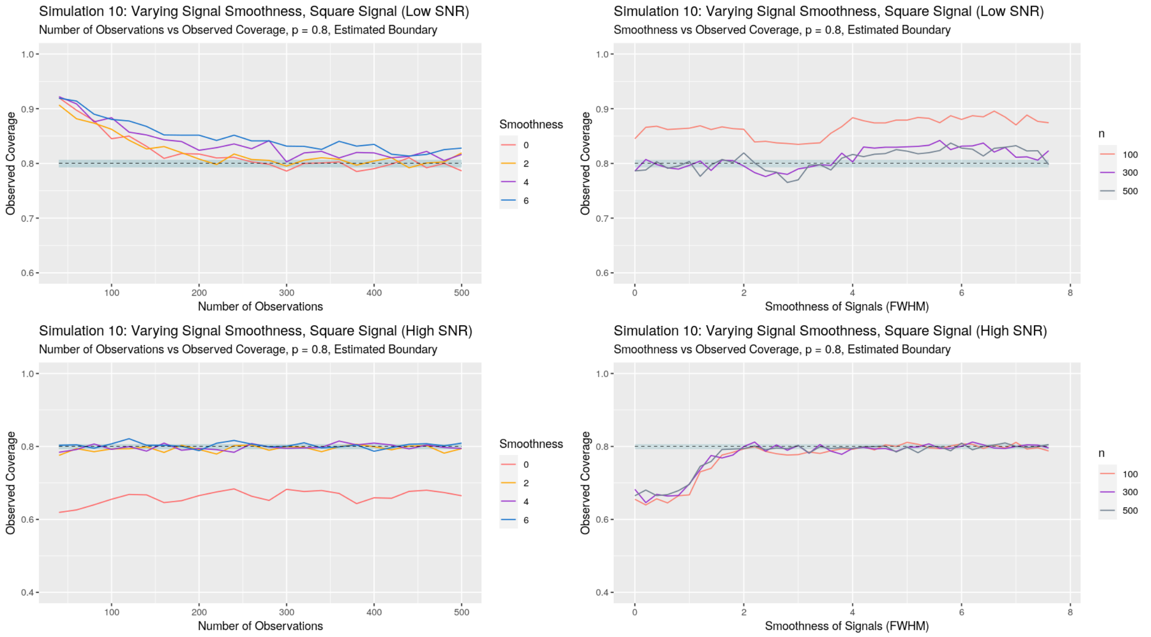

Figure S11: Empirical coverage for simulation 10. Top: Low-SNR synthetic data results. Bottom: High-SNR synthetic data results. Left: Number of observations vs observed coverage, shown for signal smoothness of $0, 2, 4$ and 6 FWHM. Right: Signal smoothness vs observed coverage, shown for $n = 100, 300$ and 500.

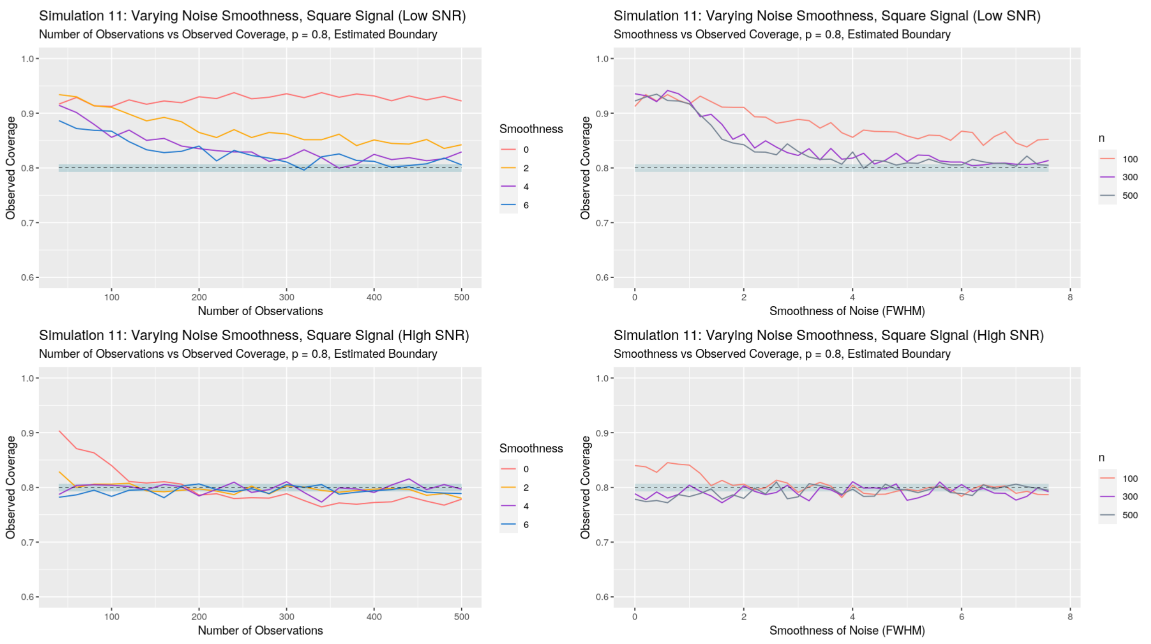

Figure S12: Empirical coverage for simulation 11. Top: Low-SNR synthetic data results. Bottom: High-SNR synthetic data results. Left: Number of observations vs observed coverage, shown for noise smoothness of $0, 2, 4$ and 6 FWHM. Right: Noise smoothness vs observed coverage, shown for $n = 100, 300$ and 500.

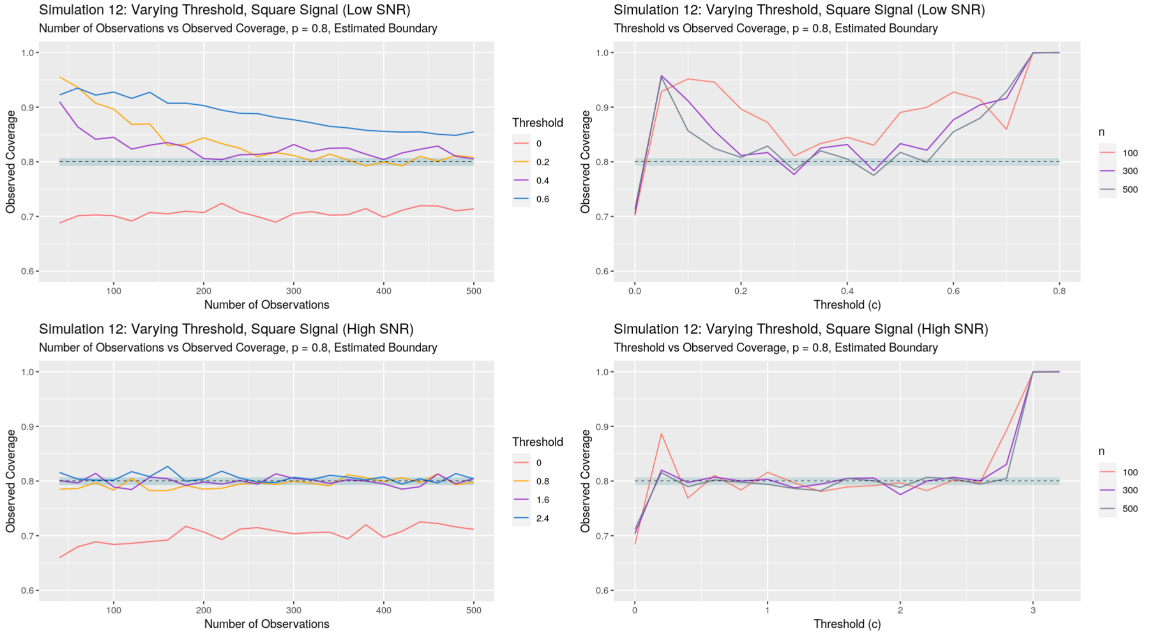

Figure S13: Empirical coverage for simulation 12. Top: Low-SNR synthetic data results. Bottom: High-SNR synthetic data results. Left: Number of observations vs observed coverage, shown for thresholds $c = 0, 0.2, 0.4$ and 0.6 (top) and $c = 0, 0.8, 1.6$ and 2.4 (bottom). Right: Threshold vs observed coverage, shown for $n = 100, 300$ and 500.

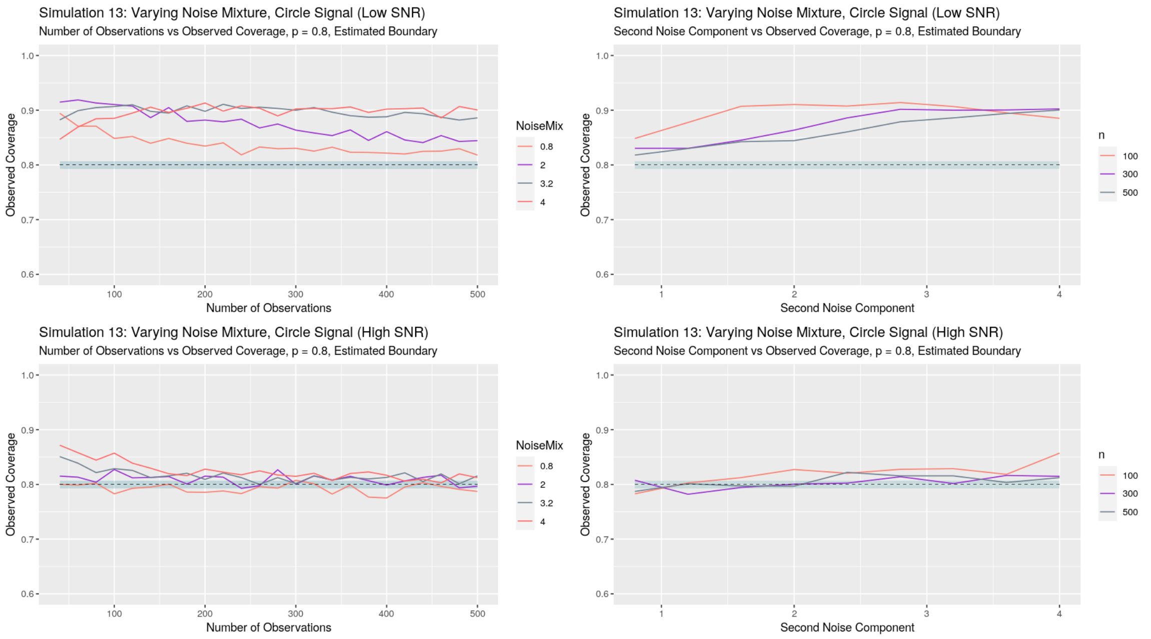

Figure S14: Empirical coverage for simulation 13. Top: Low-SNR synthetic data results. Bottom: High-SNR synthetic data results. Left: Number of observations vs observed coverage, shown for even Gaussian noise mixtures of $N(0,1)$ and $N(0,\sigma^2)$ with $\sigma = 0.8, 2, 3.2$ and 4. Right: Second noise component (σ) vs observed coverage, shown for $n = 100, 300$ and 500.

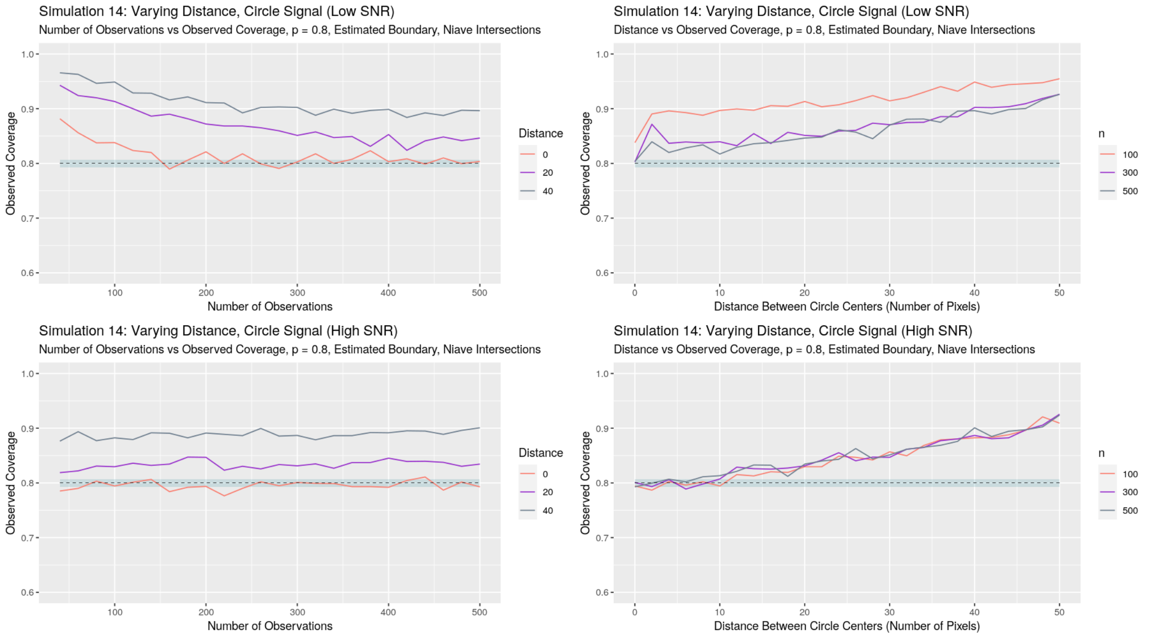

Figure S15: Empirical coverage for simulation 14. Top: Low-SNR synthetic data results. Bottom: High-SNR synthetic data results. Left: Number of observations vs observed coverage, shown for circle separations of 0, 20 and 40 pixels. Right: Separation between circles vs observed coverage, shown for $n = 100, 300$ and 500.

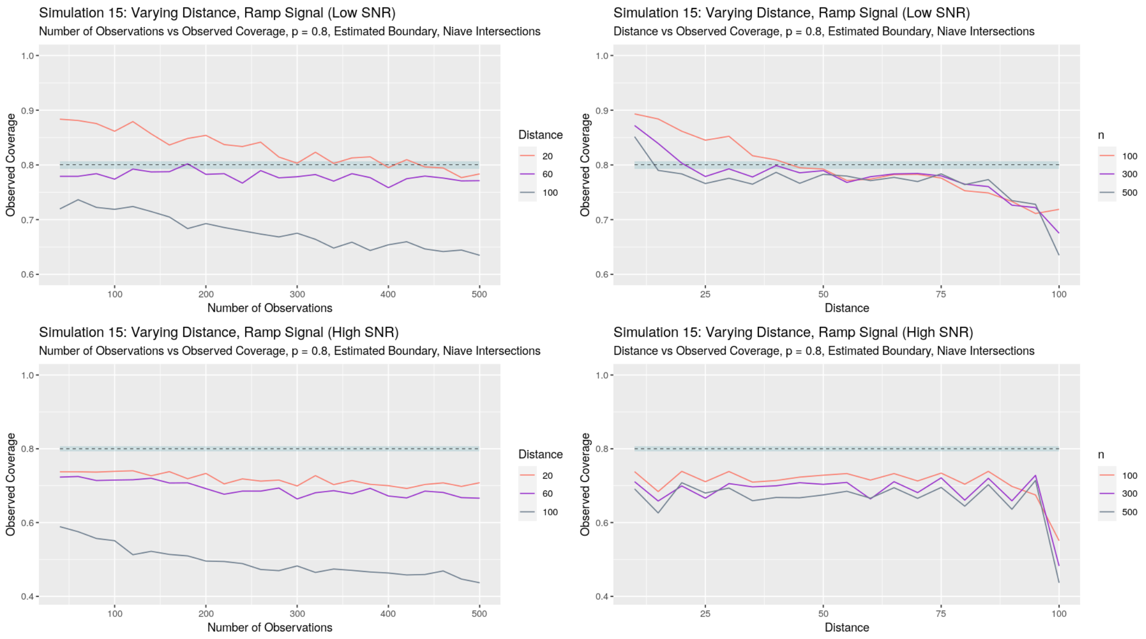

Figure S16: Empirical coverage for simulation 15. Top: Low-SNR synthetic data results. Bottom: High-SNR synthetic data results. Left: Number of observations vs observed coverage, shown for ramp separations of $20, 60$ and 100 pixels. Right: Separation between ramps vs observed coverage, shown for $n = 100, 300$ and 500.

S5.2 Nominal Coverage: 90%

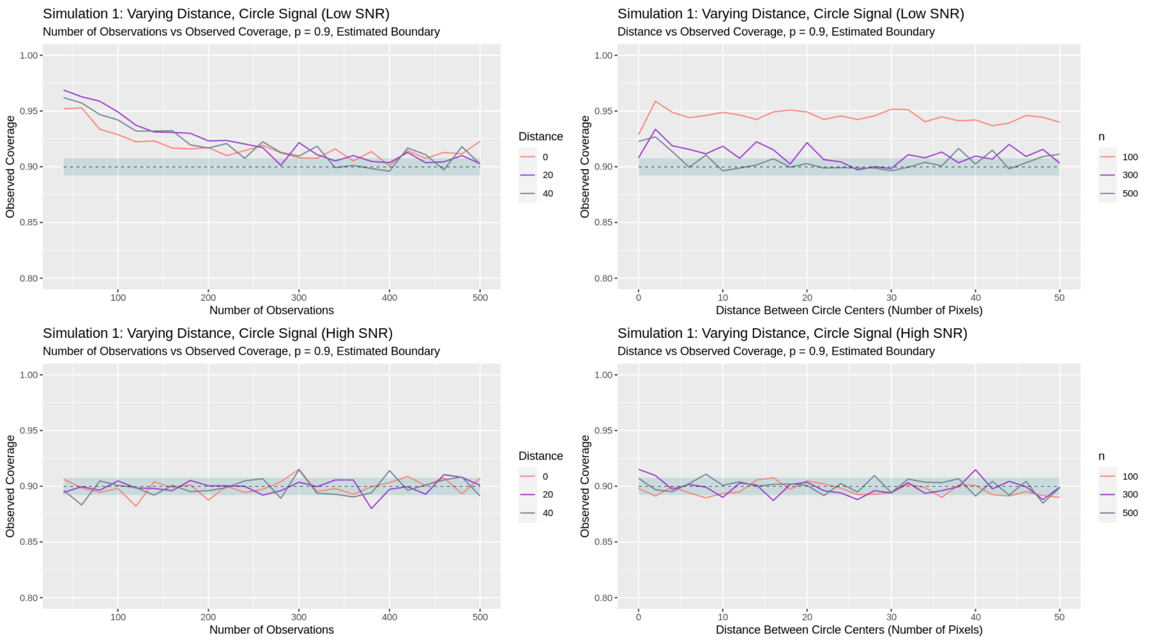

Figure S17: Empirical coverage for simulation 1. Top: Low-SNR synthetic data results. Bottom: High-SNR synthetic data results. Left: Number of observations vs observed coverage, shown for circle separations of $0, 20$ and 40 pixels. Right: Separation between circles vs observed coverage, shown for $n = 100, 300$ and 500.

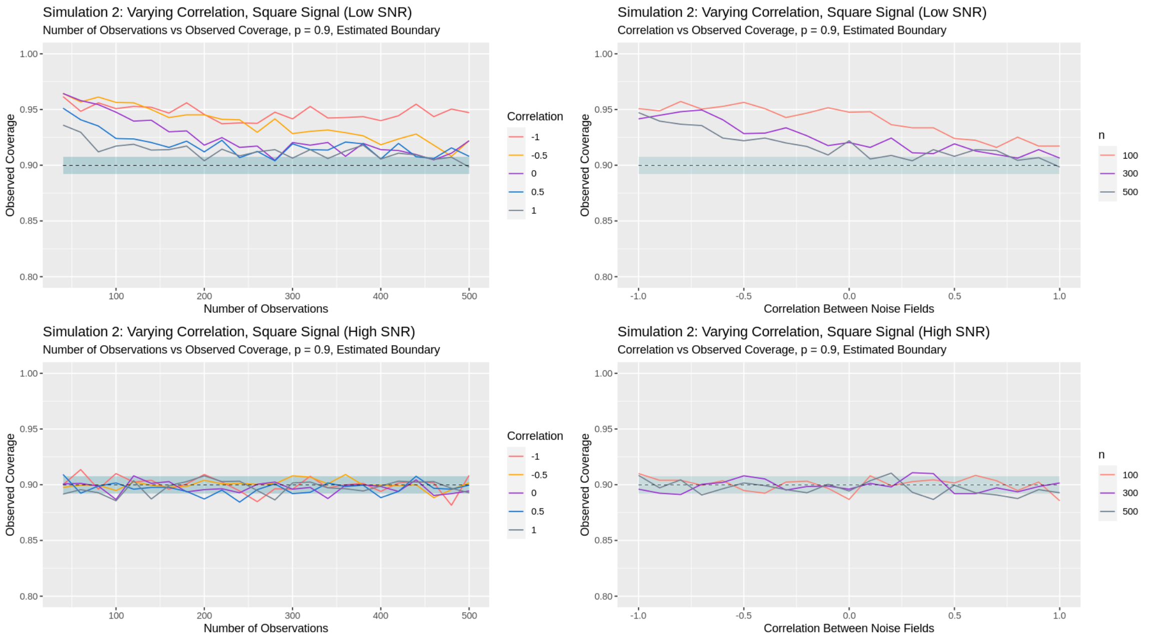

Figure S18: Empirical coverage for simulation 2. Top: Low-SNR synthetic data results. Bottom: High-SNR synthetic data results. Left: Number of observations vs observed coverage, shown for correlations of $-1, -0.5, 0, 0.5$ and 1. Right: Correlation between noise fields vs observed coverage, shown for $n = 100, 300$ and 500.

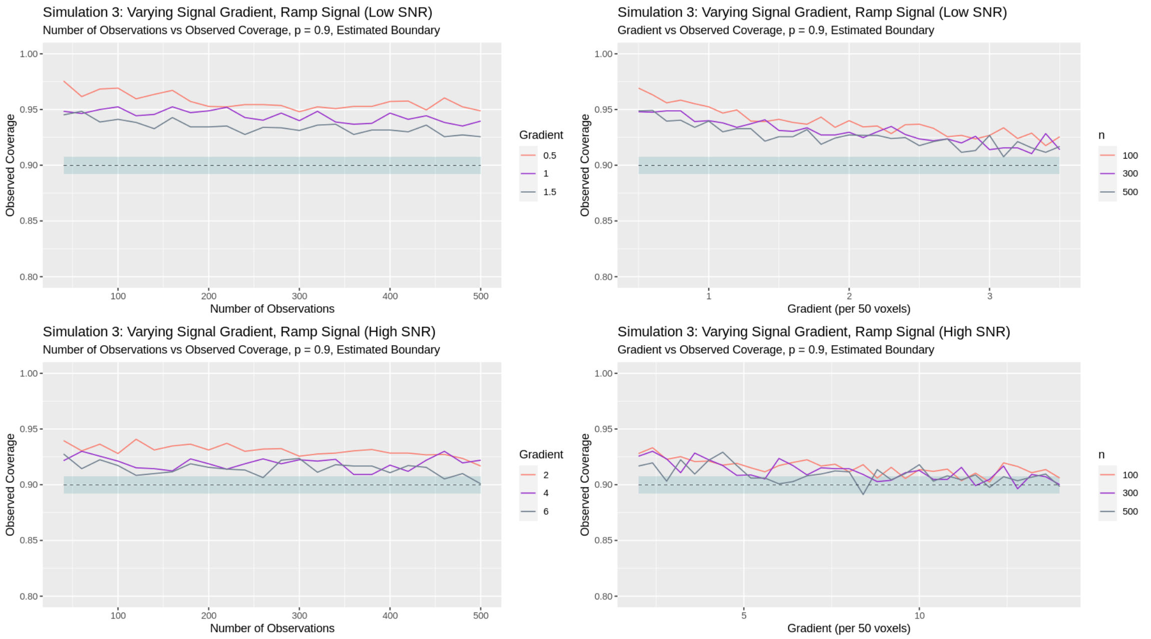

Figure S19: Empirical coverage for simulation 3. Top: Low-SNR synthetic data results. Bottom: High-SNR synthetic data results. Left: Number of observations vs observed coverage, shown for gradients of $0.5, 1$ and 1.5 per 50 pixels (top), and $2, 4$ and 6 per 50 pixels (bottom). Right: Ramp gradient per 50 pixels vs observed coverage, shown for $n = 100, 300$ and 500.

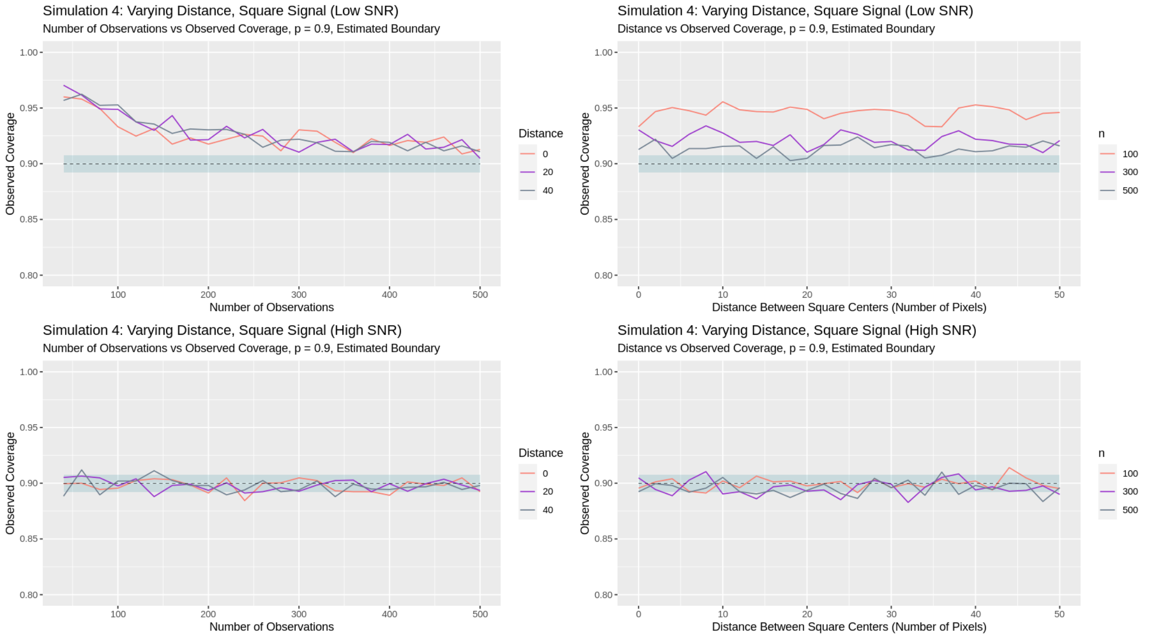

Figure S20: Empirical coverage for simulation 4. Top: Low-SNR synthetic data results. Bottom: High-SNR synthetic data results. Left: Number of observations vs observed coverage, shown for square separations of $0, 20$ and 40 pixels. Right: Separation between squares vs observed coverage, shown for $n = 100, 300$ and 500.

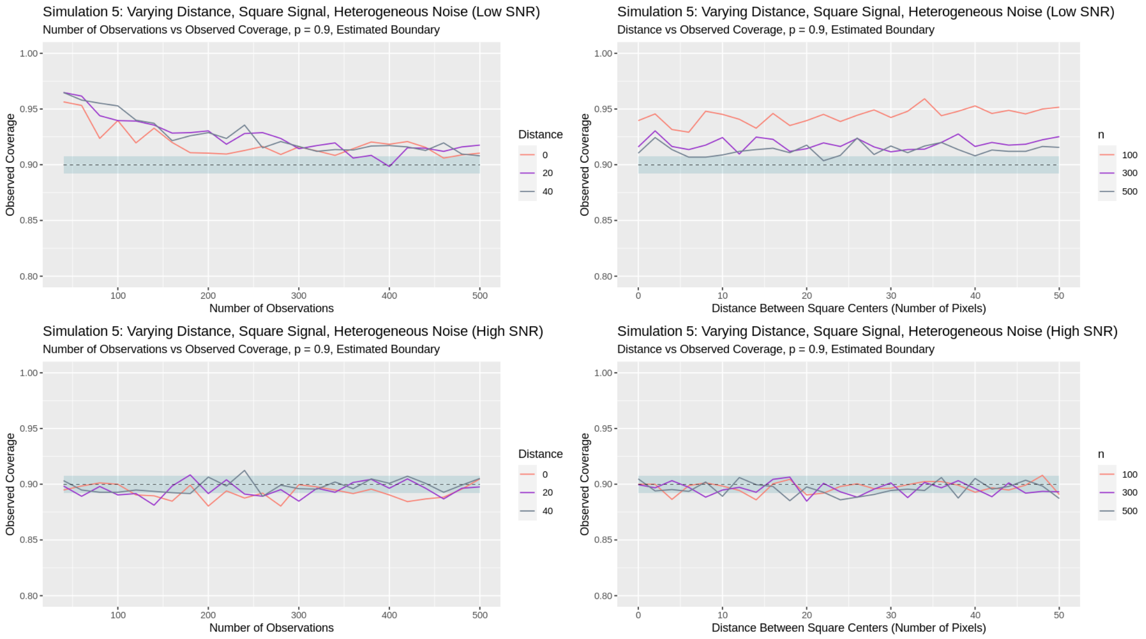

Figure S21: Empirical coverage for simulation 5. Top: Low-SNR synthetic data results. Bottom: High-SNR synthetic data results. Left: Number of observations vs observed coverage, shown for square separations of $0, 20$ and 40 pixels. Right: Separation between squares vs observed coverage, shown for $n = 100, 300$ and 500.

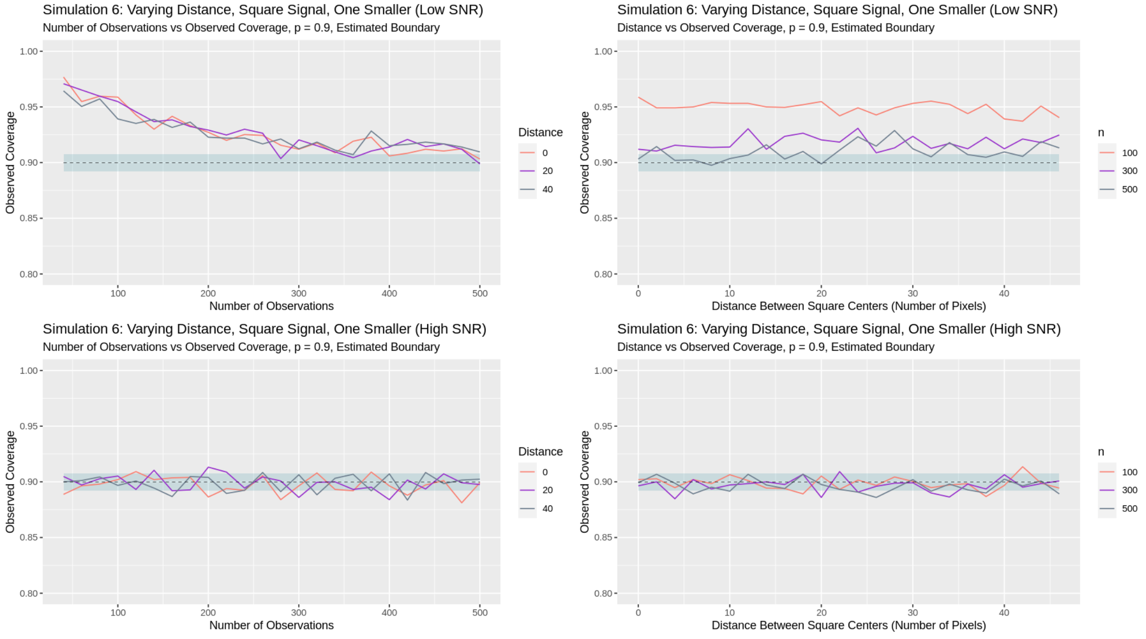

Figure S22: Empirical coverage for simulation 6. Top: Low-SNR synthetic data results. Bottom: High-SNR synthetic data results. Left: Number of observations vs observed coverage, shown for square separations of $0, 20$ and 40 pixels. Right: Separation between squares vs observed coverage, shown for $n = 100, 300$ and 500.

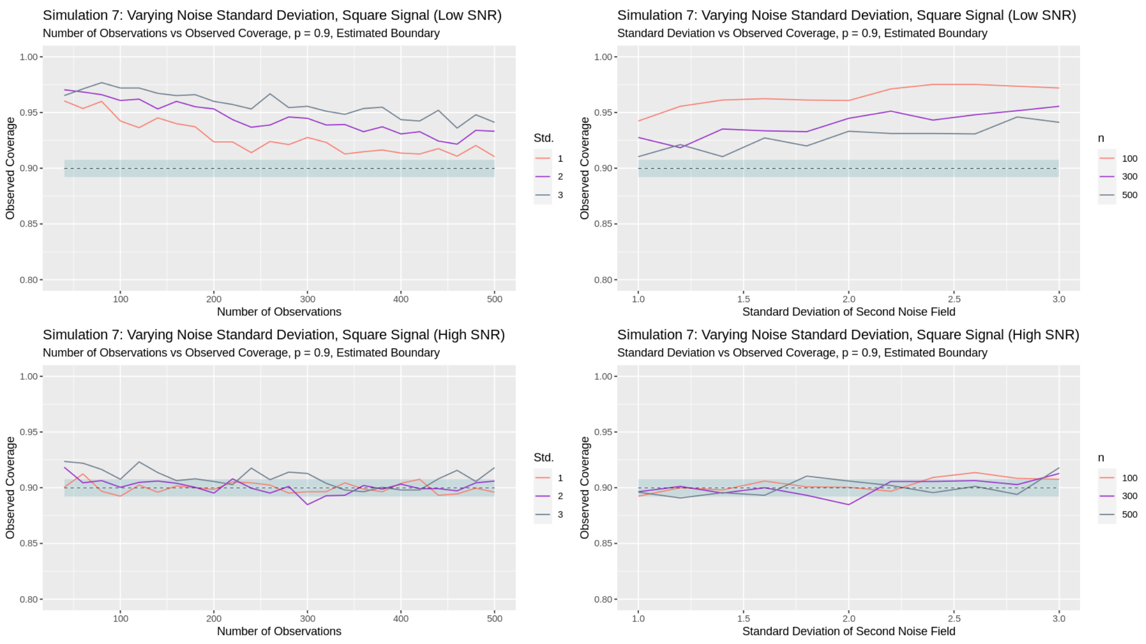

Figure S23: Empirical coverage for simulation 7. Top: Low-SNR synthetic data results. Bottom: High-SNR synthetic data results. Left: Number of observations vs observed coverage, shown for noise standard deviations of $1, 2$ and 3. Right: The standard deviation of the second noise field vs observed coverage, shown for $n = 100, 300$ and 500.

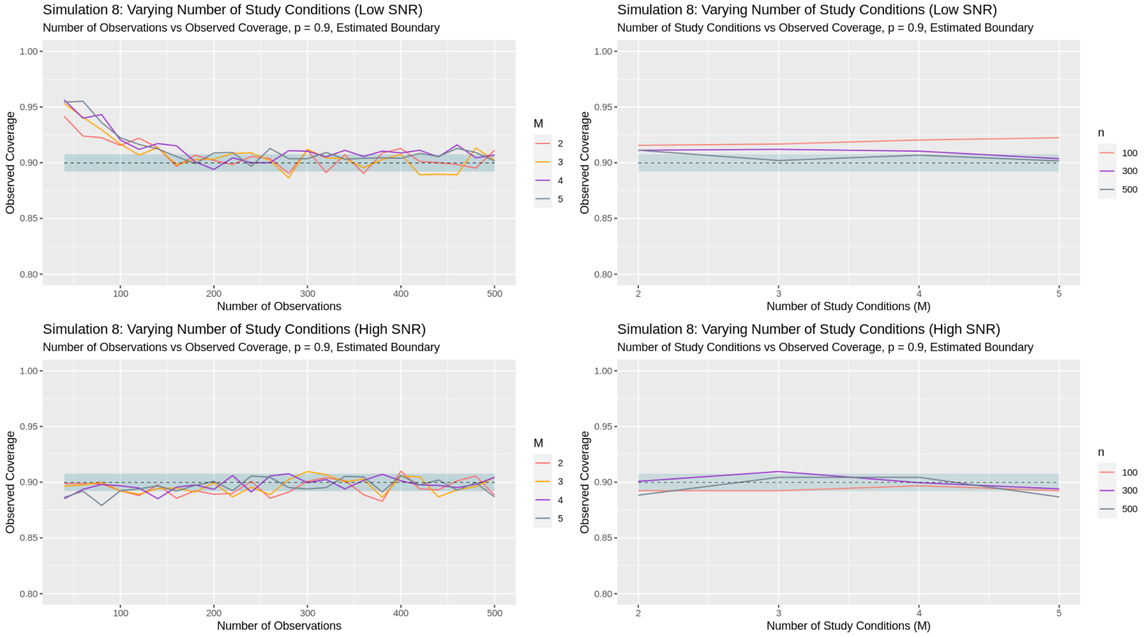

Figure S24: Empirical coverage for simulation 8. Top: Low-SNR synthetic data results. Bottom: High-SNR synthetic data results. Left: Number of observations vs observed coverage, shown for $M = 2, 3, 4$ and 5. Right: Number of study conditions (M) vs observed coverage, shown for $n = 100, 300$ and 500.

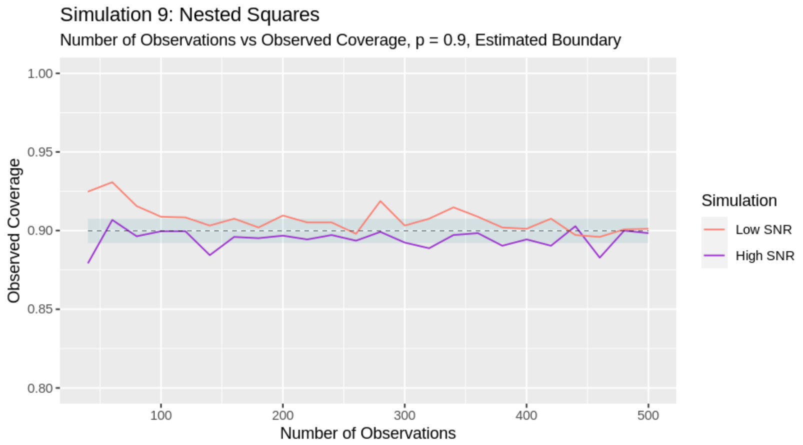

Figure S25: Empirical coverage for simulation 9. Results are shown for both low-SNR synthetic data and high-SNR synthetic data. Displayed is the number of observations vs observed coverage.

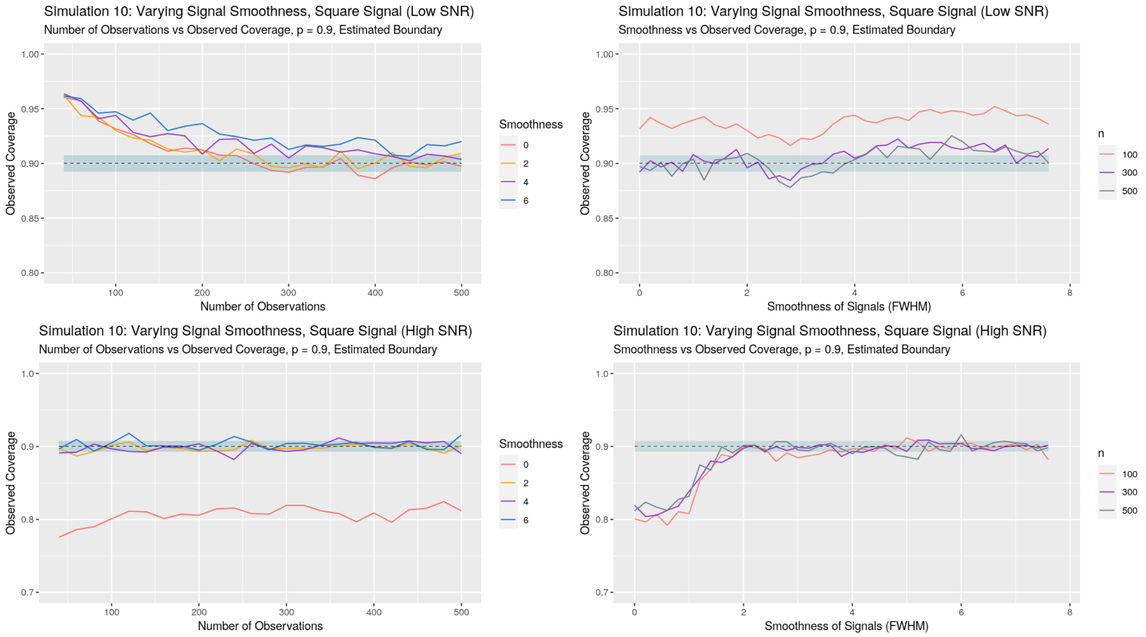

Figure S26: Empirical coverage for simulation 10. Top: Low-SNR synthetic data results. Bottom: High-SNR synthetic data results. Left: Number of observations vs observed coverage, shown for signal smoothness of $0, 2, 4$ and 6 FWHM. Right: Signal smoothness vs observed coverage, shown for $n = 100, 300$ and 500.

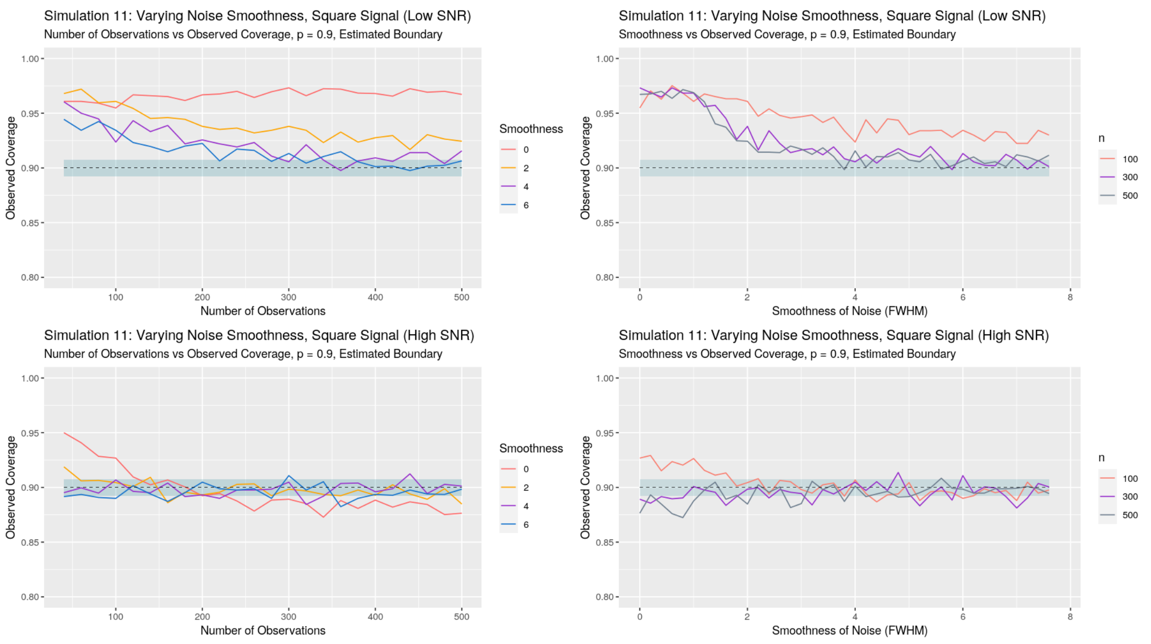

Figure S27: Empirical coverage for simulation 11. Top: Low-SNR synthetic data results. Bottom: High-SNR synthetic data results. Left: Number of observations vs observed coverage, shown for noise smoothness of $0, 2, 4$ and 6 FWHM. Right: Noise smoothness vs observed coverage, shown for $n = 100, 300$ and 500.

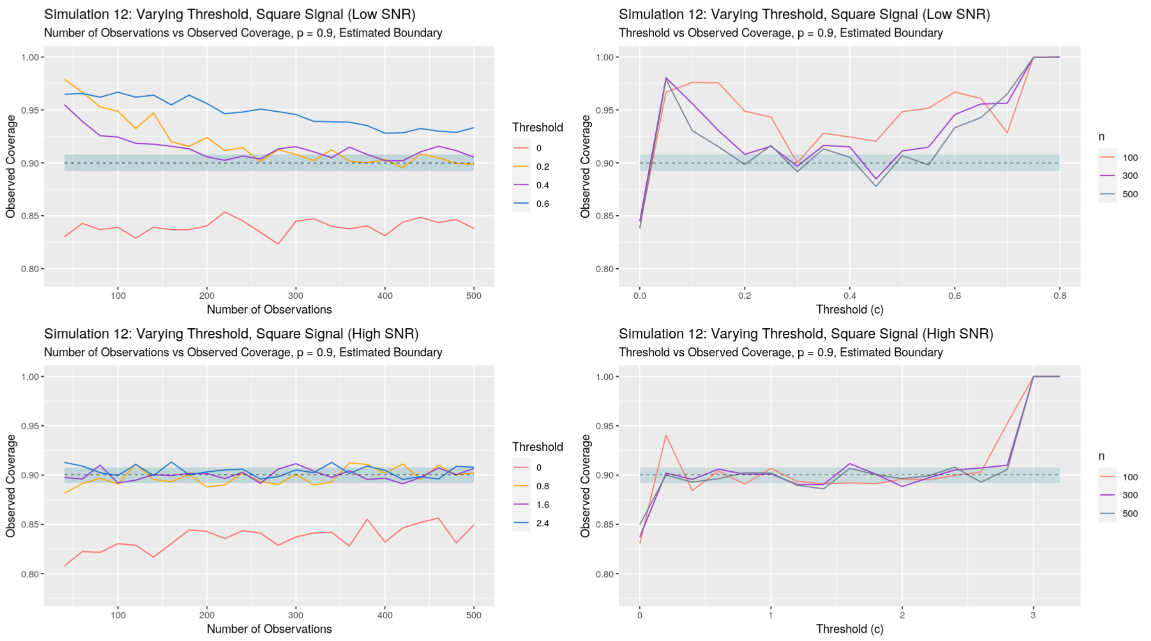

Figure S28: Empirical coverage for simulation 12. Top: Low-SNR synthetic data results. Bottom: High-SNR synthetic data results. Left: Number of observations vs observed coverage, shown for thresholds $c = 0, 0.2, 0.4$ and 0.6 (top) and $c = 0, 0.8, 1.6$ and 2.4 (bottom). Right: Threshold vs observed coverage, shown for $n = 100, 300$ and 500.

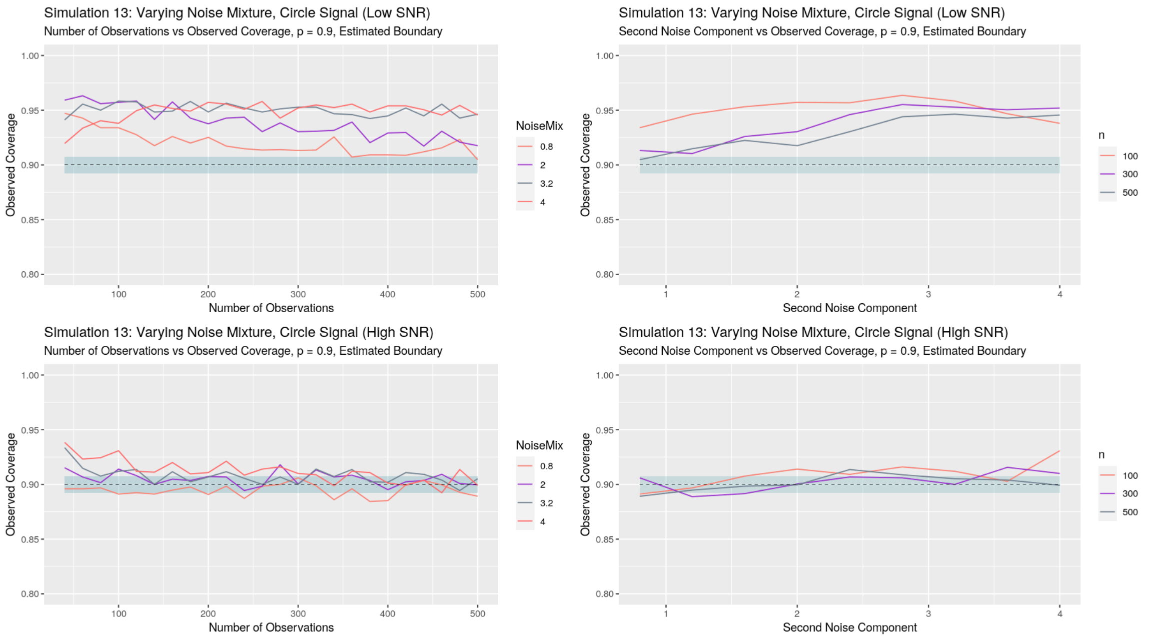

Figure S29: Empirical coverage for simulation 13. Top: Low-SNR synthetic data results. Bottom: High-SNR synthetic data results. Left: Number of observations vs observed coverage, shown for even Gaussian noise mixtures of $N(0,1)$ and $N(0,\sigma^2)$ with $\sigma = 0.8, 2, 3.2$ and 4. Right: Second noise component (σ) vs observed coverage, shown for $n = 100, 300$ and 500.

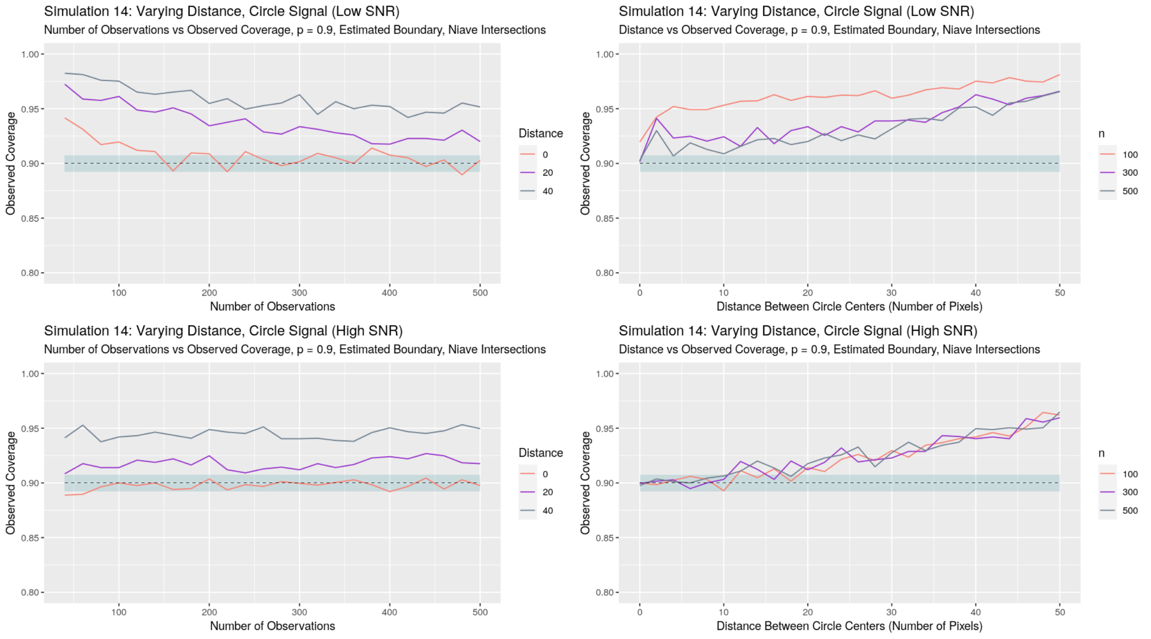

Figure S30: Empirical coverage for simulation 14. Top: Low-SNR synthetic data results. Bottom: High-SNR synthetic data results. Left: Number of observations vs observed coverage, shown for circle separations of 0, 20 and 40 pixels. Right: Separation between circles vs observed coverage, shown for $n = 100, 300$ and 500.

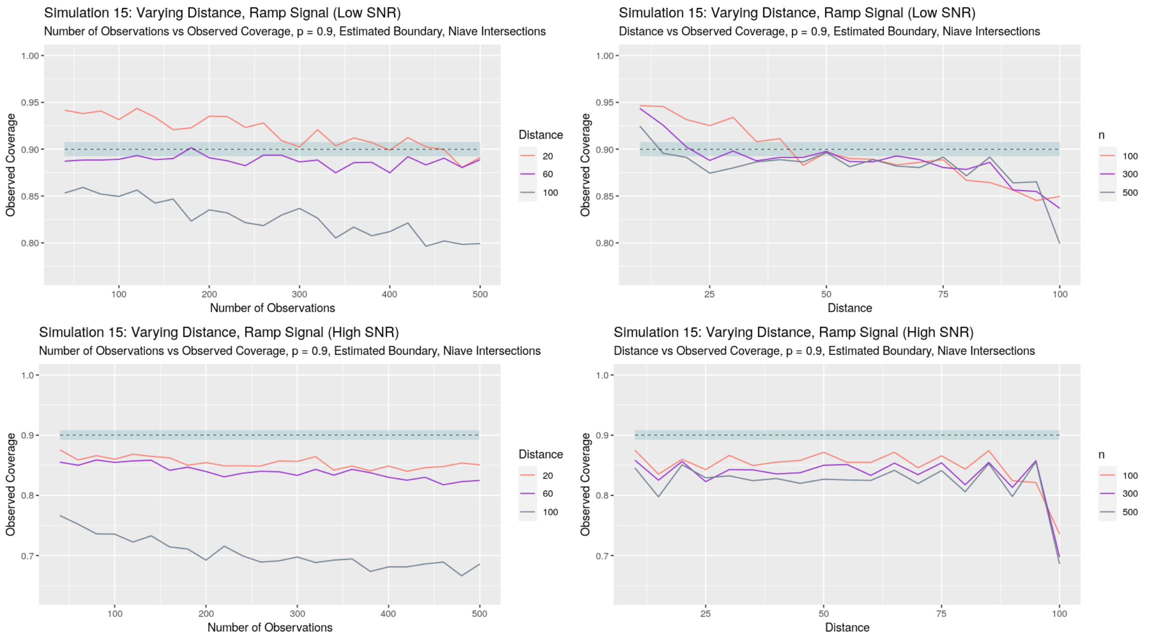

Figure S31: Empirical coverage for simulation 15. Top: Low-SNR synthetic data results. Bottom: High-SNR synthetic data results. Left: Number of observations vs observed coverage, shown for ramp separations of $20, 60$ and 100 pixels. Right: Separation between ramps vs observed coverage, shown for $n = 100, 300$ and 500.

S5.3 Nominal Coverage: 95%

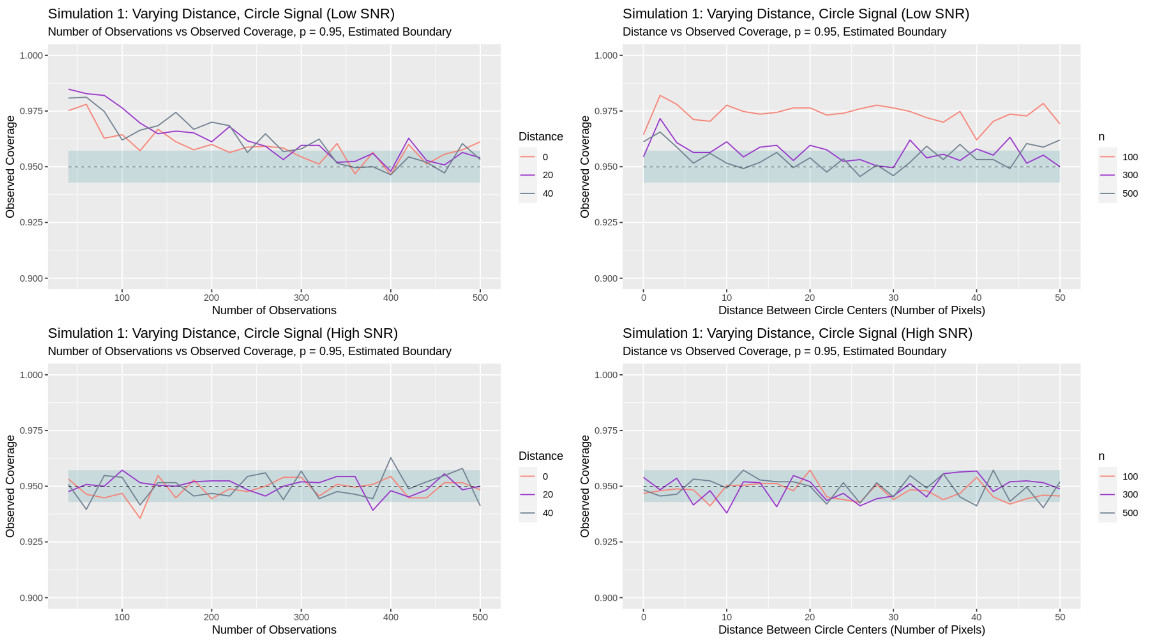

Figure S32: Empirical coverage for simulation 1. Top: Low-SNR synthetic data results. Bottom: High-SNR synthetic data results. Left: Number of observations vs observed coverage, shown for circle separations of $0, 20$ and 40 pixels. Right: Separation between circles vs observed coverage, shown for $n = 100, 300$ and 500.

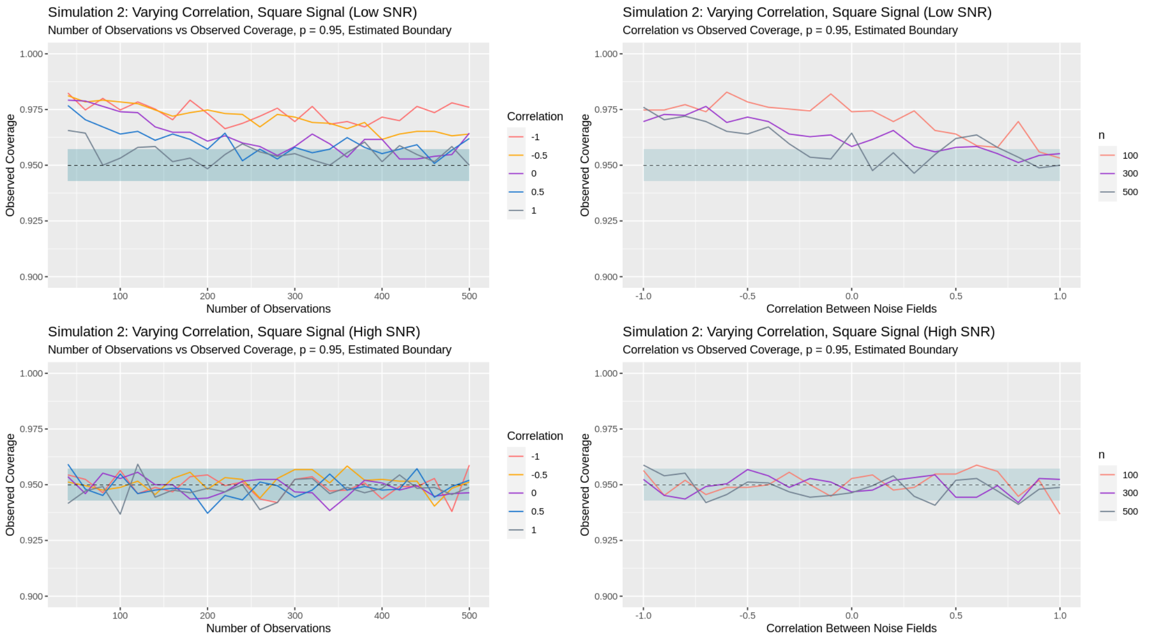

Figure S33: Empirical coverage for simulation 2. Top: Low-SNR synthetic data results. Bottom: High-SNR synthetic data results. Left: Number of observations vs observed coverage, shown for correlations of $-1, -0.5, 0, 0.5$ and 1. Right: Correlation between noise fields vs observed coverage, shown for $n = 100, 300$ and 500.

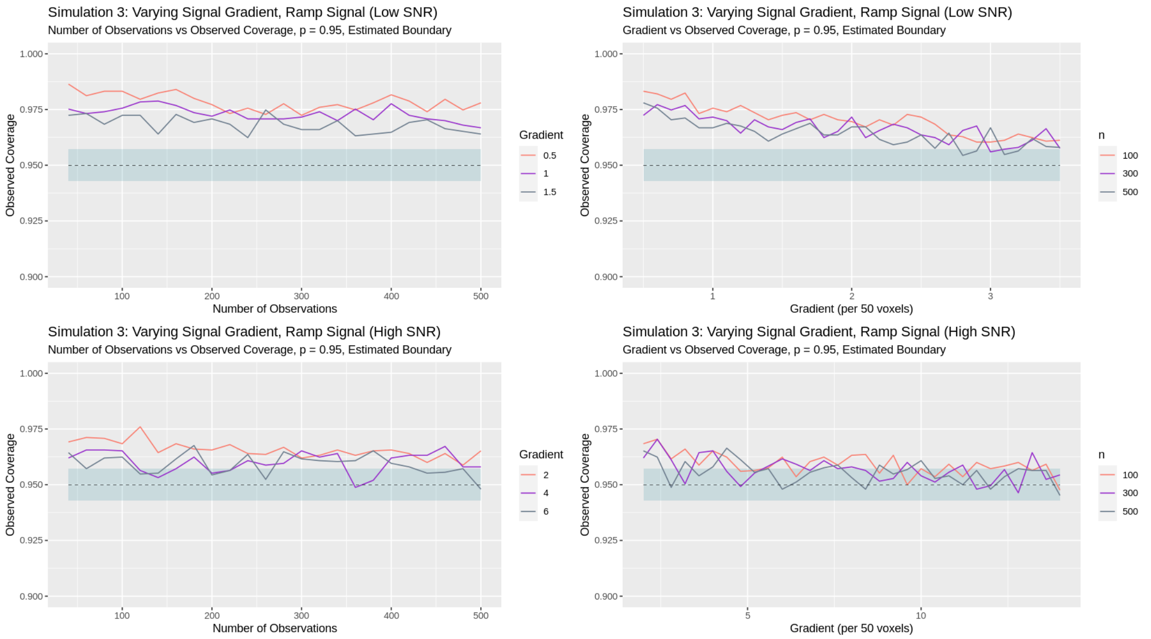

Figure S34: Empirical coverage for simulation 3. Top: Low-SNR synthetic data results. Bottom: High-SNR synthetic data results. Left: Number of observations vs observed coverage, shown for gradients of $0.5, 1$ and 1.5 per 50 pixels (top), and $2, 4$ and 6 per 50 pixels (bottom). Right: Ramp gradient per 50 pixels vs observed coverage, shown for $n = 100, 300$ and 500.

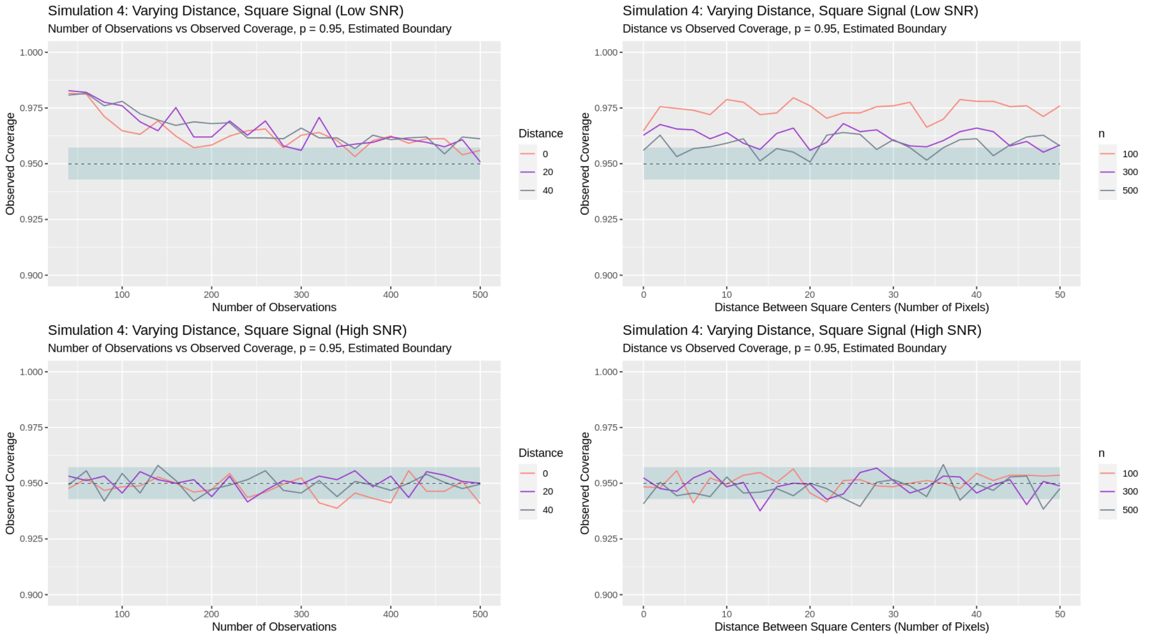

Figure S35: Empirical coverage for simulation 4. Top: Low-SNR synthetic data results. Bottom: High-SNR synthetic data results. Left: Number of observations vs observed coverage, shown for square separations of $0, 20$ and 40 pixels. Right: Separation between squares vs observed coverage, shown for $n = 100, 300$ and 500.

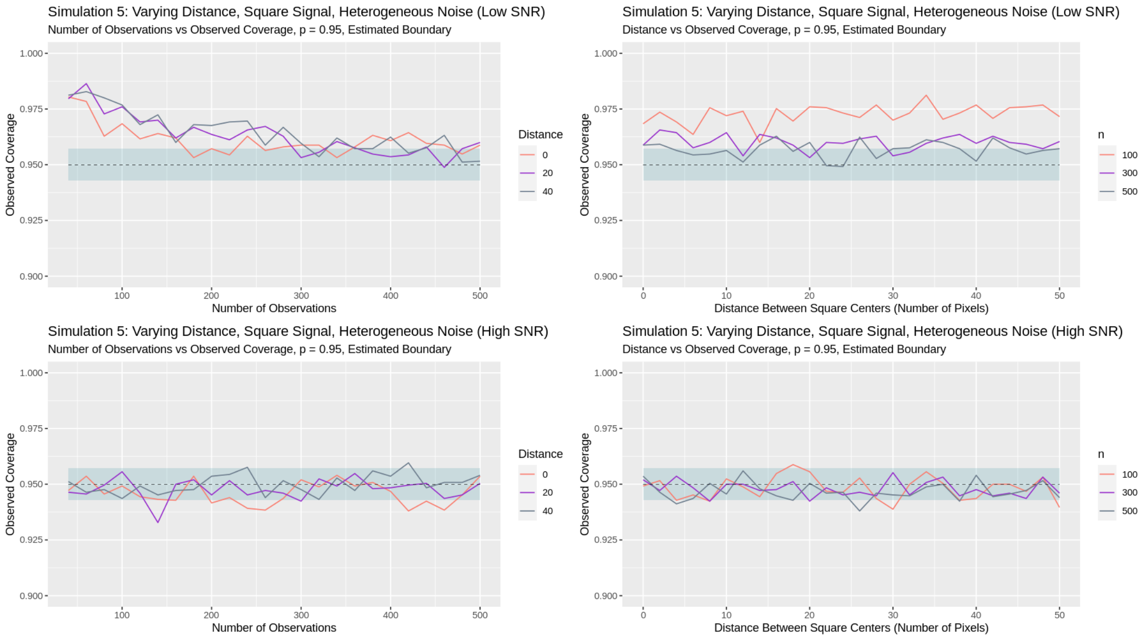

Figure S36: Empirical coverage for simulation 5. Top: Low-SNR synthetic data results. Bottom: High-SNR synthetic data results. Left: Number of observations vs observed coverage, shown for square separations of $0, 20$ and 40 pixels. Right: Separation between squares vs observed coverage, shown for $n = 100, 300$ and 500.

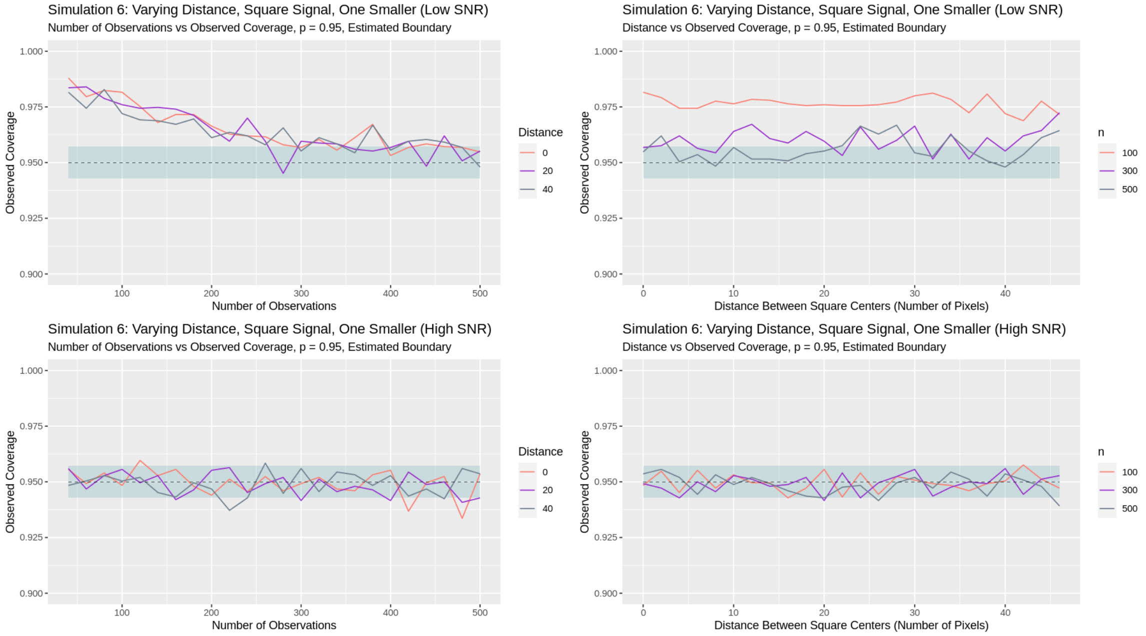

Figure S37: Empirical coverage for simulation 6. Top: Low-SNR synthetic data results. Bottom: High-SNR synthetic data results. Left: Number of observations vs observed coverage, shown for square separations of $0, 20$ and 40 pixels. Right: Separation between squares vs observed coverage, shown for $n = 100, 300$ and 500.

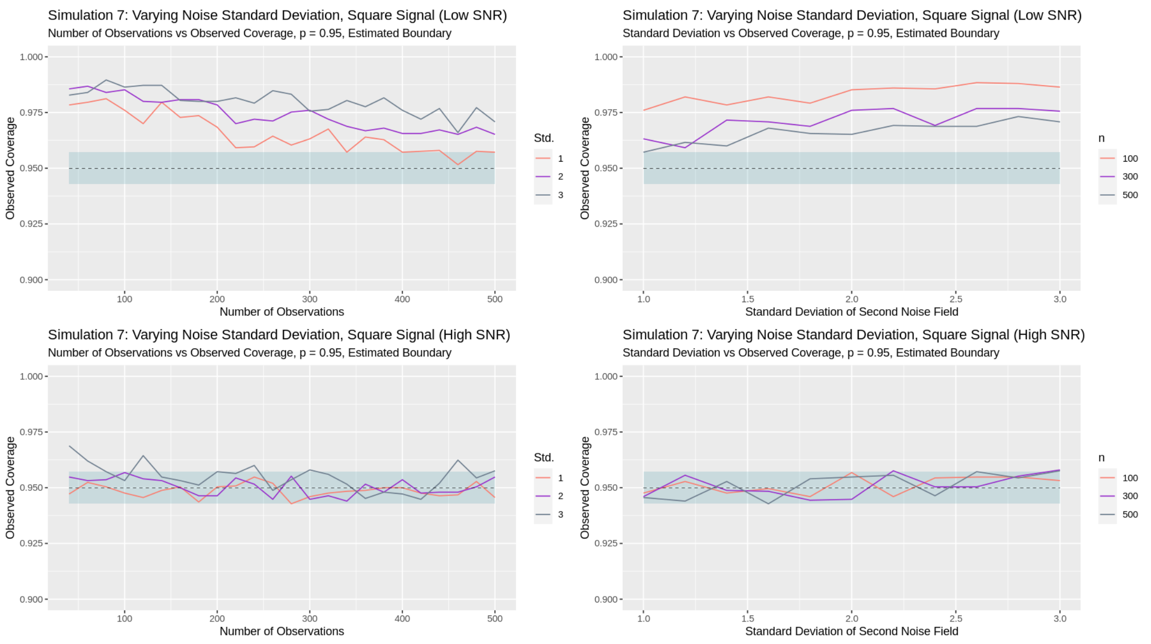

Figure S38: Empirical coverage for simulation 7. Top: Low-SNR synthetic data results. Bottom: High-SNR synthetic data results. Left: Number of observations vs observed coverage, shown for noise standard deviations of $1, 2$ and 3. Right: The standard deviation of the second noise field vs observed coverage, shown for $n = 100, 300$ and 500.

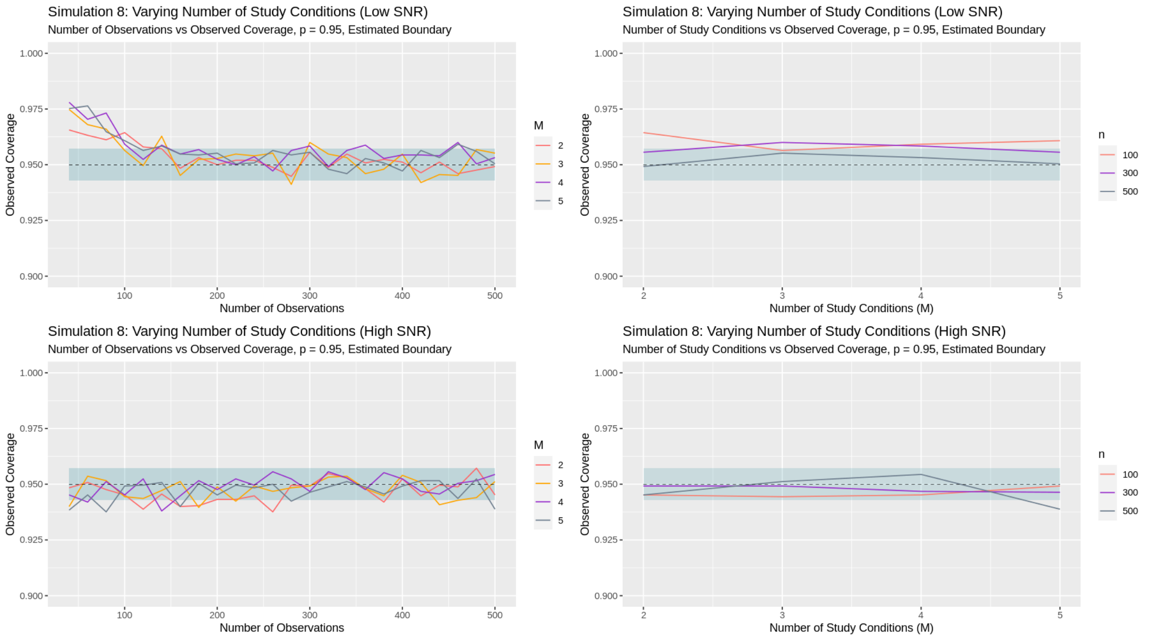

Figure S39: Empirical coverage for simulation 8. Top: Low-SNR synthetic data results. Bottom: High-SNR synthetic data results. Left: Number of observations vs observed coverage, shown for $M = 2, 3, 4$ and 5. Right: Number of study conditions (M) vs observed coverage, shown for $n = 100, 300$ and 500.

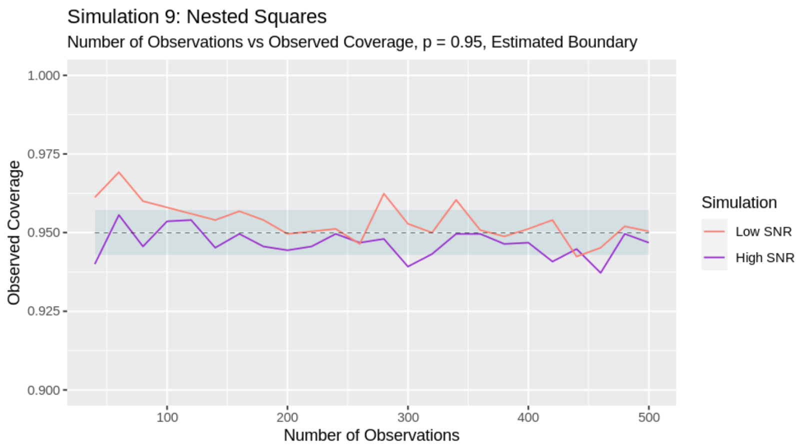

Figure S40: Empirical coverage for simulation 9. Results are shown for both low-SNR synthetic data and high-SNR synthetic data. Displayed is the number of observations vs observed coverage.

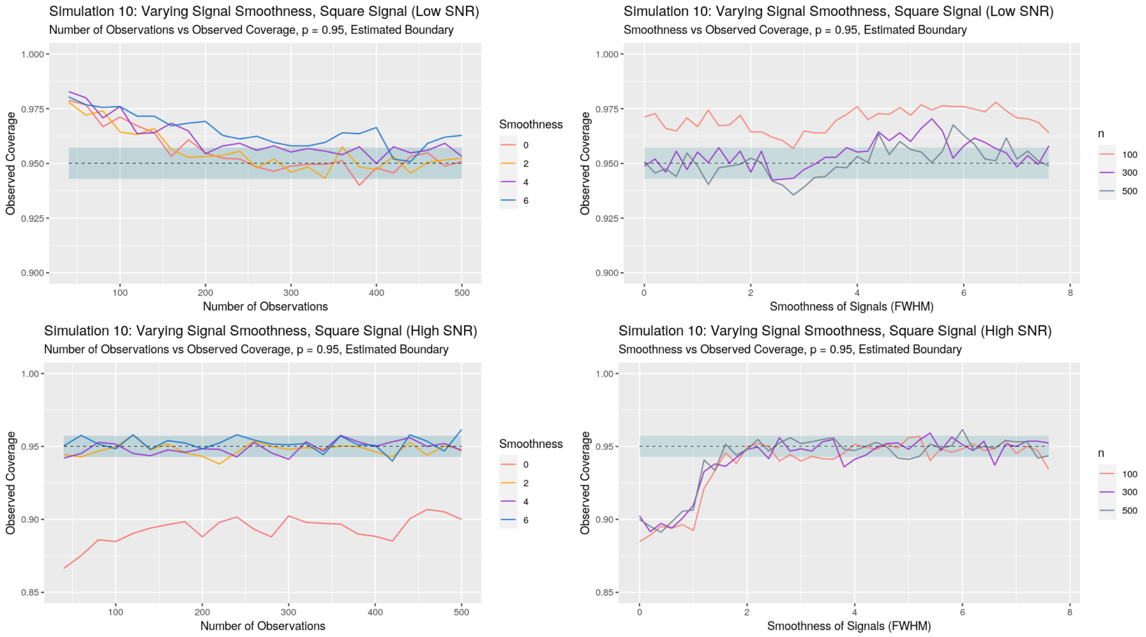

Figure S41: Empirical coverage for simulation 10. Top: Low-SNR synthetic data results. Bottom: High-SNR synthetic data results. Left: Number of observations vs observed coverage, shown for signal smoothness of 0, 2, 4 and 6 FWHM. Right: Signal smoothness vs observed coverage, shown for $n = 100, 300$ and 500.

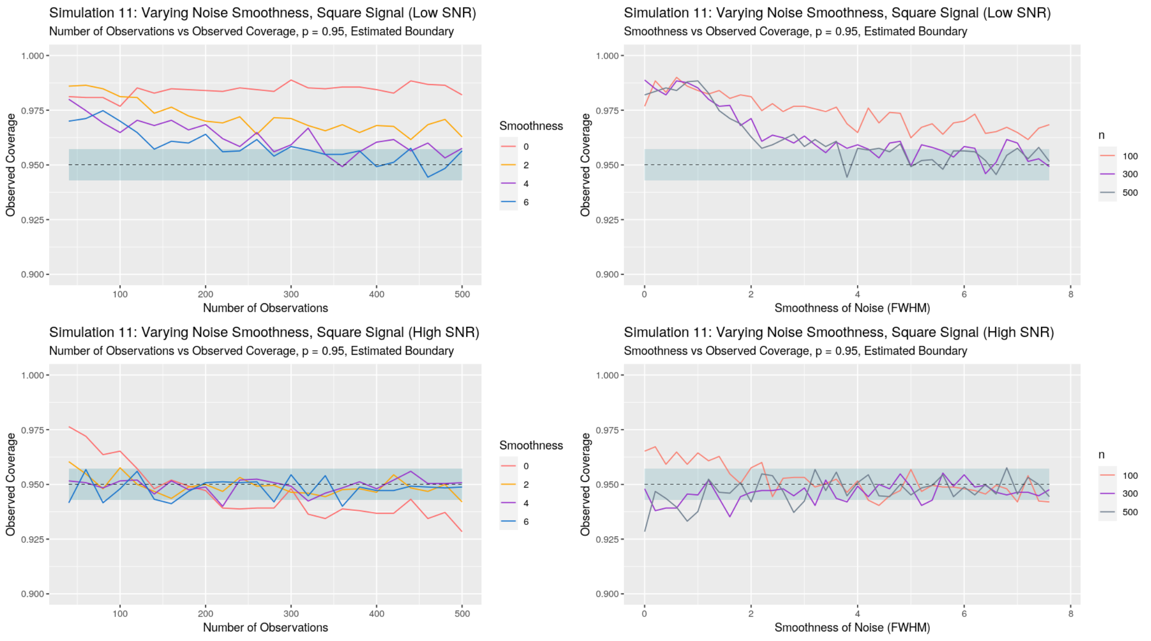

Figure S42: Empirical coverage for simulation 11. Top: Low-SNR synthetic data results. Bottom: High-SNR synthetic data results. Left: Number of observations vs observed coverage, shown for noise smoothness of $0, 2, 4$ and 6 FWHM. Right: Noise smoothness vs observed coverage, shown for $n = 100, 300$ and 500.

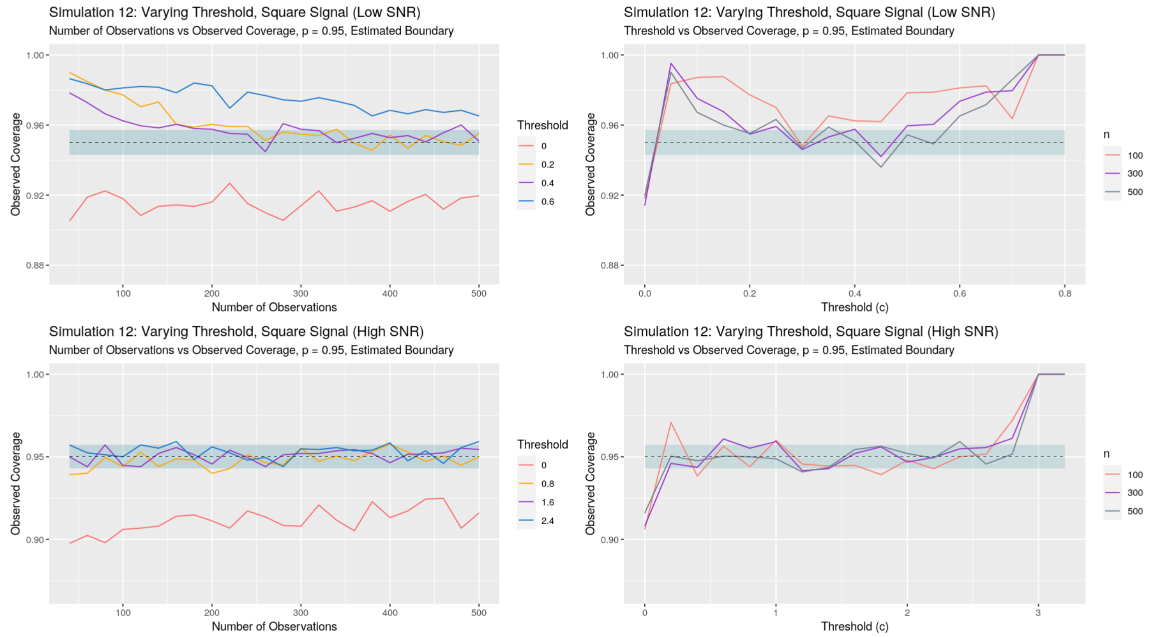

Figure S43: Empirical coverage for simulation 12. Top: Low-SNR synthetic data results. Bottom: High-SNR synthetic data results. Left: Number of observations vs observed coverage, shown for thresholds $c = 0, 0.2, 0.4$ and 0.6 (top) and $c = 0, 0.8, 1.6$ and 2.4 (bottom). Right: Threshold vs observed coverage, shown for $n = 100, 300$ and 500.

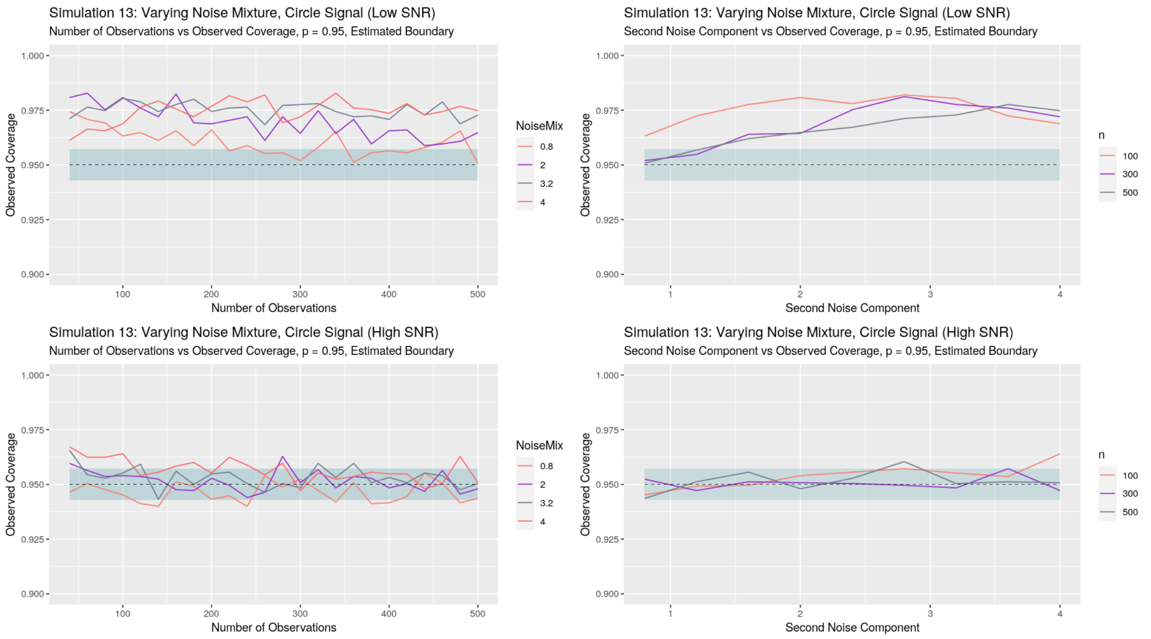

Figure S44: Empirical coverage for simulation 13. Top: Low-SNR synthetic data results. Bottom: High-SNR synthetic data results. Left: Number of observations vs observed coverage, shown for even Gaussian noise mixtures of $N(0,1)$ and $N(0,\sigma^2)$ with $\sigma = 0.8, 2, 3.2$ and 4. Right: Second noise component (σ) vs observed coverage, shown for $n = 100, 300$ and 500.

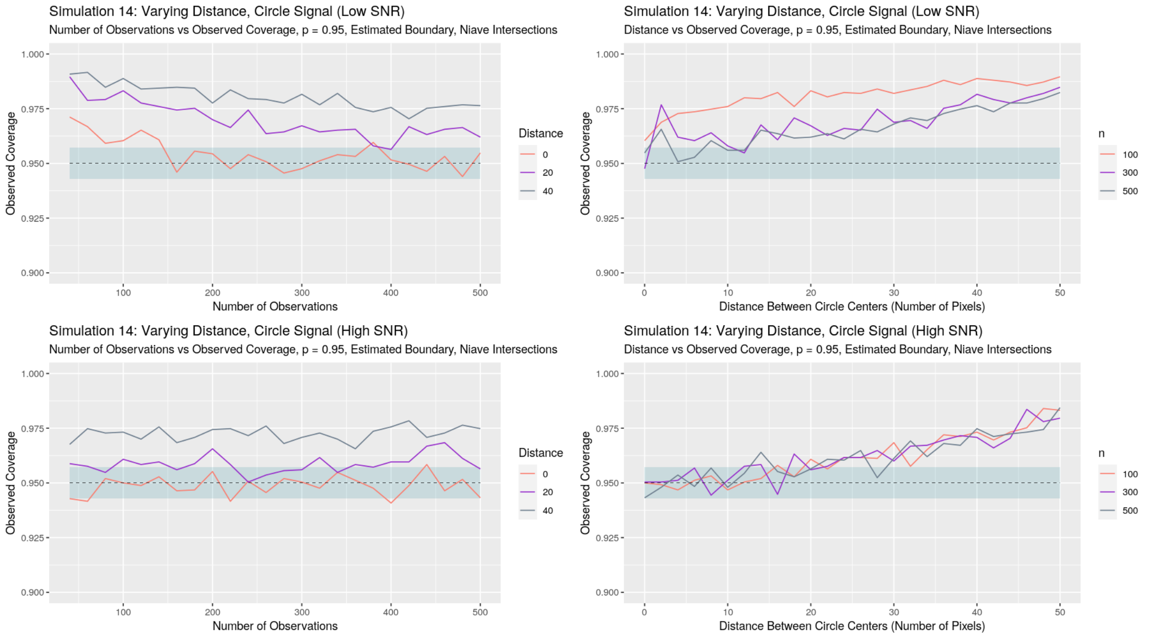

Figure S45: Empirical coverage for simulation 14. Top: Low-SNR synthetic data results. Bottom: High-SNR synthetic data results. Left: Number of observations vs observed coverage, shown for circle separations of 0, 20 and 40 pixels. Right: Separation between circles vs observed coverage, shown for $n = 100, 300$ and 500.

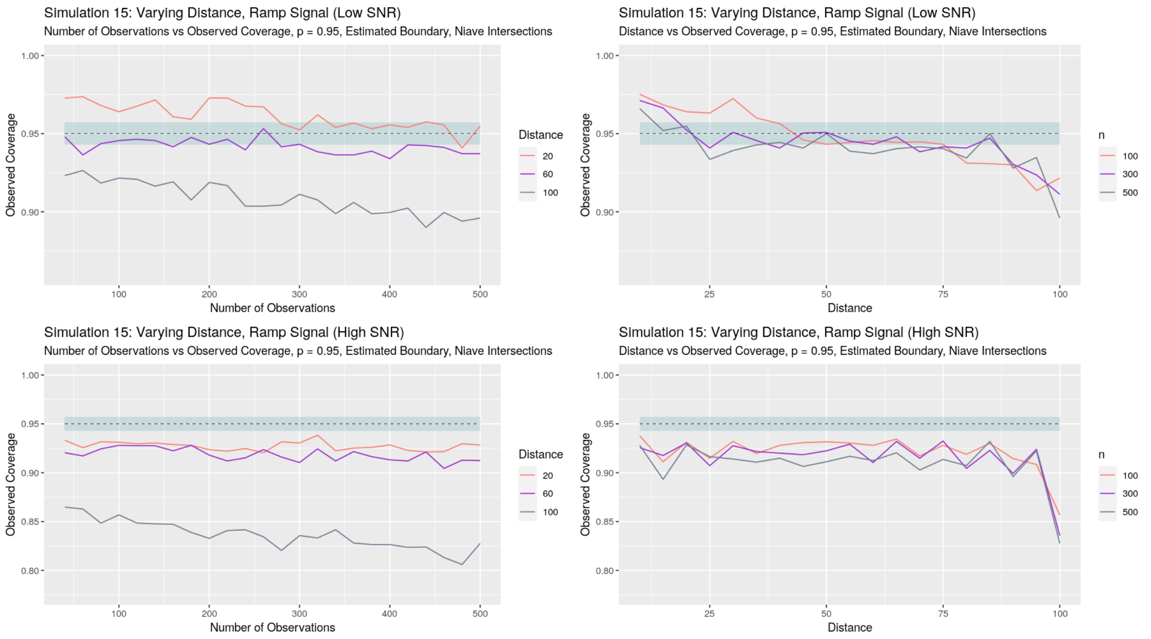

Figure S46: Empirical coverage for simulation 15. Top: Low-SNR synthetic data results. Bottom: High-SNR synthetic data results. Left: Number of observations vs observed coverage, shown for ramp separations of $20, 60$ and 100 pixels. Right: Separation between ramps vs observed coverage, shown for $n = 100, 300$ and 500.

S6 Empirical Coverage Along the True Boundary

In this section, we provide the simulation results that were obtained by using the true boundary, $\partial\mathcal{F}_c$, to evaluate the suprema which appears in Equation (S1). In general, the results in the following sections agree with those of Section S5 and demonstrate the method's robustness to the secondary factors previously listed.

However, unlike in the previous section, the method tended to provide liberal estimates for the CRs (e.g. the inclusion statement, $\hat{\mathcal{F}}_c^+ \subseteq \mathcal{F}_c \subseteq \hat{\mathcal{F}}_c^-$, held "too little") when low-SNR data was used, and the number of observations was low. This effect was most prominent for Simulations 3 and 7, and is corroborated by the previous literature on CRs (c.f. Sommerfeld et al. [2018], Bowring et al. [2019]). As noted by previous authors, the apparent conflict between the method's liberal behaviour in this section and the previous conservative results may be explained by a combination of competing factors.

When performing the method on a discrete lattice, it is expected that the suprema appearing in Equation (S1) will be underestimated (as samples cannot be drawn for the entire continuous region, $\partial\mathcal{F}_c$). This downwards bias is expected to cause an underestimation of the quantiles of H and, consequently, a. In turn, the underestimation of a results in tighter estimates for the CRs $\hat{\mathcal{F}}_c^+$ and $\hat{\mathcal{F}}_c^-$, thus causing the observed liberal behaviour of the method. Based on this argument, it may be natural to ask why no such liberal behaviour was observed in the results of Section S5.

We argue that the previous results were subject to the downward biases described above. However, these downward biases were countered by the upward bias caused by the uncertainty of the location of $\partial\hat{\mathcal{F}}_c$ discussed in the previous section. As the results of the previous section (and of Sommerfeld et al. [2018] and Bowring et al. [2019]) demonstrate, in practice, the upward bias tends to dominate. Consequently, we suggest that the method is more likely to produce conservative estimates than liberal estimates in practice.

This section is organized as follows. Section S6.1 provides the empirical coverage results for $\alpha = 0.2$, Section S6.2 provides equivalent results for $\alpha = 0.1$ and Section S6.3 provides results for $\alpha = 0.05$. In all plots, nominal coverage is shown as a dotted gray line and a corresponding binomial confidence interval is shaded in blue. All data points displayed are averages taken across 2500 simulation instances, each using 5000 bootstrap realizations.

S6.1 Nominal Coverage: 80%

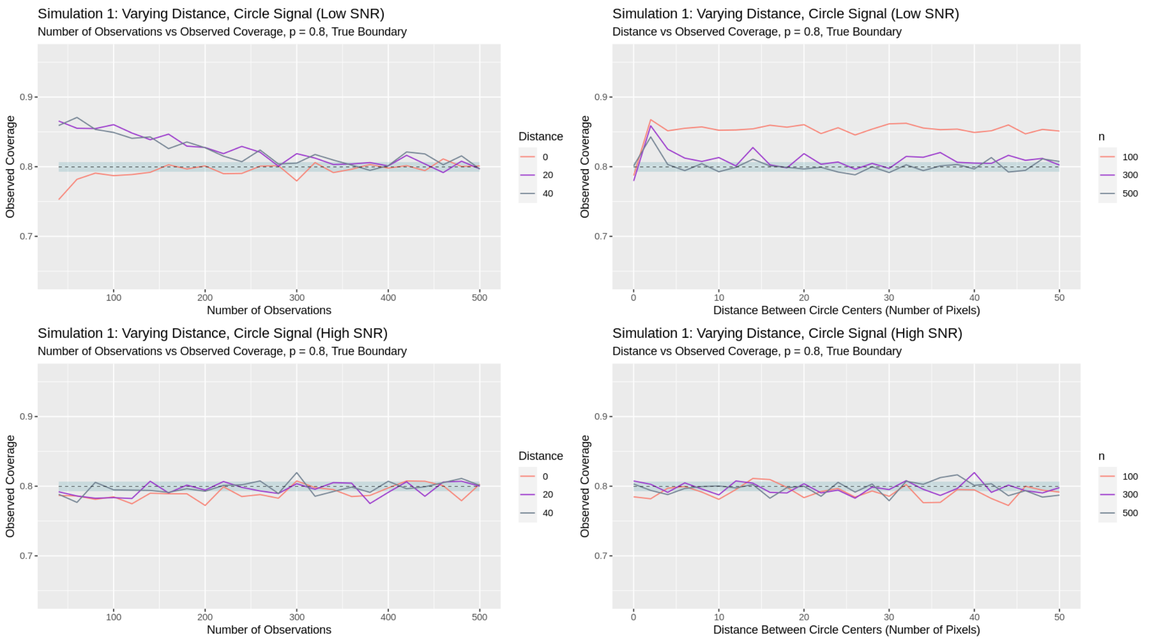

Figure S47: Empirical coverage for simulation 1. Top: Low-SNR synthetic data results. Bottom: High-SNR synthetic data results. Left: Number of observations vs observed coverage, shown for circle separations of $0, 20$ and 40 pixels. Right: Separation between circles vs observed coverage, shown for $n = 100, 300$ and 500.

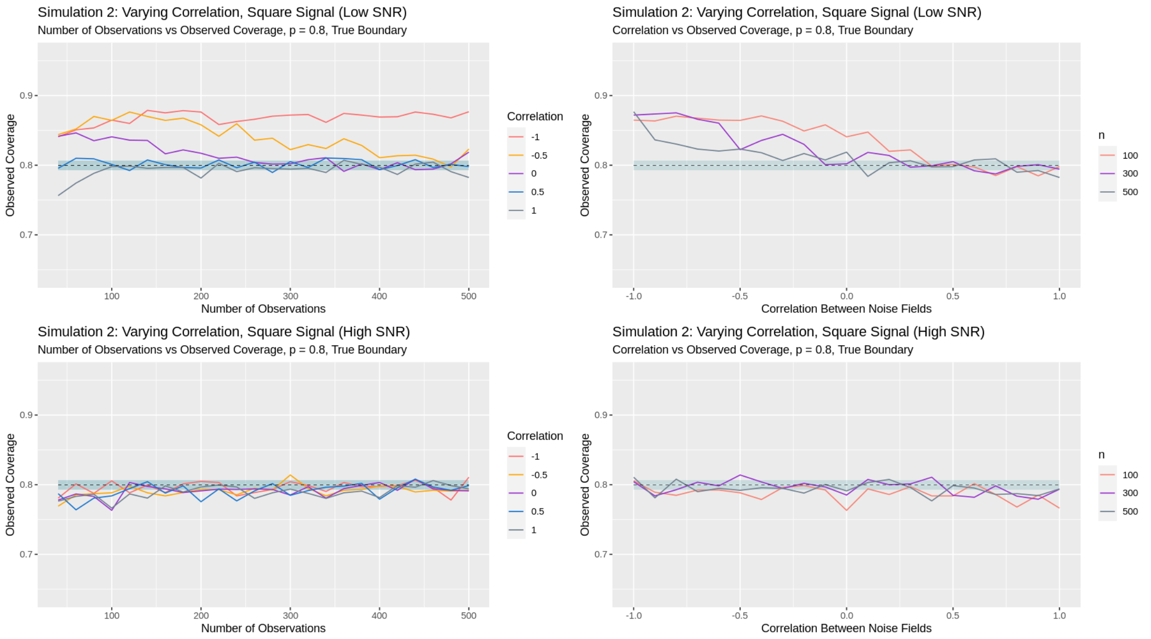

Figure S48: Empirical coverage for simulation 2. Top: Low-SNR synthetic data results. Bottom: High-SNR synthetic data results. Left: Number of observations vs observed coverage, shown for correlations of $-1, -0.5, 0, 0.5$ and 1. Right: Correlation between noise fields vs observed coverage, shown for $n = 100, 300$ and 500.

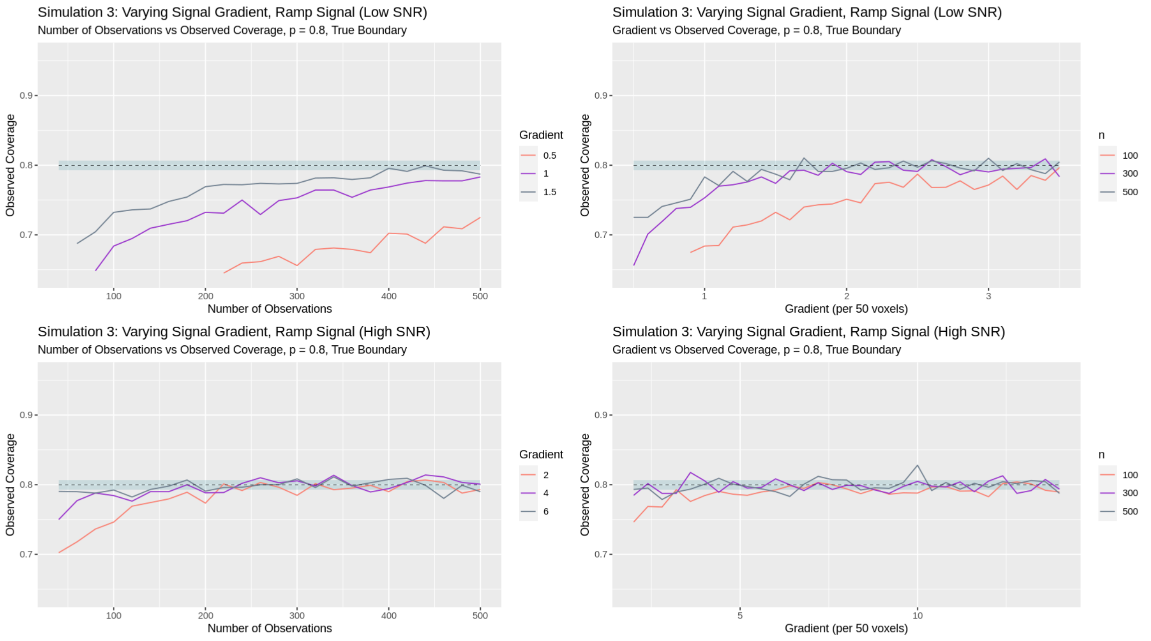

Figure S49: Empirical coverage for simulation 3. Top: Low-SNR synthetic data results. Bottom: High-SNR synthetic data results. Left: Number of observations vs observed coverage, shown for gradients of $0.5, 1$ and 1.5 per 50 pixels (top), and $2, 4$ and 6 per 50 pixels (bottom). Right: Ramp gradient per 50 pixels vs observed coverage, shown for $n = 100, 300$ and 500.

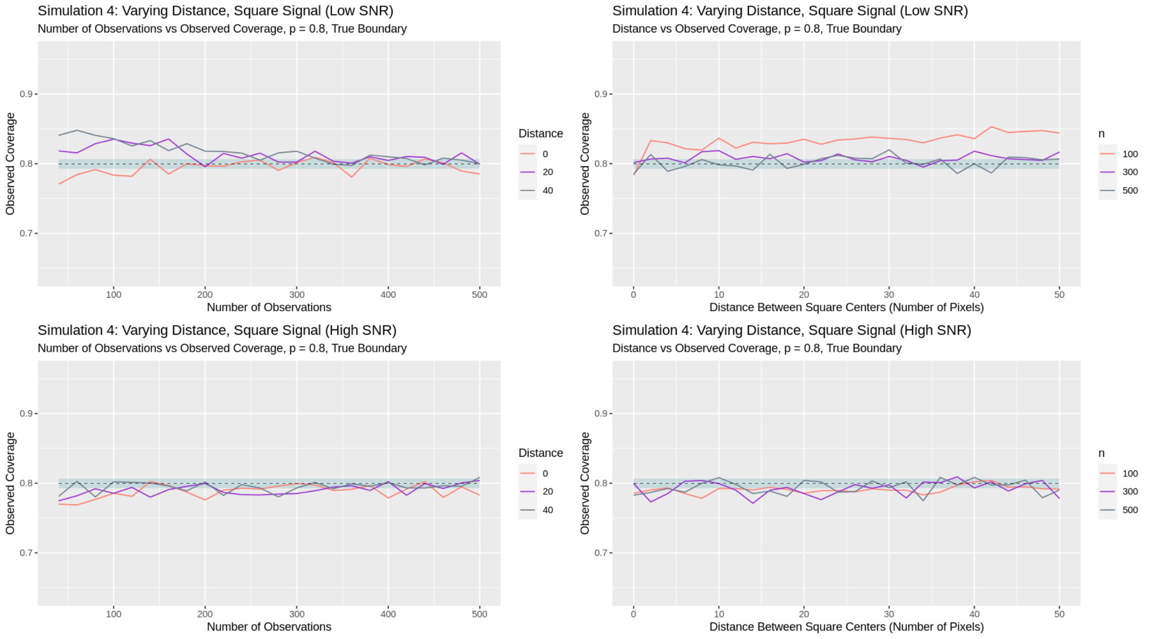

Figure S50: Empirical coverage for simulation 4. Top: Low-SNR synthetic data results. Bottom: High-SNR synthetic data results. Left: Number of observations vs observed coverage, shown for square separations of $0, 20$ and 40 pixels. Right: Separation between squares vs observed coverage, shown for $n = 100, 300$ and 500.

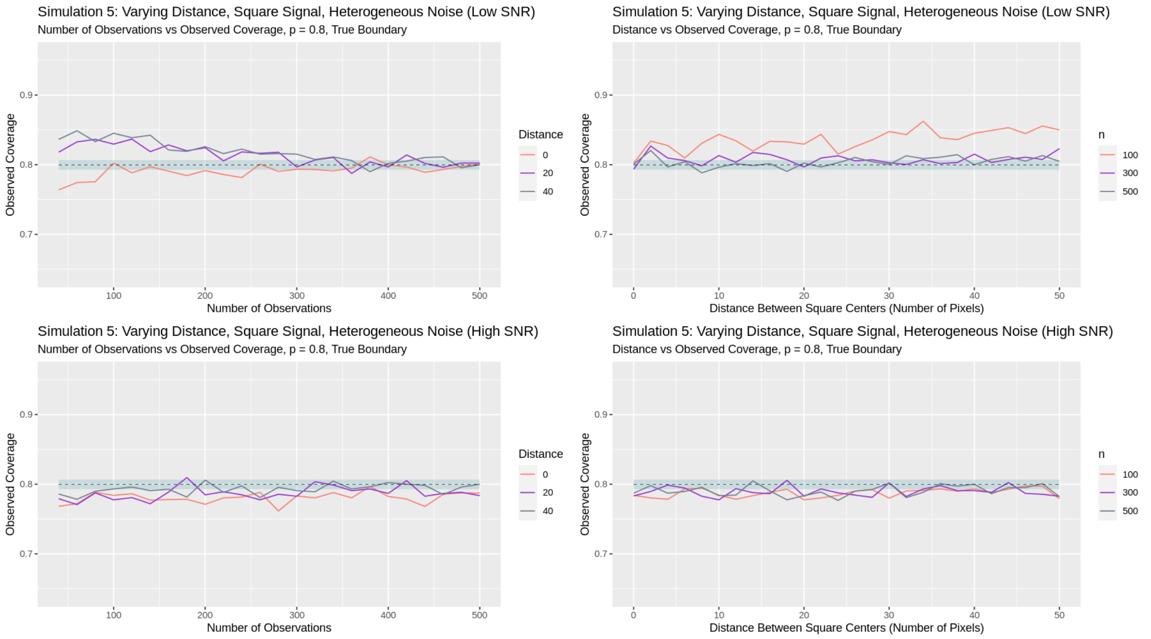

Figure S51: Empirical coverage for simulation 5. Top: Low-SNR synthetic data results. Bottom: High-SNR synthetic data results. Left: Number of observations vs observed coverage, shown for square separations of $0, 20$ and 40 pixels. Right: Separation between squares vs observed coverage, shown for $n = 100, 300$ and 500.

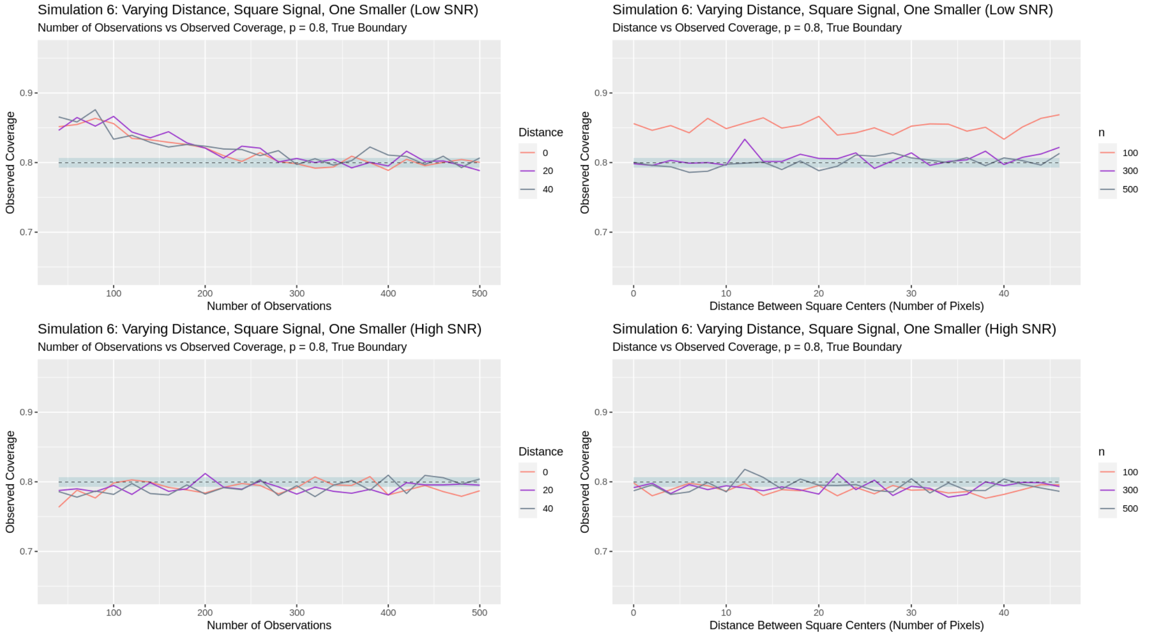

Figure S52: Empirical coverage for simulation 6. Top: Low-SNR synthetic data results. Bottom: High-SNR synthetic data results. Left: Number of observations vs observed coverage, shown for square separations of $0, 20$ and 40 pixels. Right: Separation between squares vs observed coverage, shown for $n = 100, 300$ and 500.

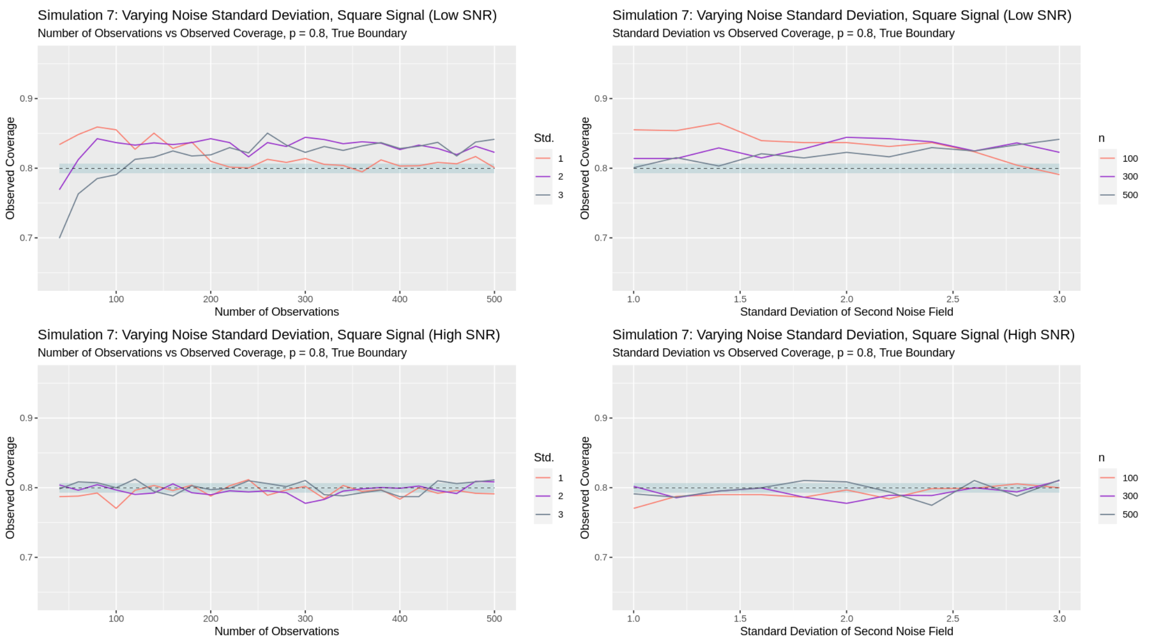

Figure S53: Empirical coverage for simulation 7. Top: Low-SNR synthetic data results. Bottom: High-SNR synthetic data results. Left: Number of observations vs observed coverage, shown for noise standard deviations of $1, 2$ and 3. Right: The standard deviation of the second noise field vs observed coverage, shown for $n = 100, 300$ and 500.

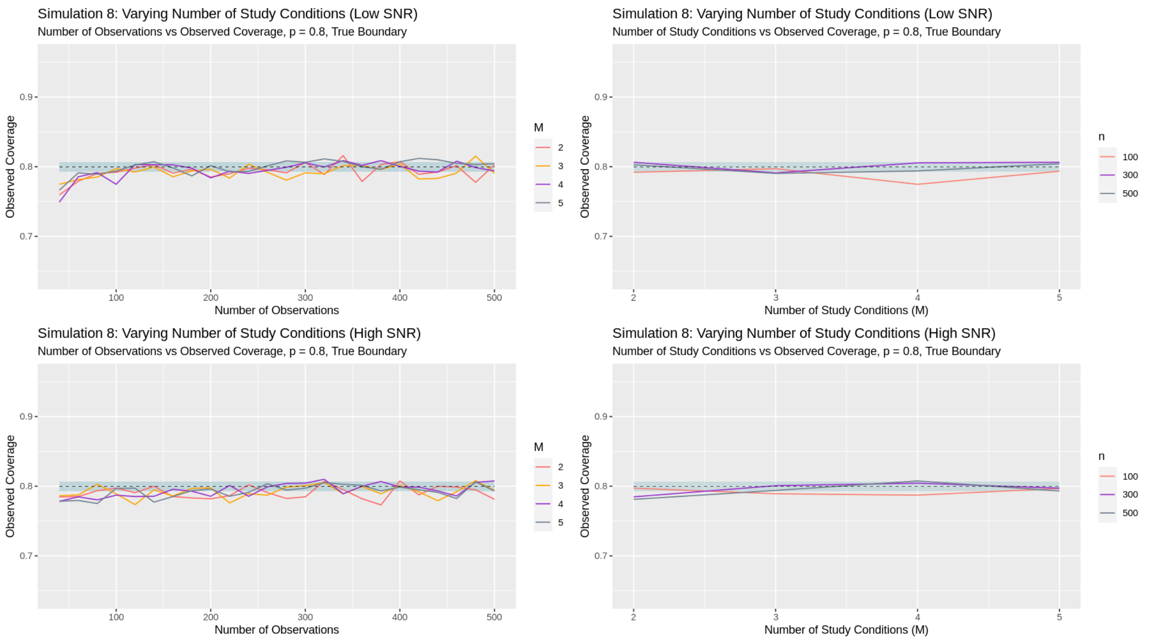

Figure S54: Empirical coverage for simulation 8. Top: Low-SNR synthetic data results. Bottom: High-SNR synthetic data results. Left: Number of observations vs observed coverage, shown for $M = 2, 3, 4$ and 5. Right: Number of study conditions (M) vs observed coverage, shown for $n = 100, 300$ and 500.

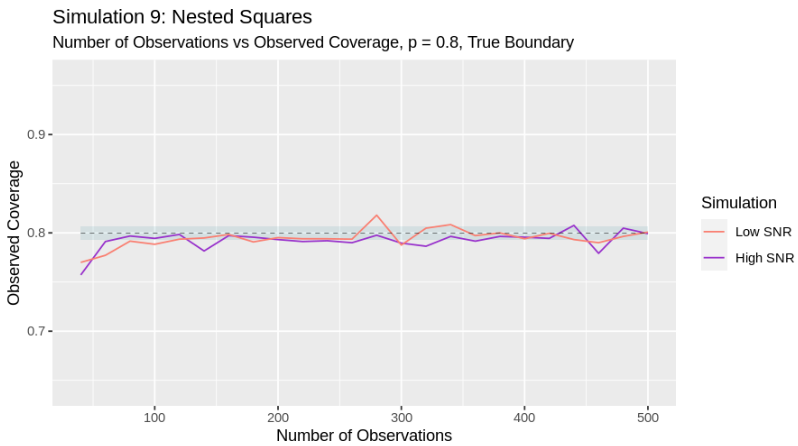

Figure S55: Empirical coverage for simulation 9. Results are shown for both low-SNR synthetic data and high-SNR synthetic data. Displayed is the number of observations vs observed coverage.

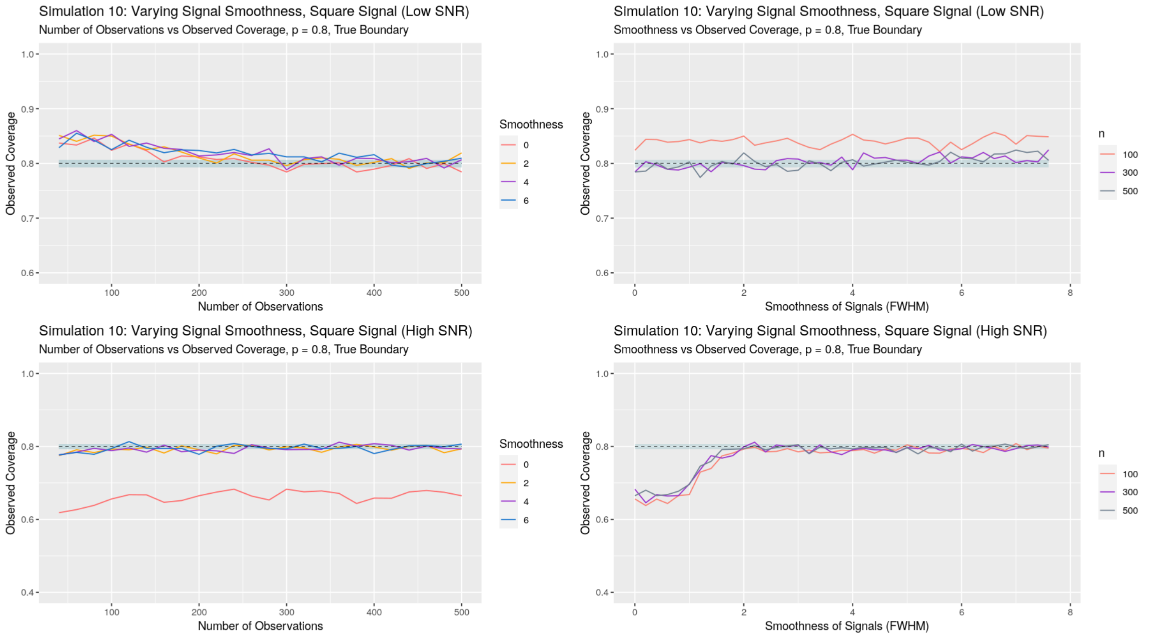

Figure S56: Empirical coverage for simulation 10. Top: Low-SNR synthetic data results. Bottom: High-SNR synthetic data results. Left: Number of observations vs observed coverage, shown for signal smoothness of $0, 2, 4$ and 6 FWHM. Right: Signal smoothness vs observed coverage, shown for $n = 100, 300$ and 500.

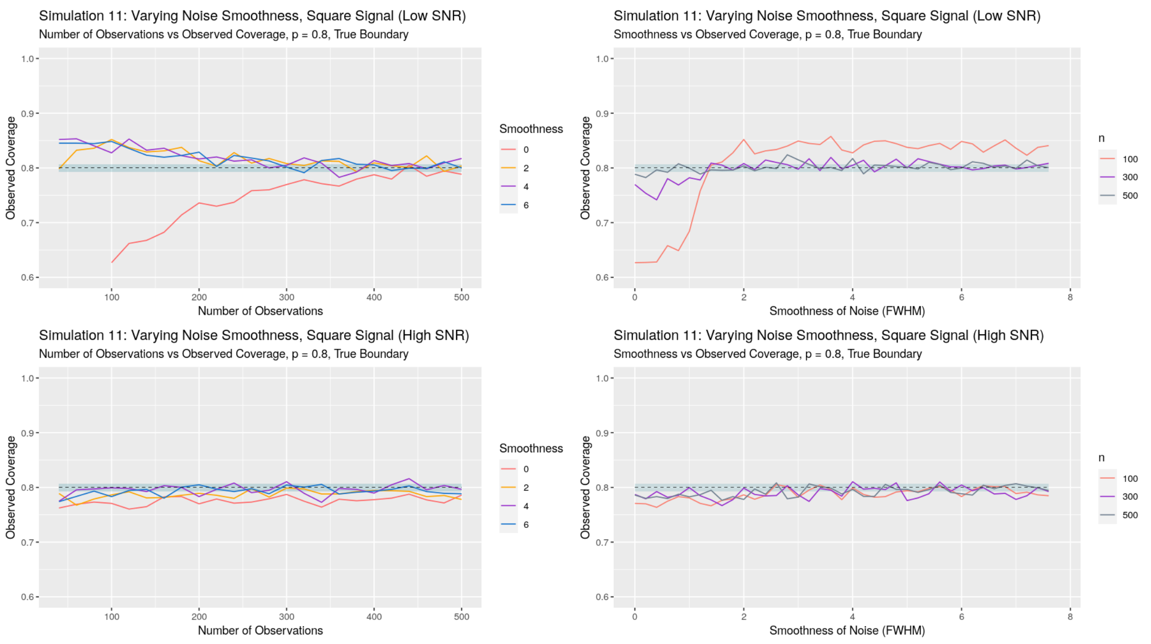

Figure S57: Empirical coverage for simulation 11. Top: Low-SNR synthetic data results. Bottom: High-SNR synthetic data results. Left: Number of observations vs observed coverage, shown for noise smoothness of $0, 2, 4$ and 6 FWHM. Right: Noise smoothness vs observed coverage, shown for $n = 100, 300$ and 500.

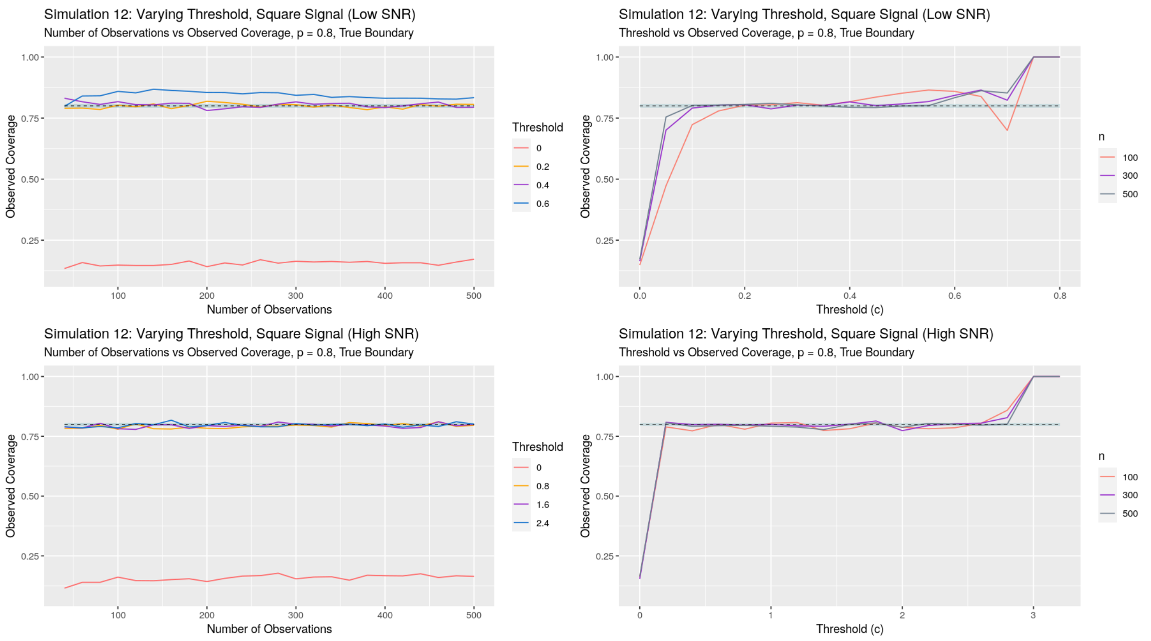

Figure S58: Empirical coverage for simulation 12. Top: Low-SNR synthetic data results. Bottom: High-SNR synthetic data results. Left: Number of observations vs observed coverage, shown for thresholds $c = 0, 0.2, 0.4$ and 0.6 (top) and $c = 0, 0.8, 1.6$ and 2.4 (bottom). Right: Threshold vs observed coverage, shown for $n = 100, 300$ and 500.

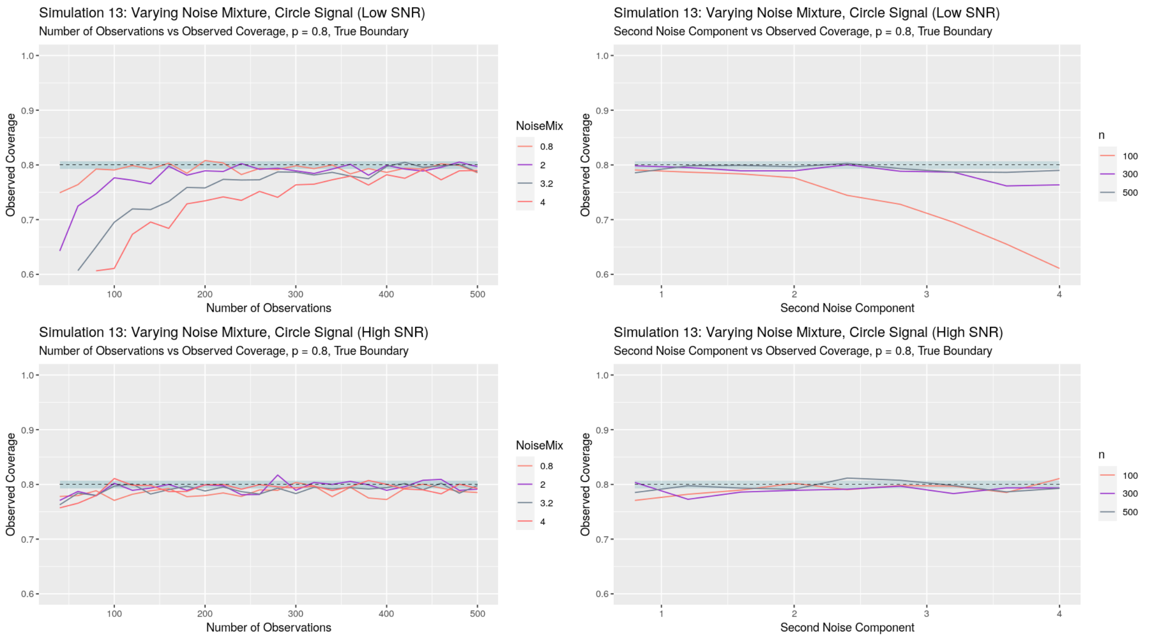

Figure S59: Empirical coverage for simulation 13. Top: Low-SNR synthetic data results. Bottom: High-SNR synthetic data results. Left: Number of observations vs observed coverage, shown for even Gaussian noise mixtures of $N(0,1)$ and $N(0,\sigma^2)$ with $\sigma = 0.8, 2, 3.2$ and 4. Right: Second noise component (σ) vs observed coverage, shown for $n = 100, 300$ and 500.

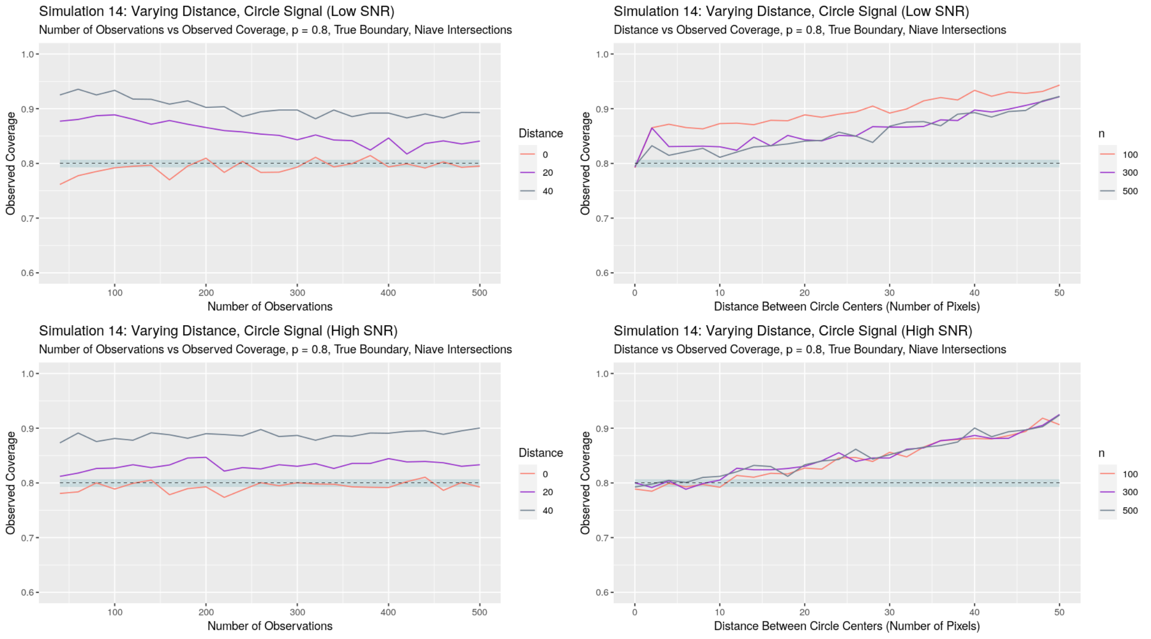

Figure S60: Empirical coverage for simulation 14. Top: Low-SNR synthetic data results. Bottom: High-SNR synthetic data results. Left: Number of observations vs observed coverage, shown for circle separations of 0, 20 and 40 pixels. Right: Separation between circles vs observed coverage, shown for $n = 100, 300$ and 500.

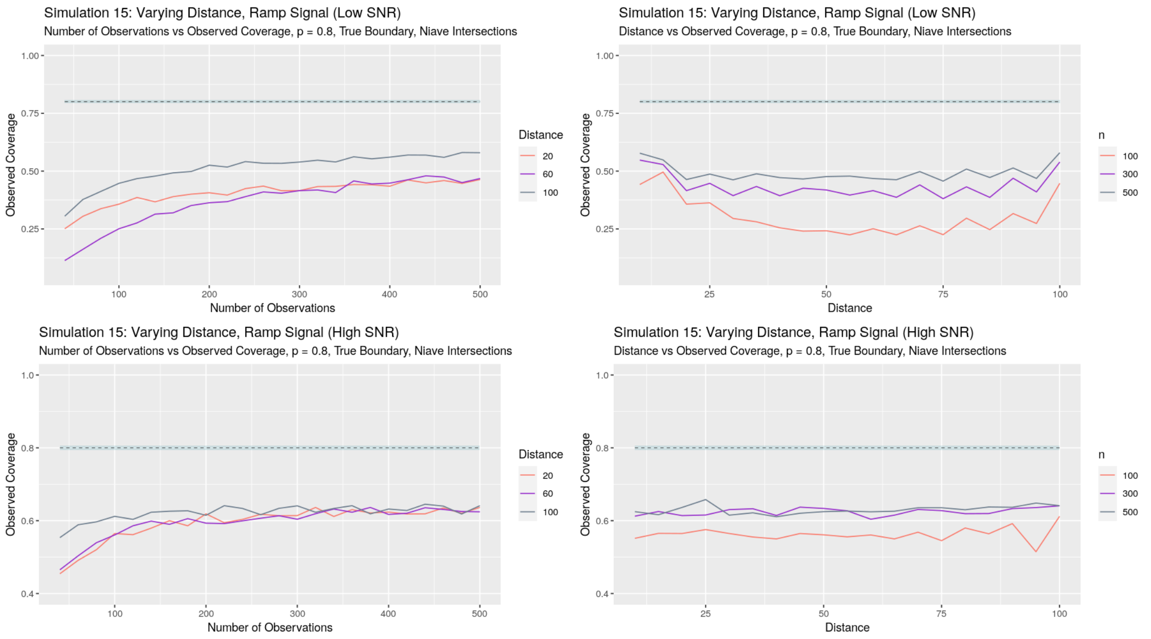

Figure S61: Empirical coverage for simulation 15. Top: Low-SNR synthetic data results. Bottom: High-SNR synthetic data results. Left: Number of observations vs observed coverage, shown for ramp separations of $20, 60$ and 100 pixels. Right: Separation between ramps vs observed coverage, shown for $n = 100, 300$ and 500.

S6.2 Nominal Coverage: 90%

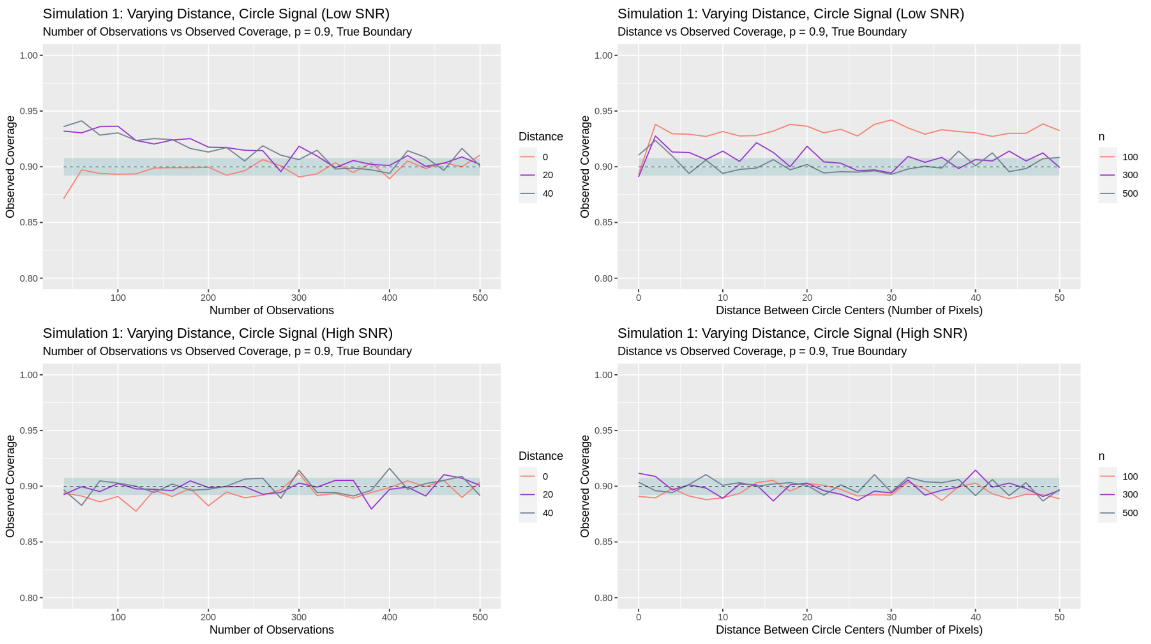

Figure S62: Empirical coverage for simulation 1. Top: Low-SNR synthetic data results. Bottom: High-SNR synthetic data results. Left: Number of observations vs observed coverage, shown for circle separations of $0, 20$ and 40 pixels. Right: Separation between circles vs observed coverage, shown for $n = 100, 300$ and 500.

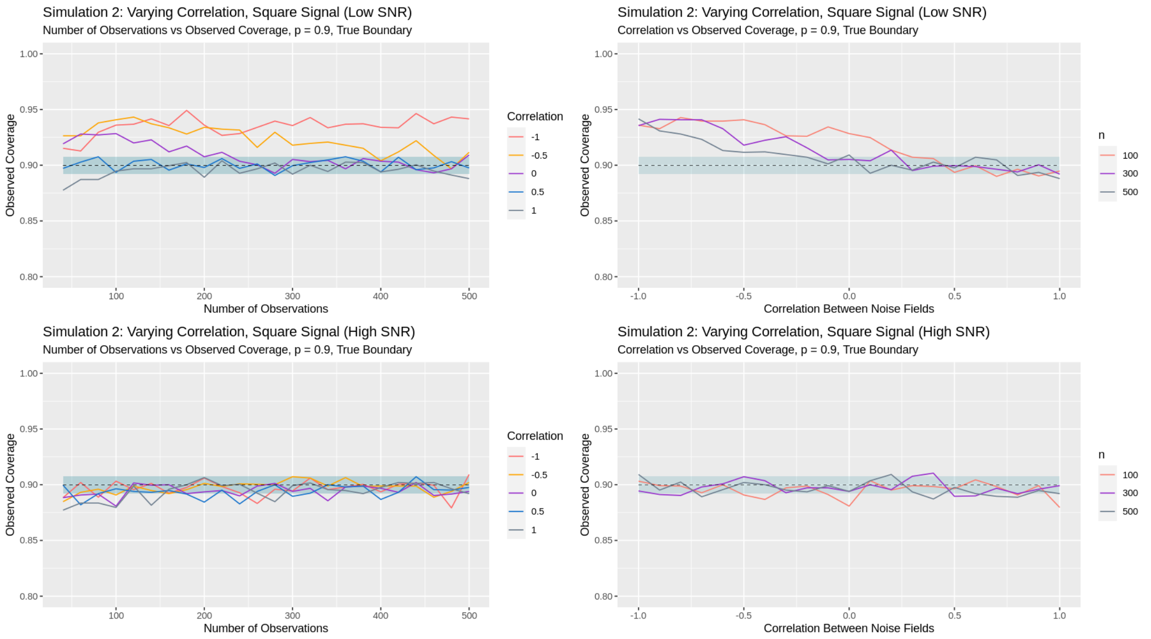

Figure S63: Empirical coverage for simulation 2. Top: Low-SNR synthetic data results. Bottom: High-SNR synthetic data results. Left: Number of observations vs observed coverage, shown for correlations of $-1, -0.5, 0, 0.5$ and 1. Right: Correlation between noise fields vs observed coverage, shown for $n = 100, 300$ and 500.

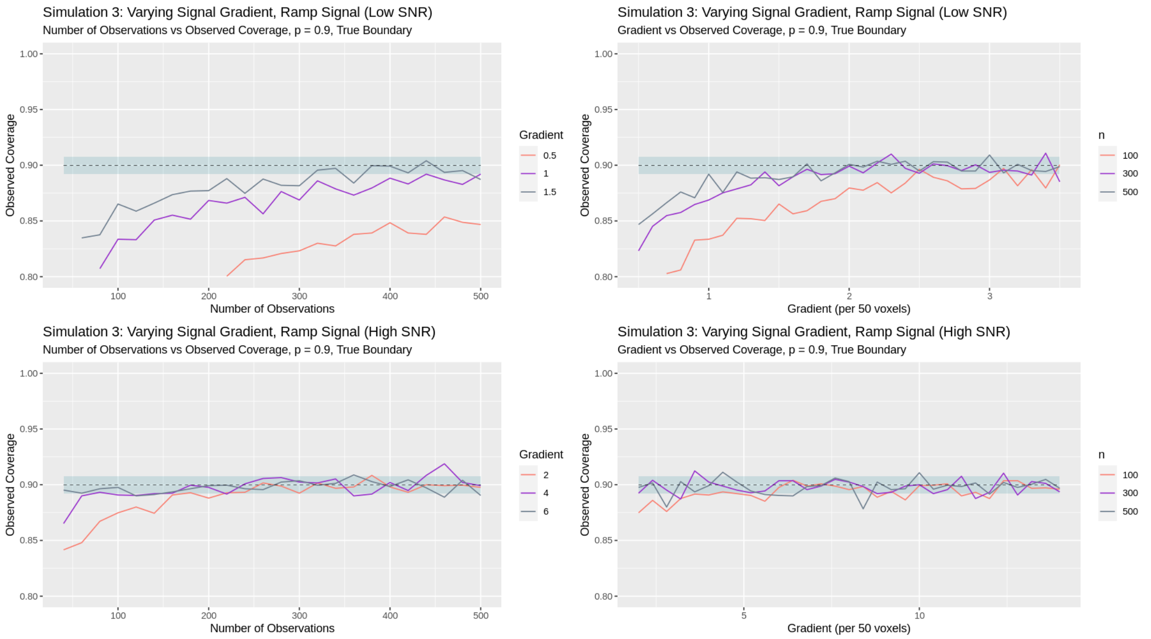

Figure S64: Empirical coverage for simulation 3. Top: Low-SNR synthetic data results. Bottom: High-SNR synthetic data results. Left: Number of observations vs observed coverage, shown for gradients of $0.5, 1$ and 1.5 per 50 pixels (top), and $2, 4$ and 6 per 50 pixels (bottom). Right: Ramp gradient per 50 pixels vs observed coverage, shown for $n = 100, 300$ and 500.

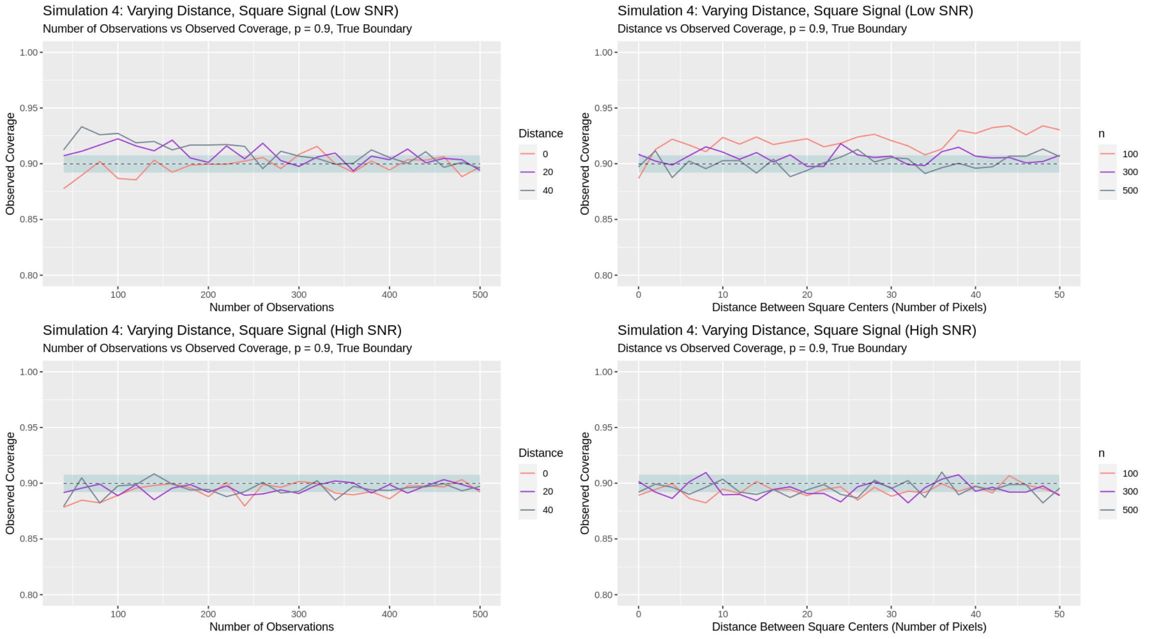

Figure S65: Empirical coverage for simulation 4. Top: Low-SNR synthetic data results. Bottom: High-SNR synthetic data results. Left: Number of observations vs observed coverage, shown for square separations of $0, 20$ and 40 pixels. Right: Separation between squares vs observed coverage, shown for $n = 100, 300$ and 500.

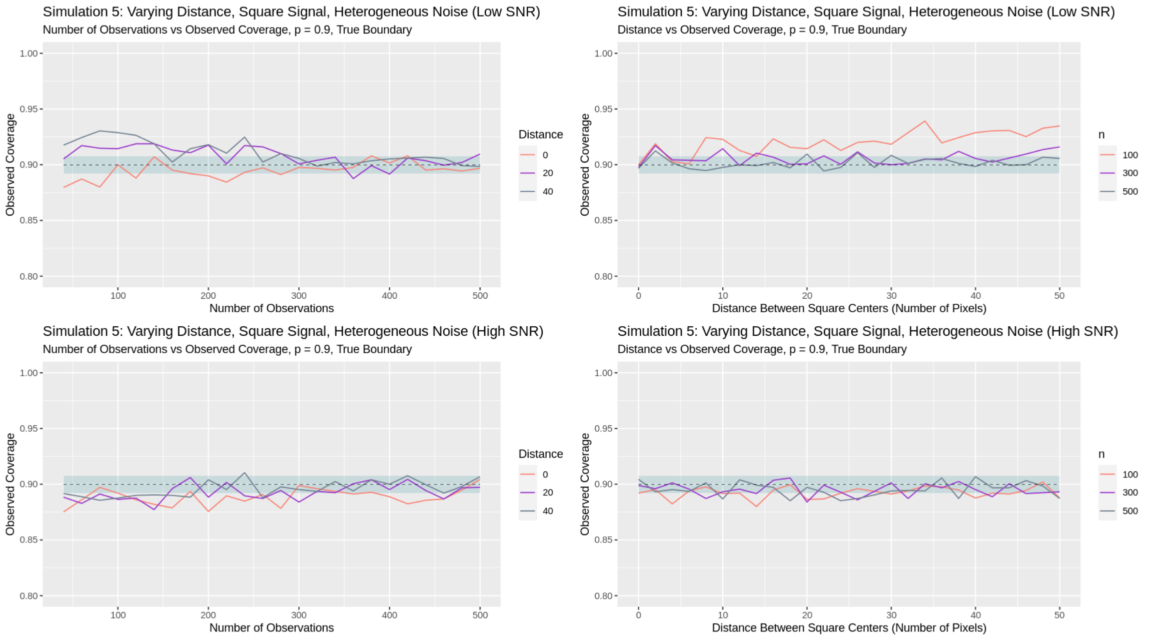

Figure S66: Empirical coverage for simulation 5. Top: Low-SNR synthetic data results. Bottom: High-SNR synthetic data results. Left: Number of observations vs observed coverage, shown for square separations of $0, 20$ and 40 pixels. Right: Separation between squares vs observed coverage, shown for $n = 100, 300$ and 500.

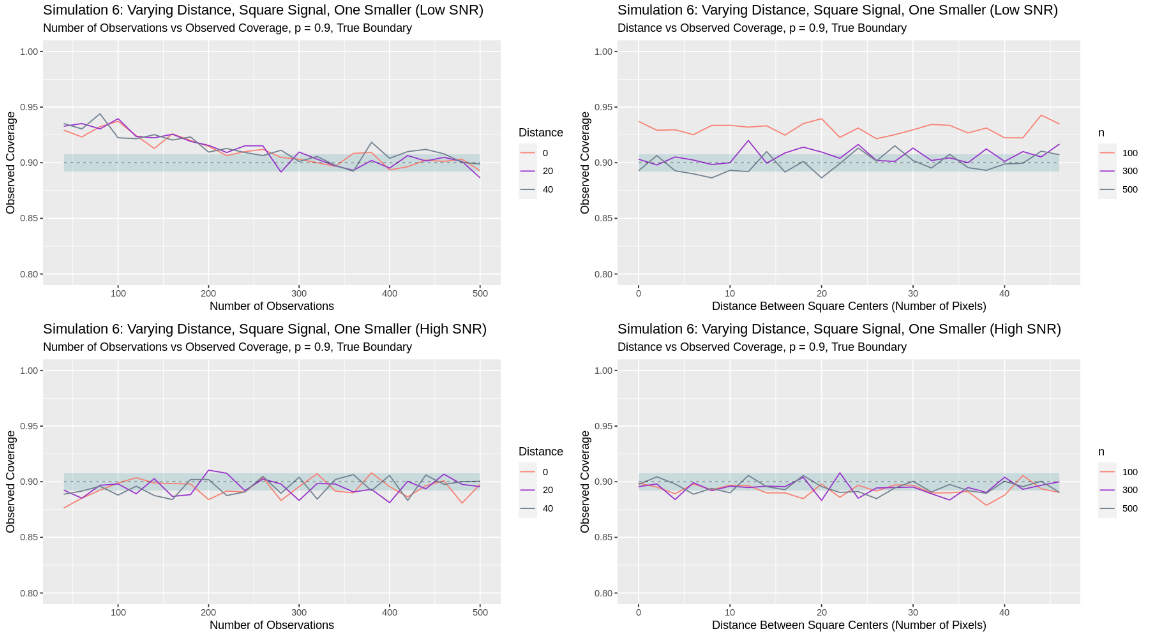

Figure S67: Empirical coverage for simulation 6. Top: Low-SNR synthetic data results. Bottom: High-SNR synthetic data results. Left: Number of observations vs observed coverage, shown for square separations of $0, 20$ and 40 pixels. Right: Separation between squares vs observed coverage, shown for $n = 100, 300$ and 500.

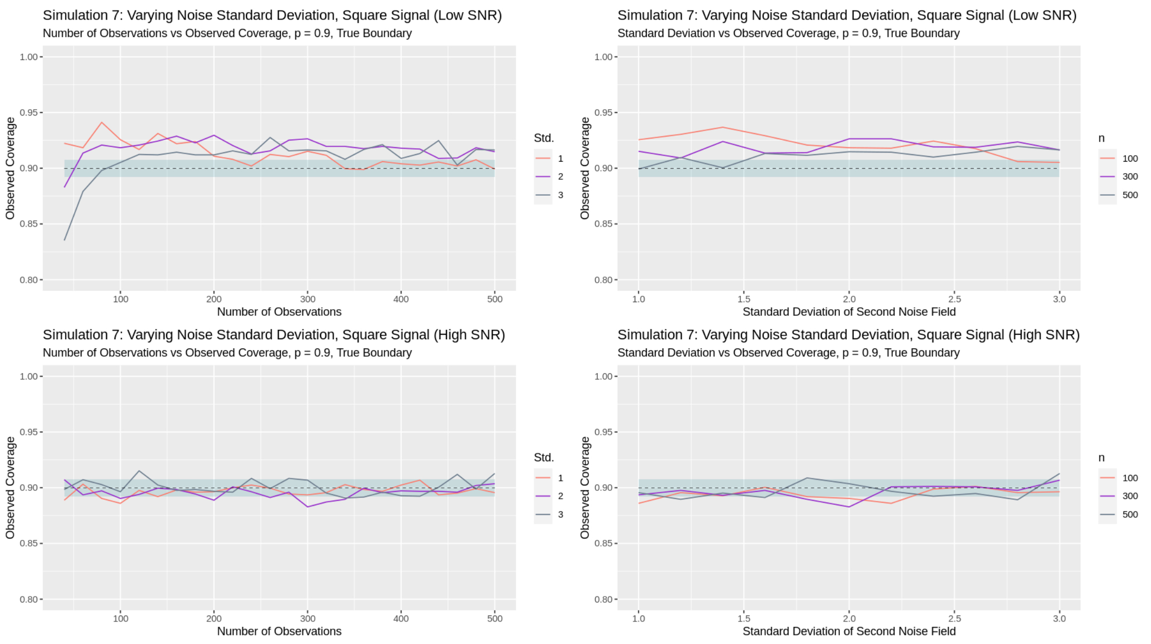

Figure S68: Empirical coverage for simulation 7. Top: Low-SNR synthetic data results. Bottom: High-SNR synthetic data results. Left: Number of observations vs observed coverage, shown for noise standard deviations of $1, 2$ and 3. Right: The standard deviation of the second noise field vs observed coverage, shown for $n = 100, 300$ and 500.

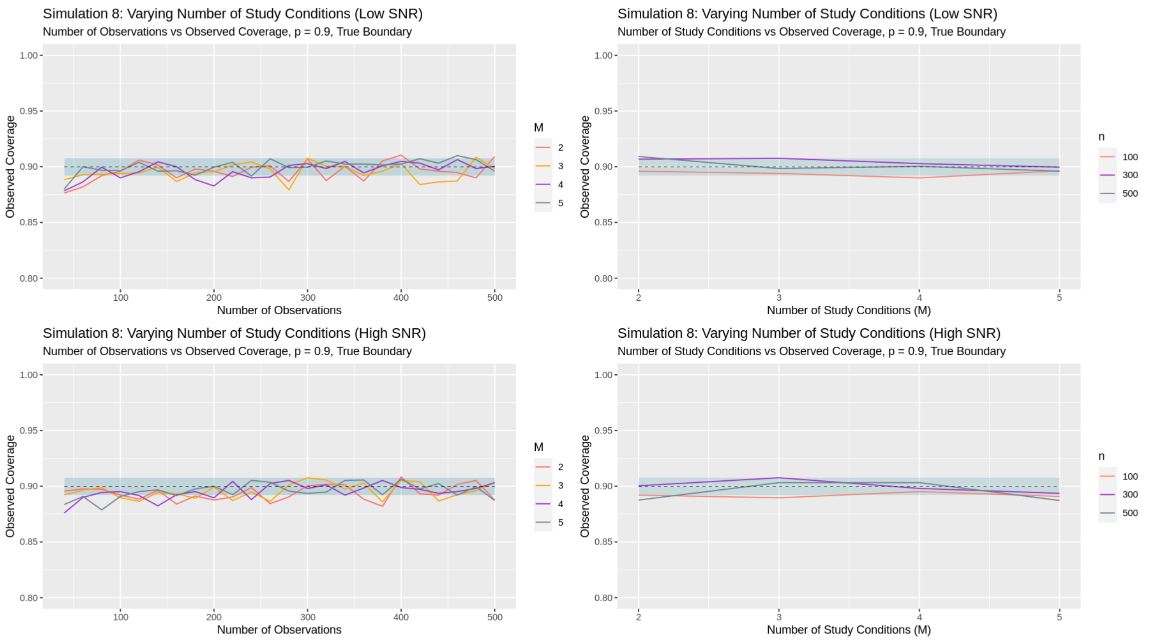

Figure S69: Empirical coverage for simulation 8. Top: Low-SNR synthetic data results. Bottom: High-SNR synthetic data results. Left: Number of observations vs observed coverage, shown for $M = 2, 3, 4$ and 5. Right: Number of study conditions (M) vs observed coverage, shown for $n = 100, 300$ and 500.

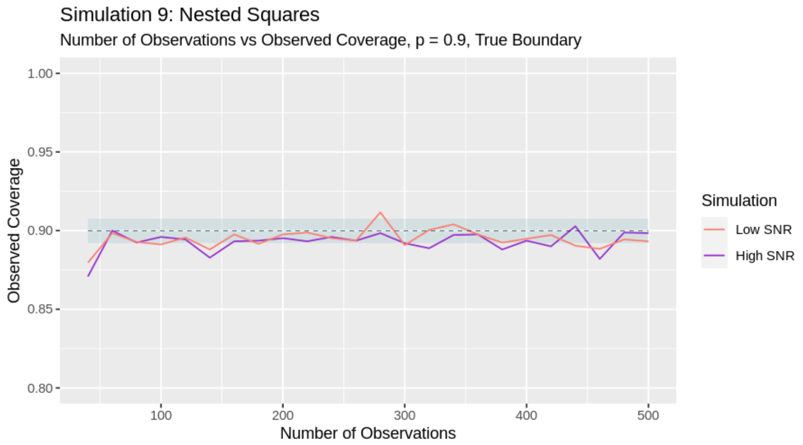

Figure S70: Empirical coverage for simulation 9. Results are shown for both low-SNR synthetic data and high-SNR synthetic data. Displayed is the number of observations vs observed coverage.

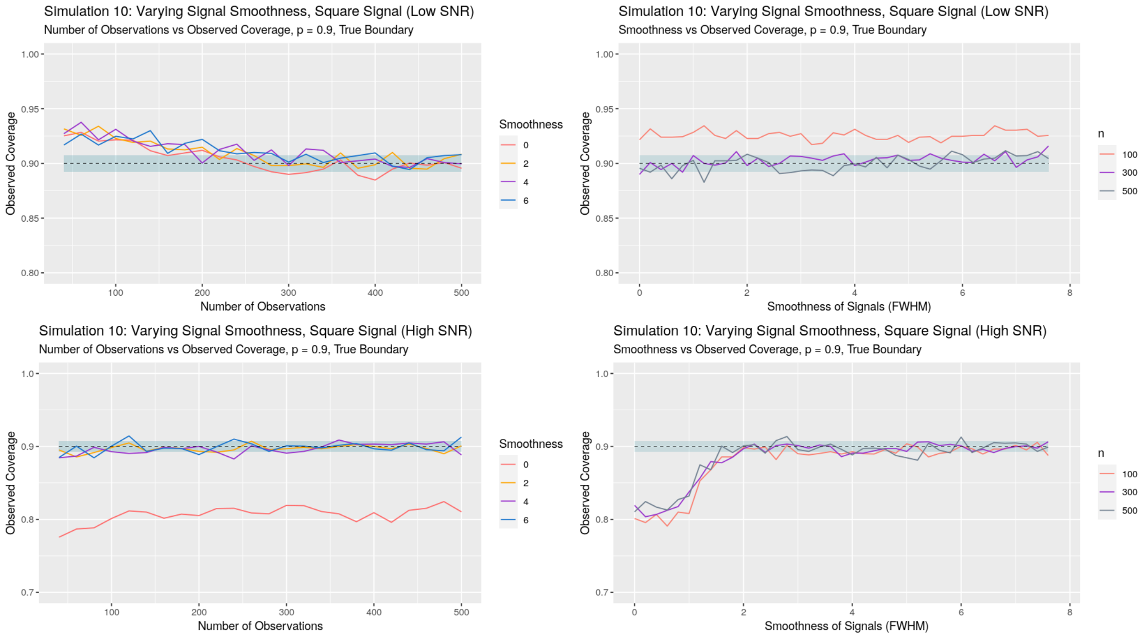

Figure S71: Empirical coverage for simulation 10. Top: Low-SNR synthetic data results. Bottom: High-SNR synthetic data results. Left: Number of observations vs observed coverage, shown for signal smoothness of $0, 2, 4$ and 6 FWHM. Right: Signal smoothness vs observed coverage, shown for $n = 100, 300$ and 500.

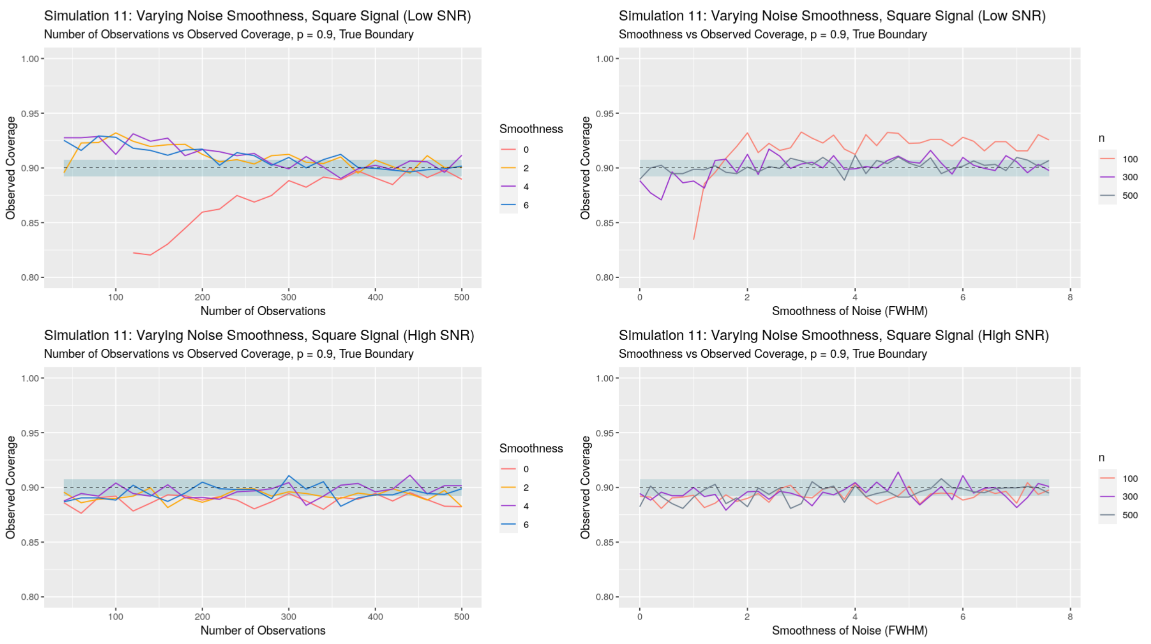

Figure S72: Empirical coverage for simulation 11. Top: Low-SNR synthetic data results. Bottom: High-SNR synthetic data results. Left: Number of observations vs observed coverage, shown for noise smoothness of $0, 2, 4$ and 6 FWHM. Right: Noise smoothness vs observed coverage, shown for $n = 100, 300$ and 500.

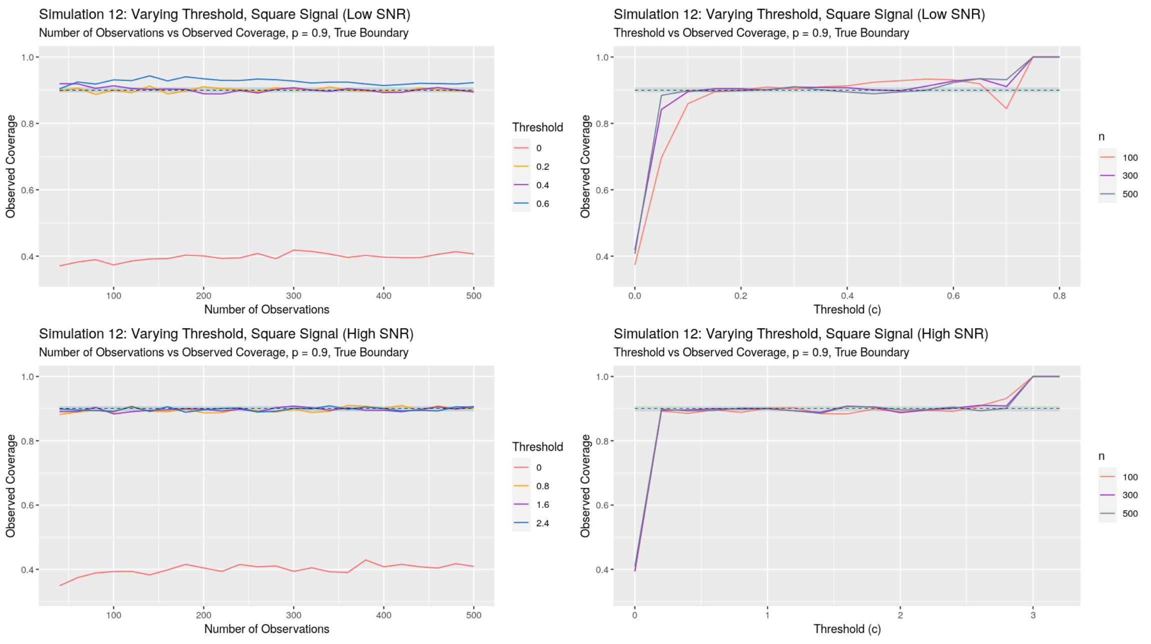

Figure S73: Empirical coverage for simulation 12. Top: Low-SNR synthetic data results. Bottom: High-SNR synthetic data results. Left: Number of observations vs observed coverage, shown for thresholds $c = 0, 0.2, 0.4$ and 0.6 (top) and $c = 0, 0.8, 1.6$ and 2.4 (bottom). Right: Threshold vs observed coverage, shown for $n = 100, 300$ and 500.

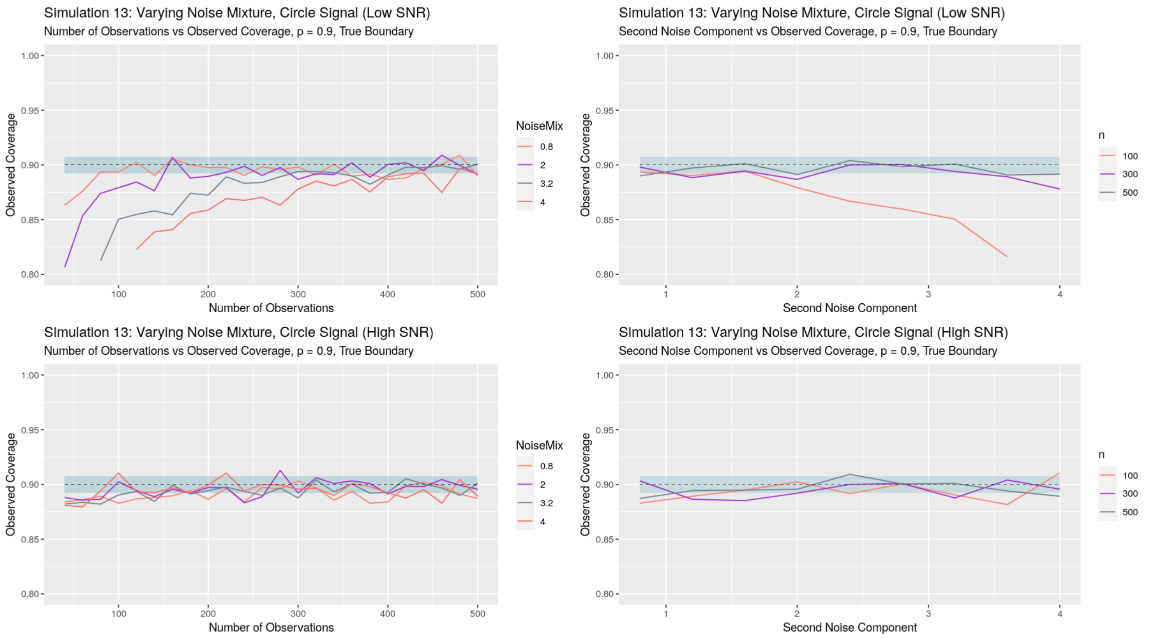

Figure S74: Empirical coverage for simulation 13. Top: Low-SNR synthetic data results. Bottom: High-SNR synthetic data results. Left: Number of observations vs observed coverage, shown for even Gaussian noise mixtures of $N(0,1)$ and $N(0,\sigma^2)$ with $\sigma = 0.8, 2, 3.2$ and 4. Right: Second noise component (σ) vs observed coverage, shown for $n = 100, 300$ and 500.

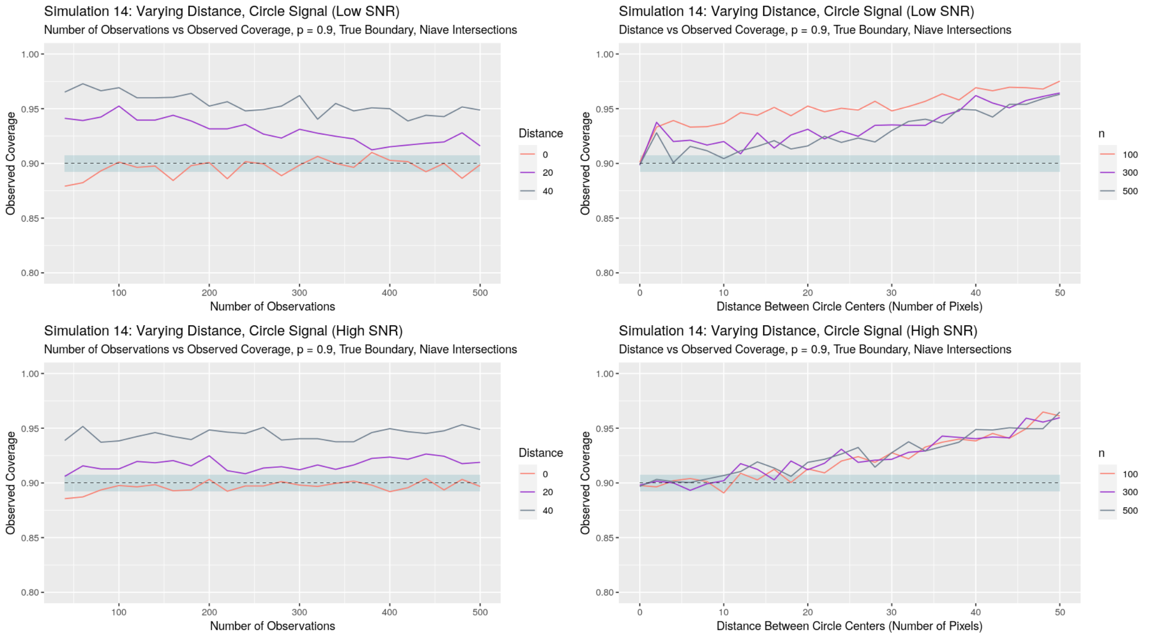

Figure S75: Empirical coverage for simulation 14. Top: Low-SNR synthetic data results. Bottom: High-SNR synthetic data results. Left: Number of observations vs observed coverage, shown for circle separations of 0, 20 and 40 pixels. Right: Separation between circles vs observed coverage, shown for $n = 100, 300$ and 500.

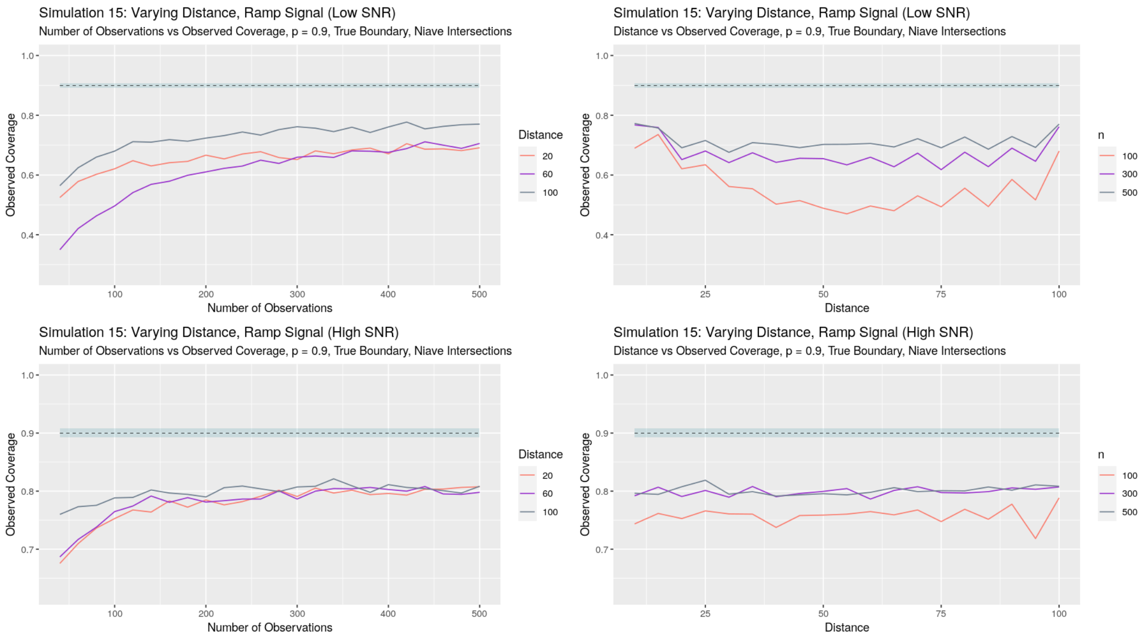

Figure S76: Empirical coverage for simulation 15. Top: Low-SNR synthetic data results. Bottom: High-SNR synthetic data results. Left: Number of observations vs observed coverage, shown for ramp separations of $20, 60$ and 100 pixels. Right: Separation between ramps vs observed coverage, shown for $n = 100, 300$ and 500.

S6.3 Nominal Coverage: 95%

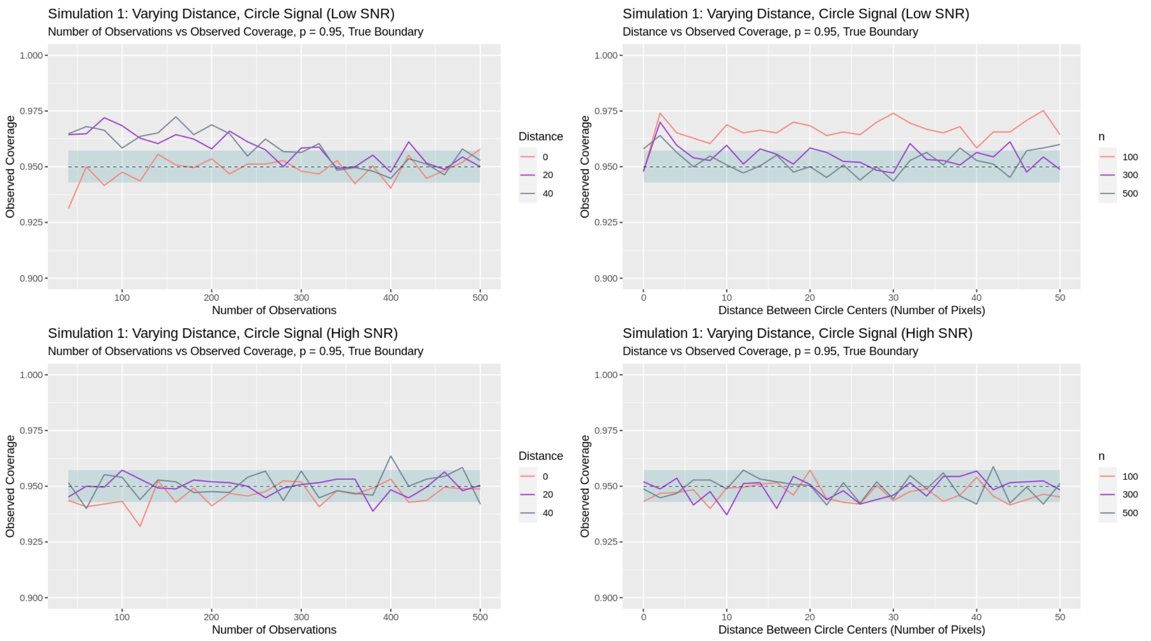

Figure S77: Empirical coverage for simulation 1. Top: Low-SNR synthetic data results. Bottom: High-SNR synthetic data results. Left: Number of observations vs observed coverage, shown for circle separations of $0, 20$ and 40 pixels. Right: Separation between circles vs observed coverage, shown for $n = 100, 300$ and 500.

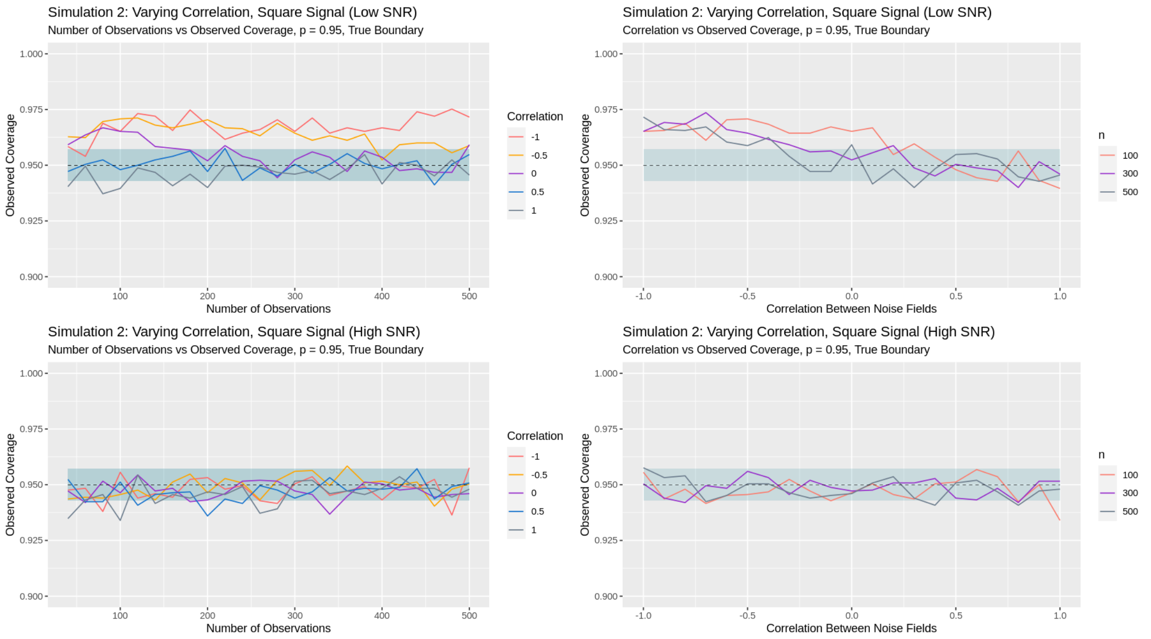

Figure S78: Empirical coverage for simulation 2. Top: Low-SNR synthetic data results. Bottom: High-SNR synthetic data results. Left: Number of observations vs observed coverage, shown for correlations of $-1, -0.5, 0, 0.5$ and 1. Right: Correlation between noise fields vs observed coverage, shown for $n = 100, 300$ and 500.

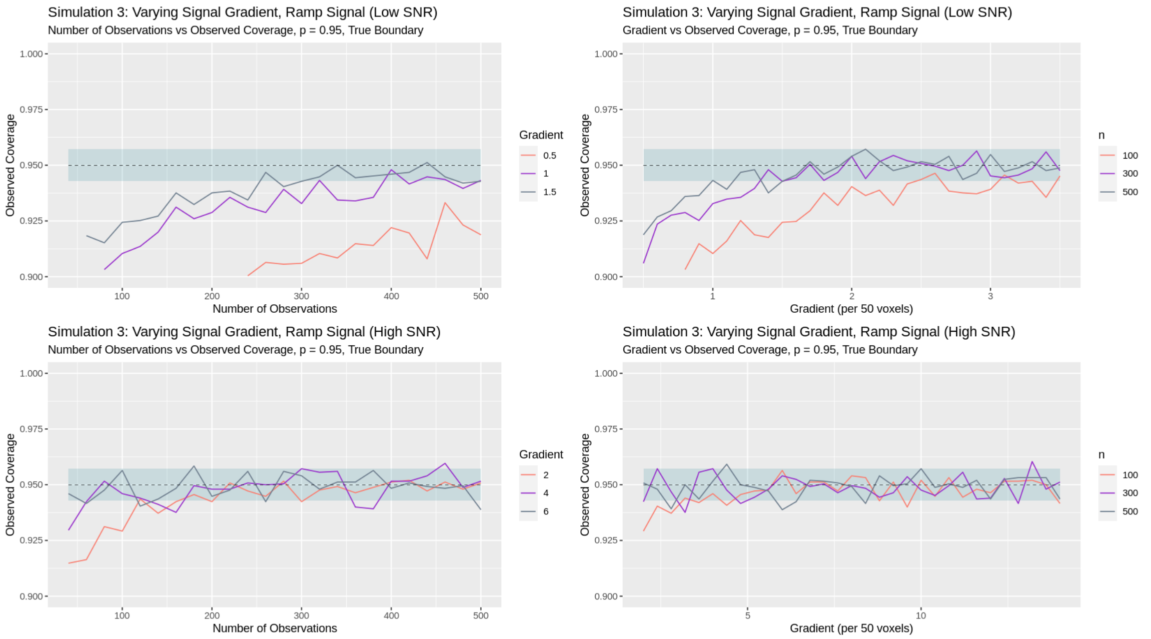

Figure S79: Empirical coverage for simulation 3. Top: Low-SNR synthetic data results. Bottom: High-SNR synthetic data results. Left: Number of observations vs observed coverage, shown for gradients of $0.5, 1$ and 1.5 per 50 pixels (top), and $2, 4$ and 6 per 50 pixels (bottom). Right: Ramp gradient per 50 pixels vs observed coverage, shown for $n = 100, 300$ and 500.

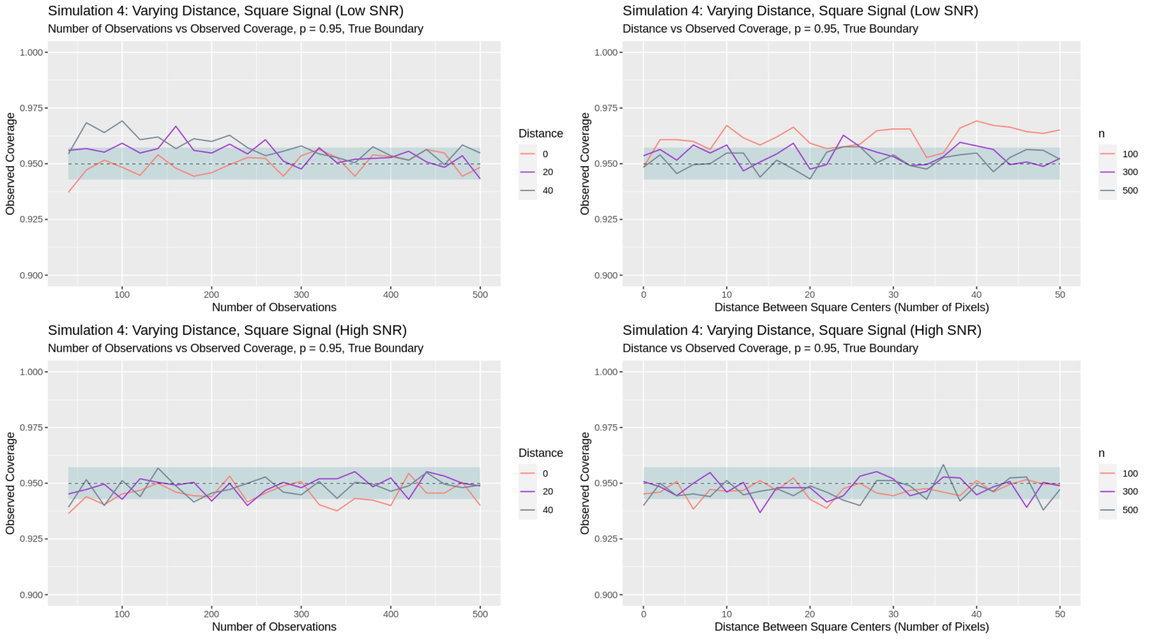

Figure S80: Empirical coverage for simulation 4. Top: Low-SNR synthetic data results. Bottom: High-SNR synthetic data results. Left: Number of observations vs observed coverage, shown for square separations of $0, 20$ and 40 pixels. Right: Separation between squares vs observed coverage, shown for $n = 100, 300$ and 500.

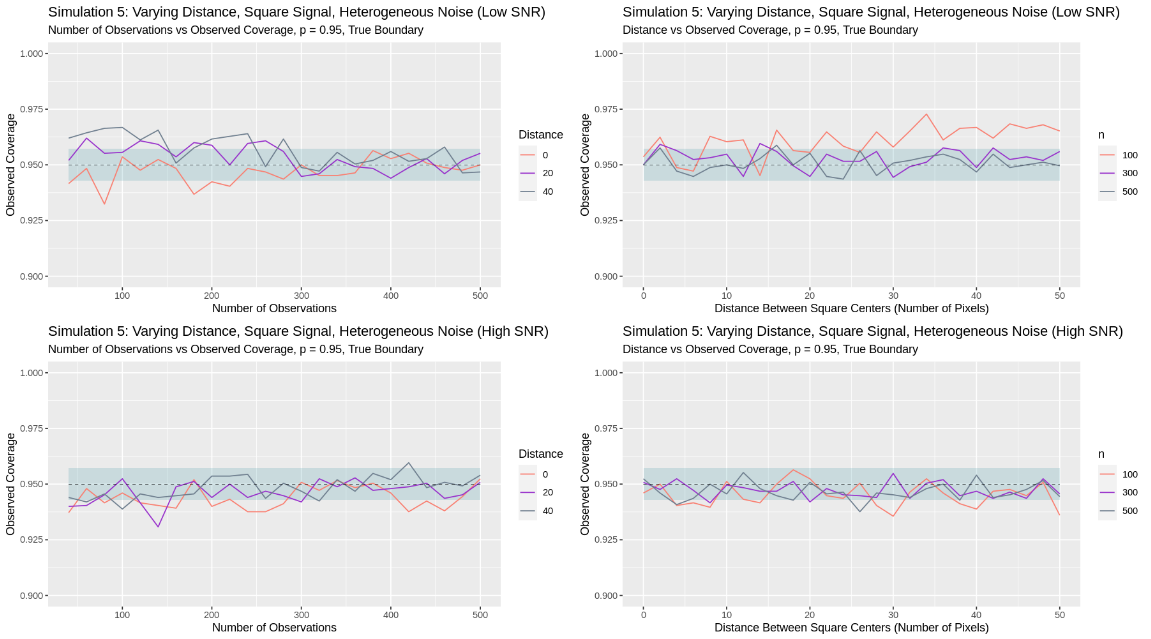

Figure S81: Empirical coverage for simulation 5. Top: Low-SNR synthetic data results. Bottom: High-SNR synthetic data results. Left: Number of observations vs observed coverage, shown for square separations of $0, 20$ and 40 pixels. Right: Separation between squares vs observed coverage, shown for $n = 100, 300$ and 500.

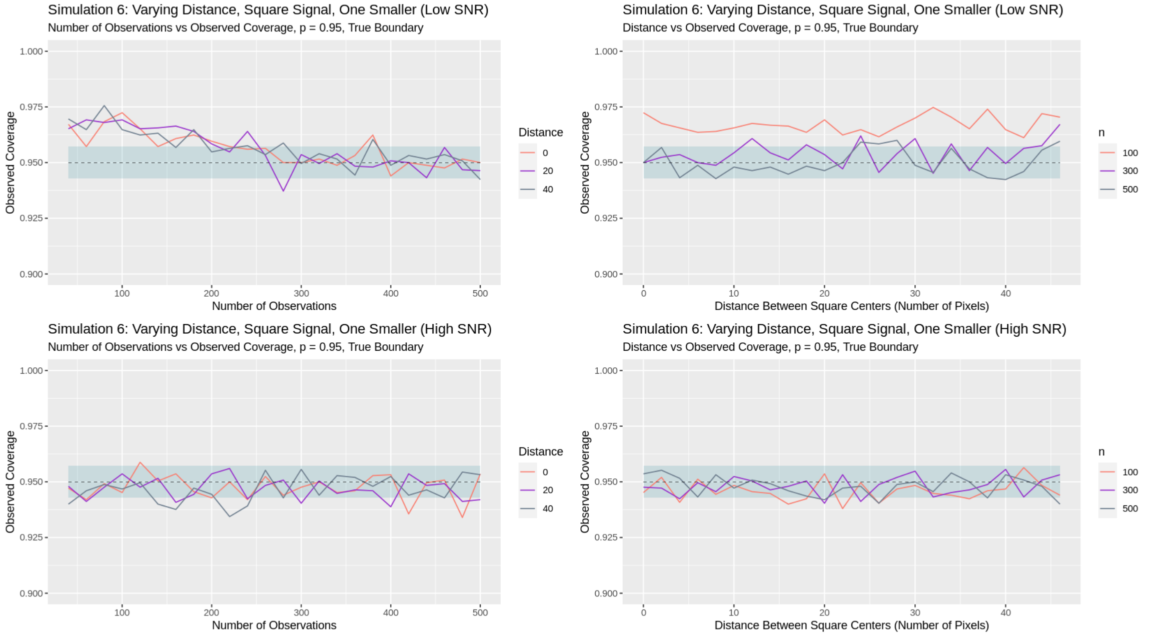

Figure S82: Empirical coverage for simulation 6. Top: Low-SNR synthetic data results. Bottom: High-SNR synthetic data results. Left: Number of observations vs observed coverage, shown for square separations of $0, 20$ and 40 pixels. Right: Separation between squares vs observed coverage, shown for $n = 100, 300$ and 500.

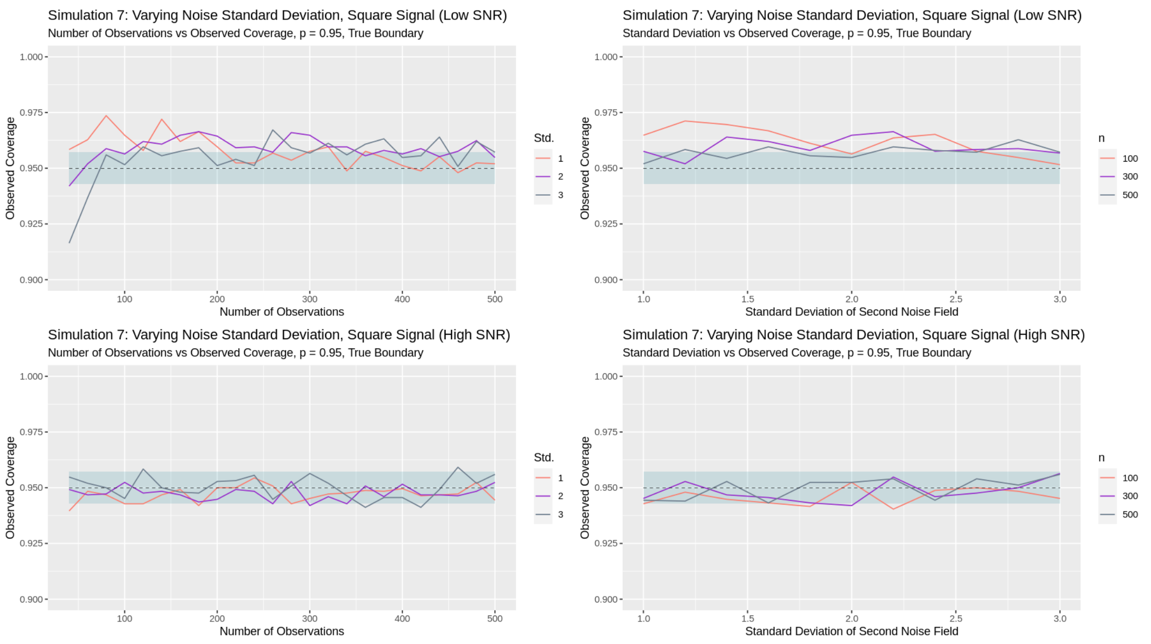

Figure S83: Empirical coverage for simulation 7. Top: Low-SNR synthetic data results. Bottom: High-SNR synthetic data results. Left: Number of observations vs observed coverage, shown for noise standard deviations of $1, 2$ and 3. Right: The standard deviation of the second noise field vs observed coverage, shown for $n = 100, 300$ and 500.

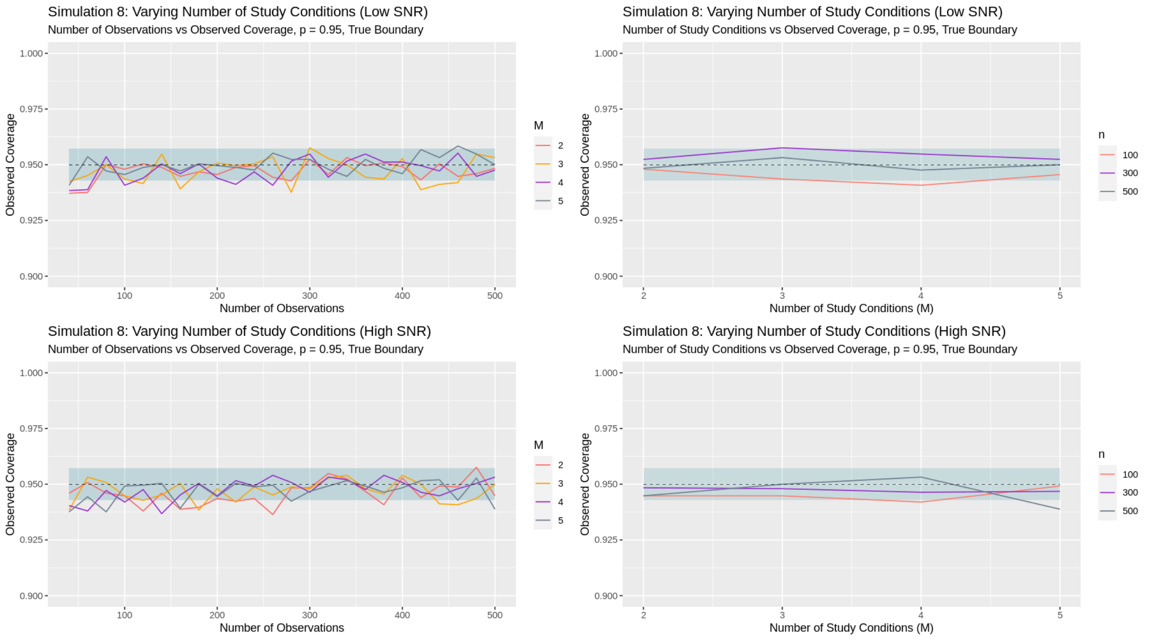

Figure S84: Empirical coverage for simulation 8. Top: Low-SNR synthetic data results. Bottom: High-SNR synthetic data results. Left: Number of observations vs observed coverage, shown for $M = 2, 3, 4$ and 5. Right: Number of study conditions (M) vs observed coverage, shown for $n = 100, 300$ and 500.

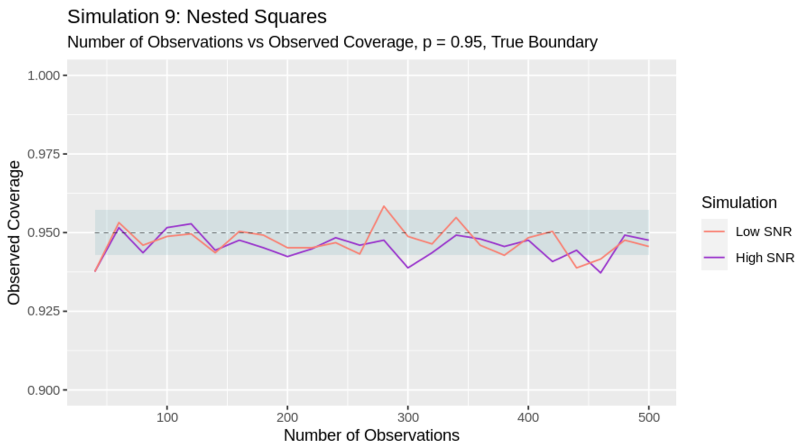

Figure S85: Empirical coverage for simulation 9. Results are shown for both low-SNR synthetic data and high-SNR synthetic data. Displayed is the number of observations vs observed coverage.

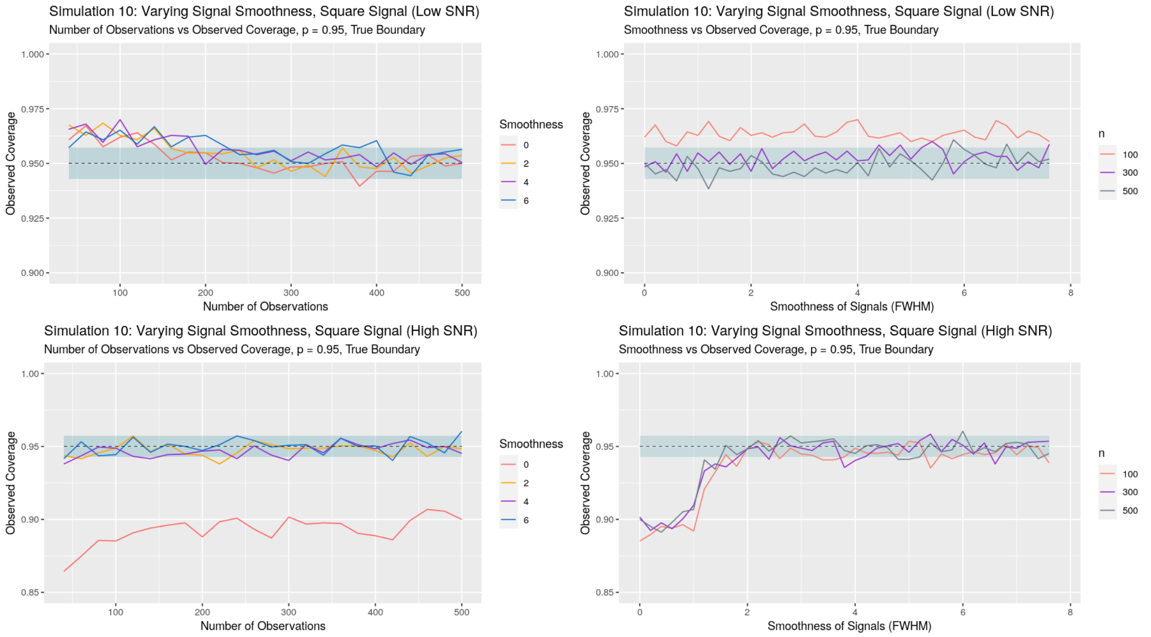

Figure S86: Empirical coverage for simulation 10. Top: Low-SNR synthetic data results. Bottom: High-SNR synthetic data results. Left: Number of observations vs observed coverage, shown for signal smoothness of $0, 2, 4$ and 6 FWHM. Right: Signal smoothness vs observed coverage, shown for $n = 100, 300$ and 500.

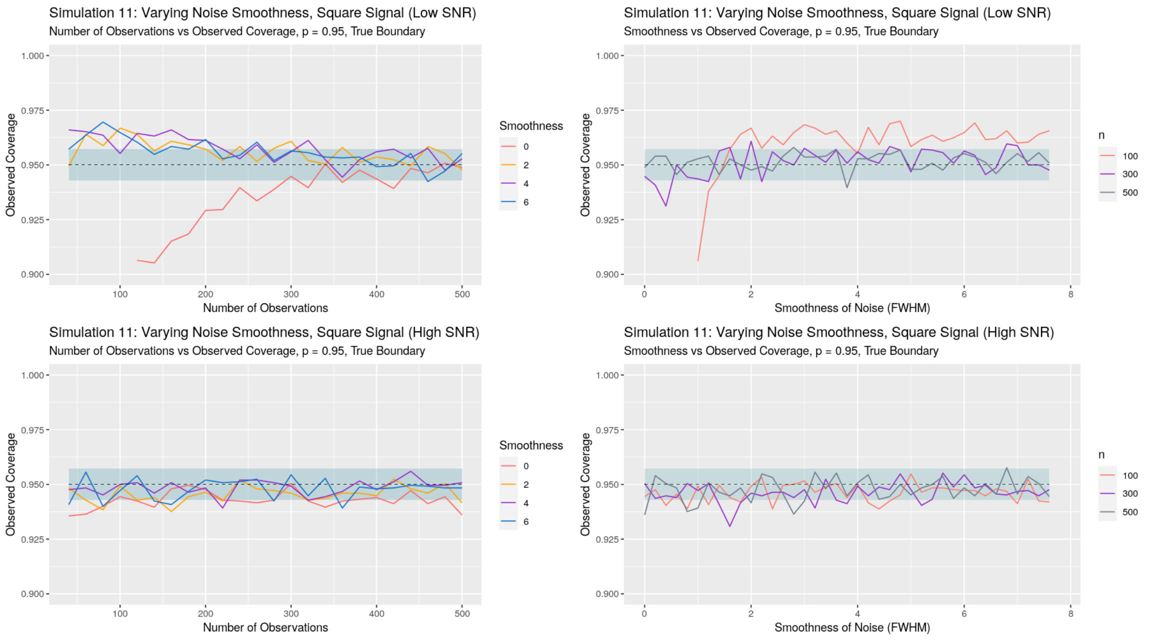

Figure S87: Empirical coverage for simulation 11. Top: Low-SNR synthetic data results. Bottom: High-SNR synthetic data results. Left: Number of observations vs observed coverage, shown for noise smoothness of $0, 2, 4$ and 6 FWHM. Right: Noise smoothness vs observed coverage, shown for $n = 100, 300$ and 500.

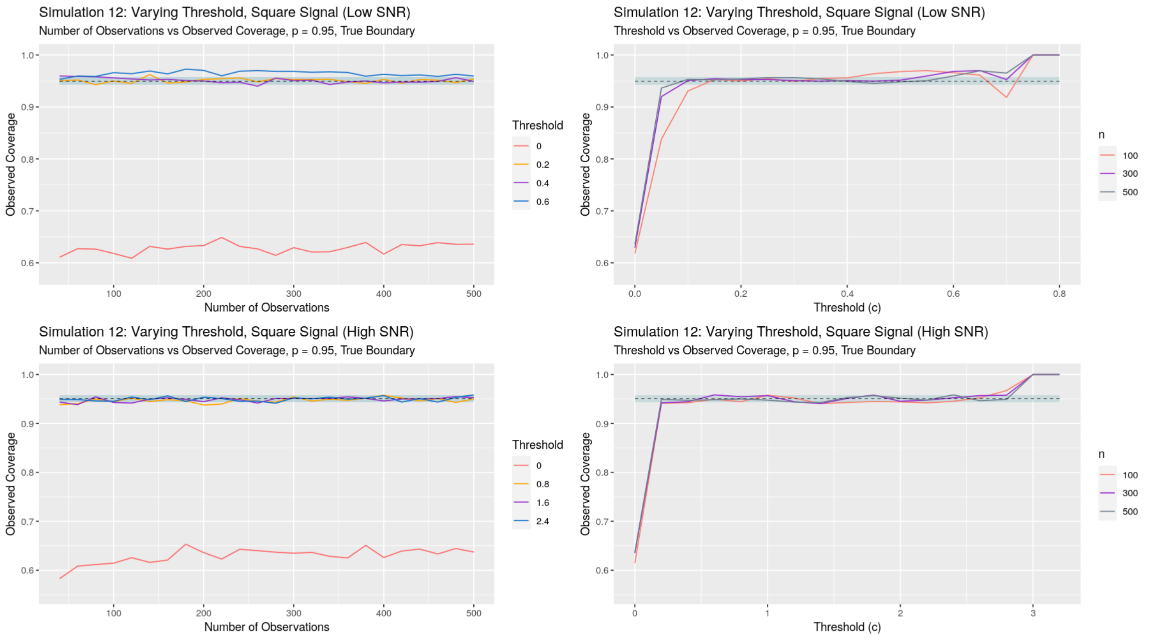

Figure S88: Empirical coverage for simulation 12. Top: Low-SNR synthetic data results. Bottom: High-SNR synthetic data results. Left: Number of observations vs observed coverage, shown for thresholds $c = 0, 0.2, 0.4$ and 0.6 (top) and $c = 0, 0.8, 1.6$ and 2.4 (bottom). Right: Threshold vs observed coverage, shown for $n = 100, 300$ and 500.

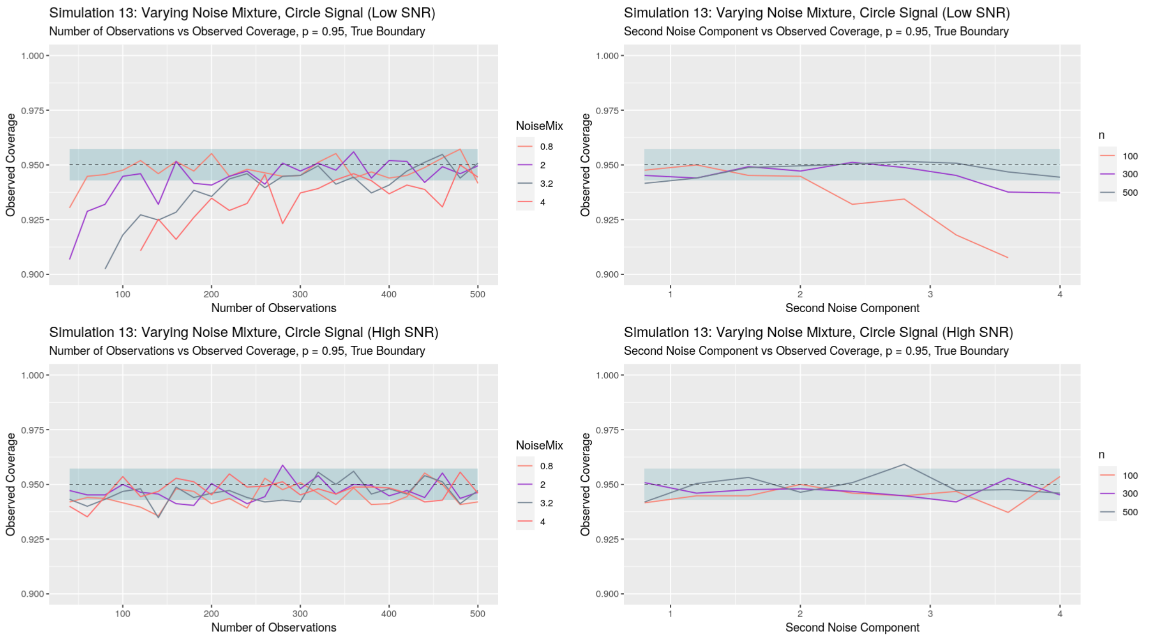

Figure S89: Empirical coverage for simulation 13. Top: Low-SNR synthetic data results. Bottom: High-SNR synthetic data results. Left: Number of observations vs observed coverage, shown for even Gaussian noise mixtures of $N(0,1)$ and $N(0,\sigma^2)$ with $\sigma = 0.8, 2, 3.2$ and 4. Right: Second noise component (σ) vs observed coverage, shown for $n = 100, 300$ and 500.

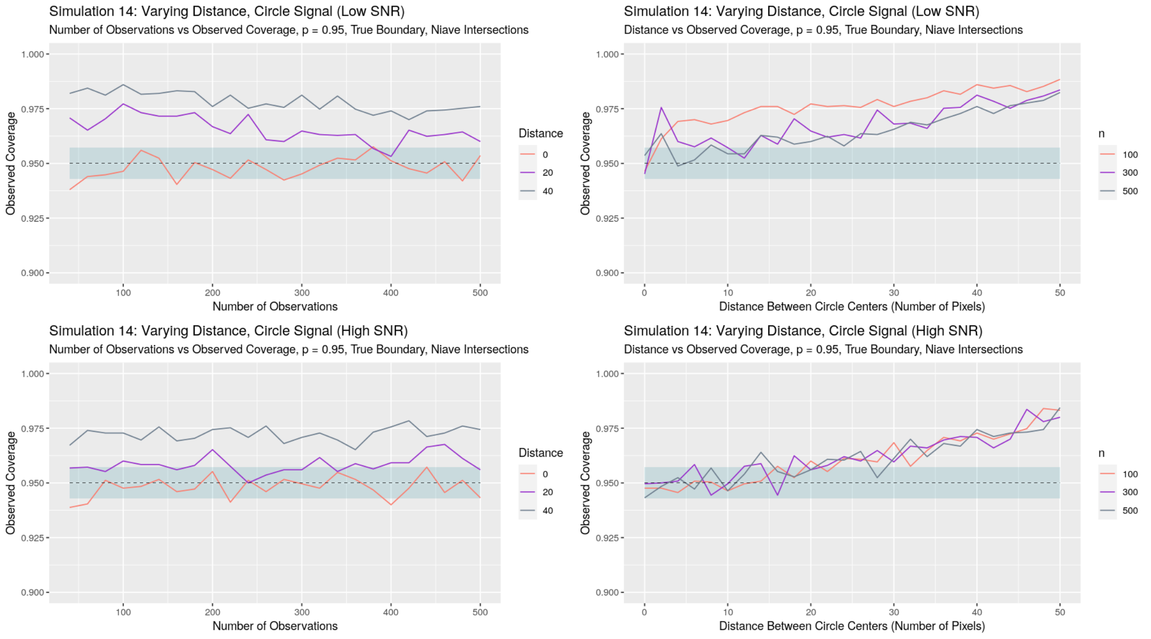

Figure S90: Empirical coverage for simulation 14. Top: Low-SNR synthetic data results. Bottom: High-SNR synthetic data results. Left: Number of observations vs observed coverage, shown for circle separations of 0, 20 and 40 pixels. Right: Separation between circles vs observed coverage, shown for $n = 100, 300$ and 500.

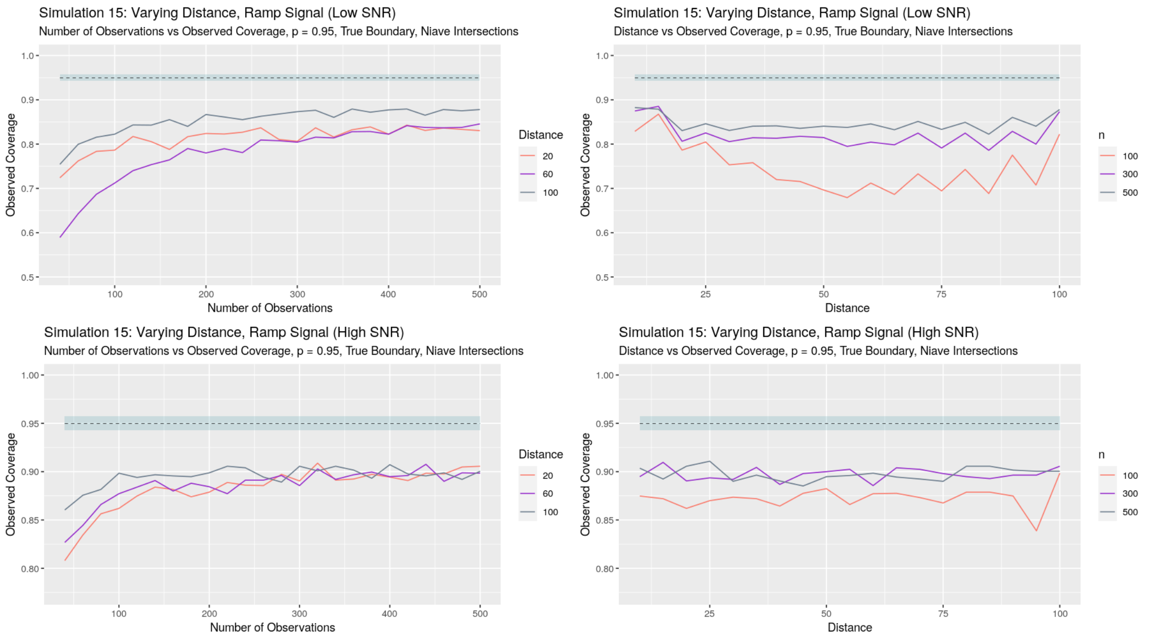

Figure S91: Empirical coverage for simulation 15. Top: Low-SNR synthetic data results. Bottom: High-SNR synthetic data results. Left: Number of observations vs observed coverage, shown for ramp separations of 20, 60 and 100 pixels. Right: Separation between ramps vs observed coverage, shown for $n = 100, 300$ and 500.

S7 Computation Time

In this section, we present the observed computation times for the 15 simulations described in Section S4 of this document and Section 4.1 of the main text. All results reported in this section were obtained using an Intel(R) Xeon(R) Gold 6126 2.60GHz processor with 16GB RAM and are given as averages taken over 5000 simulation instances. 2500 of the simulation instances used the estimated boundary, $\partial\hat{\mathcal{F}}_c$, to evaluate the suprema in Equation (S1), whilst the other 2500 used the true boundary, $\partial\mathcal{F}_c$. Each simulation instance was performed using 5000 bootstrap realisations. We stress here that the implementation of CRs provided alongside this work is far from optimal. The purpose of the inclusion of the following results is to highlight trends in computation time rather than to showcase our implementation of the method.

Across all simulations, it can be seen that computation time appeared to increase linearly with the number of observations. In addition, linear trends in computation time were observed as the overlap between excursion sets increased (c.f. Figs. S92 and S95-S97), the signal gradient was reduced (Fig. S94) and the noise standard deviation was increased (Fig. S98). Notably, varying the correlation between the noise fields, and changing the noise and signal smoothness, appeared to have little impact on the observed computation time (c.f. Figs. S93, S101 and S102). However, an exponential increase in computation time was observed when the number of study conditions, M, was increased (c.f. Fig. S99). This exponential increase in computation time is to be expected as the size of the power set, $\mathcal{P}^+(\mathcal{M})$, (and thus the number of boundary segments which must be considered during the bootstrap) increases exponentially with M.

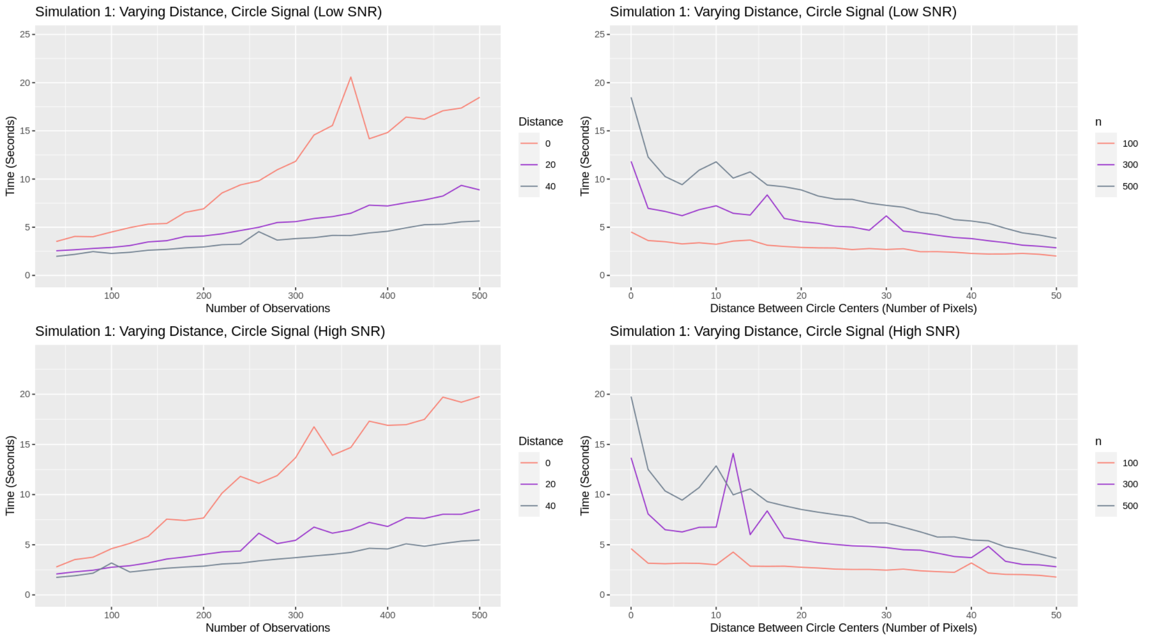

Figure S92: Computation times for Simulation 1. Top: Low-SNR synthetic data results. Bottom: High-SNR synthetic data results. Left: Number of observations vs computation time, shown for circle separations of $0, 20$ and 40 pixels. Right: Separation between circles vs computation time, shown for $n = 100, 300$ and 500.

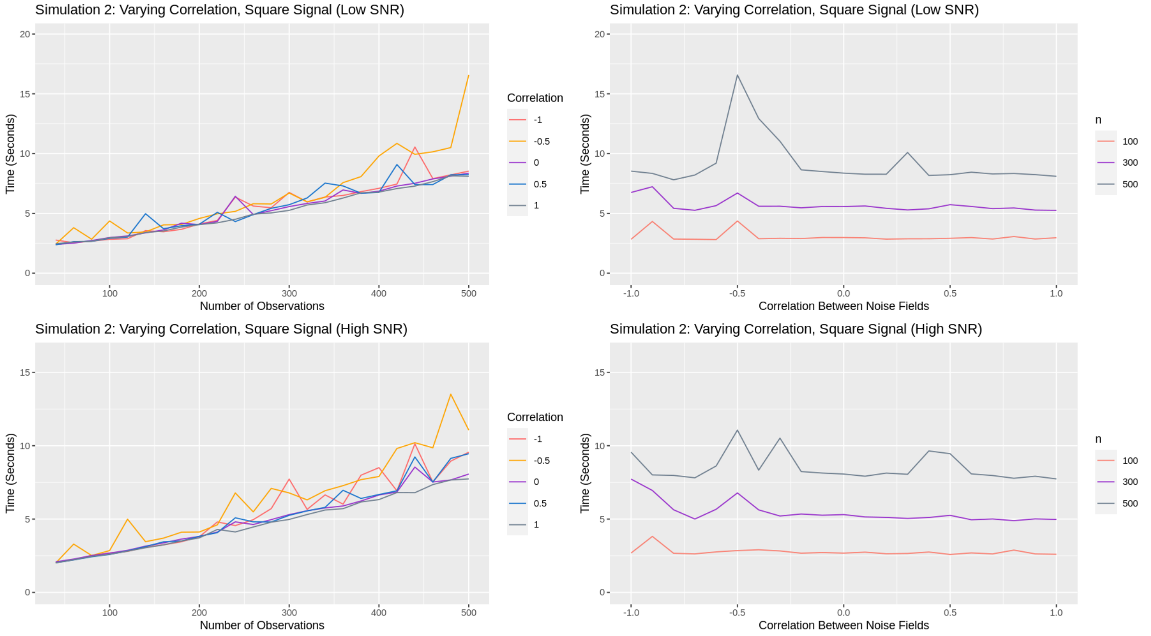

Figure S93: Computation times for Simulation 2. Top: Low-SNR synthetic data results. Bottom: High-SNR synthetic data results. Left: Number of observations vs computation time, shown for correlations of $-1, -0.5, 0, 0.5$ and 1. Right: Correlation between noise fields vs computation time, shown for $n = 100, 300$ and 500.

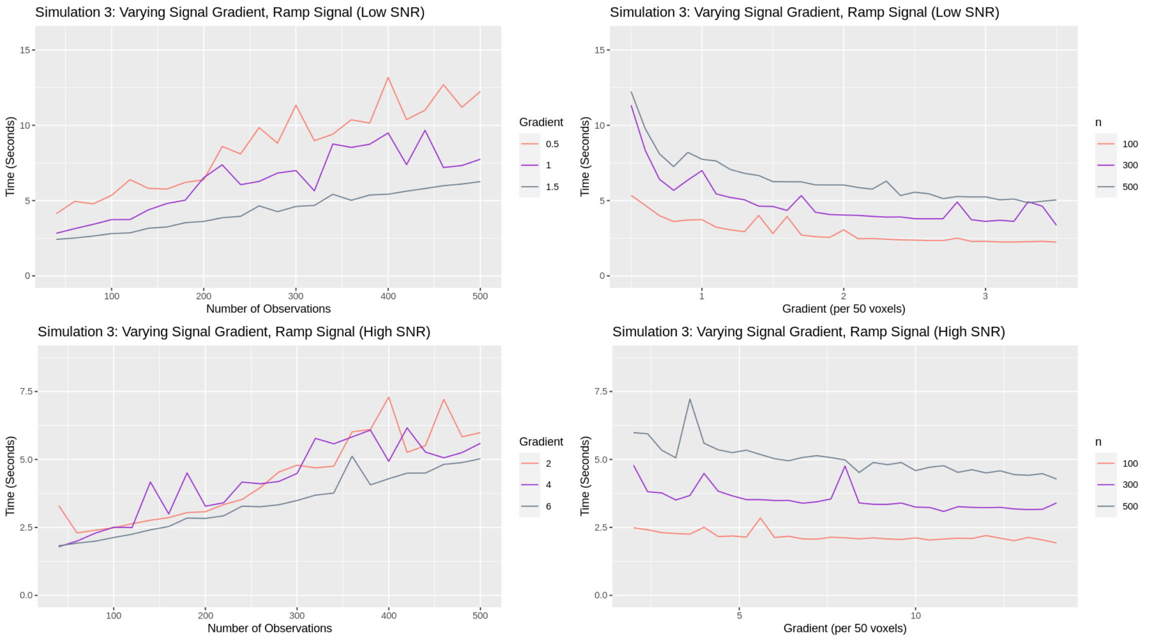

Figure S94: Computation times for Simulation 3. Top: Low-SNR synthetic data results. Bottom: High-SNR synthetic data results. Left: Number of observations vs computation time, shown for gradients of $0.5, 1$ and 1.5 per 50 pixels (top), and $2, 4$ and 6 per 50 pixels (bottom). Right: Ramp gradient per 50 pixels vs computation time, shown for $n = 100, 300$ and 500.

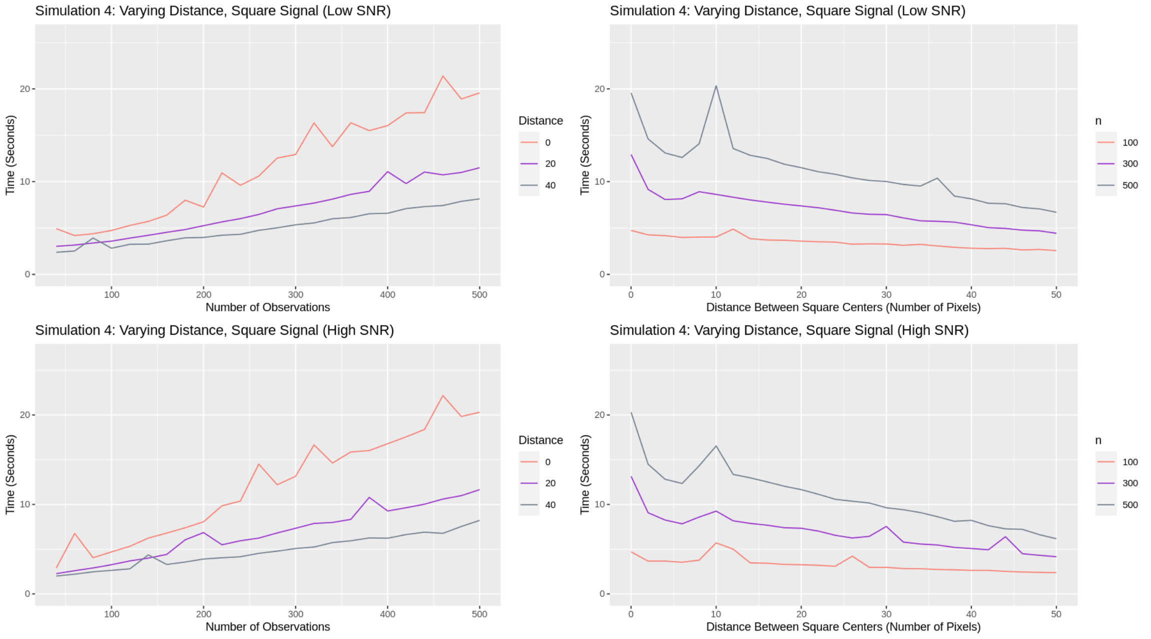

Figure S95: Computation times for Simulation 4. Top: Low-SNR synthetic data results. Bottom: High-SNR synthetic data results. Left: Number of observations vs computation time, shown for square separations of $0, 20$ and 40 pixels. Right: Separation between squares vs computation time, shown for $n = 100, 300$ and 500.

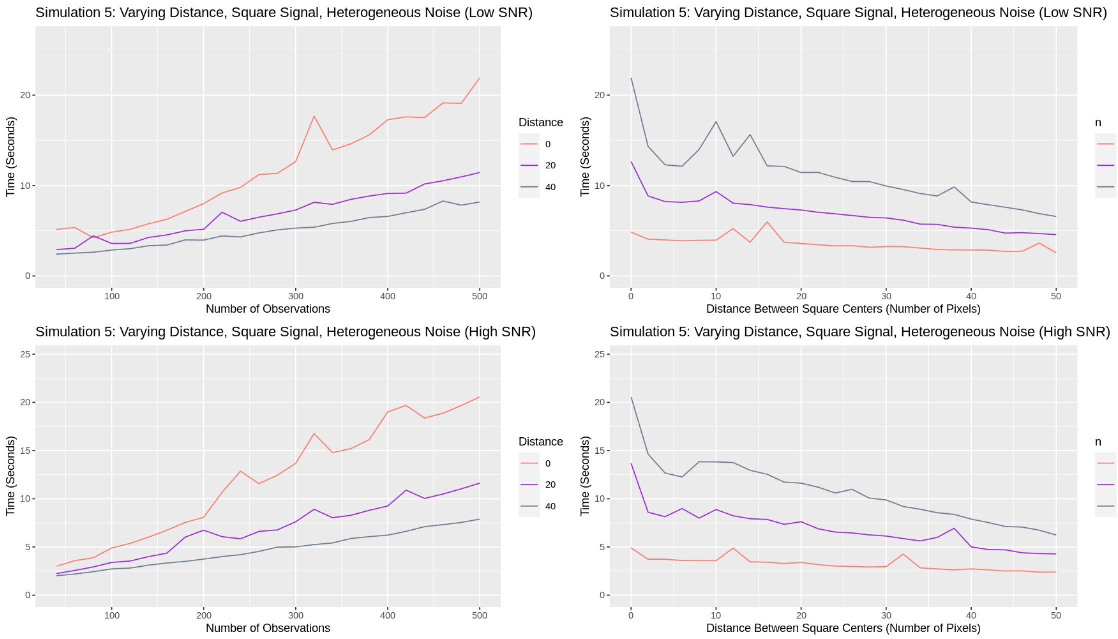

Figure S96: Computation times for Simulation 5. Top: Low-SNR synthetic data results. Bottom: High-SNR synthetic data results. Left: Number of observations vs computation time, shown for square separations of $0, 20$ and 40 pixels. Right: Separation between squares vs computation time, shown for $n = 100, 300$ and 500.

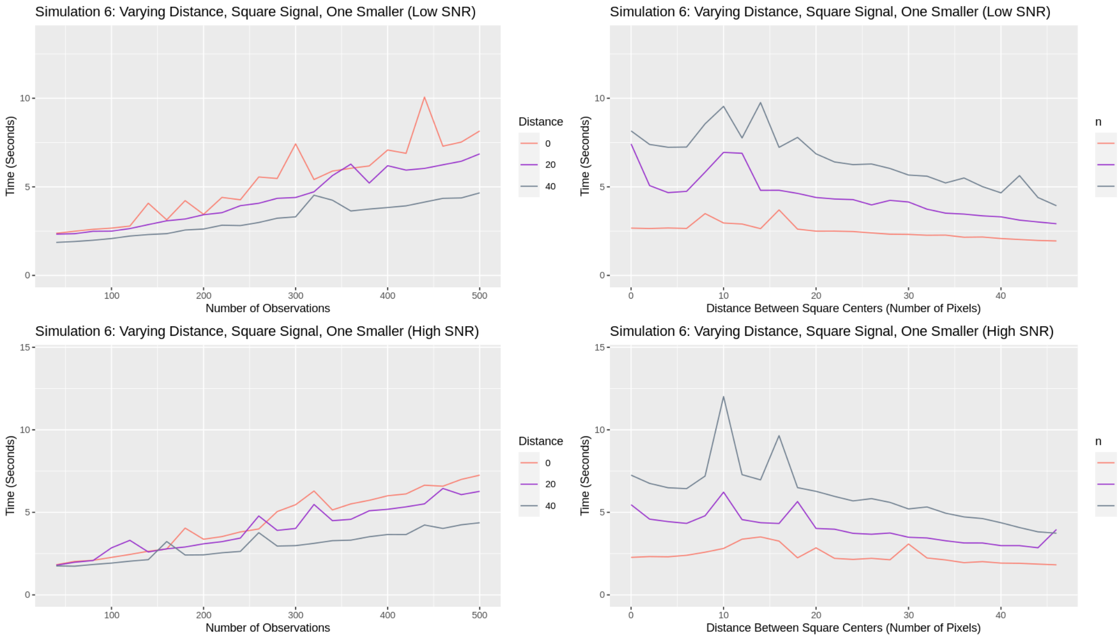

Figure S97: Computation times for Simulation 6. Top: Low-SNR synthetic data results. Bottom: High-SNR synthetic data results. Left: Number of observations vs computation time, shown for square separations of $0, 20$ and 40 pixels. Right: Separation between squares vs computation time, shown for $n = 100, 300$ and 500.

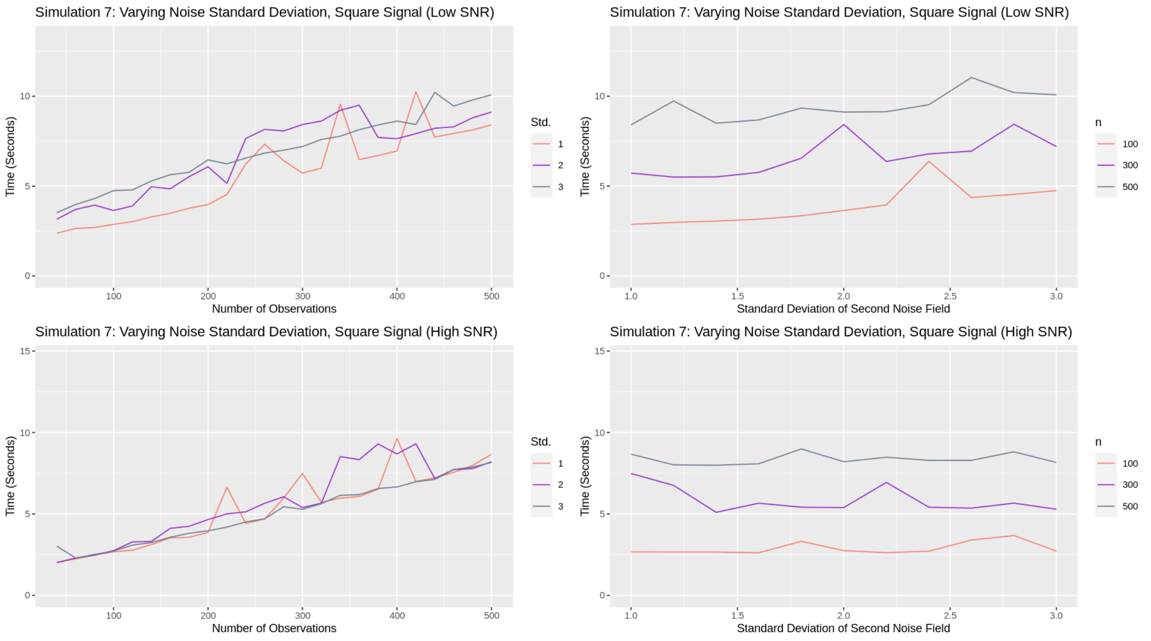

Figure S98: Computation times for Simulation 7. Top: Low-SNR synthetic data results. Bottom: High-SNR synthetic data results. Left: Number of observations vs computation time, shown for noise standard deviations of $1, 2$ and 3. Right: The standard deviation of the second noise field vs computation time, shown for $n = 100, 300$ and 500.

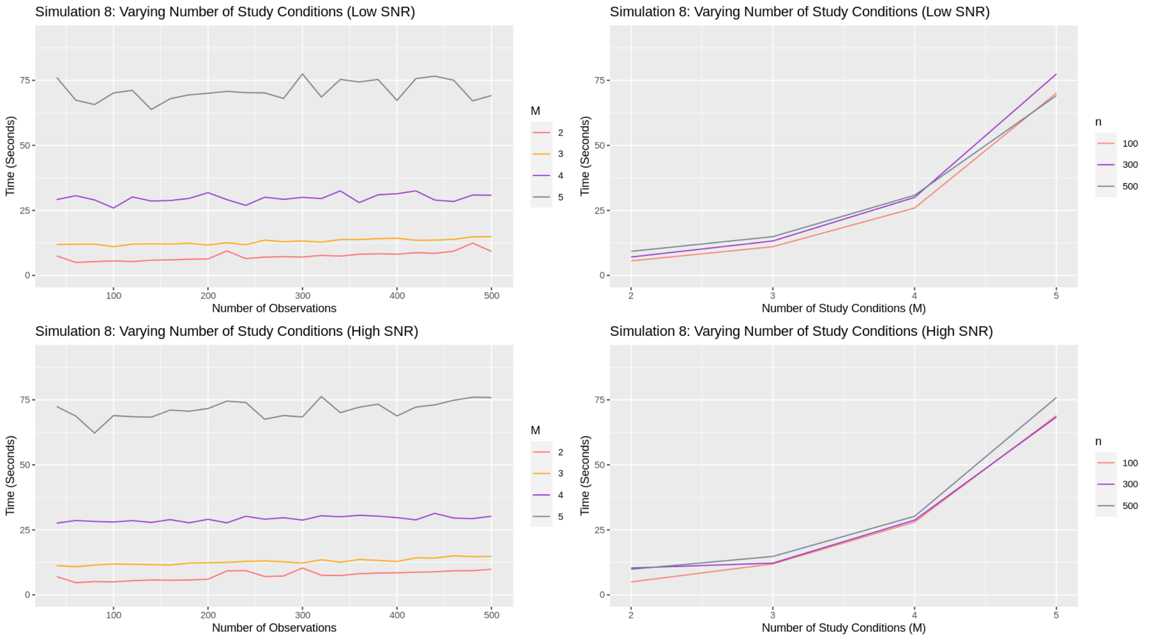

Figure S99: Computation times for Simulation 8. Top: Low-SNR synthetic data results. Bottom: High-SNR synthetic data results. Left: Number of observations vs computation time, shown for $M = 2, 3, 4$ and 5. Right: Number of study conditions (M) vs computation time, shown for $n = 100, 300$ and 500.

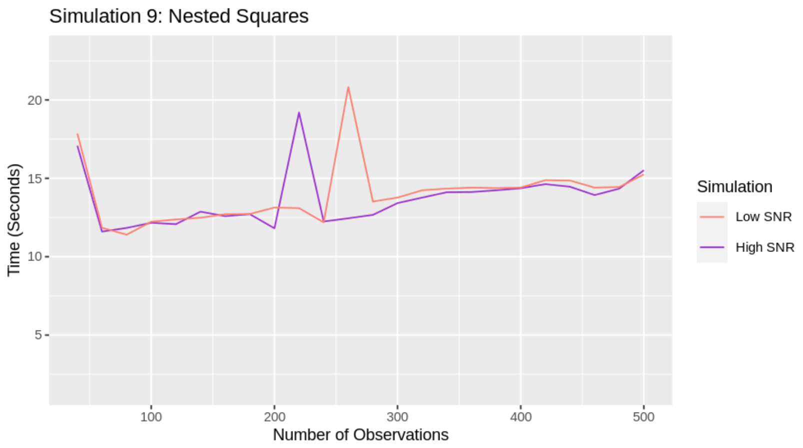

Figure S100: Computation times for Simulation 9. Results are shown for both low-SNR synthetic data and high-SNR synthetic data. Displayed is the number of observations vs computation time.

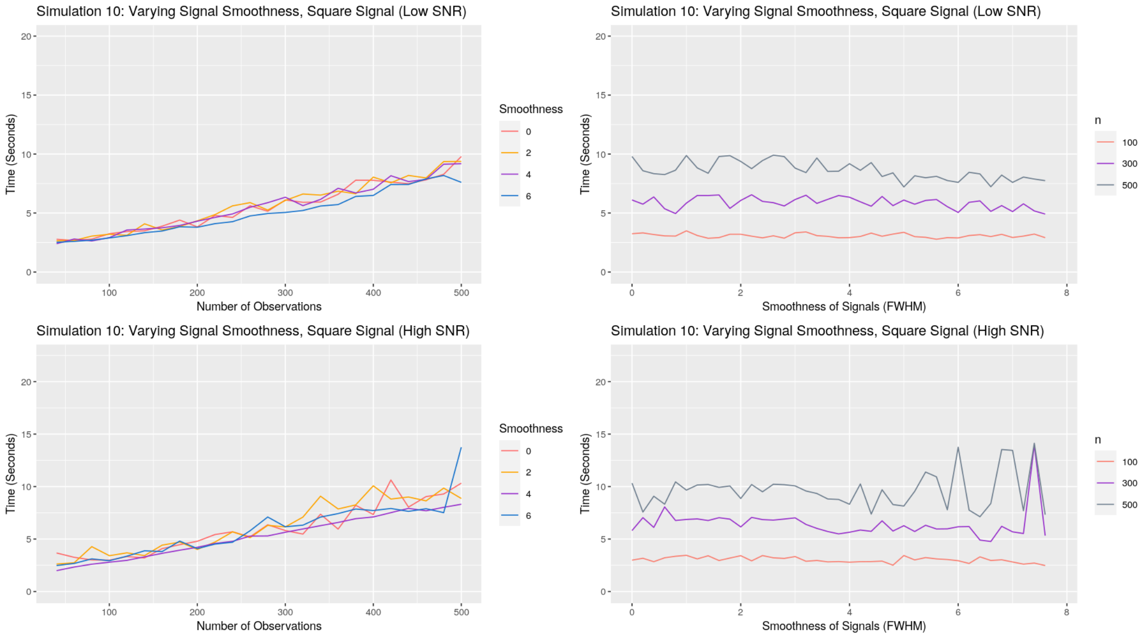

Figure S101: Computation times for simulation 10. Top: Low-SNR synthetic data results. Bottom: High-SNR synthetic data results. Left: Number of observations vs computation time, shown for signal smoothness of $0, 2, 4$ and 6 FWHM. Right: Signal smoothness vs computation time, shown for $n = 100, 300$ and 500.

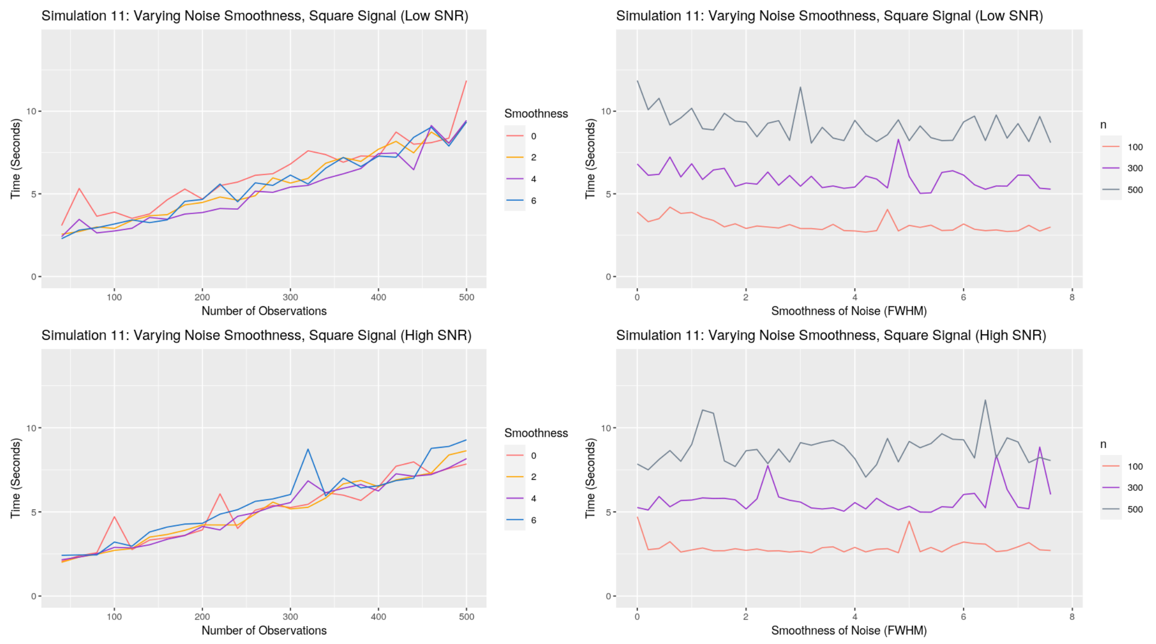

Figure S102: Computation times for simulation 11. Top: Low-SNR synthetic data results. Bottom: High-SNR synthetic data results. Left: Number of observations vs computation time, shown for noise smoothness of $0, 2, 4$ and 6 FWHM. Right: Noise smoothness vs computation time, shown for $n = 100, 300$ and 500.

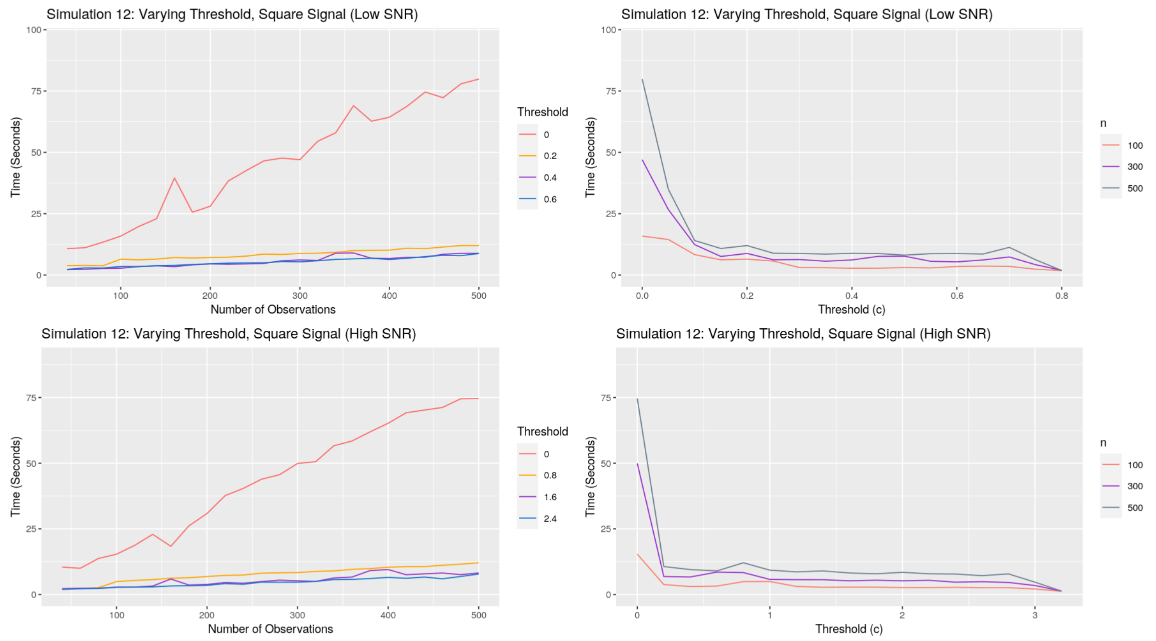

Figure S103: Computation times for simulation 12. Top: Low-SNR synthetic data results. Bottom: High-SNR synthetic data results. Left: Number of observations vs computation time, shown for thresholds $c = 0, 0.2, 0.4$ and 0.6 (top) and $c = 0, 0.8, 1.6$ and 2.4 (bottom). Right: Threshold vs computation time, shown for $n = 100, 300$ and 500.

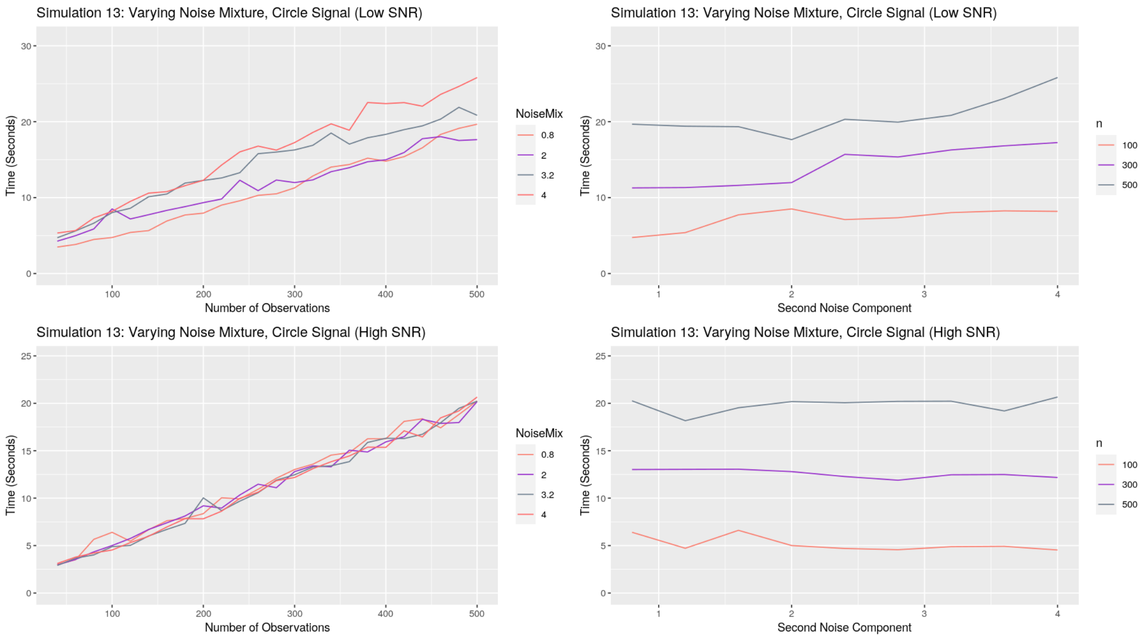

Figure S104: Computation time for simulation 13. Top: Low-SNR synthetic data results. Bottom: High-SNR synthetic data results. Left: Number of observations vs computation time, shown for even Gaussian noise mixtures of $N(0,1)$ and $N(0,\sigma^2)$ with $\sigma = 0.8, 2, 3.2$ and 4. Right: Second noise component (σ) vs computation time, shown for $n = 100, 300$ and 500.

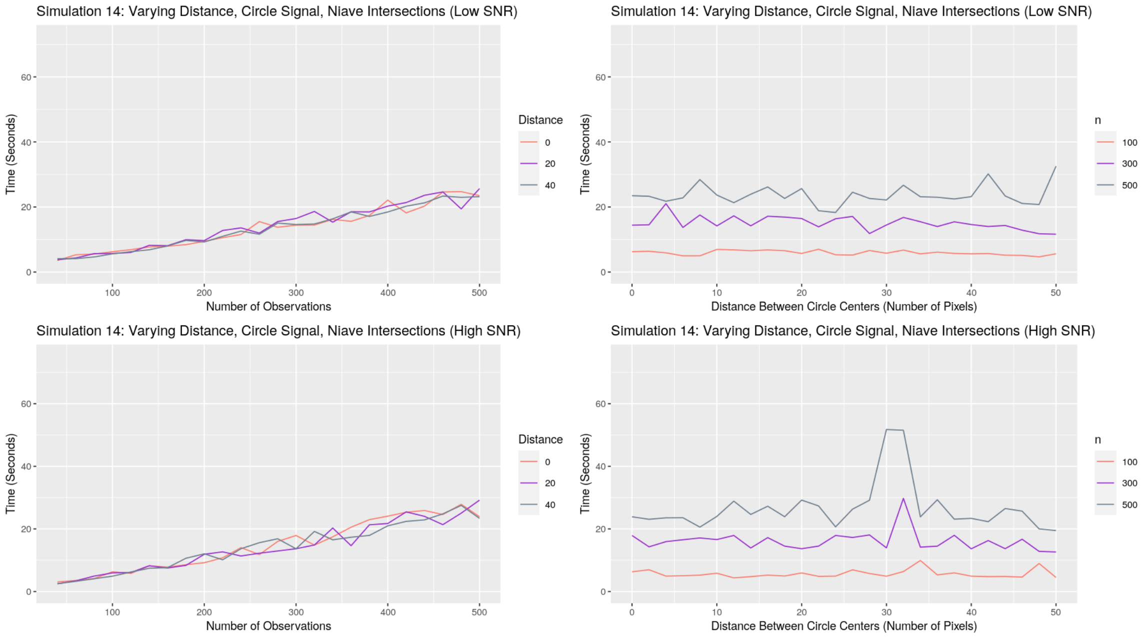

Figure S105: Computation time for simulation 14. Top: Low-SNR synthetic data results. Bottom: High-SNR synthetic data results. Left: Number of observations vs computation time, shown for circle separations of 0, 20 and 40 pixels. Right: Separation between circles vs computation time, shown for $n = 100, 300$ and 500.

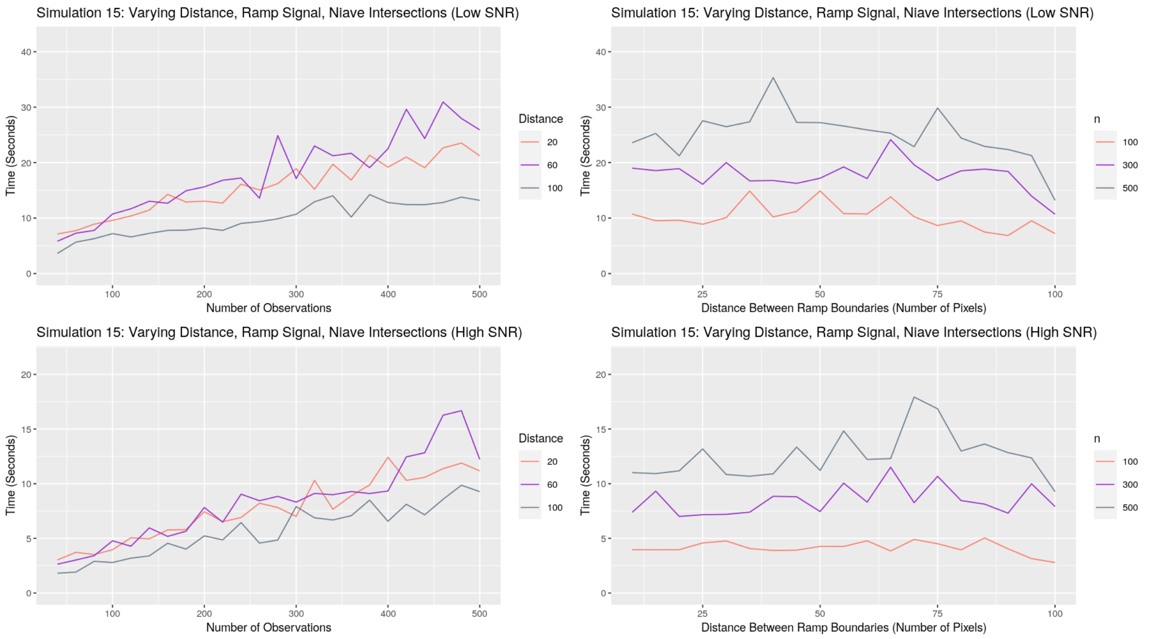

Figure S106: Computation time for simulation 15. Top: Low-SNR synthetic data results. Bottom: High-SNR synthetic data results. Left: Number of observations vs computation time, shown for ramp separations of $20, 60$ and 100 pixels. Right: Separation between ramps vs computation time, shown for $n = 100, 300$ and 500.

References

Alexander Bowring, Fabian Telschow, Armin Schwartzman, and Thomas E. Nichols. Spatial confidence sets for raw effect size images. *NeuroImage*, 203:116187, 2019. ISSN 1053-8119. doi: https://doi.org/10.1016/j.neuroimage.2019.116187. URL `https://www.sciencedirect.com/science/article/pii/S1053811919307785`.

Mark W. Woolrich, Brian D. Ripley, Michael Brady, and Stephen M. Smith. Temporal autocorrelation in univariate linear modeling of fmri data. *NeuroImage*, 14(6):1370–1386, 2001. ISSN 1053-8119. doi: https://doi.org/10.1006/nimg.2001.0931.

Max Sommerfeld, Stephan Sain, and Armin Schwartzman. Confidence regions for spatial excursion sets from repeated random field observations, with an application to climate. *Journal of the American Statistical Association*, 113(523):1327–1340, 2018. doi: 10.1080/01621459.2017.1341838. URL `https://doi.org/10.1080/01621459.2017.1341838`. PMID: 31452557.
