# Supplementary material for: Spatial confidence regions for combinations of excursion sets in image analysis
Source: J R Stat Soc Series B Stat Methodol. 2023 Sep 21;86(1):177–93. doi: 10.1093/jrsssb/qkad104 (PMC10852994; doi:10.1093/jrsssb/qkad104)
Supplement: qkad104_Supplementary_Data [file qkad104_supplementary_data.zip › Supplementary_Theory.pdf]

Spatial Confidence Regions for Combinations of Excursion Sets in Image Analysis

Supplementary Theory

Thomas Maullin-Sapey[1,*], Armin Schwartzman[2,3], and Thomas E. Nichols[1]

[1] Big Data Institute, Li Ka Shing Centre for Health Information and Discovery, Oxford, UK

[2] Division of Biostatistics, University of California, San Diego, CA, USA

[3] Halicioğlu Data Science Institute, University of California, San Diego, USA

[*]Corresponding Author: Thomas, TM, Maullin-Sapey, Thomas.Maullin-Sapey@bdi.ox.ac.uk

Submission for Journal of the Royal Statistical Society Series B (Statistical Methodology)

S1 Overview

This document contains supplementary material concerning the theory presented in the paper 'Spatial Confidence Regions for Combinations of Excursion Sets in Image Analysis'. Provided in the following sections is an in-depth overview, alongside proof and discussion, of the central theory stated in Section 2.3 of the main text. For reference, this result is restated below:

Theorem S1. *Under the assumptions of Section 2.2, the below holds:*

$$\lim_{n\to\infty} \mathbb{P}[\hat{\mathcal{F}}_c^+ \subseteq \mathcal{F}_c \subseteq \hat{\mathcal{F}}_c^-] = \mathbb{P}\left[\max_{\phi\in\mathcal{P}^+(\mathcal{M})}\left(\sup_{s\in\partial^\phi\mathcal{F}_c}\left|\min_{i\in\phi}(G^i(s))\right|\right) \leq a\right],$$

where the definitions for $\mathcal{M}, \mathcal{P}^+(\mathcal{M}), \mathcal{F}_c, \hat{\mathcal{F}}_c^+, \hat{\mathcal{F}}_c^-, \{\partial^\phi\mathcal{F}_c\}_{\phi\in\mathcal{P}^+(\mathcal{M})}$ *and* $\{G^i\}_{i\in\mathcal{M}}$ *may be found in Sections 1-2.2 of the main text.*

This document is organized as follows. Section S2 provides a detailed description of the differences between the three statements provided in Assumption 2.2.3. Next, Section S3 describes how Theorem S1 may be employed to perform disjunction, instead of conjunction, inference. Following this, Section S4 provides a full proof of Theorem S1. The proof of the theorem is separated into two halves; the first half is detailed in Section S4.1 and the second half is given in Section S4.2. Both halves of the proof require supporting lemmas, which are listed in Section S4.3, and supporting theorems, which are stated and proven in Sections S4.4-S4.11. In Section S5, we provide detail on how the assumptions of Section 2.2 of the main text may be employed in the linear modelling context of Section 3.1. Finally, in Section S6, we discuss why confidence regions for $\mathcal{F}_c$ cannot be obtained by simply intersecting the single-'study condition' confidence regions obtained using the methods of Sommerfeld et al. [2018].

S2 Discussion and Illustration of Assumption 2.2.3

Each of the three statements provided by Assumption 2.2.3 serves a separate purpose, handling different use cases that the others do not. In the following subsections, we explicitly discuss such cases for each assumption in turn.

S2.1 Statement (a): The Ball Assumption

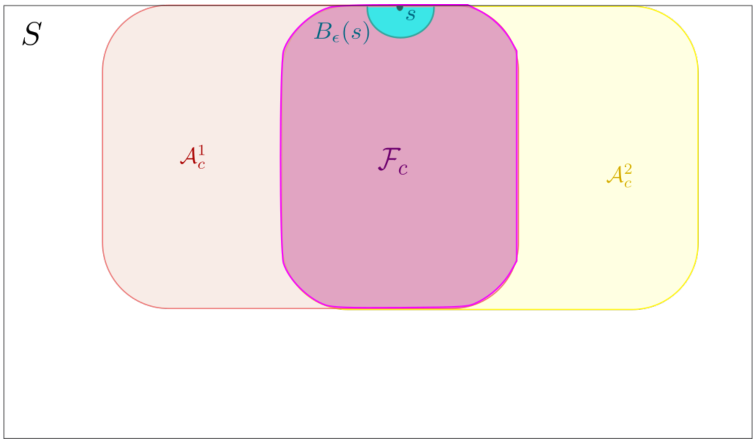

Figure S1: An example in which Assumption 2.2.3 Statements (b) and (c) are satisfied but the ball assumption is not. In this example, $\partial\mathcal{F}_c$ coincides perfectly with ∂S, and as such, at the point $s \in \partial^{\{1,2\}}\mathcal{F}_c$, it is possible to draw a ball, $B_\epsilon(s)$, which does not intersect $\mathcal{J}_c^{\{1,2\}}$.

Statement (a), which we shall refer to as 'the ball assumption', ensures that $\partial\mathcal{F}_c$ does not coincide exactly with ∂S by explicitly requiring well-defined regions to exist on either side of $\partial\mathcal{F}_c$. To ensure this criterion is met, the ball assumption defines the set $\mathcal{J}_c^\phi$ which, broadly speaking, may be thought of as the set which we would 'naturally expect' to lie on the outside of $\partial^\phi\mathcal{F}_c$. To understand the definition of $\mathcal{J}_c^\phi$, consider a brief example in which $M = 2$. In this setting, if $s \in \partial^{\{1\}}\mathcal{F}_c$, then $g^1(s) = 0$ and $g^2(s) > 0$. In a neighbourhood of s, we wish to assume that $g^1 > 0$ on one 'side' of $\partial\mathcal{F}_c$, and $g^1 < 0$ on the other. As $g^2(s) > 0$, it follows that $g^2 > 0$ everywhere in a small enough neighbourhood of s and, therefore, such a neighbourhood intersects $(\mathcal{F}_c)^\circ$ (where $g^1 > 0$ and $g^2 > 0$), and $\mathcal{J}_c^{\{1\}}$ (where $g^1 < 0$ and $g^2 > 0$). These are the sets stated by the ball assumption.

The ball assumption is necessary to the proofs of Section S4 as we shall often need to define sequences of points, which lie either inside or outside $\mathcal{F}_c$, that tend to $\partial\mathcal{F}_c$. We note that the ball assumption is naturally satisfied in many practical applications which involve set intersections (or unions) (c.f. Fig. 3 of the main text) and rarely impacts the applicability of our results. Similar assumptions may also be found in precursors to this work (e.g. Assumption 2.1 (a) of Sommerfeld et al. [2018]). There are some use cases that the ball assumption removes from consideration which the other statements provided by Assumption 2.2.3 do not. These are the cases in which $\partial\mathcal{F}_c$ perfectly aligns with ∂S, (see Fig. S1). We emphasize that this assumption does not forbid $\partial\mathcal{F}_c$ from touching ∂S in general, but instead restricts the degree to which the two sets may coincide.

S2.2 Statement (b): The Gradient Assumption

Statement (b), which we shall refer to as 'the gradient assumption', ensures that, in a neighbourhood of $\partial\mathcal{F}_c$, the functions $\{g^i\}_{i\in\mathcal{M}}$ are C^1. By applying implicit function theorem to the functions $\{g^i\}_{i\in\mathcal{M}}$, it can be seen that this statement ensures that $\{\partial\mathcal{A}_c^i\}_{i\in\mathcal{M}}$ are well-defined C^1 curves. As it is also required that, along the boundary $\partial\mathcal{F}_c$, the gradients are non-zero and finite, this assumption also ensures that the functions $\{g^i\}_{i\in\mathcal{M}}$ neither flatten out nor become directly vertical, in a neighbourhood of $\partial\mathcal{F}_c$. This observation is particularly relevant in the proofs of Section S4 when we consider spatial regions, which are defined using the functions $\{g^i\}_{i\in\mathcal{M}}$, that 'shrink' to the boundary segments $\{\partial^\phi\mathcal{F}_c\}_{\phi\in\mathcal{P}^+(\mathcal{M})}$. Without bounding the rate of change of the functions $\{g^i\}_{i\in\mathcal{M}}$ in this manner, the steps in the proof which involve such 'shrinking' spatial regions would not be possible.

S2.3 Statement (c): The Path-Connectedness Assumption

Statement (c), which we shall refer to as 'the path-connectedness assumption', ensures that near the boundary of $\mathcal{F}_c$, the boundary sets $\{\partial(\cap_{i\in\phi}\mathcal{A}_c^i)\}_{\phi\in\mathcal{P}^+(\mathcal{M})}$ are not self-intersecting and do not exhibit fractal-like behaviour. Most notably, as $\partial\mathcal{F}_c$ is a set of the form $\partial(\cap_{i\in\phi}\mathcal{A}_c^i)$ (with $\phi=\mathcal{M}$), it follows that this assumption prevents $\partial\mathcal{F}_c$ from crossing itself or exhibiting fractal-like properties.

Assumption 2.2.3 Statement (c) is necessary as it requires 'path-connectedness'. This

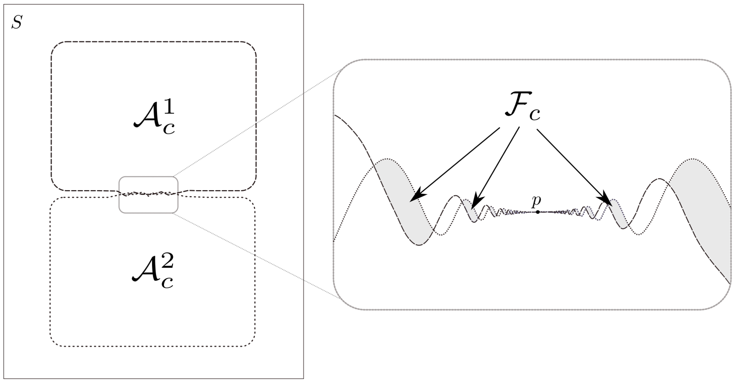

Figure S2: An example in which $N = 2$ and Assumption 2.2.3 Statements (a) and (b) are satisfied but the path-connectedness assumption is not. In the neighbourhood of p shown on the right, $\partial\mathcal{A}_c^1$ and $\partial\mathcal{A}_c^2$ resemble the functions $y = x^2 \sin(\frac{\pi}{2} - \frac{\pi}{x})$ and $y = x^2 \sin(\frac{\pi}{x})$ respectively (Note that both of these functions are C^1 and therefore consistent with Assumption 2.2.3 Statement (b)). However, the path-connectedness assumption does not hold at the point p and, as a result, $(\mathcal{F}_c)^\circ$ possesses the undesirable property of being a fractal.

requirement ensures that, for arbitrary $p \in \partial\mathcal{F}_c$, all boundaries of the form $\partial(\cap_{i\in\phi}\mathcal{A}_c^i)$ which contain p (including $\partial\mathcal{F}_c$) partition a neighbourhood of p into exactly two components (e.g. one component inside $(\cap_{i\in\phi}\mathcal{A}_c^i)^\circ$ and one component inside $(\cap_{i\in\phi}\mathcal{A}_c^i)^c$). Scenarios which satisfy Assumption 2.2.3 Statements (a) and (b) but violate the path-connectedness assumption are often difficult to visualise and rarely of practical interest. However, neglecting the path-connectedness assumption from consideration can have significant consequences. Fig. S2 provides an example in which both Assumption 2.2.3 Statements (a) and (b) are satisfied, but $\mathcal{F}_c$ is a fractal. Similarly, Fig. S3 provides an example, this time in 3 dimensions, in which the set $(\mathcal{F}_c)^\circ$ is not path-connected everywhere but instead consists of four conic regions whose apex meet at a single point $p \in \partial\mathcal{F}_c$.

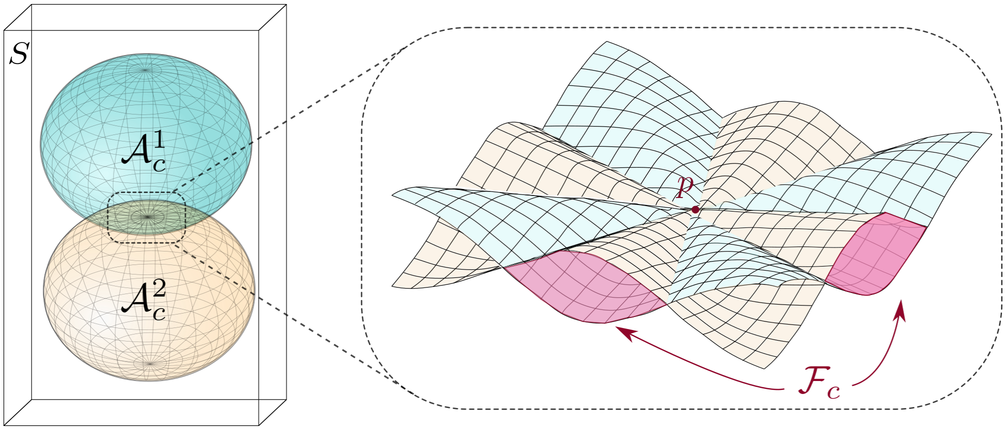

Figure S3: An example in which $N = 3$ and Assumption 2.2.3 Statements (a) and (b) are satisfied but the path-connectedness assumption is not. Here, the path-connectedness assumption does not hold at the point p, but (allowing artistic license) it may be assumed that all derivatives are continuous at p and meet the requirements of Assumption 2.2.3 Statement (b). In this instance, $(\mathcal{F}_c)^\circ$ possesses the undesirable property of consisting of multiple disconnected components (the conic regions highlighted in red) in all neighbourhoods of the point p. The theory presented in this work is not equipped to handle topologies of this form.

S3 From Conjunction to Disjunction Inference

Theorem 2.1 is key to generating CRs for conjunction inference (intersections of excursion sets). However, Theorem 2.1 may also be extended to allow investigation of other logical statements involving negations or disjunctions. Below we provide two corollaries, each of which is illustrated via a worked example. Corollary S3.1 extends Theorem 2.1 to account for logical negations, whilst Corollary S3.2 provides an equivalent statement for inference performed upon logical disjunctions (unions of excursion sets). In this section, for closed B, the notation B^1 and B^{-1} shall represent B and $\overline{B^c}$, respectively.

Example S1. Suppose, given two target functions, μ^1 and μ^2, we were interested in the region defined by the logical conjunction '$\mu^1 \geq c$ *and* $\mu^2 \leq c$' (i.e. "Where did a variable of interest exceed a threshold under one study condition *and not* under another?"). In the notation of Section 2.1, this region is readily seen to be given by $(\mathcal{A}_c^1)^1 \cap (\mathcal{A}_c^2)^{-1}$.

To apply the result of Theorem 2.1, we first define $\tilde{\mu}^1 = \mu^1$ and $\tilde{\mu}^2 = 2c - \mu^2$ and note that the logical statement '$\mu^1 \geq c$ *and* $\mu^2 \leq c$' is identical to the statement '$\tilde{\mu}^1 \geq c$ *and* $\tilde{\mu}^2 \geq c$'. By using the latter statement, Theorem 2.1 may now be applied to obtain the desired CRs (assuming that an appropriate mechanism exists for evaluating the quantiles of H). However, during this process, the limiting variables $\{G^i\}_{i\in\{1,2\}}$, which correspond to the target functions $\{\mu^i\}_{i\in\{1,2\}}$, must be replaced by variables corresponding to the functions $\{\tilde{\mu}^i\}_{i\in\{1,2\}}$, $\{\tilde{G}^i\}_{i\in\{1,2\}}$. By noting the definitions of $\{\tilde{\mu}^i\}_{i\in\{1,2\}}$ and $\{\tilde{G}^i\}_{i\in\{1,2\}}$, it can be seen that $\tilde{G}^1 = G^1$ and $\tilde{G}^2 = -G^2$.

In summary, a procedure for generating CRs corresponding to the logical conjunction '$\mu^1 \geq c$ *and* $\mu^2 \leq c$' would be identical to that previously described, except that G^2 would be substituted for $-G^2$ and $\partial\mathcal{F}_c$ would be substituted for $\partial((\mathcal{A}_c^1)^1 \cap (\mathcal{A}_c^2)^{-1})$. In general, this example may be extended to allow for inference to be performed upon arbitrary conjunctions of statements and negated statements. To achieve this, the definitions of $\mathcal{F}_c$, $\hat{\mathcal{F}}_c^-$ and $\hat{\mathcal{F}}_c^+$ must be extended as follows.

Corollary S3.1. *Let $\{\delta_i\}_{i\in\mathcal{M}}$ be an arbitrary sequence of integers in $\{-1,1\}$ and define $\mathcal{F}_c$ as $\mathcal{F}_c = \bigcap_{i\in\mathcal{M}}(\mathcal{A}_c^i)^{\delta_i}$. Similarly, extend the definition of the sets $\hat{\mathcal{F}}_c^+$ and $\hat{\mathcal{F}}_c^-$ to:*

$$\hat{\mathcal{F}}_c^+ = \bigcap_{i\in\mathcal{M}} \{s \in S : \tau_n^{-1}\hat{g}_n^i(s) \geq +a\}^{\delta_i} \quad \text{and} \quad \hat{\mathcal{F}}_c^- = \bigcap_{i\in\mathcal{M}} \{s \in S : \tau_n^{-1}\hat{g}_n^i(s) \geq -a\}^{\delta_i},$$

respectively and, for each $\phi \in \mathcal{P}^+(\mathcal{M})$, define $\partial^\phi \mathcal{F}_c$ as before. If $\mathcal{F}_c$ satisfies the assumptions of Section 2.2, then the below statement holds asymptotically:

$$\lim_{n\to\infty} \mathbb{P}[\hat{\mathcal{F}}_c^+ \subseteq \mathcal{F}_c \subseteq \hat{\mathcal{F}}_c^-] = \mathbb{P}\left[\max_{\phi\in\mathcal{P}^+(\mathcal{M})}\left(\sup_{s\in\partial^\phi\mathcal{F}_c}\left|\min_{i\in\phi}(\delta_i G^i(s))\right|\right) \leq a\right]$$

Example S2. Suppose, given two target functions, μ^1 and μ^2, interest lay in the logical disjunction of '$\mu^1 \geq c$ *or* $\mu^2 \geq c$' (i.e. "Where did *at least one* of the variables of interest exceed the threshold?"). The region of space over which this statement holds is given by $\mathcal{A}_c^1 \cup \mathcal{A}_c^2$, and shall be denoted $\mathcal{G}_c$.

To generate CRs for $\mathcal{G}_c$, we first assume that $\mathcal{G}_c$ satisfies Assumptions 2.2.1-2.2.3 Statement (b). By Assumptions 2.2.2 and 2.2.3 Statement (b) and De Morgan's law, it can be seen that $\mathcal{G}_c = ((\mathcal{A}_c^1)^{-1} \cap (\mathcal{A}_c^2)^{-1})^{-1}$. It must be noted that Assumptions 2.2.2 and 2.2.3 Statement (b) are necessary for this statement to hold due to the fact that $(\mathcal{A}_c^i)^{-1} = \overline{(\mathcal{A}_c^i)^c}$ and not $(\mathcal{A}_c^i)^c$. By employing Corollary S3.1, for a fixed value of α, CRs, $\hat{\mathcal{F}}_c^-$ and $\hat{\mathcal{F}}_c^+$, may be obtained which satisfy the following;

$$\lim_{n\to\infty} \mathbb{P}[(\hat{\mathcal{F}}_c^-)^c \subseteq ((\mathcal{A}_c^1)^{-1} \cap (\mathcal{A}_c^2)^{-1})^c \subseteq (\hat{\mathcal{F}}_c^+)^c] = 1-\alpha.$$

By taking the closure of each of the sets in the above probability, and again via careful consideration of Assumptions 2.2.2 and 2.2.3 Statement (b), the below is obtained;

$$\lim_{n\to\infty} \mathbb{P}[(\hat{\mathcal{F}}_c^-)^{-1} \subseteq \mathcal{G}_c \subseteq (\hat{\mathcal{F}}_c^+)^{-1}] = 1-\alpha.$$

In other words, the sets $(\hat{\mathcal{F}}_c^-)^{-1}$ and $(\hat{\mathcal{F}}_c^+)^{-1}$ serve as CRs for disjunction inference on μ^1 and μ^2. Note that, as in the previous example, great attention must paid to the signs which appear in front of the variables $\{G^i\}_{i\in\mathcal{M}}$ in the definition of H. As this example began by generating CRs for $(\mathcal{A}_c^1)^{-1} \cap (\mathcal{A}_c^2)^{-1}$, it can be seen that the variables G^1 and G^2 must be appended with negative signs (i.e. when applying the result of Corollary S3.1, both δ_1 and δ_2 were set to -1). This example may be expanded upon to obtain similar results for larger values of M, as shown by Corollary S3.2.

Corollary S3.2. *Let $\{\delta_i\}_{i\in\mathcal{M}}$ be an arbitrary sequence of integers in $\{-1,1\}$ and define $\mathcal{G}_c$ as $\mathcal{G}_c = \bigcup_{i\in\mathcal{M}}(\mathcal{A}_c^i)^{\delta_i}$. Define the sets $\hat{\mathcal{G}}_c^+$ and $\hat{\mathcal{G}}_c^-$ as:*

$$\hat{\mathcal{G}}_c^+ = \left(\bigcap_{i\in\mathcal{M}}\{s\in S : \tau_n^{-1}\hat{g}_n^i(s) \geq -a\}^{\delta_i}\right)^{-1} \quad \textit{and} \quad \hat{\mathcal{G}}_c^- = \left(\bigcap_{i\in\mathcal{M}}\{s\in S : \tau_n^{-1}\hat{g}_n^i(s) \geq +a\}^{\delta_i}\right)^{-1},$$

respectively and, for each $\phi \in \mathcal{P}^+(\mathcal{M})$*, define* $\partial^\phi \mathcal{G}_c$ *analogously to* $\partial^\phi \mathcal{F}_c$ *in the previous sections. If* $\mathcal{G}_c$ *satisfies the assumptions of Section 2.2, then the below statement holds asymptotically:*

$$\lim_{n\to\infty} \mathbb{P}[\hat{\mathcal{G}}_c^+ \subseteq \mathcal{G}_c \subseteq \hat{\mathcal{G}}_c^-] = \mathbb{P}\left[\max_{\phi \in \mathcal{P}^+(\mathcal{M})} \left(\sup_{s \in \partial^\phi \mathcal{G}_c} \big| \min_{i \in \phi}(-\delta_i G^i(s)) \big| \right) \leq a \right].$$

S4 Proof

In this section, we provide a full proof of Theorem S1. To do so, we shall prove each of the below statements in turn:

$$\liminf_{n\to\infty} \mathbb{P}[\hat{\mathcal{F}}_c^+ \subseteq \mathcal{F}_c \subseteq \hat{\mathcal{F}}_c^-] \geq \mathbb{P}\bigg[\max_{\phi\in\mathcal{P}^+(\mathcal{M})}\bigg(\sup_{s\in\partial^\phi\mathcal{F}_c}\big|\min_{i\in\phi}(G^i(s))\big|\bigg) \leq a\bigg], \tag{S1}$$

$$\limsup_{n\to\infty} \mathbb{P}[\hat{\mathcal{F}}_c^+ \subseteq \mathcal{F}_c \subseteq \hat{\mathcal{F}}_c^-] \leq \mathbb{P}\bigg[\max_{\phi\in\mathcal{P}^+(\mathcal{M})}\bigg(\sup_{s\in\partial^\phi\mathcal{F}_c}\big|\min_{i\in\phi}(G^i(s))\big|\bigg) \leq a\bigg]. \tag{S2}$$

It is clear that, when taken together, Equations (S1) and (S2) imply the result of Theorem S1. In Sections S4.1 and S4.2 we shall outline the proofs of Equations (S1) and (S2), respectively. Both Section S4.1 and Section S4.2 describe the 'broad strokes' of the proof, whilst the 'heavy lifting' is relegated to the theorems listed in Sections S4.4-S4.11, with supporting lemmas provided in Section S4.3.

Before proceeding, it must be noted that the excursion sets defined in Section 1 of the main text may be reformulated in terms of the functions $\{g^i\}_{i\in\mathcal{M}}$ and $\{\hat{g}_n^i\}_{i\in\mathcal{M}}$ (c.f. Section 2.2) as:

$$\mathcal{A}_c^i = \{s \in S : g^i(s) \geq 0\} \quad \text{and} \quad \hat{\mathcal{A}}_c^i = \{s \in S : \hat{g}_n^i(s) \geq 0\}.$$

In a similar manner, the sets $\mathcal{F}_c$ and $\hat{\mathcal{F}}_c$ may be reformulated as

$$\mathcal{F}_c = \{s \in S : \min_{i\in\mathcal{M}} g^i(s) \geq 0\} \quad \text{and} \quad \hat{\mathcal{F}}_c = \{s \in S : \min_{i\in\mathcal{M}} \hat{g}_n^i(s) \geq 0\},$$

respectively. By noting the above, the notations μ^i, $\hat{\mu}_n^i$ and σ^i may dispensed with and the following proof may be given solely in terms of the functions $\{g^i\}_{i\in\mathcal{M}}$ and $\{\hat{g}_n^i\}_{i\in\mathcal{M}}$.

S4.1 Part I: Proof of Equation (S1)

In this section, we shall prove Equation (S1). To do so, we begin by defining $\{\eta_n\}_{n\in\mathbb{N}}$ and $\{K_n\}_{n\in\mathbb{N}}$ as arbitrary sequences of positive integers which satisfy $\eta_n \to 0, \tau_n^{-1}\eta_n \to \infty, K_n \to \infty, K_n^M \eta_n \to 0$ and $K_n\tau_n^{-1}\eta_n \to \infty$ as $n \to \infty$. Lemma S1 demonstrates that it is always possible to find such sequences. Next, we define the inflated boundary for $\mathcal{F}_c$ as:

$$\mathcal{F}_c^n = \{s \in S : |\min_{i\in\mathcal{M}}(g^i(s))| \leq \eta_n\}.$$

And similarly, for $k \in \mathcal{M}$, we define the β-inflated boundary for $\mathcal{A}_c^k$ as:

$$\mathcal{A}_c^{\beta,k} = \{s \in S : |g^k(s)| \leq \beta\},$$

for arbitrary $\beta \in \mathbb{R}^+$. Intuitively, $\mathcal{F}_c^n$ may be expected to form an inflated 'band' around $\partial\mathcal{F}_c$ which shrinks to $\partial\mathcal{F}_c$ as $n \to \infty$. Similarly, the η_n-inflated boundary for $\partial\mathcal{A}_c^k$, $\mathcal{A}_c^{\eta_n,k}$, forms an inflated 'band' around $\partial\mathcal{A}_c^k$ and shrinks to $\partial\mathcal{A}_c^k$ as $n \to \infty$. Using the above definitions, our aim is now to build similar 'inflated' boundaries for the sets $\{\partial^\phi\mathcal{F}_c\}_{\phi\in\mathcal{P}^+(\mathcal{M})}$.

For each $\phi \in \mathcal{P}^+(\mathcal{M})$, we shall meet this aim by defining two 'components' of the inflated boundary for $\partial^\phi\mathcal{F}_c$; an 'inner' component (i.e. the component which lies inside the set $\mathcal{F}_c$) and an 'outer' component (e.g. the component which lies outside the set $\mathcal{F}_c$). For all $\phi \in \mathcal{P}^+(\mathcal{M})$, the inner component, $\mathcal{I}_c^{n,\phi}$, is defined as;

$$\mathcal{I}_c^{n,\phi} = \mathcal{F}_c \cap \bigg(\bigcap_{i\in\phi} \mathcal{A}_c^{(K_n)^{|\phi|-1}\eta_n,i}\bigg) \bigg\backslash \bigg(\bigcup_{\substack{\phi'\in\mathcal{P}^+(\mathcal{M}) \\ \text{Ord}(\phi')>\text{Ord}(\phi)}} \mathcal{I}_c^{n,\phi'}\bigg), \tag{S3}$$

where 'Ord' represents the index of a set in $\mathcal{P}^+(\mathcal{M})$ when $\mathcal{P}^+(\mathcal{M})$ is listed in lexicographic order. For example, if $M = 3$ then the Ord maps the sets $(\{1\}, \{2\}, \{3\}, \{1, 2\}, \{1, 3\}, ...)$, to the integers $(1, 2, 3, 4, 5, ...)$.

The outer component, which will be denoted $\mathcal{O}_c^{n,\phi}$, is defined for $\phi \in \mathcal{P}^+(\mathcal{M})$ as:

$$\mathcal{O}_c^{n,\phi} = \mathcal{F}_c^n \cap \bigg(\bigcap_{i\in\phi}(\mathcal{A}_c^i)^c\bigg) \cap \bigg(\bigcap_{j\in\mathcal{M}\setminus\phi} \mathcal{A}_c^j\bigg), \tag{S4}$$

where, if $\mathcal{M} \setminus \phi$ is empty, the last term is defined to be equal to S. To aid understanding, in Section S4.1.1, we provide further discussion of the definitions of $\mathcal{I}_c^{n,\phi}$ and $\mathcal{O}_c^{n,\phi}$. For

now, it suffices to state that $\mathcal{I}_c^{n,\phi}$ and $\mathcal{O}_c^{n,\phi}$ are sets, located on the inside and outside of $\mathcal{F}_c$ respectively, each of which increasingly resembles $\partial^\phi \mathcal{F}_c$ as $n \to \infty$. Illustrations of both sets are provided in Section S4.1.1 (c.f. Figs. S4 and S5).

Next, we define the sets $\mathcal{O}_c^{n,\emptyset}$ and $\mathcal{I}_c^{n,\emptyset}$ as the regions outside and inside $\mathcal{F}_c$, which are not covered by the sets $\{\mathcal{O}_c^{n,\phi}\}_{\phi\in\mathcal{P}^+(\mathcal{M})}$ and $\{\mathcal{I}_c^{n,\phi}\}_{\phi\in\mathcal{P}^+(\mathcal{M})}$, respectively. I.e:

$$\mathcal{O}_c^{n,\emptyset} = (\mathcal{F}_c)^c \Bigg\backslash \Bigg(\bigcup_{\phi\in\mathcal{P}^+(\mathcal{M})} \mathcal{O}_c^{n,\phi} \Bigg), \qquad \mathcal{I}_c^{n,\emptyset} = \mathcal{F}_c \Bigg\backslash \Bigg(\bigcup_{\phi\in\mathcal{P}^+(\mathcal{M})} \mathcal{I}_c^{n,\phi} \Bigg).$$

It now follows that the sets $\{\mathcal{O}_c^{n,\phi}\}_{\phi\in\mathcal{P}(\mathcal{M})}$ and $\{\mathcal{I}_c^{n,\phi}\}_{\phi\in\mathcal{P}(\mathcal{M})}$ cover $(\mathcal{F}_c)^c$ and $\mathcal{F}_c$, respectively. We now define the 'inflated' boundary for $\partial^\phi\mathcal{F}_c$ as $\mathcal{F}_c^{n,\phi} = \mathcal{O}_c^{n,\phi} \cup \mathcal{I}_c^{n,\phi}$.

Finally, we define the following events, for $\phi \in \mathcal{P}^+(\mathcal{M})$:

$$\mathcal{E}_{\mathcal{I}}^{\phi,n} = \Bigg\{ \sup_{s\in\mathcal{I}_c^{n,\phi}} \Bigg| \tau_n^{-1} \min\Bigg(\min_{i\in\phi}(\hat{g}_n^i(s) - g^i(s)), \min_{j\in\mathcal{M}\setminus\phi}(\hat{g}_n^j(s)) \Bigg) \Bigg| \leq a \Bigg\},$$

$$\mathcal{E}_{\mathcal{O}}^{\phi,n} = \Bigg\{ \sup_{s\in\mathcal{O}_c^{n,\phi}} \Bigg| \tau_n^{-1} \min_{i\in\phi}(\hat{g}_n^i(s) - g^i(s)) \Bigg| \leq a \Bigg\},$$

and, for the empty set, $\emptyset$;

$$\mathcal{E}_{\mathcal{I}}^{\emptyset,n} = \Bigg\{ \sup_{s\in\mathcal{I}_c^{n,\emptyset}} \max_{i\in\mathcal{M}} \Bigg(\tau_n^{-1} |\hat{g}_n^i(s) - g^i(s)| \Bigg) \leq a + \tau_n^{-1}\eta_n \Bigg\},$$

$$\mathcal{E}_{\mathcal{O}}^{\emptyset,n} = \Bigg\{ \sup_{s\in\mathcal{O}_c^{n,\emptyset}} \max_{i\in\mathcal{M}} \Bigg(\tau_n^{-1} |\hat{g}_n^i(s) - g^i(s)| \Bigg) \leq a + \tau_n^{-1}\eta_n \Bigg\}.$$

In the following text, if $\mathcal{M} \setminus \phi$ is empty, it will be assumed that any minimum function taken over $\mathcal{M} \setminus \phi$ is defined as $+\infty$ (this is the equivalent of 'removing' such terms from the proof entirely when $\phi = \mathcal{M}$).

We now are in a position to begin the proof. The first step of the proof is to show that if the events $\mathcal{E}_{\mathcal{I}}^{\phi,n}$ and $\mathcal{E}_{\mathcal{O}}^{\phi,n}$ occur for all $\phi \in \mathcal{P}(\mathcal{M})$, then the inclusion $\hat{\mathcal{F}}_c^+ \subseteq \mathcal{F}_c \subseteq \hat{\mathcal{F}}_c^-$ holds. This implication is proven by Theorem S2 in Section S4.4. From this result and by using De Morgan's law, it can now be seen that:

$$\mathbb{P}[\hat{\mathcal{F}}_c^+ \subseteq \mathcal{F}_c \subseteq \hat{\mathcal{F}}_c^-] \geq \mathbb{P}\Bigg[\bigcap_{\phi\in\mathcal{P}^+(\mathcal{M})} \{\mathcal{E}_{\mathcal{I}}^{\phi,n} \wedge \mathcal{E}_{\mathcal{O}}^{\phi,n}\} \Bigg] + \mathbb{P}[\mathcal{E}_{\mathcal{I}}^{\emptyset,n} \wedge \mathcal{E}_{\mathcal{O}}^{\emptyset,n}] - 1.$$

Next, we apply $\liminf_{n\to\infty}$ to both sides of the above expression. By the definitions of $\mathcal{E}_{\mathcal{I}}^{\emptyset,n}$ and $\mathcal{E}_{\mathcal{O}}^{\emptyset,n}$ it can be seen that the second term on the right hand side becomes:

$$\mathbb{P}[\mathcal{E}_{\mathcal{I}}^{\emptyset,n} \wedge \mathcal{E}_{\mathcal{O}}^{\emptyset,n}] = \mathbb{P}\bigg[\sup_{s\in\mathcal{F}_c^{n,\emptyset}} \max_{i\in\mathcal{M}} \bigg(\tau_n^{-1}|\hat{g}_n^i(s) - g^i(s)|\bigg) \leq a + \tau_n^{-1}\eta_n\bigg] \xrightarrow{n\to\infty} 1.$$

The above convergence follows from the fact that $\tau_n^{-1}\eta_n \to \infty$ as $n \to \infty$, whilst the expression involving the suprema and maxima converges in distribution to a well-defined, random variable by Assumption 2.2.1. It can now be seen that:

$$\liminf_{n\to\infty} \mathbb{P}[\hat{\mathcal{F}}_c^+ \subseteq \mathcal{F}_c \subseteq \hat{\mathcal{F}}_c^-] \geq \liminf_{n\to\infty} \mathbb{P}\bigg[\bigcap_{\phi\in\mathcal{P}^+(\mathcal{M})} \{\mathcal{E}_{\mathcal{I}}^{\phi,n} \wedge \mathcal{E}_{\mathcal{O}}^{\phi,n}\}\bigg]. \tag{S5}$$

We now define the following shorthand:

$$A_\phi^{n,\mathcal{I}} = \sup_{s\in\mathcal{I}_c^{n,\phi}} \bigg|\tau_n^{-1} \min\bigg(\min_{i\in\phi}(\hat{g}_n^i(s) - g^i(s)), \min_{j\in\mathcal{M}\setminus\phi}(\hat{g}_n^j(s))\bigg)\bigg|,$$

$$B_\phi^{n,\mathcal{I}} = \sup_{s\in\mathcal{I}_c^{n,\phi}} \bigg|\tau_n^{-1} \min_{i\in\phi}(\hat{g}_n^i(s) - g^i(s))\bigg|, \quad B_\phi^{n,\mathcal{O}} = A_\phi^{n,\mathcal{O}} = \sup_{s\in\mathcal{O}_c^{n,\phi}} \bigg|\tau_n^{-1} \min_{i\in\phi}(\hat{g}_n^i(s) - g^i(s))\bigg|.$$

And define the following four quantities:

$$A_\phi^n = \max(A_\phi^{n,\mathcal{I}}, A_\phi^{n,\mathcal{O}}), \qquad B_\phi^n = \max(B_\phi^{n,\mathcal{I}}, B_\phi^{n,\mathcal{O}}),$$

$$C_\phi^n = \sup_{s\in\partial^\phi\mathcal{F}_c} \bigg|\tau_n^{-1} \min_{i\in\phi}(\hat{g}_n^i(s) - g^i(s))\bigg|, \qquad D_\phi = \sup_{s\in\partial^\phi\mathcal{F}_c} |\min_{i\in\phi}(G^i(s))|.$$

Using theorems given in Sections S4.5 and S4.6, alongside Assumption 2.2.1, we now note that the below relations hold:

$$\max_{\phi\in\mathcal{P}^+(\mathcal{M})}(A_\phi^n) - \max_{\phi\in\mathcal{P}^+(\mathcal{M})}(B_\phi^n) \xrightarrow{p} 0, \qquad \text{(By the result of Theorem S3)}$$

$$\max_{\phi\in\mathcal{P}^+(\mathcal{M})}(B_\phi^n) - \max_{\phi\in\mathcal{P}^+(\mathcal{M})}(C_\phi^n) \xrightarrow{p} 0, \qquad \text{(By the result of Theorem S4)}$$

$$\max_{\phi\in\mathcal{P}^+(\mathcal{M})}(C_\phi^n) - \max_{\phi\in\mathcal{P}^+(\mathcal{M})}(D_\phi) \xrightarrow{d} 0, \qquad \text{(By Assumption 2.2.1)}$$

where $\xrightarrow{p}$ and $\xrightarrow{d}$ represent convergence in probability and distribution, respectively. By combining the above results, it can now be seen that:

$$\max_{\phi\in\mathcal{P}^+(\mathcal{M})}(A_\phi^n) \xrightarrow{d} \max_{\phi\in\mathcal{P}^+(\mathcal{M})}(D_\phi).$$

It now follows that:

$$\liminf_{n\to\infty} \mathbb{P}\left[\bigcap_{\phi\in\mathcal{P}^+(\mathcal{M})} \{\mathcal{E}_{\mathcal{I}}^{\phi,n} \wedge \mathcal{E}_{\mathcal{O}}^{\phi,n}\}\right] = \liminf_{n\to\infty} \mathbb{P}\left[\max_{\phi\in\mathcal{P}^+(\mathcal{M})} (A_\phi^n) \leq a\right]$$

$$= \mathbb{P}\left[\max_{\phi\in\mathcal{P}^+(\mathcal{M})} (D_\phi) \leq a\right] = \mathbb{P}\left[\max_{\phi\in\mathcal{P}^+(\mathcal{M})} \left(\sup_{s\in\partial^\phi\mathcal{F}_c} |\min_{i\in\phi}(G^i(s))|\right) \leq a\right],$$

where the first equality follows from the definition of A_ϕ^n, the second follows from the previous argument, and the third follows from the definition of D_ϕ. By combining the above with Equation (S5), it can now be seen that Statement (S1) holds, as desired.

S4.1.1 Intuition for $\mathcal{I}_c^{n,\phi}$ and $\mathcal{O}_c^{n,\phi}$

Here, we provide further discussion of the definitions of $\{\mathcal{I}_c^{n,\phi}\}_{\phi\in\mathcal{P}^+(\mathcal{M})}$ and $\{\mathcal{O}_c^{n,\phi}\}_{\phi\in\mathcal{P}^+(\mathcal{M})}$. Illustrations of $\{\mathcal{I}_c^{n,\phi}\}_{\phi\in\mathcal{P}^+(\mathcal{M})}$ and $\{\mathcal{O}_c^{n,\phi}\}_{\phi\in\mathcal{P}^+(\mathcal{M})}$ are provided by Figs. S4 and S5, respectively, with a corresponding illustration of $\{\partial^\phi\mathcal{F}_c\}_{\phi\in\mathcal{P}^+(\mathcal{M})}$ given by Fig. S6.

To begin, we fix $\phi \in \mathcal{P}^+(\mathcal{M})$ and describe why each of the terms appearing in the definition of $\mathcal{I}_c^{n,\phi}$, Equation (S3), is necessary. The first term in Equation (S3) is $\mathcal{F}_c$, whose inclusion guarantees that $\mathcal{I}_c^{n,\phi}$ lies inside $\mathcal{F}_c$ (thus living up to the name "inner"). The second term is $\cap_{i\in\phi}\mathcal{A}_c^{(K_n)^{|\phi|-1}\eta_n,i}$. To understand the role of this term note that, by construction, $(K_n)^{|\phi|-1}\eta_n \to 0$ as $n \to \infty$. From the definition of the β-inflated boundary for $\mathcal{A}_c^i$, it follows that, for all $i \in \mathcal{M}$, $\mathcal{A}_c^{(K_n)^{|\phi|-1}\eta_n,i}$ must 'shrink' towards $\partial\mathcal{A}_c^i$ as $n \to \infty$. By consequence, the set $\mathcal{F}_c \cap (\cap_{i\in\phi}\mathcal{A}_c^{(K_n)^{|\phi|-1}\eta_n,i})$ is expected to 'shrink'* to $\mathcal{F}_c \cap (\cap_{i\in\phi}\partial\mathcal{A}_c^i) = \cup_{\phi'\supseteq\phi}\partial^{\phi'}\mathcal{F}_c$ as $n \to \infty$.

In the following sections, we require $\mathcal{I}_c^{n,\phi}$ to shrink to a set resembling $\partial^\phi\mathcal{F}_c = \cup_{\phi'\supseteq\phi}\partial^{\phi'}\mathcal{F}_c \setminus \cup_{\phi'\supset\phi}\partial^{\phi'}\mathcal{F}_c$. To remove $\cup_{\phi'\supset\phi}\partial^{\phi'}\mathcal{F}_c$ from consideration, the union which appears following the set minus in Equation (S3) is required. This term ensures that $\mathcal{I}_c^{n,\phi}$ does not intersect the set $\cup_{\phi'\supset\phi}\mathcal{I}_c^{n,\phi'}$†. However, if $\cup_{\phi'\supset\phi}\mathcal{I}_c^{n,\phi'}$ were to shrink to $\cup_{\phi'\supset\phi}\partial^{\phi'}\mathcal{F}_c$ this would still not necessarily guarantee that $\mathcal{I}_c^{n,\phi} \setminus \cup_{\phi'\supset\phi}\mathcal{I}_c^{n,\phi'}$ would shrink to a set which does not contain

*We note here that we have not defined the word 'shrink'. In the following sections, 'shrink' shall mean 'converge in Hausdorff distance'. For ease of reading, in this section we shall continue to use 'shrink'.

†In fact, the inclusion of the union guarantees something slightly stronger; that $\mathcal{I}_c^{n,\phi}$ does not intersect $\cup_{\text{Ord}(\phi')>\text{Ord}(\phi)}\mathcal{I}_c^{n,\phi'}$. This slight deviation is to ensure the sets $\{\mathcal{I}_c^{n,\phi}\}_{\phi\in\mathcal{P}^+(\mathcal{M})}$ are disjoint.

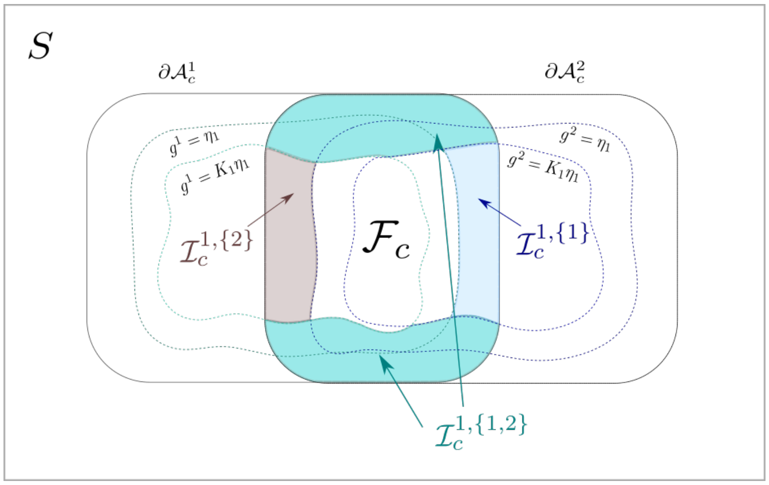

Figure S4: Illustration of the inner inflated sets in a setting in which $n = 1$, $M = 2$ and the excursion sets $\mathcal{A}_c^1$ and $\mathcal{A}_c^2$ both resemble rectangles with rounded corners. Shown are the inner inflated sets for $\phi = \{1\}$ (light blue), $\phi = \{2\}$ (light brown) and $\phi = \{1, 2\}$ (green). Also depicted are the contours $g^1 = \eta_1$, $g^2 = \eta_1$, $g^1 = K_1\eta_1$ and $g^2 = K_1\eta_1$.

$\cup_{\phi'\supset\phi}\partial^{\phi'}\mathcal{F}_c$. For this reason, it is an additional requirement that $\cup_{\phi'\supset\phi}\mathcal{I}_c^{n,\phi'}$ must shrink to $\cup_{\phi'\supset\phi}\partial^{\phi'}\mathcal{F}_c$ at a much 'slower rate' than the rate at which $\mathcal{I}_c^{n,\phi}$ shrinks to $\partial^{\phi}\mathcal{F}_c$. This requirement is handled by the constant $(K_n)^{|\phi|-1}$ which appears earlier in the definition of the inflated boundaries. As, for all $\phi' \supset \phi$, $|\phi'| \geq |\phi| + 1$, it follows that the rate at which $\mathcal{I}_c^{n,\phi}$ shrinks is, in some sense, at least a factor of K_n faster than the rate at which $\cup_{\phi'\supset\phi}\mathcal{I}_c^{n,\phi'}$ shrinks. As $K_n \to \infty$ as $n \to \infty$, it may be expected that this ensures $\mathcal{I}_c^{n,\phi}$ cannot shrink to a region containing $\cup_{\phi'\supset\phi}\partial^{\phi'}\mathcal{F}_c$. This intuition is argued formally in Section S4.6.

Visually, this concept may be understood by considering Fig. S4 and taking $\phi = \{1\}$ and $\phi' = \{1, 2\}$. In this case, for $n = 1$, $\mathcal{I}_c^{n,\phi}$ is represented by the light blue region, whilst $\mathcal{I}_c^{n,\phi'}$ is represented by the green region. In this example, $\mathcal{I}_c^{n,\phi}$ is much 'thinner' than $\mathcal{I}_c^{n,\phi'}$. In general, as $K_n \to \infty$ when $n \to \infty$, it is expected that the contour $g^1 = \eta_n$ will 'expand' outwards towards $\partial\mathcal{A}_c^1$ at a much faster rate than the rate at which the contour $g^2 = K_n\eta_n$ will 'expand' outwards towards $\partial\mathcal{A}_c^2$. By consequence, in the limit, $\mathcal{I}_c^{n,\phi}$ will shrink 'faster' than $\mathcal{I}_c^{n,\phi'}$ (the light blue region will continue to be much 'thinner' than the green region). As a result, it is not possible for $\mathcal{I}_c^{n,\phi}$ to 'shrink' to a region which includes the set $\partial^{\phi'}\mathcal{F}_c$ (the light blue region cannot 'get close' to arbitrary points belonging to the the top or the

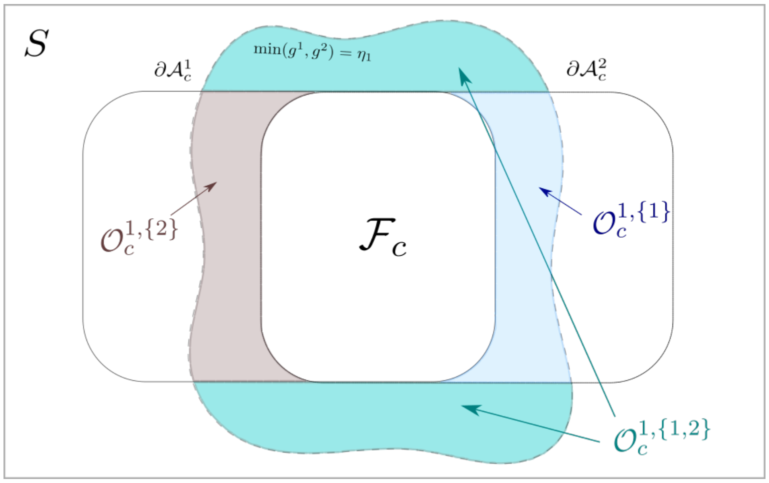

Figure S5: Illustration of the outer inflated sets in a setting in which $n = 1$, $M = 2$ and the excursion sets $\mathcal{A}_c^1$ and $\mathcal{A}_c^2$ both resemble rectangles with rounded corners. Shown are the outer inflated sets for $\phi = \{1\}$ (light blue), $\phi = \{2\}$ (light brown) and $\phi = \{1, 2\}$ (green). Also depicted is the contour $\min(g^1, g^2) = \eta_1$, which corresponds to the outer boundary of the set $\mathcal{F}_c^1$.

bottom of $\partial\mathcal{F}_c$).

It is worth briefly recapping the roles of the sequences $\{\tau_n\}_{n\in\mathbb{N}}$, $\{\eta_n\}_{n\in\mathbb{N}}$ and $\{K_n\}_{n\in\mathbb{N}}$. The sequence $\{\tau_n\}_{n\in\mathbb{N}}$ is typically a measurement of sample size or statistical power and is dictated by application. In contrast, $\{\eta_n\}_{n\in\mathbb{N}}$ and $\{K_n\}_{n\in\mathbb{N}}$ are synthetic sequences constructed for the purposes of proof. As $n \to \infty$, $\eta_n \to 0$ in order to ensure that the inner inflated sets, $\{\mathcal{I}_c^{n,\phi}\}_{\phi\in\mathcal{P}^+(\mathcal{M})}$, shrink to resemble the partitioned boundary sets, $\{\partial^\phi\mathcal{F}_c\}_{\phi\in\mathcal{P}^+(\mathcal{M})}$. Conversely, $K_n \to \infty$ as $n \to \infty$ and is used to distinguish between the rates of convergence of different inner inflated sets.

We now consider the definition of the 'outer' inflated boundaries $\{\mathcal{O}_c^{n,\phi}\}_{\phi\in\mathcal{P}^+(\mathcal{M})}$, given by Equation (S4). The first term on the right hand side of Equation (S4) is the set $\mathcal{F}_c^n$ whose inclusion guarantees that the set $\mathcal{O}_c^{n,\phi}$ must shrink to some subset of $\partial\mathcal{F}_c$ as $n \to \infty$. The second and third terms strongly resemble the definition of $\mathcal{J}_c^\phi$ given in Section 2.2 of the main text. The intuition behind the construction of $\mathcal{J}_c^\phi$ has already been discussed in Section S2.1 and shall not recounted again here. Here, it suffices to note that, for fixed $\phi \in \mathcal{P}^+(\mathcal{M})$, the second and third terms describe the region outside $\mathcal{F}_c$ (thus living up to

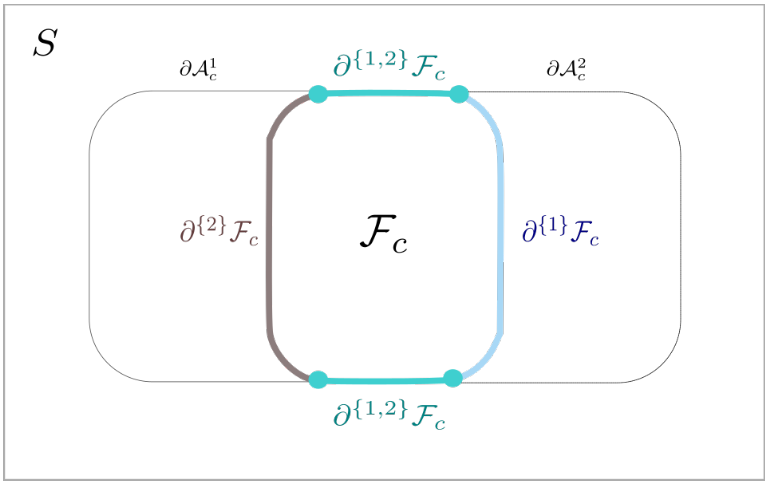

Figure S6: Illustration of the boundary segments in a setting in which the excursion sets $\mathcal{A}_c^1$ and $\mathcal{A}_c^2$ both resemble rectangles with rounded corners. Shown are the boundary segments for $\phi = \{1\}$ (light blue), $\phi = \{2\}$ (light brown) and $\phi = \{1, 2\}$ (green).

the name 'outer') which we may 'naturally expect' to border the boundary segment $\partial^\phi \mathcal{F}_c$.

In summary, for fixed $\phi \in \mathcal{P}^+(\mathcal{M})$, intuition suggests that the sets $\mathcal{O}_c^{n,\phi}$ and $\mathcal{I}_c^{n,\phi}$ border the boundary segment $\partial^\phi \mathcal{F}_c$ and shrinks towards it as $n \to \infty$. This intuition can still fail, however, as in certain circumstances the sets $\mathcal{I}_c^{n,\phi}$ and $\mathcal{O}_c^{n,\phi}$ can shrink to sets which contain $\partial^\phi \mathcal{F}_c$ and have equal measure to $\partial^\phi \mathcal{F}_c$, but do not equal $\partial^\phi \mathcal{F}_c$ (c.f. Fig. S7 in Section S4.6). Such circumstances are explicitly handled by the theorems presented in Sections S4.8 and S4.9.

S4.2 Part II: Proof of Equation (S2)

In this section, we shall prove Equation (S2). To do so, we first define, for every $\phi \in \mathcal{P}^+(\mathcal{M})$, the 'shrunken' boundary for $\partial^\phi \mathcal{F}_c$ as:

$$\mathcal{F}_c^{-n,\phi} = \{s \in S : \forall i \in \phi, g^i(s) = 0 \text{ and } \forall j \in \mathcal{M} \setminus \phi, g^j(s) > \eta_n\}.$$

$\mathcal{F}_c^{-n,\phi}$ is a subset of $\partial^\phi \mathcal{F}_c$ (thus living up to the name 'shrunken') and is expected to increasingly resemble $\partial^\phi \mathcal{F}_c$ as $n \to \infty$. Next, we define the shrunken boundary for $\partial \mathcal{F}_c$, as $\mathcal{F}_c^{-n} = \cup_{\phi \in \mathcal{P}^+(\mathcal{M})} \mathcal{F}_c^{-n,\phi}$.

Now, suppose the below statement were true:

$$\exists \delta > 0, s \in \mathcal{F}_c^{-n} \text{ such that } \tau_n^{-1} \min_{i \in \mathcal{M}} (\hat{g}_n^i(s)) \geq a + \delta. \tag{S6}$$

As, for n large enough, $\tau_n^{-1} \min_{i \in \mathcal{M}} (\hat{g}_n^i)$ is continuous, it follows that, for n large enough, there must be an $s \in \mathcal{F}_c^{-n}$ such that $\tau_n^{-1} \min_{i \in \mathcal{M}} (\hat{g}_n^i) > a$ in some open neighbourhood of s. As such a neighbourhood must intersect $\mathcal{J}_c^\phi$ by the ball assumption, the below now follows:

$$\exists \tilde{s} \in \mathcal{J}_c^\phi \subseteq (\mathcal{F}_c)^c \text{ such that } \tau_n^{-1} \min_{i \in \mathcal{M}} (\hat{g}_n^i(\tilde{s})) > a.$$

And therefore, by the definitions of $\mathcal{F}_c$ and $\hat{\mathcal{F}}_c^+$, it follows that there exists an $\tilde{s} \in (\mathcal{F}_c)^c$ which also belongs to $\hat{\mathcal{F}}_c^+$. Therefore, $\hat{\mathcal{F}}_c^+ \not\subseteq \mathcal{F}_c$.

Through similar logic, it can be seen that if the below statement is true:

$$\exists \delta > 0, s \in \mathcal{F}_c^{-n} \text{ such that } \tau_n^{-1} \min_{i \in \mathcal{M}} (\hat{g}_n^i(s)) \leq -a - \delta, \tag{S7}$$

then $\mathcal{F}_c \not\subseteq \hat{\mathcal{F}}_c^-$. By combining Statements (S6) and (S7) and the above arguments it can now be seen that if the below statement holds:

$$\exists \delta > 0, s \in \mathcal{F}_c^{-n} \text{ such that } \tau_n^{-1} |\min_{i \in \mathcal{M}} (\hat{g}_n^i(s))| \geq a + \delta,$$

then it cannot be the case that the inclusion statement, $\hat{\mathcal{F}}_c^+ \subseteq \mathcal{F}_c \subseteq \hat{\mathcal{F}}_c^-$, holds. Therefore:

$$1 - \mathbb{P}[\hat{\mathcal{F}}_c^+ \subseteq \mathcal{F}_c \subseteq \hat{\mathcal{F}}_c^-] \geq \mathbb{P}\left[\exists \delta > 0 \text{ such that } \sup_{s \in \mathcal{F}_c^{-n}} |\tau_n^{-1} \min_{i \in \mathcal{M}} (\hat{g}_n^i(s))| \geq a + \delta\right]$$

$$= \mathbb{P}\bigg[\sup_{s\in\mathcal{F}_c^{-n}} |\tau_n^{-1}\min_{i\in\mathcal{M}}(\hat{g}_n^i(s))| > a\bigg].$$

By rearranging the above, the following may be obtained:

$$\mathbb{P}[\hat{\mathcal{F}}_c^+ \subseteq \mathcal{F}_c \subseteq \hat{\mathcal{F}}_c^-] \leq \mathbb{P}\bigg[\max_{\phi\in\mathcal{P}^+(\mathcal{M})}\bigg(\sup_{s\in\mathcal{F}_c^{-n,\phi}} |\tau_n^{-1}\min_{i\in\mathcal{M}}(\hat{g}_n^i(s))|\bigg) \leq a\bigg].$$

The aim is now to show that, as $n\to\infty$, the limit supremum of the probability appearing on the right hand side of the above tends to the right hand side of Equation (S2). To achieve this, we define the following four quantities, for $\phi \in \mathcal{P}^+(\mathcal{M})$:

$$W_\phi^n = \sup_{s\in\mathcal{F}_c^{-n,\phi}} \left|\tau_n^{-1}\min_{i\in\mathcal{M}}(\hat{g}_n^i(s))\right|, \qquad X_\phi^n = \sup_{s\in\mathcal{F}_c^{-n,\phi}} \left|\tau_n^{-1}\min_{i\in\phi}(\hat{g}_n^i(s)-g^i(s))\right|,$$

$$Y_\phi^n = \sup_{s\in\partial^\phi\mathcal{F}_c} \left|\tau_n^{-1}\min_{i\in\phi}(\hat{g}_n^i(s)-g^i(s))\right|, \qquad Z_\phi = \sup_{s\in\partial^\phi\mathcal{F}_c} |\min_{i\in\phi}(G^i(s))|.$$

Using theorems given in Sections S4.10 and S4.11, alongside Assumption 2.2.1, we now note that the below relations hold:

$$\max_{\phi\in\mathcal{P}^+(\mathcal{M})}(W_\phi^n) - \max_{\phi\in\mathcal{P}^+(\mathcal{M})}(X_\phi^n) \xrightarrow{p} 0, \qquad \text{(By the result of Theorem S8)}$$

$$\max_{\phi\in\mathcal{P}^+(\mathcal{M})}(X_\phi^n) - \max_{\phi\in\mathcal{P}^+(\mathcal{M})}(Y_\phi^n) \xrightarrow{p} 0, \qquad \text{(By the result of Theorem S9)}$$

$$\max_{\phi\in\mathcal{P}^+(\mathcal{M})}(Y_\phi^n) - \max_{\phi\in\mathcal{P}^+(\mathcal{M})}(Z_\phi) \xrightarrow{d} 0, \qquad \text{(By Assumption 2.2.1)}$$

where $\xrightarrow{p}$ and $\xrightarrow{d}$ represent convergence in probability and distribution, respectively. By combining the above results, it can now be seen that:

$$\max_{\phi\in\mathcal{P}^+(\mathcal{M})}(W_\phi^n) \xrightarrow{d} \max_{\phi\in\mathcal{P}^+(\mathcal{M})}(Z_\phi).$$

Combining the preceding arguments, it now follows that:

$$\limsup_{n\to\infty}\mathbb{P}[\hat{\mathcal{F}}_c^+ \subseteq \mathcal{F}_c \subseteq \hat{\mathcal{F}}_c^-] \leq \limsup_{n\to\infty}\mathbb{P}\bigg[\max_{\phi\in\mathcal{P}^+(\mathcal{M})}(W_\phi^n) \leq a\bigg]$$

$$= \mathbb{P}\bigg[\max_{\phi\in\mathcal{P}^+(\mathcal{M})}(Z_\phi) \leq a\bigg] = \mathbb{P}\bigg[\max_{\phi\in\mathcal{P}^+(\mathcal{M})}\bigg(\sup_{s\in\partial^\phi\mathcal{F}_c}\big|\min_{i\in\phi}(G^i(s))\big|\bigg) \leq a\bigg],$$

where the inequality follows from the definition of W_ϕ^n, the first equality follows from the argument above, and the second equality follows from the definition of Z_ϕ. It now follows that Equation (S2) holds. □

S4.3 Supporting Lemmas

Lemma S1. *Given an arbitrary positive sequence* $\{\tau_n\}_{n\in\mathbb{N}}$ *such that* $\tau_n \to 0$ *as* $n \to \infty$*, it is possible to define two positive sequences* $\{\eta_n\}_{n\in\mathbb{N}}$ *and* $\{K_n\}_{n\in\mathbb{N}}$ *such that, as* $n \to \infty$*:*

$$\eta_n \to 0, \quad \tau_n^{-1}\eta_n \to \infty, \quad K_n \to \infty, \quad K_n^M \eta_n \to 0 \quad \text{and} \quad K_n\tau_n^{-1}\eta_n \to \infty.$$

Proof. The above limits are satisfied by choosing $\eta_n = \tau_n^{\frac{1}{2}}$ and $K_n = \eta_n^{-\frac{1}{2M}}$. □

Lemma S2. *For arbitrary* a, b, c *and* d*, it is true that:*

$$\min(a-c, b-d) \leq \min(a,b) - \min(c,d) \leq \max(a-c, b-d).$$

Lemma S3. *If* $\{A_n\}_{n\in\mathbb{N}}, \{\tilde{A}_n\}_{n\in\mathbb{N}}$ *and* $\{B_n\}_{n\in\mathbb{N}}$ *are sequences of random variables and* $A_n - \tilde{A}_n \xrightarrow{p} 0$ *then the below convergence in probability holds:*

$$\max(A_n, B_n) - \max(\tilde{A}_n, B_n) \xrightarrow{p} 0.$$

Lemma S4. *For functions* a *and* b *acting on a closed set* X*, it is true that:*

$$\left| \sup_{x\in X} |a(x)| - \sup_{x\in X} |b(x)| \right| \leq \sup_{x\in X} \left| a(x) - b(x) \right|.$$

Lemma S5. *If* $\{A_n\}_{n\in\mathbb{N}}$ *and* $\{B_n\}_{n\in\mathbb{N}}$ *are sequences of random variables which jointly tend in probability to random variables* A *and* B*, respectively, then the below convergence in probability holds:*

$$\max(A_n, B_n) \xrightarrow{p} \max(A, B).$$

Lemma S6. *For every $\phi \in \mathcal{P}^+(\mathcal{M})$, it is true that;*

$$\forall s \in \mathcal{I}_c^{n,\phi}, \min_{j \in \mathcal{M} \setminus \phi} (g^j(s)) \geq K_n^{|\phi|} \eta_n.$$

Proof. We can discount the case $\phi = \mathcal{M}$ as the minimum taken over the empty set is equal to $+\infty$ (see Section S4.1). For $\phi \neq \mathcal{M}$, suppose that the negation of the above were true and fix $s \in \mathcal{I}_c^{n,\phi}$ and $j \in \mathcal{M} \setminus \phi$ such that $g^j(s) < K_n^{|\phi|} \eta_n$. By the definition of $\mathcal{I}_c^{n,\phi}$, it is also true that for all $i \in \phi$, $g^i(s) < K_n^{|\phi|} \eta_n$. Define $\tilde{\phi} = \phi \cup \{j\}$. As $|\tilde{\phi}| = |\phi| + 1$, it now follows that $g^k(s) < K_n^{|\tilde{\phi}|-1} \eta_n$ for all $k \in \tilde{\phi}$. By the definition of the $\beta-$inflated boundary for $\mathcal{A}_c^k$, it now follows that $s \in \mathcal{A}_c^{K_n^{(|\tilde{\phi}|-1)} \eta_n, k}$ for all $k \in \tilde{\phi}$. However, by the definition of $\mathcal{I}_c^{n,\tilde{\phi}}$, this implies that either $s \in \mathcal{I}_c^{n,\tilde{\phi}}$ or $s \in \mathcal{I}_c^{n,\tilde{\phi}'}$ for some $\tilde{\phi}'$ such that $\text{Ord}(\tilde{\phi}') > \text{Ord}(\tilde{\phi})$. In either case, $s \in \mathcal{I}_c^{n,\phi'}$ for some ϕ' such that $\text{Ord}(\phi') > \text{Ord}(\phi)$. From the definition of $\mathcal{I}_c^{n,\phi}$, it follows that $s \notin \mathcal{I}_c^{n,\phi}$. This is a contradiction, as $s \in \mathcal{I}_c^{n,\phi}$ by construction. The result of the lemma now follows. □

Lemma S7. *Define the modulus of continuity, ω, of a function, f, and a distance, δ, by:*

$$\omega(f, \delta) = \sup_{s_1, s_2 \in S : |s_1 - s_2| < \delta} |f(s_1) - f(s_2)|.$$

If $\{f_n\}_{n \in \mathbb{N}}$ is a sequence of continuous random variables on S which converges in distribution to a function f which has continuous sample paths, we have that:

$$\forall \epsilon > 0, \lim_{\delta \to 0} \limsup_{n \to \infty} \mathbb{P}[\omega(f_n, \delta) \geq \epsilon] = 0.$$

Proof. See Theorem 3.3.1 of Khoshnevisan [2002]. □

S4.4 Theorem S2: The Events Implication Theorem

Theorem S2. *Defining the events* $\{\mathcal{E}_{\mathcal{I}}^{\phi,n}\}_{\phi\in\mathcal{P}(\mathcal{M})}$ *and* $\{\mathcal{E}_{\mathcal{O}}^{\phi,n}\}_{\phi\in\mathcal{P}(\mathcal{M})}$ *as in Section S4.1, the following implication holds:*

$$\bigcap_{\phi\in\mathcal{P}(\mathcal{M})}\{\mathcal{E}_{\mathcal{I}}^{\phi,n}\wedge\mathcal{E}_{\mathcal{O}}^{\phi,n}\} \implies \{\hat{\mathcal{F}}_c^{+}\subseteq\mathcal{F}_c\subseteq\hat{\mathcal{F}}_c^{-}\}.$$

Proof. We shall first show that if the events $\{\mathcal{E}_{\mathcal{I}}^{\phi,n}\wedge\mathcal{E}_{\mathcal{O}}^{\phi,n}\}_{\phi\in\mathcal{P}(\mathcal{M})}$ occur then $\{\mathcal{F}_c\subseteq\hat{\mathcal{F}}_c^{-}\}$ holds. In other words, we will show that, under the assumption that the events occur, if $s\in\mathcal{F}_c$, then $s\in\hat{\mathcal{F}}_c^{-}$. As $\mathcal{F}_c=\cup_{\phi\in\mathcal{P}(\mathcal{M})}\mathcal{I}_c^{n,\phi}$, we split the statement $s\in\mathcal{F}_c$ into two cases; $s\in\mathcal{I}_c^{n,\emptyset}$ (Case 1), and $s\in\mathcal{I}_c^{n,\phi}$ for some $\phi\in\mathcal{P}^{+}(\mathcal{M})$ (Case 2).

Case 1:

Let $s\in\mathcal{I}_c^{n,\emptyset}$. As $\mathcal{I}_c^{n,\emptyset}\subseteq\mathcal{F}_c$, it follows that $s\in\mathcal{F}_c$. Now, suppose that for some $i\in\mathcal{M}$, $|g^i(s)|\leq\eta_n$. This would mean that $s\in\mathcal{A}_c^{\eta_n,i}$ by the definition of $\mathcal{A}_c^{\eta_n,i}$. By considering the definition of $\mathcal{I}_c^{n,\{i\}}$, it can be seen that this implies that either $s\in\mathcal{I}_c^{n,\{i\}}$ or $s\in\mathcal{I}_c^{n,\phi'}$ for some $\phi'\in\mathcal{P}^{+}(\mathcal{M})$ such that $\text{Ord}(\phi')>\text{Ord}(\{i\})$. However, this is a contradiction, as $s\in\mathcal{I}_c^{n,\emptyset}$ which is disjoint from the sets $\{\mathcal{I}_c^{n,\phi}\}_{\phi\in\mathcal{P}^{+}(\mathcal{M})}$. Therefore, it follows that for all $i\in\mathcal{M}$, $|g^i(s)|>\eta_n$.

As $s\in\mathcal{F}_c$, $g^i(s)\geq 0$ for all $i\in\mathcal{M}$. Therefore, for all $i\in\mathcal{M}$, $g^i(s)>\eta_n$. Consequently, for all $i\in\mathcal{M}$, $\eta_n-g^i(s)<0$ and:

$$\tau_n^{-1}\min_{i\in\mathcal{M}}(\hat{g}_n^i(s))>\tau_n^{-1}\min_{i\in\mathcal{M}}\left(\hat{g}_n^i(s)+\eta_n-g^i(s)\right)$$

$$=\tau_n^{-1}\min_{i\in\mathcal{M}}\left(\hat{g}_n^i(s)-g^i(s)\right)+\tau_n^{-1}\eta_n\geq-\tau_n^{-1}\max_{i\in\mathcal{M}}\left|\hat{g}_n^i(s)-g^i(s)\right|+\tau_n^{-1}\eta_n$$

$$\geq -a-\tau_n^{-1}\eta_n+\tau_n^{-1}\eta_n=-a,$$

where the second inequality follows from basic properties of 'min' and 'max' and the third from the definition of $\mathcal{E}_{\mathcal{I}}^{\emptyset,n}$. By the definition of $\hat{\mathcal{F}}_c^{-}$, it follows that $s\in\hat{\mathcal{F}}_c^{-}$ as required.

Therefore, if the events $\{\mathcal{E}_{\mathcal{I}}^{\phi,n}\}_{\phi\in\mathcal{P}(\mathcal{M})}$ and $\{\mathcal{E}_{\mathcal{O}}^{\phi,n}\}_{\phi\in\mathcal{P}(\mathcal{M})}$ occur then $\mathcal{I}_c^{n,\emptyset} \subseteq \hat{\mathcal{F}}_c^-$. In other words;

$$\bigcap_{\phi\in\mathcal{P}(\mathcal{M})} \{\mathcal{E}_{\mathcal{I}}^{\phi,n} \wedge \mathcal{E}_{\mathcal{O}}^{\phi,n}\} \implies \{\mathcal{I}_c^{n,\emptyset} \subseteq \hat{\mathcal{F}}_c^-\}.$$

Case 2:

Let $s \in \mathcal{I}_c^{n,\phi}$ for some $\phi \in \mathcal{P}^+(\mathcal{M})$. Again, as $s \in \mathcal{F}_c$ it follows that $g^i(s) \geq 0$ for all $i \in \phi$. It therefore follows that;

$$\tau_n^{-1} \min_{i\in\mathcal{M}}(\hat{g}_n^i(s)) = \tau_n^{-1} \min\left(\min_{i\in\phi}(\hat{g}_n^i(s)), \min_{j\in\mathcal{M}\setminus\phi}(\hat{g}_n^j(s))\right)$$

$$\geq \tau_n^{-1} \min\left(\min_{i\in\phi}(\hat{g}_n^i(s) - g^i(s)), \min_{j\in\mathcal{M}\setminus\phi}(\hat{g}_n^j(s))\right) \geq -a,$$

where the last inequality follows from the definition of $\mathcal{E}_{\mathcal{I}}^{\phi,n}$. Using similar logic to that employed in case 1, it now follows that if $\{\mathcal{E}_{\mathcal{I}}^{\phi,n}\}_{\phi\in\mathcal{P}(\mathcal{M})}$ and $\{\mathcal{E}_{\mathcal{O}}^{\phi,n}\}_{\phi\in\mathcal{P}(\mathcal{M})}$ occur then, for any $\phi \in \mathcal{P}^+(\mathcal{M})$, it is true that $\mathcal{I}_c^{n,\phi} \subseteq \hat{\mathcal{F}}_c^-$. And therefore, for all $\phi \in \mathcal{P}^+(\mathcal{M})$;

$$\bigcap_{\phi\in\mathcal{P}(\mathcal{M})} \{\mathcal{E}_{\mathcal{I}}^{\phi,n} \wedge \mathcal{E}_{\mathcal{O}}^{\phi,n}\} \implies \{\mathcal{I}_c^{n,\phi} \subseteq \hat{\mathcal{F}}_c^-\}.$$

By combining the results of both cases, it can now be seen that:

$$\bigcap_{\phi\in\mathcal{P}(\mathcal{M})} \{\mathcal{E}_{\mathcal{I}}^{\phi,n} \wedge \mathcal{E}_{\mathcal{O}}^{\phi,n}\} \implies \{\mathcal{F}_c \subseteq \hat{\mathcal{F}}_c^-\}. \tag{S8}$$

We shall now show that if $\{\mathcal{E}_{\mathcal{I}}^{\phi,n}\}_{\phi\in\mathcal{P}(\mathcal{M})}$ and $\{\mathcal{E}_{\mathcal{O}}^{\phi,n}\}_{\phi\in\mathcal{P}(\mathcal{M})}$ occur then it follows that $\hat{\mathcal{F}}_c^+ \subseteq \mathcal{F}_c$. To do this, we shall show that, if the events hold and $s \in (\mathcal{F}_c)^c$ then $s \in (\hat{\mathcal{F}}_c^+)^c$. We begin by noting that $(\mathcal{F}_c)^c = \cup_{\phi\in\mathcal{P}(\mathcal{M})}\mathcal{O}_c^{n,\phi}$ and again split the statement $s \in (\mathcal{F}_c)^c$ into two cases; $s \in \mathcal{O}_c^{n,\emptyset}$ (Case 1), and $s \in \mathcal{O}_c^{n,\phi}$ for some $\phi \in \mathcal{P}^+(\mathcal{M})$ (Case 2).

Case 1:

Let $s \in \mathcal{O}_c^{n,\emptyset}$. As $\mathcal{O}_c^{n,\emptyset} \subseteq (\mathcal{F}_c)^c$, it follows that $\min_{i\in\mathcal{M}} g^i(s) < 0$. Furthermore, by the definition of $\mathcal{F}_c^n$ and the fact that $\mathcal{O}_c^{n,\emptyset}$ and $\mathcal{F}_c^n$ are disjoint, it follows that $|\min_{i\in\mathcal{M}} g^i(s)| > \eta_n$. Combining these facts we have that $\min_{i\in\mathcal{M}} g^i(s) < -\eta_n$ and therefore $-\eta_n - \min_{i\in\mathcal{M}} g^i(s) > 0$. It now follows that:

$$\tau_n^{-1} \min_{i\in\mathcal{M}} \hat{g}_n^i(s) < \tau_n^{-1}\left(\min_{i\in\mathcal{M}} \hat{g}_n^i(s) - \min_{i\in\mathcal{M}} g^i(s)\right) - \tau_n^{-1}\eta_n$$

$$\leq \tau_n^{-1} \max_{i \in \mathcal{M}} \left(\hat{g}_n^i(s) - g^i(s) \right) - \tau_n^{-1}\eta_n \leq a + \tau_n^{-1}\eta_n - \tau_n^{-1}\eta_n = a,$$

where the second inequality follows from Lemma S2 and the third follows from the definition of $\mathcal{E}_{\mathcal{O}}^{\emptyset,n}$. From the definition of $\hat{\mathcal{F}}_c^+$, it follows that $s \in (\hat{\mathcal{F}}_c^+)^c$ as required. Therefore, if $\{\mathcal{E}_{\mathcal{I}}^{\phi,n}\}_{\phi \in \mathcal{P}(\mathcal{M})}$ and $\{\mathcal{E}_{\mathcal{O}}^{\phi,n}\}_{\phi \in \mathcal{P}(\mathcal{M})}$ occur then $\mathcal{O}_c^{n,\emptyset} \subseteq (\hat{\mathcal{F}}_c^+)^c$. In other words, the below holds:

$$\bigcap_{\phi \in \mathcal{P}(\mathcal{M})} \{\mathcal{E}_{\mathcal{I}}^{\phi,n} \wedge \mathcal{E}_{\mathcal{O}}^{\phi,n}\} \implies \{\mathcal{O}_c^{n,\emptyset} \subseteq (\hat{\mathcal{F}}_c^+)^c\}.$$

<u>Case 2:</u>

Let $s \in \mathcal{O}_c^{n,\phi}$ for some $\phi \in \mathcal{P}^+(\mathcal{M})$. By the definition of $\mathcal{O}_c^{n,\phi}$ it follows that $g^i(s) < 0$ for all $i \in \phi$. Therefore, it follows that:

$$\tau_n^{-1} \min_{i \in \mathcal{M}} \hat{g}_n^i(s) \leq \tau_n^{-1} \min_{i \in \phi} \hat{g}_n^i(s) < \tau_n^{-1} \min_{i \in \phi} \left(\hat{g}_n^i(s) - g^i(s) \right) \leq a,$$

where the first inequality follows as $\phi \subseteq \mathcal{M}$ and the third follows from the definition of $\mathcal{E}_{\mathcal{O}}^{\phi,n}$ for $\phi \in \mathcal{P}^+(\mathcal{M})$. From the definition of $\hat{\mathcal{F}}_c^+$, it now follows that $s \in (\hat{\mathcal{F}}_c^+)^c$. By using similar logic to that employed in case 1, it can now be seen that:

$$\bigcap_{\phi \in \mathcal{P}(\mathcal{M})} \{\mathcal{E}_{\mathcal{I}}^{\phi,n} \wedge \mathcal{E}_{\mathcal{O}}^{\phi,n}\} \implies \{\mathcal{O}_c^{n,\phi} \subseteq (\hat{\mathcal{F}}_c^+)^c\}.$$

By combining this with the result of case 1, it can be seen that:

$$\bigcap_{\phi \in \mathcal{P}(\mathcal{M})} \{\mathcal{E}_{\mathcal{I}}^{\phi,n} \wedge \mathcal{E}_{\mathcal{O}}^{\phi,n}\} \implies \{(\mathcal{F}_c)^c \subseteq (\hat{\mathcal{F}}_c^+)^c\} \equiv \{\hat{\mathcal{F}}_c^+ \subseteq \mathcal{F}_c\}, \tag{S9}$$

as required. The result of Theorem S2 now follows directly from Statements (S8) and (S9).

□

S4.5 Theorem S3: Convergence of $\max(A^n_\phi) - \max(B^n_\phi)$

Theorem S3. *Defining A^n_ϕ and B^n_ϕ as in Section S4.1, it is true that:*

$$\max_{\phi \in \mathcal{P}^+(\mathcal{M})}(A^n_\phi) - \max_{\phi \in \mathcal{P}^+(\mathcal{M})}(B^n_\phi) \xrightarrow{p} 0.$$

Proof. By Lemma S5, it suffices to show that, for arbitrary $\phi \in \mathcal{P}^+(\mathcal{M})$, $A^n_\phi - B^n_\phi \xrightarrow{p} 0$. Further, by Lemma S3, it suffices to show that $A^{n,\mathcal{I}}_\phi - B^{n,\mathcal{I}}_\phi \xrightarrow{p} 0$. From the definitions of $A^{n,\mathcal{I}}_\phi$ and $B^{n,\mathcal{I}}_\phi$ it follows that if $\phi = \mathcal{M}$ then $A^{n,\mathcal{I}}_\phi = B^{n,\mathcal{I}}_\phi$ for all $n \in \mathbb{N}$ and the convergence trivially holds. To prove the convergence for $\phi \neq \mathcal{M}$, we employ Lemma S4 to see that:

$$|A^{n,\mathcal{I}}_\phi - B^{n,\mathcal{I}}_\phi| \leq \tau_n^{-1} \sup_{s \in \mathcal{I}^{n,\phi}_c} \left| \min\left(\min_{i\in\phi}(\hat{g}^i_n(s) - g^i(s)), \min_{j \in \mathcal{M}\setminus\phi}(\hat{g}^j_n(s))\right) - \min_{i\in\phi}(\hat{g}^i_n(s) - g^i(s)) \right|$$

$$= \tau_n^{-1} \sup_{s \in \mathcal{I}^{n,\phi}_c} \left| \min\left(0, \min_{j \in \mathcal{M}\setminus\phi}(\hat{g}^j_n(s)) - \min_{i\in\phi}(\hat{g}^i_n(s) - g^i(s))\right) \right|.$$

Therefore, for arbitrary $\epsilon > 0$, it follows that:

$$\mathbb{P}\left[|A^{n,\mathcal{I}}_\phi - B^{n,\mathcal{I}}_\phi| \geq \epsilon\right] \leq \mathbb{P}\left[\tau_n^{-1} \sup_{s \in \mathcal{I}^{n,\phi}_c} \left| \min\left(0, \min_{j \in \mathcal{M}\setminus\phi}(\hat{g}^j_n(s)) - \min_{i\in\phi}(\hat{g}^i_n(s) - g^i(s))\right) \right| \geq \epsilon\right]$$

$$= \mathbb{P}\left[\exists s \in \mathcal{I}^{n,\phi}_c \text{ such that } \tau_n^{-1}\left(\min_{j \in \mathcal{M}\setminus\phi}(\hat{g}^j_n(s)) - \min_{i\in\phi}(\hat{g}^i_n(s) - g^i(s))\right) \leq -\epsilon\right]. \tag{S10}$$

Now, suppose that an $s \in \mathcal{I}^{n,\phi}_c$ exists which satisfies the inequality appearing inside the above probability. This would trivially imply that;

$$\tau_n^{-1}\left(\min_{j \in \mathcal{M}\setminus\phi}(\hat{g}^j_n(s)) - \min_{j \in \mathcal{M}\setminus\phi}(g^j(s)) - \min_{i\in\phi}(\hat{g}^i_n(s) - g^i(s))\right) \leq -\epsilon - \tau_n^{-1} \min_{j \in \mathcal{M}\setminus\phi}(g^j(s)).$$

However, by Lemma S2, it can be seen that the above implies:

$$\tau_n^{-1}\left(\min_{j \in \mathcal{M}\setminus\phi}(\hat{g}^j_n(s) - g^j(s)) - \min_{i\in\phi}(\hat{g}^i_n(s) - g^i(s))\right) \leq -\epsilon - \tau_n^{-1} \min_{j \in \mathcal{M}\setminus\phi}(g^j(s)).$$

We now note that by Lemma S6, it follows that for all $s \in \mathcal{I}_c^{n,\phi}$ and $j \in \mathcal{M} \setminus \phi$ it is true that $g^j(s) \geq K_n^{|\phi|}\eta_n$. Therefore, the above now implies that;

$$\tau_n^{-1}\left(\min_{j\in\mathcal{M}\setminus\phi}(\hat{g}_n^j(s) - g^j(s)) - \min_{i\in\phi}(\hat{g}_n^i(s) - g^i(s))\right) \leq -\epsilon - \tau_n^{-1}K_n^{|\phi|}\eta_n.$$

Therefore, Equation (S10) must be less than or equal to the following:

$$\mathbb{P}\left[\exists s \in \mathcal{I}_c^{n,\phi} \text{ such that } \tau_n^{-1}\left(\min_{j\in\mathcal{M}\setminus\phi}(\hat{g}_n^j(s) - g^j(s)) - \min_{i\in\phi}(\hat{g}_n^i(s) - g^i(s))\right) \leq -\epsilon - \tau_n^{-1}K_n^{|\phi|}\eta_n\right]$$

$$= \mathbb{P}\left[\tau_n^{-1}\inf_{s\in\mathcal{I}_c^{n,\phi}}\left(\min_{j\in\mathcal{M}\setminus\phi}(\hat{g}_n^j(s) - g^j(s)) - \min_{i\in\phi}(\hat{g}_n^i(s) - g^i(s))\right) \leq -\epsilon - \tau_n^{-1}K_n^{|\phi|}\eta_n\right].$$

The left hand side of the expression inside the above probability tends in distribution to a well defined random variable by Assumption 2.2.1. However, the right hand side converges to $-\infty$ by the definition of K_n. Therefore, the above probability converges to 0. It now follows that, for arbitrary $\epsilon > 0$, $\mathbb{P}[|A_\phi^{n,\mathcal{I}} - B_\phi^{n,\mathcal{I}}| \geq \epsilon] \to 0$ as $n \to \infty$. Therefore, $A_\phi^{n,\mathcal{I}} - B_\phi^{n,\mathcal{I}} \xrightarrow{p} 0$ as required. □

S4.6 Theorem S4: Convergence of $\max(B_\phi^n) - \max(C_\phi^n)$

Theorem S4. *Defining B_ϕ^n and C_ϕ^n as in Section S4.1, it is true that:*

$$\max_{\phi\in\mathcal{P}^+(\mathcal{M})}(B_\phi^n) - \max_{\phi\in\mathcal{P}^+(\mathcal{M})}(C_\phi^n) \xrightarrow{p} 0.$$

Proof. By Lemma S5, it suffices to show the following two statements:

$$\max_{\phi\in\mathcal{P}^+(\mathcal{M})}(B_\phi^{n,\mathcal{I}}) - \max_{\phi\in\mathcal{P}^+(\mathcal{M})}(C_\phi^n) \xrightarrow{p} 0, \tag{S11}$$

$$\max_{\phi\in\mathcal{P}^+(\mathcal{M})}(B_\phi^{n,\mathcal{O}}) - \max_{\phi\in\mathcal{P}^+(\mathcal{M})}(C_\phi^n) \xrightarrow{p} 0. \tag{S12}$$

To prove each statement we shall employ arguments based on convergence in Hausdorff distance. However, these arguments are complicated by the fact that the sets $\mathcal{I}_c^{n,\phi}$ and $\mathcal{O}_c^{n,\phi}$ may not converge in Hausdorff distance to $\partial^\phi\mathcal{F}_c$ (or, in the case of $\mathcal{I}_c^{n,\phi}$, converge at all). To understand why, define $\mathcal{D}_\phi$ as;

$$\mathcal{D}_\phi = \Bigg(\bigcup_{\substack{\phi_1\in\mathcal{P}^+(\mathcal{M})\\ \phi_1\subset\phi}} \overline{\partial^{\phi_1}\mathcal{F}_c}\Bigg) \cap \Bigg(\bigcup_{\substack{\phi_2\in\mathcal{P}^+(\mathcal{M})\\ \phi_2\supset\phi}} \overline{\partial^{\phi_2}\mathcal{F}_c}\Bigg).$$

$\mathcal{D}_\phi$ can be perceived as a set of "difficult" points, at which a section of $\partial^\phi\mathcal{F}_c$ has shrunk to a point and essentially 'disappeared' (i.e. is no longer considered part of $\partial^\phi\mathcal{F}_c$, see Fig. S7).

Proof of Statement (S11):

Due to the resemblance between $\mathcal{D}_\phi$ and $\partial^\phi\mathcal{F}_c$, $\mathcal{I}_c^{n,\phi}$ may converge in Hausdorff distance to a set containing a point in $\mathcal{D}_\phi$ or to a set disjoint from $\mathcal{D}_\phi$. It is also possible that $\mathcal{I}_c^{n,\phi}$ converges to neither option but instead oscillates infinitely between the two, thus not converging at all. To handle this issue define $\tilde{\epsilon}_\phi(s) = d(s, \partial^\phi\mathcal{F}_c)$ (where $d(x, A)$ is the minimum distance from the point x to the set A) and $T_\delta(\mathcal{D}_\phi)$ as:

$$T_\delta(\mathcal{D}_\phi) = \bigcup_{s\in\mathcal{D}_\phi} B_{\min(\delta,\tilde{\epsilon}_\phi(s))}(s),$$

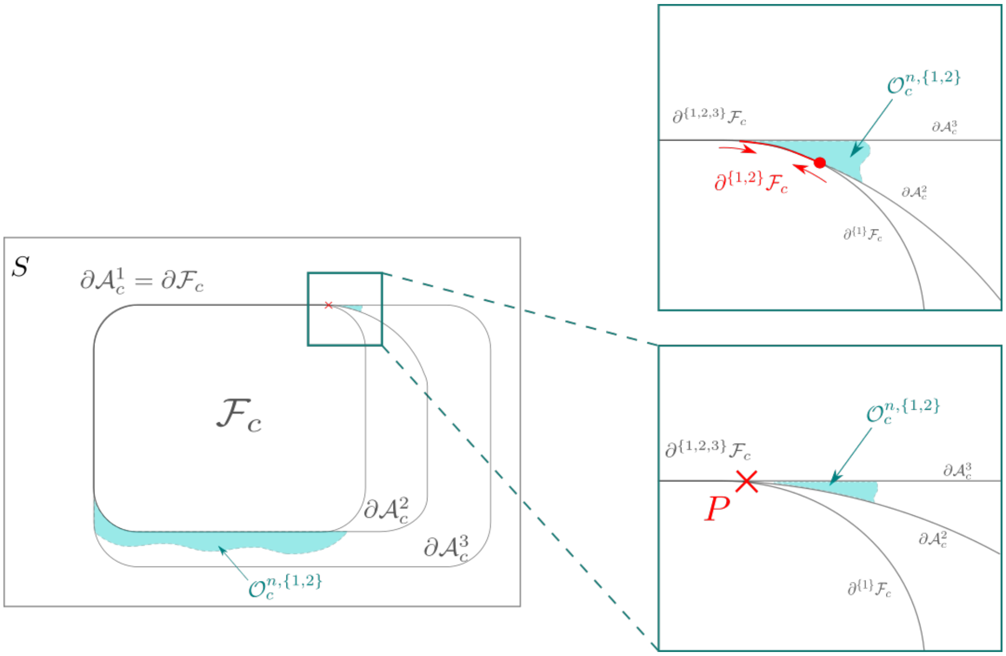

Figure S7: An example in which $\mathcal{O}_c^{n,\{1,2\}}$ may converge to $\partial^{\{1,2\}}\mathcal{F}_c \cup P$ where $P \in \mathcal{D}_\phi$. In this instance, it is possible for the set $\mathcal{O}_c^{n,\{1,2\}}$ to border the point P (see bottom right) despite P being located far from the boundary $\partial^{\{1,2\}}\mathcal{F}_c$. This situation has arisen as P strongly resembles a segment of $\partial^{\{1,2\}}\mathcal{F}_c$ which has become vanishingly small (see top right).

where $B_\epsilon(s)$ is the open ϵ-ball about s. To illustrate the role of $T_\delta(\mathcal{D}_\phi)$, consider an example in 3 dimensions. In this example, each boundary segment $\partial^\phi\mathcal{F}_c$ may appear as a plane and $\mathcal{D}_\phi$ consists of regions at which two planes intersect. Suppose that $\mathcal{D}_\phi$ is a line segment. In this case, $T_\delta(\mathcal{D}_\phi)$ is an open tube of width δ around $\mathcal{D}_\phi$ which tapers to a point whenever one of the tubes endpoints gets close to $\partial^\phi\mathcal{F}_c$. Fix $\delta > 0$ and define:

$$L_\phi^n = \sup_{s\in\mathcal{I}_c^{n,\phi}\setminus T_\delta(\mathcal{D}_\phi)} \left|\tau_n^{-1}\min_{i\in\phi}(\hat{g}_n^i(s) - g^i(s))\right|, \qquad U_\phi^n = \sup_{s\in\mathcal{I}_c^{n,\phi}\cup\mathcal{D}_\phi} \left|\tau_n^{-1}\min_{i\in\phi}(\hat{g}_n^i(s) - g^i(s))\right|.$$

L_ϕ^n and U_ϕ^n are lower and upper bounds for $B_\phi^{n,\mathcal{I}}$ (i.e. $L_\phi^n \leq B_\phi^{n,\mathcal{I}} \leq U_\phi^n$). Therefore, in order to show Statement (S11), it suffices to show that $\max_{\phi\in\mathcal{P}^+(\mathcal{M})}(L_\phi^n) - \max_{\phi\in\mathcal{P}^+(\mathcal{M})}(C_\phi^n) \xrightarrow{p} 0$ and $\max_{\phi\in\mathcal{P}^+(\mathcal{M})}(U_\phi^n) - \max_{\phi\in\mathcal{P}^+(\mathcal{M})}(C_\phi^n) \xrightarrow{p} 0$. To prove convergence for the lower bound we note that, by the definitions of L_ϕ^n and C_ϕ^n:

$$|L_\phi^n - C_\phi^n| = \left| \sup_{s\in\mathcal{I}_c^{n,\phi}\setminus T_\delta(\mathcal{D}_\phi)} \left|\tau_n^{-1}\min_{i\in\phi}(\hat{g}_n^i(s) - g^i(s))\right| - \sup_{s\in\partial^\phi\mathcal{F}_c} \left|\tau_n^{-1}\min_{i\in\phi}(\hat{g}_n^i(s) - g^i(s))\right| \right|$$

$$\leq \omega\left(|\tau_n^{-1}\min_{i\in\phi}(\hat{g}_n^i(s)-g^i(s))|, \delta_n^{\phi,L}\right),$$

where $\delta_n^{\phi,L}$ is the Hausdorff distance between $\mathcal{I}_c^{n,\phi}\setminus T_\delta(\mathcal{D}_\phi)$ and $\partial^\phi\mathcal{F}_c$, and ω is the modulus of continuity (c.f. Lemma S7). By Lemma S7, it now follows that, for any $\epsilon>0$;

$$\lim_{n\to\infty}\mathbb{P}[|L_\phi^n - C_\phi^n|\geq\epsilon]\leq\limsup_{n\to\infty}\mathbb{P}\left[\omega\left(|\tau_n^{-1}\min_{i\in\phi}(\hat{g}_n^i(s)-g^i(s))|, \delta_n^{\phi,L}\right)\geq\epsilon\right]=0,$$

if $\delta_n^{\phi,L}\to 0$ as $n\to\infty$. Therefore, if $\delta_n^{\phi,L}\to 0$ as $n\to\infty$, the convergence of the lower bound holds. We now must show that $\delta_n^{\phi,L}\to 0$ as $n\to\infty$. As S is compact, by standard properties of the Hausdorff distance, it suffices to show that:

$$\limsup_{n\to\infty}\left(\mathcal{I}_c^{n,\phi}\setminus T_\delta(\mathcal{D}_\phi)\right)=\liminf_{n\to\infty}\left(\mathcal{I}_c^{n,\phi}\setminus T_\delta(\mathcal{D}_\phi)\right)=\overline{\partial^\phi\mathcal{F}_c}.$$

To do so, first fix $t^*\in\partial^\phi\mathcal{F}_c$. Suppose that $t^*\in\mathcal{I}_c^{n,\phi'}$ for some $\phi'\in\mathcal{P}^+(\mathcal{M})$ such that $\text{Ord}(\phi')>\text{Ord}(\phi)$. By the definition of Ord, it follows that $\phi'\cap(\mathcal{M}\setminus\phi)$ is non-empty. Fix $j\in\phi'\cap(\mathcal{M}\setminus\phi)$. As $K_n^{|\phi'|-1}\eta_n\to 0$ as $n\to\infty$ and $g^j(t^*)>0$ (by the definition of $\partial^\phi\mathcal{F}_c$), it follows that, for n large enough, $g^j(t^*)>K_n^{|\phi'|-1}\eta_n$. Therefore, for n large enough, $t^*\not\in\mathcal{A}_c^{K_n^{|\phi'|-1}\eta_n,j}$. However, this contradicts the definition of $\mathcal{I}_c^{n,\phi'}$. It therefore follows, that for n large enough, $t^*\not\in\mathcal{I}_c^{n,\phi'}$ for any $\phi'\in\mathcal{P}^+(\mathcal{M})$ such that $\text{Ord}(\phi')>\text{Ord}(\phi)$.

By the definition of $\partial^\phi\mathcal{F}_c$, $t^*\in\mathcal{F}_c$ and $t^*\in\mathcal{A}_c^{(K_n)^{|\phi|-1}\eta_n,i}$ for all $i\in\phi$. By the definition of $T_\delta(\mathcal{D}_\phi)$, $t^*\not\in T_\delta(\mathcal{D}_\phi)$. Combining these observations, for n large enough:

$$t^*\in\mathcal{F}_c\cap\left(\bigcap_{i\in\phi}\mathcal{A}_c^{(K_n)^{|\phi|-1}\eta_n,i}\right)\setminus\left(T_\delta(\mathcal{D}_\phi)\cup\bigcup_{\substack{\phi'\in\mathcal{P}^+(\mathcal{M})\\ \text{Ord}(\phi')>\text{Ord}(\phi)}}\mathcal{I}_c^{n,\phi'}\right)=\mathcal{I}_c^{n,\phi}\setminus T_\delta(\mathcal{D}_\phi).$$

Therefore, for every $t^*\in\partial^\phi\mathcal{F}_c$, if n is large enough $t^*\in\mathcal{I}_c^{n,\phi}\setminus T_\delta(\mathcal{D}_\phi)$. It now follows that;

$$\overline{\partial^\phi\mathcal{F}_c}\subseteq\liminf_{n\to\infty}\left(\mathcal{I}_c^{n,\phi}\setminus T_\delta(\mathcal{D}_\phi)\right).$$

Now, fix $s^*\in\limsup_{n\to\infty}\left(\mathcal{I}_c^{n,\phi}\setminus T_\delta(\mathcal{D}_\phi)\right)$. It follows that there exists a sequence of points $\{s_{n_m}\}_{m\in\mathbb{N}}$ such that $s_{n_m}\in\mathcal{I}_c^{n_m,\phi}\setminus T_\delta(\mathcal{D}_\phi)$ and $s_{n_m}\to s^*$ as $m\to\infty$. As $s_{n_m}\in\mathcal{I}_c^{n_m,\phi}$, it follows that for each $m\in\mathbb{N}$, s_{n_m} belongs in either $(\mathcal{F}_c)^\circ$ or $\partial^{\tilde{\phi}}\mathcal{F}_c$ for some $\tilde{\phi}\subseteq\phi$ (c.f. Lemma S6). For ease, we shall now assume that the points $\{s_{n_m}\}_{m\in\mathbb{N}}$ lie only in $(\mathcal{F}_c)^\circ$ and not in

$\partial^{\tilde{\phi}}\mathcal{F}_c$ for any $\tilde{\phi} \subseteq \phi$ and return to the latter possibility later in the proof. As $s_{n_m} \in \mathcal{I}_c^{n_m,\phi}$, it follows that for all $i \in \phi$, $g^i(s_{n_m}) < (K_{n_m})^{|\phi|-1}\eta_{n_m}$. Therefore, for all $i \in \phi$;

$$0 \leq g^i(s^*) = \lim_{m\to\infty} g^i(s_{n_m}) \leq \lim_{m\to\infty} (K_{n_m})^{|\phi|-1}\eta_{n_m} = 0.$$

And therefore, $g^i(s^*) = 0$ for all $i \in \phi$. This implies that $s^* \in \partial^{\phi_1}\mathcal{F}_c$ for some $\phi_1 \supseteq \phi$. Now suppose that $s^* \notin \overline{\partial^{\phi}\mathcal{F}_c}$. This would imply that $s^* \in \cup_{\phi_1 \supset \phi}\partial^{\phi_1}\mathcal{F}_c \setminus \overline{\partial^{\phi}\mathcal{F}_c}$.

Suppose further that $s^* \in \mathcal{D}_\phi$. As for all $m \in \mathbb{N}$, $s_{n_m} \in \mathcal{I}_c^{n_m,\phi} \setminus T_\delta(\mathcal{D}_\phi)$, it follows that $s_{n_m} \notin T_\delta(\mathcal{D}_\phi)$. By the definition of $T_\delta(\mathcal{D}_\phi)$, this means that, for all $m \in \mathbb{N}$, $s_{n_m} \notin B_\epsilon(s^*)$ for all $\epsilon < \min(\delta, \tilde{\epsilon}_\phi(s^*))$. This is a contradiction as $s_{n_m} \to s^*$. Consequently $s^* \notin \mathcal{D}_\phi$. Thus;

$$s^* \in \bigcup_{\phi_1 \supset \phi} \partial^{\phi_1}\mathcal{F}_c \setminus \left(\overline{\partial^{\phi}\mathcal{F}_c} \cup \mathcal{D}_\phi\right) = \bigcup_{\phi_1 \supset \phi} \partial^{\phi_1}\mathcal{F}_c \setminus \bigcup_{\phi_2 \subseteq \phi} \overline{\partial^{\phi_2}\mathcal{F}_c},$$

where the equality follows from the definition of $\mathcal{D}_\phi$. Now, by the definition of $\mathcal{I}_c^{n,\phi}$ and Lemma S6, it follows that for any $i \in \phi$ and $j \in \mathcal{M} \setminus \phi$, $0 < g^i(s_{n_m}) \leq (K_{n_m})^{|\phi|-1}\eta_{n_m}$ and $g^j(s_{n_m}) \geq (K_{n_m})^{|\phi|}\eta_{n_m}$. It follows that, for any $i \in \phi$ and $j \in \mathcal{M}\setminus\phi$, $K_{m_n} \leq g^j(s_{n_m})/g^i(s_{n_m})$. And, therefore;

$$a_{n_m} := \min_{\substack{i\in\phi \\ j\in\mathcal{M}\setminus\phi}} \left(\frac{g^j(s_{n_m})}{g^i(s_{n_m})}\right) \geq K_{n_m}.$$

As $K_{n_m} \to \infty$ as $m \to \infty$, it follows that $\{a_n\}_{n\in\mathbb{N}}$ is unbounded. However, this contradicts Theorem S5 which states that $\{a_n\}_{n\in\mathbb{N}}$ is bounded. Therefore, it cannot be the case that $s^* \in \limsup_{n\to\infty}\left(\mathcal{I}_c^{n,\phi} \setminus T_\delta(\mathcal{D}_\phi)\right)$ unless $s^* \in \overline{\partial^{\phi}\mathcal{F}_c}$. It therefore follows that;

$$\limsup_{n\to\infty}\left(\mathcal{I}_c^{n,\phi} \setminus T_\delta(\mathcal{D}_\phi)\right) \subseteq \overline{\partial^{\phi}\mathcal{F}_c}.$$

We now return to our earlier assumption, in which we discounted sequences $\{s_{n_m}\}_{m\in\mathbb{N}}$ which lay inside $\partial^{\tilde{\phi}}\mathcal{F}_c$ for some $\tilde{\phi} \subseteq \phi$ from consideration. Trivially, it is true that if infinitely many $\{s_{n_m}\}_{m\in\mathbb{N}}$ belonged to $\partial^{\phi}\mathcal{F}_c$ the above result would still hold. To handle the cases in which infinitely many $\{s_{n_m}\}_{m\in\mathbb{N}}$ belong to $\partial^{\tilde{\phi}}\mathcal{F}_c$ for some $\tilde{\phi} \subset \phi$, the above argument may be employed with ϕ substituted for $\bar{\phi} = \phi \setminus \tilde{\phi}$ and $\mathcal{M}$ substituted for $\bar{\mathcal{M}} = \mathcal{M} \setminus \tilde{\phi}$ throughout.

We now have that the limit superior and inferior agree and therefore $\delta_n^{\phi,L} \to 0$ as $n \to \infty$ as desired. The convergence for the lower bound follows.

To adapt the proof to show convergence for the upper bound, we now define $\hat{C}^n_\phi$ as follows;

$$\hat{C}^n_\phi = \sup_{s \in \partial^\phi \mathcal{F}_c \cup \mathcal{D}_\phi} \left| \tau_n^{-1} \min_{i \in \phi} (\hat{g}^i_n(s) - g^i(s)) \right|.$$

By employing an identical argument to the above, but neglecting the steps of the proof which involved $T_\delta(\mathcal{D}_\phi)$, and noting that trivially $\overline{\mathcal{D}_\phi} \subseteq \liminf_{n\to\infty}(\mathcal{I}^{n,\phi}_c \cup \mathcal{D}_\phi)$, it can be seen that;

$$\liminf_{n\to\infty} \left(\mathcal{I}^{n,\phi}_c \cup \mathcal{D}_\phi\right) = \limsup_{n\to\infty} \left(\mathcal{I}^{n,\phi}_c \cup \mathcal{D}_\phi\right) = \overline{\partial^\phi \mathcal{F}_c} \cup \overline{\mathcal{D}}_\phi.$$

Therefore, $\mathcal{I}^{n,\phi}_c \cup \mathcal{D}_\phi$ converges to $\partial^\phi \mathcal{F}_c \cup \mathcal{D}_\phi$ in Hausdorff distance. By Lemma S7, it can be seen that the above implies that $U^n_\phi - \hat{C}^n_\phi \xrightarrow{p} 0$ for every $\phi \in \mathcal{P}^+(\mathcal{M})$. Finally, noting the result of Theorem S6, which states that $\max_{\phi\in\mathcal{P}^+(\mathcal{M})}(C^n_\phi) = \max_{\phi\in\mathcal{P}^+(\mathcal{M})}(\hat{C}^n_\phi)$, it can be seen that $\max_{\phi\in\mathcal{P}^+(\mathcal{M})}(U^n_\phi) - \max_{\phi\in\mathcal{P}^+(\mathcal{M})}(C^n_\phi) \xrightarrow{p} 0$.

As we have now shown convergence for both the lower and upper bounds, we can conclude that $\max_{\phi\in\mathcal{P}^+(\mathcal{M})}(B^{n,\mathcal{I}}_\phi) - \max_{\phi\in\mathcal{P}^+(\mathcal{M})}(C^n_\phi) \xrightarrow{p} 0$ as desired.

Proof of Statement (S12):

To begin, we claim that for any $\phi \in \mathcal{P}^+(\mathcal{M})$ there must exist a set $\tilde{\mathcal{D}}_\phi \subseteq \mathcal{D}_\phi$ such that $\delta^{\phi,\mathcal{O}}_n := d_H(\mathcal{O}^{n,\phi}_c, \partial^\phi \mathcal{F}_c \cup \tilde{\mathcal{D}}_\phi) \to 0$ as $n \to \infty$. Proof of this claim is provided by Theorem S7. Fix ϕ and $\tilde{\mathcal{D}}_\phi$, and define $\tilde{C}^n_\phi$ as:

$$\tilde{C}^n_\phi = \sup_{s \in \partial^\phi \mathcal{F}_c \cup \tilde{\mathcal{D}}_\phi} \left| \tau_n^{-1} \min_{i \in \phi} (\hat{g}^i_n(s) - g^i(s)) \right|.$$

By the definitions of $B^{n,\mathcal{O}}_\phi$ and $\tilde{C}^n_\phi$ we have that:

$$\begin{aligned} |B^{n,\mathcal{O}}_\phi - \tilde{C}^n_\phi| &= \left| \sup_{s \in \mathcal{O}^{n,\phi}_c} \left| \tau_n^{-1} \min_{i \in \phi} (\hat{g}^i_n(s) - g^i(s)) \right| - \sup_{s \in \partial^\phi \mathcal{F}_c \cup \tilde{\mathcal{D}}_\phi} \left| \tau_n^{-1} \min_{i \in \phi} (\hat{g}^i_n(s) - g^i(s)) \right| \right| \\ &\leq \omega\left(|\tau_n^{-1} \min_{i \in \phi} (\hat{g}^i_n(s) - g^i(s))|, \delta^{\phi,\mathcal{O}}_n \right). \end{aligned}$$

As $\delta^{\phi,\mathcal{O}}_n \to 0$ as $n \to \infty$, we can apply Lemma S7 to see that:

$$\forall \epsilon > 0, \mathbb{P}[|B^{n,\mathcal{O}}_\phi - \tilde{C}^n_\phi| \geq \epsilon] \leq \mathbb{P}\left[\omega\left(|\tau_n^{-1} \min_{i \in \phi} (\hat{g}^i_n(s) - g^i(s))|, \delta^{\phi,\mathcal{O}}_n \right) \geq \epsilon \right] \xrightarrow{n\to\infty} 0,$$

and therefore, for every $\phi \in \mathcal{P}^+(\mathcal{M})$, $B^{n,\mathcal{O}}_\phi - \tilde{C}^n_\phi \xrightarrow{p} 0$. Again, by employing Lemma S5 and the result of Theorem S6, this can now be seen to imply that Statement (S12) holds. □

S4.7 Theorem S5: Boundedness of $\{a_n\}_{n\in\mathbb{N}}$

Theorem S5. *If* $\phi \in \mathcal{P}^+(\mathcal{M})$ *and* $\{s_n\}_{n\in\mathbb{N}} \in (\mathcal{F}_c)^\circ$ *is a convergent sequence with limit* s^* *where;*

$$s^* \in \bigcup_{\phi_1 \supset \phi} \partial^{\phi_1}\mathcal{F}_c \setminus \bigcup_{\phi_2 \subseteq \phi} \overline{\partial^{\phi_2}\mathcal{F}_c},$$

then the sequence a_n*, defined in Section S4.6, is bounded above and below.*

Whilst its final form no longer bears a strong resemblance, we would like to acknowledge that the following proof drew heavy inspiration from the seminal proofs of Lawlor [2020].

Proof. For ease assume that $s^* \in \text{Int}(S)$. If $s^* \notin \text{Int}(S)$, an analagous argument holds when considering $s^* \in \partial S$ using the functions $\{g^i|_{\partial S}\}$. As $s^* \in \partial^{\phi_1}\mathcal{F}_c$ for some $\phi_1 \supset \phi$, we can choose $\tilde{i} \in \phi$ such that $g^{\tilde{i}}(s^*) = 0$. As $s^* \notin \overline{\partial^{\phi_2}\mathcal{F}_c}$ for any $\phi_2 \subseteq \phi$, we can define an open neighbourhood of s^*, $\mathcal{N}_0$, which does not intersect $\overline{\partial^{\phi_2}\mathcal{F}_c}$ for any $\phi_2 \subseteq \phi$. Further, by Assumptions 2.2.2 and 2.2.3, we can define an open neighbourhood $\mathcal{N}_1 \subseteq \mathcal{N}_0$ such that the functions $\{g^i\}_{i\in\mathcal{M}}$ are continuously differentiable inside $\mathcal{N}_1$.

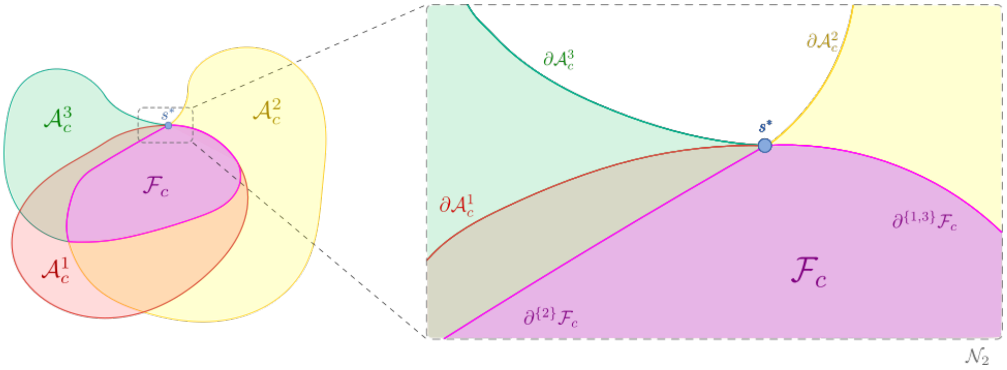

Figure S8: An example in which $M = 3$ and $s^* \in \partial\mathcal{F}_c$ lies at the intersection of $\partial\mathcal{A}_c^1$, $\partial\mathcal{A}_c^2$ and $\partial\mathcal{A}_c^3$, and therefore belongs to $\partial^{\{1,2,3\}}\mathcal{F}_c$. It may also be seen above that $s^* \in \overline{\partial^{\{1,3\}}\mathcal{F}_c}$ and $s^* \in \overline{\partial^{\{2\}}\mathcal{F}_c}$. By taking $\phi = \{1\}$, it follows that s^* lies inside the set specified by Theorem S5. $\mathcal{N}_2$ is represented as a gray dashed rectangle.

By Assumption 2.2.3 Statement (b), $\nabla g^i(s^*) \neq 0$ for all $i \in \mathcal{M}$. Therefore, the directional derivative of $g^{\tilde{i}}$ in the direction of $\nabla g^{\tilde{i}}(s^*)$, $g^{\tilde{i}}_{\nabla g^{\tilde{i}}(s^*)}(s^*)$, must be strictly greater than zero. As $g^{\tilde{i}}_{\nabla g^{\tilde{i}}(s)}(s) = \nabla g^{\tilde{i}}(s) \cdot \nabla g^{\tilde{i}}(s)$ is a continuous function of s inside $\mathcal{N}_1$ we can further restrict our attention to a convex open neighbourhood of s^*, $\mathcal{N}_2 \subseteq \mathcal{N}_1$, over which $g^{\tilde{i}}_{\nabla g^{\tilde{i}}} > 0$. An example, which we shall refer to throughout the proof, is provided by Fig. S8.

Now, define the projection of s_n to $\partial \mathcal{A}^i_c$ as;

$$\mathfrak{p}_i(s_n) = \underset{s \in \partial \mathcal{A}^i_c}{\arg\inf} d(s, s_n).$$

Assume for ease, that $\mathfrak{p}_i(s_n)$ is uniquely defined. Define $L(s_n)$ to be the closed line segment which runs from $\mathfrak{p}_{\tilde{i}}(s_n)$ to s_n and $\vec{L}(s_n)$ to be the corresponding vector running from $\mathfrak{p}_{\tilde{i}}(s_n)$ to s_n. Define the projection of s_n to $\partial \mathcal{F}_c$ as:

$$\mathfrak{p}_{\partial \mathcal{F}_c}(s_n) = \underset{s \in L(s_n) \cap \partial \mathcal{F}_c}{\arg\inf} d(s, s_n).$$

The existence of such a projection is guaranteed by fact that $s_n \in (\mathcal{F}_c)^\circ$, $\mathfrak{p}_{\tilde{i}}(s_n) \notin (\mathcal{F}_c)^\circ$, and $\{g^j\}_{j \in \mathcal{M}}$ are continuous along $L(s_n)$. From the definition of $\mathfrak{p}_{\tilde{i}}(s_n)$ and noting that $s^* \in \partial \mathcal{A}^{\tilde{i}}_c$, it can be seen that $d(s_n, \mathfrak{p}_{\tilde{i}}(s_n)) \to 0$ as $n \to \infty$ and therefore $\mathfrak{p}_{\tilde{i}}(s_n) \to s^*$. Similarly, as $\mathfrak{p}_{\partial \mathcal{F}_c}(s_n) \in L(s_n)$, it follows that $\mathfrak{p}_{\partial \mathcal{F}_c}(s_n) \to s^*$. Using the example given in Fig. S8, these sequences are illustrated by Fig. S9.

Assume that n is large enough such that $\mathfrak{p}_{\tilde{i}}(s_n) \in \mathcal{N}_2$. This means that $\mathfrak{p}_{\tilde{i}}(s_n)$ lies on the (locally) continuous curve $\partial \mathcal{A}^{\tilde{i}}_c$. As $\mathcal{N}_2$ is open and $g^{\tilde{i}}$ is continuously differentiable over $\mathcal{N}_2$, it follows that in an open neighbourhood of $\mathfrak{p}_{\tilde{i}}(s_n)$, $g^{\tilde{i}}$ is continuously differentiable. By construction, $\mathfrak{p}_{\tilde{i}}(s_n)$ is the point s which locally minimizes the distance function $d(s_n, s)$ subject to the constraint $g^{\tilde{i}}(s) = 0$. Therefore, $\mathfrak{p}_{\tilde{i}}(s_n)$ is a local minima of the Lagrangian function;

$$\mathcal{L}(s) = d(s_n, s) + \lambda_n g^{\tilde{i}}(s).$$

By setting $\nabla \mathcal{L}(s)$ equal to zero, we can see that $\mathfrak{p}_{\tilde{i}}(s_n)$ satisfies:

$$2(s_n - \mathfrak{p}_{\tilde{i}}(s_n)) = \lambda_n \nabla g^{\tilde{i}}(\mathfrak{p}_{\tilde{i}}(s_n)).$$

We note that λ_n must be non-negative as if it were negative, it would be possible to find a point $p \in L(s_n)$ which is closer to s_n than $\mathfrak{p}_{\tilde{i}}(s_n)$ such that $g^{\tilde{i}}(p) = 0$. The existence of

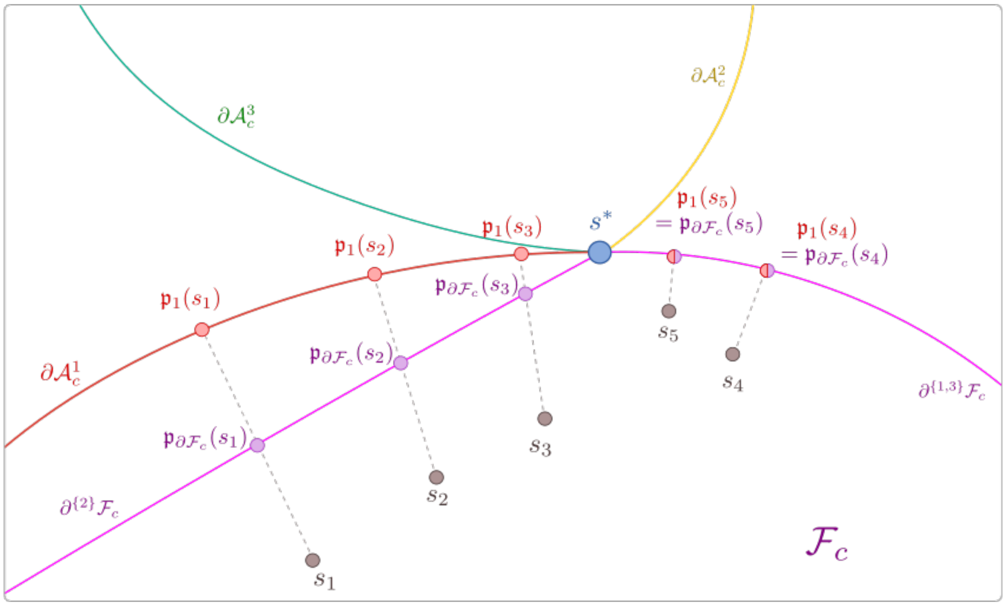

Figure S9: An illustration of the sequences $\{s_n\}_{n\in\mathbb{N}}$ (shown in gray), $\{\mathfrak{p}_{\tilde{i}}(s_n)\}_{n\in\mathbb{N}}$ (red) and $\{\mathfrak{p}_{\partial\mathcal{F}_c}(s_n)\}_{n\in\mathbb{N}}$ (purple) for an example in which $\phi = \{1\}$ and $\tilde{i} = 1$. Also displayed are dashed gray lines representing the line segments $\{L(s_n)\}_{n\in\mathbb{N}}$ and the point s^* in dark blue.

such a point p would contradict the definition of $\mathfrak{p}_{\tilde{i}}(s_n)$. If λ_n were equal to zero, it would follow that $s_n = \mathfrak{p}_{\tilde{i}}(s_n)$. However, this cannot happen as, by construction, $s_n \in (\mathcal{F}_c)^\circ$ and $\mathfrak{p}_{\tilde{i}}(s_n) \notin (\mathcal{F}_c)^\circ$. Therefore, $\lambda_n > 0$.

We shall now show that for n large enough, $g^{\tilde{i}}$ is strictly increasing along $\vec{L}(s_n)$ from $\mathfrak{p}_{\tilde{i}}(s_n)$ to s_n. To show this, we proceed by proof by contradiction and assume the negation:

$$\forall N \in \mathbb{N}, \exists n \geq N \text{ such that } \exists c_n \in L(s_n) \text{ such that } g^{\tilde{i}}_{\vec{L}(s_n)}(c_n) \leq 0.$$

Let $\{c_{n_m}\}_{m\in\mathbb{N}}$ be a sequence satisfying the above. Trivially, as $c_{n_m} \in L(s_{n_m})$, it follows that $c_{n_m} \to s^*$ as $m \to \infty$. By noting $\vec{L}(s_n)$ is a positive multiple of $\nabla g^{\tilde{i}}(\mathfrak{p}_{\tilde{i}}(s_n))$, it follows that;

$$g^{\tilde{i}}_{\nabla g^{\tilde{i}}(s^*)}(s^*) = \lim_{m\to\infty} g^{\tilde{i}}_{\nabla g^{\tilde{i}}(\mathfrak{p}_{\tilde{i}}(s_{n_m}))}(c_{n_m}) \leq 0.$$

However, this is a contradiction as;

$$g^{\tilde{i}}_{\nabla g^{\tilde{i}}(s^*)}(s^*) = \nabla g^{\tilde{i}}(s^*) \cdot \nabla g^{\tilde{i}}(s^*) > 0.$$

Therefore, for n large enough, $g^{\tilde{i}}$ is strictly increasing along $\vec{L}(s_n)$ from $\mathfrak{p}_{\tilde{i}}(s_n)$ to s_n and;

$$0 = g^{\tilde{i}}(\mathfrak{p}_{\tilde{i}}(s_n)) \leq g^{\tilde{i}}(\mathfrak{p}_{\partial\mathcal{F}_c}(s_n)) < g^{\tilde{i}}(s_n),$$

which implies;

$$0 < g^{\tilde{i}}(s_n) - g^{\tilde{i}}(\mathfrak{p}_{\partial\mathcal{F}_c}(s_n)) \leq g^{\tilde{i}}(s_n) - g^{\tilde{i}}(\mathfrak{p}_{\tilde{i}}(s_n)).$$

As $\mathcal{N}_2$ does not intersect $\overline{\partial^{\phi_2}\mathcal{F}_c}$ for any $\phi_2 \subseteq \phi$, it follows that for n large enough:

$$\mathfrak{p}_{\partial\mathcal{F}_c}(s_n) \in \partial\mathcal{F}_c \setminus \bigcup_{\phi_2 \subseteq \phi} \partial^{\phi_2}\mathcal{F}_c = \bigcup_{\phi_1 \not\subseteq \phi} \partial^{\phi_1}\mathcal{F}_c.$$

For fixed n large enough, this means that $\mathfrak{p}_{\partial\mathcal{F}_c}(s_n) \in \partial^{\phi_1}\mathcal{F}_c$ for some $\phi_1 \not\subseteq \phi$. As $\phi_1 \not\subseteq \phi$, it follows that $\phi_1 \setminus \phi$ is non-empty. Let $\tilde{j}_n \in \phi_1 \setminus \phi$. From the definition of $\partial^{\phi_1}\mathcal{F}_c$, it now follows that $g^{\tilde{j}_n}(\mathfrak{p}_{\partial\mathcal{F}_c}(s_n)) = 0$.

By applying the generalized mean value theorem in the direction of $\vec{L}(s_n)$ between $\mathfrak{p}_{\partial\mathcal{F}_c}(s_n)$ and s_n, it can be seen that there must exist a point $t_n \in L(s_n)$ between $\mathfrak{p}_{\partial\mathcal{F}_c}(s_n)$ and s_n which satisfies;

$$\frac{g^{\tilde{j}_n}_{\vec{L}(s_n)}(t_n)}{g^{\tilde{i}}_{\vec{L}(s_n)}(t_n)} = \frac{g^{\tilde{j}_n}(s_n) - g^{\tilde{j}_n}(\mathfrak{p}_{\partial\mathcal{F}_c}(s_n))}{g^{\tilde{i}}(s_n) - g^{\tilde{i}}(\mathfrak{p}_{\partial\mathcal{F}_c}(s_n))} = \frac{g^{\tilde{j}_n}(s_n)}{g^{\tilde{i}}(s_n) - g^{\tilde{i}}(\mathfrak{p}_{\partial\mathcal{F}_c}(s_n))}$$

$$\geq \frac{g^{\tilde{j}_n}(s_n)}{g^{\tilde{i}}(s_n) - g^{\tilde{i}}(\mathfrak{p}_{\tilde{i}}(s_n))} = \frac{g^{\tilde{j}_n}(s_n)}{g^{\tilde{i}}(s_n)},$$

where the second and third equality follow by noting that $g^{\tilde{j}_n}(\mathfrak{p}_{\partial\mathcal{F}_c}(s_n)) = 0$ and $g^{\tilde{i}}(\mathfrak{p}_{\tilde{i}}(s_n)) = 0$, whilst the inequality follows by the previous argument. The sequence $\{t_n\}_{n\in\mathbb{N}}$ is illustrated by Fig. S10, alongside the sequences $\{s_n\}_{n\in\mathbb{N}}$ and $\{\mathfrak{p}_{\partial\mathcal{F}_c}(s_n)\}_{n\in\mathbb{N}}$.

We have shown that for n sufficiently large, there must exist an $\tilde{i} \in \phi$ and $\tilde{j}_n \in \mathcal{M} \setminus \phi$ such that;

$$\frac{g^{\tilde{j}_n}_{\vec{L}(s_n)}(t_n)}{g^{\tilde{i}}_{\vec{L}(s_n)}(t_n)} \geq \frac{g^{\tilde{j}_n}(s_n)}{g^{\tilde{i}}(s_n)}.$$

It follows that for n sufficiently large, there exists an $\tilde{i} \in \phi$ and $\tilde{j}_n \in \phi_1 \setminus \phi \subseteq \mathcal{M} \setminus \phi$ such that;

$$\frac{g^{\tilde{j}_n}_{\vec{L}(s_n)}(t_n)}{g^{\tilde{i}}_{\vec{L}(s_n)}(t_n)} \geq \min_{\substack{i\in\phi \\ j\in\mathcal{M}\setminus\phi}} \left(\frac{g^j(s_n)}{g^i(s_n)}\right) = a_n > 0,$$

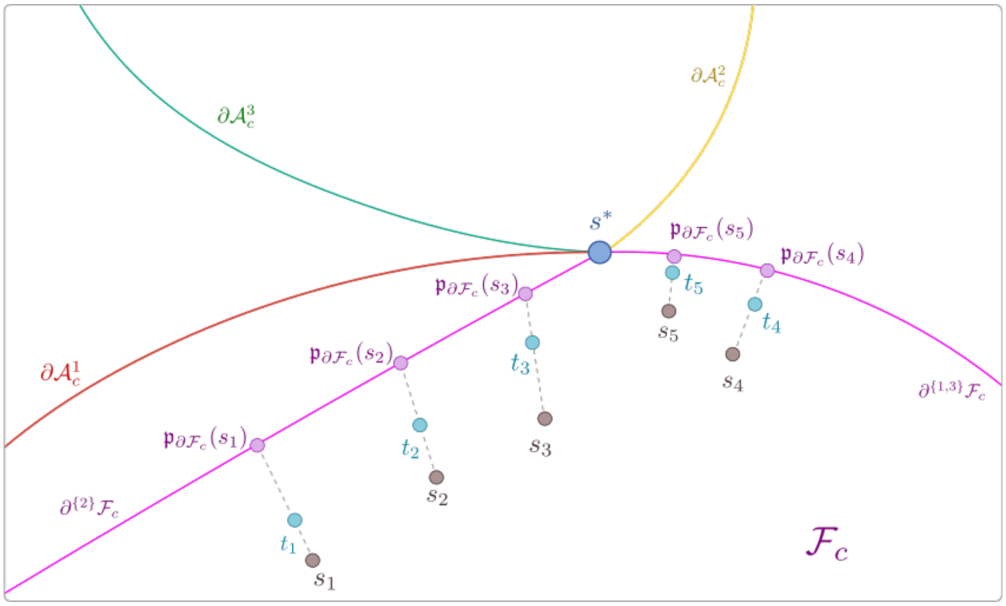

Figure S10: An illustration of the sequences $\{s_n\}_{n\in\mathbb{N}}$ (shown in gray), $\{t_n\}_{n\in\mathbb{N}}$ (light blue) and $\{\mathfrak{p}_{\partial\mathcal{F}_c}(s_n)\}_{n\in\mathbb{N}}$ (purple) for an example in which $\phi=\{1\}$ and $\tilde{i}=1$. Also displayed are dashed gray lines representing segments of $\{L(s_n)\}_{n\in\mathbb{N}}$ and the point s^* in dark blue. In this example, a possible choice for the sequence $\{\tilde{j}_n\}_{n\in\mathbb{N}}$ is given by $\tilde{j}_1=\tilde{j}_2=\tilde{j}_3=2$ and $\tilde{j}_4=\tilde{j}_5=3$.

where the second inequality follows from the fact that $s_n\in(\mathcal{F}_c)^\circ$. As $\tilde{j}_n$ is dependent on n, we note that:

$$\max_{j\in\mathcal{M}\setminus\phi}\left(\frac{g^j_{\vec{L}(s_n)}(t_n)}{g^{\tilde{i}}_{\vec{L}(s_n)}(t_n)}\right)\geq a_n>0.$$

Now we note that, by construction $t_n\to s^*$ as $n\to\infty$ (c.f. Fig. S10). Therefore, the below convergence holds:

$$\max_{j\in\mathcal{M}\setminus\phi}\left(\frac{g^j_{\vec{L}(s_n)}(t_n)}{g^{\tilde{i}}_{\vec{L}(s_n)}(t_n)}\right)\to\max_{j\in\mathcal{M}\setminus\phi}\left(\frac{\nabla g^{\tilde{i}}(s^*)\cdot\nabla g^j(s^*)}{||\nabla g^{\tilde{i}}(s^*)||^2}\right)\text{ as }n\to\infty.$$

As $\nabla g^{\tilde{i}}(s^*)$ is non-zero by assumption the above limit is finite and it follows that we have bounded $\{a_n\}_{n\in\mathbb{N}}$ above and below with convergent sequences.

□

S4.8 Theorem S6: Equality of Maxima Involving $\mathcal{D}_\phi$

Theorem S6. *Let $\tilde{\mathcal{D}}_\phi$ be an arbitrary set satisfying $\tilde{\mathcal{D}}_\phi \subseteq \mathcal{D}_\phi$. For n large enough, the following equality is true:*

$$\max_{\phi \in \mathcal{P}^+(\mathcal{M})} \left(\sup_{s \in \partial^\phi \mathcal{F}_c} \left| \min_{i \in \phi}(G_n^i(s)) \right| \right) = \max_{\phi \in \mathcal{P}^+(\mathcal{M})} \left(\sup_{s \in \partial^\phi \mathcal{F}_c \cup \tilde{\mathcal{D}}_\phi} \left| \min_{i \in \phi}(G_n^i(s)) \right| \right),$$

where $G_n^i(s)$ is shorthand for $\hat{g}_n^i(s) - g^i(s)$.

Proof. To begin, note that the right hand side of the statement in Theorem S6 is equal to:

$$\max \left[\max_{\phi \in \mathcal{P}^+(\mathcal{M})} \left(\sup_{s \in \tilde{\mathcal{D}}_\phi} \left| \min_{i \in \phi}(G_n^i(s)) \right| \right), \max_{\phi \in \mathcal{P}^+(\mathcal{M})} \left(\sup_{s \in \partial^\phi \mathcal{F}_c} \left| \min_{i \in \phi}(G_n^i(s)) \right| \right) \right].$$

It therefore follows that, in order to prove the result of the theorem, we need only show that:

$$\max_{\phi \in \mathcal{P}^+(\mathcal{M})} \left(\sup_{s \in \tilde{\mathcal{D}}_\phi} \left| \min_{i \in \phi}(G_n^i(s)) \right| \right) \leq \max_{\phi \in \mathcal{P}^+(\mathcal{M})} \left(\sup_{s \in \partial^\phi \mathcal{F}_c} \left| \min_{i \in \phi}(G_n^i(s)) \right| \right). \tag{S13}$$

To show the above inequality is true, we begin by noting that, as $\tilde{\mathcal{D}}_\phi \subseteq \mathcal{D}_\phi$:

$$\max_{\phi \in \mathcal{P}^+(\mathcal{M})} \left(\sup_{s \in \tilde{\mathcal{D}}_\phi} \left| \min_{i \in \phi}(G_n^i(s)) \right| \right) \leq \max_{\phi \in \mathcal{P}^+(\mathcal{M})} \left(\sup_{s \in \mathcal{D}_\phi} \left| \min_{i \in \phi}(G_n^i(s)) \right| \right).$$

Now, let $\phi^* \in \mathcal{P}^+(\mathcal{M})$ and $s^* \in \mathcal{D}_{\phi^*}$ be the values which maximize the expression on the right-hand side of the above. In other words, let ϕ^* and s^* be the values of ϕ and s which satisfy $s^* \in \mathcal{D}_{\phi^*}$ and:

$$\max_{\phi \in \mathcal{P}^+(\mathcal{M})} \left(\sup_{s \in \mathcal{D}_\phi} \left| \min_{i \in \phi}(G_n^i(s)) \right| \right) = \left| \min_{i \in \phi^*}(G_n^i(s^*)) \right|.$$

Now, as $s^* \in \mathcal{D}_{\phi^*}$, we know that there must exist an ϕ_1 and ϕ_2 such that $\phi_1 \subset \phi^* \subset \phi_2$, $s^* \in \overline{\partial^{\phi_1} \mathcal{F}_c}$ and $s^* \in \overline{\partial^{\phi_2} \mathcal{F}_c}$. Fix such ϕ_1 and ϕ_2 and note that, from trivial properties of the minimum function and the fact that $\phi_1 \subset \phi^* \subset \phi_2$, it must be true that either :

$$\left| \min_{i \in \phi^*}(G_n^i(s^*)) \right| \leq \left| \min_{i \in \phi_1}(G_n^i(s^*)) \right| \text{ or } \left| \min_{i \in \phi^*}(G_n^i(s^*)) \right| \leq \left| \min_{i \in \phi_2}(G_n^i(s^*)) \right|.$$

In other words;

$$\big|\min_{i\in\phi^*}(G_n^i(s^*))\big| \leq \max\Bigg(\big|\min_{i\in\phi_1}(G_n^i(s^*))\big|, \big|\min_{i\in\phi_2}(G_n^i(s^*))\big|\Bigg) = \max_{\phi\in\{\phi_1,\phi_2\}}\Bigg(\big|\min_{i\in\phi}(G_n^i(s^*))\big|\Bigg).$$

As, by the construction of $\mathcal{D}_{\phi^*}$, we know that $s^* \in \overline{\partial^{\phi_1}\mathcal{F}_c}$ and $s^* \in \overline{\partial^{\phi_2}\mathcal{F}_c}$, it therefore follows that the above is less than or equal to:

$$\max_{\phi\in\{\phi_1,\phi_2\}}\Bigg(\sup_{s\in\overline{\partial^{\phi}\mathcal{F}_c}}\big|\min_{i\in\phi}(G_n^i(s))\big|\Bigg).$$

As all functions $\{G_n^i\}_{i\in\mathcal{M}}$ are continuous for n large enough it follows that suprema taken over $\partial^{\phi}\mathcal{F}_c$ attain their limit points, and therefore the above is equal to;

$$\max_{\phi\in\{\phi_1,\phi_2\}}\Bigg(\sup_{s\in\partial^{\phi}\mathcal{F}_c}\big|\min_{i\in\phi}(G_n^i(s))\big|\Bigg) \leq \max_{\phi\in\mathcal{P}^+(\mathcal{M})}\Bigg(\sup_{s\in\partial^{\phi}\mathcal{F}_c}\big|\min_{i\in\phi}(G_n^i(s))\big|\Bigg).$$

This is the expression we set out to prove. We have now shown that:

$$\max_{\phi\in\mathcal{P}^+(\mathcal{M})}\Bigg(\sup_{s\in\tilde{\mathcal{D}}_{\phi}}\big|\min_{i\in\phi}(G_n^i(s))\big|\Bigg) \leq \big|\min_{i\in\phi^*}(G_n^i(s^*))\big| \leq \max_{\phi\in\mathcal{P}^+(\mathcal{M})}\Bigg(\sup_{s\in\partial^{\phi}\mathcal{F}_c}\big|\min_{i\in\phi}(G_n^i(s))\big|\Bigg),$$

as required.

□

S4.9 Theorem S7: Convergence of $\mathcal{O}_c^{n,\phi}$

Theorem S7. *For all $\phi \in \mathcal{P}^+(\mathcal{M})$, there exists a set $\tilde{\mathcal{D}}_\phi \subseteq \mathcal{D}_\phi$ such that $d_H(\mathcal{O}_c^{n,\phi}, \partial^\phi \mathcal{F}_c \cup \tilde{\mathcal{D}}_\phi) \to 0$ as $n \to \infty$.*

Proof. Suppose $\phi = \mathcal{M}$. As $\mathcal{D}_\mathcal{M} = \emptyset$, the only way for the theorem to be satisfied is if $d_H(\mathcal{O}_c^{n,\mathcal{M}}, \partial^\mathcal{M} \mathcal{F}_c) \to 0$ as $n \to \infty$. As $\{\mathcal{O}_c^{n,\mathcal{M}}\}_{n\in\mathbb{N}}$ are strictly decreasing it is true that;

$$d_H\left(\mathcal{O}_c^{n,\mathcal{M}}, \bigcap_{m\geq 1} \overline{\mathcal{O}_c^{m,\mathcal{M}}}\right) \to 0 \text{ as } n \to \infty.$$

We shall now show that $\partial^\mathcal{M}\mathcal{F}_c = \cap_{m\geq 1}\overline{\mathcal{O}_c^{m,\mathcal{M}}}$. Let $s^* \in \cap_{m\geq 1}\overline{\mathcal{O}_c^{m,\mathcal{M}}}$. It follows that there must exist a sequence $\{s_m\}_{m\in\mathbb{N}}$ such that $s_m \in \overline{\mathcal{O}_c^{m,\mathcal{M}}}$ and $s_m \to s^*$ as $m \to \infty$. As $\overline{\mathcal{O}_c^{m,\mathcal{M}}} \subseteq \mathcal{F}_c^m$, it follows that $s_m \in \mathcal{F}_c^m$ and therefore;

$$|\min_{i\in\mathcal{M}} g^i(s^*)| = \lim_{m\to\infty} |\min_{i\in\mathcal{M}} g^i(s_m)| \leq \lim_{m\to\infty} \eta_m = 0.$$

Therefore, $\min_{i\in\mathcal{M}} g^i(s^*) = 0$ and, as a result, for all $i \in \mathcal{M}$, $g^i(s^*) \geq 0$. As $\overline{\mathcal{O}_c^{m,\mathcal{M}}} \subseteq \cap_{i\in\mathcal{M}}\overline{(\mathcal{A}_c^i)^c}$, it also follows that $s_m \in \cap_{i\in\mathcal{M}}\overline{(\mathcal{A}_c^i)^c}$. Therefore, $g^i(s_m) \leq 0$ for all $i \in \mathcal{M}$, and;

$$g^i(s^*) = \lim_{m\to\infty} g^i(s_m) \leq 0.$$

Therefore $g^i(s^*) = 0$ for all $i \in \mathcal{M}$ and, thus, $s^* \in \partial^\mathcal{M}\mathcal{F}_c$. As a result, $\cap_{m\geq 1}\overline{\mathcal{O}_c^{m,\mathcal{M}}} \subseteq \partial^\mathcal{M}\mathcal{F}_c$.

Let $t^* \in \partial^\mathcal{M}\mathcal{F}_c$ and fix $m \in \mathbb{N}$. As $\min_{i\in\mathcal{M}} g^i(t^*) = 0$, we can define a neighbourhood of t^* over which $|\min_{i\in\mathcal{M}} g^i| < \eta_m$. Such a neighbourhood must be a subset of $\mathcal{F}_c^m$ by definition. By the ball assumption, any neighbourhood of t^* must intersect $\mathcal{J}_c^\mathcal{M} = \cap_{i\in\mathcal{M}}(\mathcal{A}_c^i)^c$. Therefore, for arbitrary m, every neighbourhood of t^* intersects $\cap_{i\in\mathcal{M}}(\mathcal{A}_c^i)^c \cap \mathcal{F}_c^m = \mathcal{O}_c^{m,\mathcal{M}}$, and thus $t^* \in \cap_{m\geq 1}\overline{\mathcal{O}_c^{m,\mathcal{M}}}$. Therefore, $\partial^\mathcal{M}\mathcal{F}_c \subseteq \cap_{m\geq 1}\overline{\mathcal{O}_c^{m,\mathcal{M}}}$. Combining this with the previous, it follows that $\partial^\mathcal{M}\mathcal{F}_c = \cap_{m\geq 1}\overline{\mathcal{O}_c^{m,\mathcal{M}}}$, as desired.

Suppose $\phi \neq \mathcal{M}$. Let $s^* \in \partial^\phi \mathcal{F}_c$ and fix $n \in \mathbb{N}$. By the definition of $\partial^\phi \mathcal{F}_c$, $g^i(s^*) = 0$ for all $i \in \phi$ and $g^j(s^*) > 0$ for all $j \in \mathcal{M} \setminus \phi$. Therefore, for ϵ small enough, the open ϵ-ball about s^*, $B_\epsilon(s^*)$, satisfies the below:

$$\forall s \in B_\epsilon(s^*), g^j(s) > 0 \text{ and } -\eta_n < g^i(s) < \eta_n,$$

for all $i \in \phi$ and $j \in \mathcal{M} \setminus \phi$. By the ball assumption, $B_\epsilon(s^*) \cap \mathcal{J}_c^\phi$ is non-empty. Therefore, by the definition of $\mathcal{J}_c^\phi$, for ϵ small enough, there must exist an $s \in B_\epsilon(s^*) \cap \mathcal{J}_c^\phi$ such that $-\eta_n < g^i(s) < 0 < g^j(s)$ for all $i \in \phi$ and $j \in \mathcal{M} \setminus \phi$. For such s it follows that $|\min_{i \in \mathcal{M}} g^i(s)| < \eta_n$ and therefore $s \in \mathcal{J}_c^\phi \cap \mathcal{F}_c^n \subseteq \mathcal{O}_c^{n,\phi}$. Thus, $s \in \mathcal{O}_c^{n,\phi}$. As $\epsilon > 0$ was arbitrary, it follows that $s^* \in \overline{\mathcal{O}_c^{n,\phi}}$. As $s^* \in \partial^\phi \mathcal{F}_c$ was arbitrary, it follows that $\partial^\phi \mathcal{F}_c \subseteq \overline{\mathcal{O}_c^{n,\phi}}$. As n was arbitrary, $\partial^\phi \mathcal{F}_c \subseteq \cap_{n \geq 1} \overline{\mathcal{O}_c^{n,\phi}}$.

Define $\tilde{\mathcal{D}}_\phi$ as $\tilde{\mathcal{D}}_\phi = \bigcap_{n \geq 1} \overline{\mathcal{O}_c^{n,\phi}} \setminus \overline{\partial^\phi \mathcal{F}_c}$. It now follows that $\overline{\partial^\phi \mathcal{F}_c} \cup \tilde{\mathcal{D}}_\phi = \bigcap_{n \geq 1} \overline{\mathcal{O}_c^{n,\phi}}$. As $\{\overline{\mathcal{O}_c^{n,\phi}}\}_{n \in \mathbb{N}}$ is sequence of closed decreasing sets, it now follows that $d_H(\mathcal{O}_c^{n,\phi}, \partial^\phi \mathcal{F}_c \cup \tilde{\mathcal{D}}_\phi) \to 0$ as $n \to \infty$. We must now show that $\tilde{\mathcal{D}}_\phi \subseteq \mathcal{D}_\phi$. By the definition of $\mathcal{D}_\phi$ (c.f. Section S4.6), this means we must show that, for arbitrary $s^* \in \tilde{\mathcal{D}}_\phi$, there exists ϕ_1 and ϕ_2 such that $\phi_1 \subset \phi \subset \phi_2$ and $s^* \in \overline{\partial^{\phi_1} \mathcal{F}_c} \cap \overline{\partial^{\phi_2} \mathcal{F}_c}$. In other words, we will prove the following two statements:

$$\exists \phi_2 \supset \phi \text{ such that } s^* \in \overline{\partial^{\phi_2} \mathcal{F}_c}, \tag{S14}$$

$$\exists \phi_1 \subset \phi \text{ such that } s^* \in \overline{\partial^{\phi_1} \mathcal{F}_c}. \tag{S15}$$

It can be seen that, as $\tilde{\mathcal{D}}_\phi \subseteq \cap_{n \geq 1} \overline{\mathcal{O}_c^{n,\phi}}$, it follows that $\tilde{\mathcal{D}}_\phi \subseteq \partial \mathcal{F}_c$ and therefore $s^* \in \partial \mathcal{F}_c$. To show Statement (S14), we note that, by the ball assumption, any open neighbourhood of s^* has a non-empty intersection with $(\mathcal{F}_c)^\circ$. Further, by the construction of $\tilde{\mathcal{D}}_\phi$, it follows that any open neighbourhood of s^* has a non-empty intersection with $\mathcal{O}_c^{n,\phi}$ for arbitrary n. Fix n. By the definitions of $(\mathcal{F}_c)^\circ$ and $\mathcal{O}_c^{n,\phi}$, it now follows that for any $i \in \phi$;

$$\forall \epsilon > 0, \quad B_\epsilon(s^*) \cap \{s \in S : g^i(s) > 0\} \neq \emptyset \quad \text{ and } \quad B_\epsilon(s^*) \cap \{s \in S : g^i(s) < 0\} \neq \emptyset.$$

As $B_\epsilon(s^*)$ is path connected, it follows that for all $i \in \phi$ and $\epsilon > 0$, there exists an $s \in B_\epsilon(s^*)$ such that $g^i(s) = 0$. As ϵ is arbitrary, it now follows that $g^i(s^*) = 0$ for all $i \in \phi$. Therefore, by the definition of $\partial^\phi \mathcal{F}_c$, $s^* \in \partial^{\phi_2} \mathcal{F}_c$ for some $\phi_2 \supseteq \phi$. As by construction $\tilde{\mathcal{D}}_\phi \cap \overline{\partial^\phi \mathcal{F}_c} = \emptyset$,

it follows that $s^* \in \overline{\partial^{\phi_2}\mathcal{F}_c}$ for some $\phi_2 \supset \phi$. This concludes the proof of Statement (S14).

To prove Statement (S15), we recall from the previous argument $s^* \in \partial^{\phi_2}\mathcal{F}_c$ for some $\phi_2 \supset \phi$. From the definition of $\partial^{\phi_2}\mathcal{F}_c$, it is true that for all k in $\mathcal{M} \setminus \phi_2$, $g^k(s^*) > 0$. Therefore, we can choose $\epsilon > 0$ small enough such that;

$$\forall s \in B_\epsilon(s^*), \forall k \in \mathcal{M} \setminus \phi_2, g^k(s) > 0. \tag{S16}$$

Similarly, from the definition of $\partial^{\phi_2}\mathcal{F}_c$, it is true that for all $i \in \phi$, $g^i(s^*) = 0$. Therefore, for arbitrary n and ϵ small enough;

$$\forall s \in B_\epsilon(s^*), \forall i \in \phi, |g^i(s)| < \eta_n. \tag{S17}$$

Fix $n \in \mathbb{N}$ and let ϵ satisfy the above. As $s^* \in \partial^{\phi_2}\mathcal{F}_c$, $g^j(s^*) = 0$ for all $j \in \phi_2 \setminus \phi$. Therefore, $s^* \in S_0 := \{s \in B_\epsilon(s^*) | \min_{j \in \phi_2 \setminus \phi}(g^j(s)) = 0\}$. By the path-connectedness assumption, for ϵ small enough, S_0 must partition $B_\epsilon(s^*)$ into two path-connected components; S_1 and S_2.

As S_1 and S_2 are path-connected, $\min_{j \in \phi_2 \setminus \phi} g^j$ cannot change sign within S_1 or within S_2. By the ball assumption, $B_\epsilon(s^*)$ must intersect $(\mathcal{F}_c)^\circ$ (where $\min_{j \in \phi_2 \setminus \phi} g^j > 0$) and $\mathcal{J}_c^{\phi_2}$ (where $\min_{j \in \phi_2 \setminus \phi} g^j < 0$). It therefore follows that in one of S_1 or S_2, $\min_{j \in \phi_2 \setminus \phi} g^j < 0$, whilst in the other set $\min_{j \in \phi_2 \setminus \phi} g^j > 0$. Without loss of generality, assume that:

$$S_1 = \left\{ s \in B_\epsilon(s^*) \middle| \min_{j \in \phi_2 \setminus \phi}(g^j(s)) > 0 \right\} \quad \text{and} \quad S_2 = \left\{ s \in B_\epsilon(s^*) \middle| \min_{j \in \phi_2 \setminus \phi}(g^j(s)) < 0 \right\}.$$

By construction $s^* \in \overline{\mathcal{O}_c^{m,\phi}}$ for arbitrary m and, therefore, $B_\epsilon(s^*) \cap \mathcal{O}_c^{n,\phi}$ is non-empty. Suppose $B_\epsilon(s^*) \cap \mathcal{O}_c^{n,\phi} \subseteq S_0$ and let $t^* \in B_\epsilon(s^*) \cap \mathcal{O}_c^{n,\phi}$. By the definition of $\mathcal{O}_c^{n,\phi}$, $g^i(t^*) < 0$ for all $i \in \phi$. Therefore, we can choose $\delta > 0$ such that $B_\delta(t^*) \subset B_\epsilon(s^*)$ and for all $t \in B_\delta(t^*)$, $g^i(t) < 0$ for all $i \in \phi$. This construction is illustrated by Fig. S11.

As $B_\delta(t^*) \cap S_1$ is non-empty (this can be seen by noting that S_0 is composed of segments of C^1 curves, and that S_1 is non-empty), we can fix $t \in B_\delta(t^*) \cap S_1$. By the construction of $B_\delta(t^*)$, $g^i(t) < 0$ for all $i \in \phi$. By the definition of S_1, $g^j(t) > 0$ for all $j \in \phi_2 \setminus \phi$. By the definition of $B_\epsilon(s^*)$, $g^k(t) > 0$ for all $k \in \mathcal{M} \setminus \phi_2$ and $-\eta_n < g^i(t)$ for all $i \in \phi$ (c.f. Statements (S16) and (S17)). By combining these facts and noting the definition of $\mathcal{O}_c^{n,\phi}$, it can now be seen that $t \in \mathcal{O}_c^{n,\phi}$.

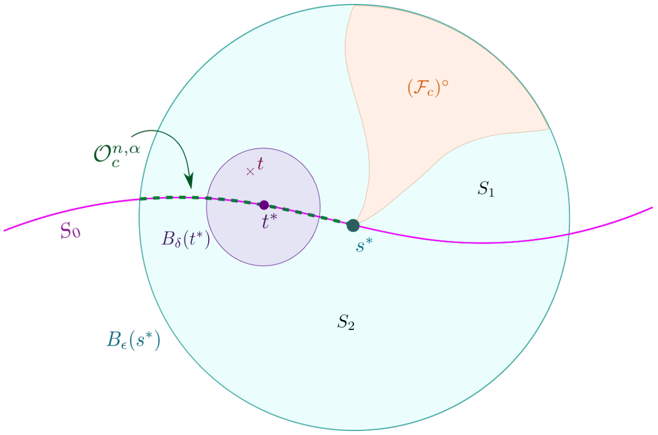

Figure S11: Illustration of the situation in which $B_\epsilon(s^*) \cap \mathcal{O}_c^{n,\phi} \subseteq S_0$. Displayed are the sets S_0 (light purple), $B_\epsilon(s^*)$ (light blue), $\mathcal{O}_c^{n,\phi}$ (dashed green), $(\mathcal{F}_c)^\circ$ (orange) and $B_\delta(t^*)$ (dark purple). s^* and t^* are labelled with circles and t is depicted as a red cross. Also labelled in black are the regions S_1 and S_2.

Therefore, $t \in B_\epsilon(s^*) \cap \mathcal{O}_c^{n,\phi} \subseteq S_0$ and $t \in S_1$. This is a contradiction as S_0 and S_1 are disjoint. Therefore, it cannot be true that $B_\epsilon(s^*) \cap \mathcal{O}_c^{n,\phi} \subseteq S_0$. By construction, $\mathcal{O}_c^{n,\phi} \cap S_2$ is empty so it must be that $\mathcal{O}_c^{n,\phi} \cap S_1$ is non-empty. By the ball assumption, $B_\epsilon(s^*) \cap (\mathcal{F}_c)^\circ$ is non-empty and therefore, by the definitions of S_0, S_1 and S_2, $(\mathcal{F}_c)^\circ \cap S_1$ is non-empty.

Let $\tilde{s} \in S_1 \cap (\mathcal{F}_c)^\circ$ and $\tilde{t} \in S_1 \cap \mathcal{O}_c^{n,\phi}$. As S_1 is path connected, we can define a path from $\tilde{s}$ to $\tilde{t}$ which does not leave S_1. By the definitions of $(\mathcal{F}_c)^\circ$ and $\mathcal{O}_c^{n,\phi}$, for all $i \in \phi$, $g^i(\tilde{s}) > 0$ and $g^i(\tilde{t}) < 0$. Therefore, along the path from $\tilde{s}$ to $\tilde{t}$ there must exist at least one point at which $g^i = 0$ for at least one $i \in \phi$. Let p be the first such point encountered along the path when moving from $\tilde{s}$ to $\tilde{t}$. This construction is illustrated by Fig. S12.

By the definition of S_1, $g^j(p) > 0$ for all $j \in \phi_2 \setminus \phi$ and, by Statement (S16), $g^k(p) > 0$ for all $k \in \mathcal{M} \setminus \phi_2$. Therefore, $g^j(p) > 0$ for all $j \in \mathcal{M} \setminus \phi$. Furthermore, $p \in \partial \mathcal{F}_c$ as it is the first point along the path from $\tilde{s}$ to $\tilde{t}$ which does not belong to $(\mathcal{F}_c)^\circ$. As $p \in \partial \mathcal{F}_c$ but $g^j(p) > 0$ for all $j \in \mathcal{M} \setminus \phi$, it follows that $p \in \partial^{\phi_1} \mathcal{F}_c$ for some $\phi_1 \subseteq \phi$. As such a point, p, can be found for any sufficiently small neighbourhood of s^*, we can define a sequence $\{p_n\}_{n \in \mathbb{N}}$ such that $p_n \to s^*$ as $n \to \infty$ and each $p_n \in \partial^{\phi_1} \mathcal{F}_c$ for some $\phi_1 \subseteq \phi$.

As there are only finitely many $\phi_1 \subseteq \phi$, for some fixed $\phi_1 \subseteq \phi$, $\{p_n\}_{n \in \mathbb{N}}$ must contain a subsequence $\{\tilde{p}_n\}_{n \in \mathbb{N}}$ which tends to s^* such that all $\tilde{p}_n \in \partial^{\phi_1} \mathcal{F}_c$. As we have identified a

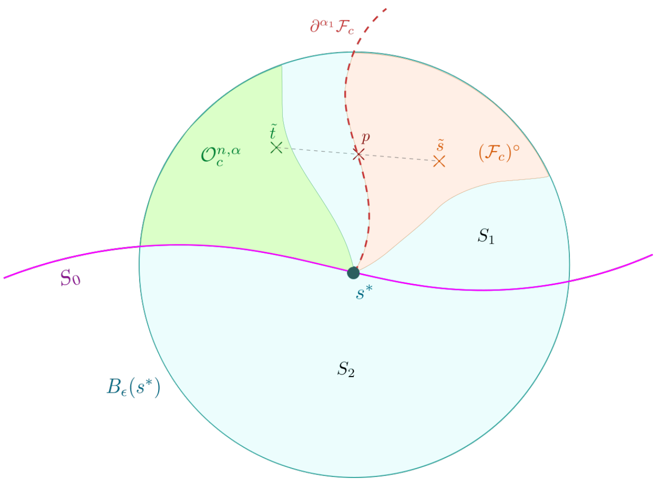

Figure S12: Illustration of the situation in which $B_\epsilon(s^*) \cap \mathcal{O}_c^{n,\phi} \not\subseteq S_0$. Displayed are the sets S_0 (light purple), $B_\epsilon(s^*)$ (light blue), $\mathcal{O}_c^{n,\phi}$ (green), $(\mathcal{F}_c)^\circ$ (orange) and $\partial^{\phi_1}\mathcal{F}_c$ (red dashed). Labelled with crosses are the points $\tilde{s}$, p and $\tilde{t}$, with the path from $\tilde{s}$ to $\tilde{t}$ displayed as a grey dotted line. Also labelled in black are the regions S_1 and S_2.

sequence $\{\tilde{p}_n\}_{n\in\mathbb{N}} \in \partial^{\phi_1}\mathcal{F}_c$ which tends to s^*, it follows that $s^* \in \overline{\partial^{\phi_1}\mathcal{F}_c}$ for some $\phi_1 \subseteq \phi$. As by construction $s^* \notin \overline{\partial^{\phi}\mathcal{F}_c}$, Statement (S15) now follows. □

S4.10 Theorem S8: Convergence of $\max(W^n_\phi) - \max(X^n_\phi)$

Theorem S8. *Defining* W^n_ϕ *and* X^n_ϕ *as in Section S4.2, it is true that:*

$$\max_{\phi\in\mathcal{P}^+(\mathcal{M})}(W^n_\phi) - \max_{\phi\in\mathcal{P}^+(\mathcal{M})}(X^n_\phi) \xrightarrow{p} 0.$$

Proof. To begin, we note that, by a similar argument to that employed in Section S4.5, it is true that $W^n_{\mathcal{M}} = X^n_{\mathcal{M}}$ and therefore, $W^n_{\mathcal{M}} - X^n_{\mathcal{M}} \xrightarrow{p} 0$ as $n \to \infty$ trivially. Suppose $\phi \neq \mathcal{M}$. By Lemma S4, it is true that:

$$|W^n_\phi - X^n_\phi| \leq \sup_{s\in\mathcal{F}_c^{-n,\phi}} \tau_n^{-1}\left|\min_{i\in\mathcal{M}}(\hat{g}^i_n(s)) - \min_{i\in\phi}(\hat{g}^i_n(s) - g^i(s))\right|$$

$$= \sup_{s\in\mathcal{F}_c^{-n,\phi}} \tau_n^{-1}\left|\min\left(\min_{i\in\phi}(\hat{g}^i_n(s)), \min_{j\in\mathcal{M}\setminus\phi}(\hat{g}^j_n(s))\right) - \min_{i\in\phi}(\hat{g}^i_n(s))\right|$$

$$= \sup_{s\in\mathcal{F}_c^{-n,\phi}} \tau_n^{-1}\left|\min\left(0, \min_{j\in\mathcal{M}\setminus\phi}(\hat{g}^j_n(s)) - \min_{i\in\phi}(\hat{g}^i_n(s))\right)\right|,$$

where the first equality is due to the fact that, by the definition of $\mathcal{F}_c^{-n,\phi}$, $g^i(s) = 0$ for all $i \in \phi$ and $s \in \mathcal{F}_c^{-n,\phi}$. By repeating the argument of Section S4.5 and noting that, by definition, for all $s \in \mathcal{F}_c^{-n,\phi}$ and $j \in \mathcal{M}\setminus\phi$ it is true that $g^j(s) > \eta_n$, it now follows that for arbitrary $\epsilon > 0$:

$$\mathbb{P}[|W^n_\phi - X^n_\phi| \geq \epsilon] \leq \mathbb{P}\left[\sup_{s\in\mathcal{F}_c^{-n,\phi}} \tau_n^{-1}\left|\min\left(0, \min_{j\in\mathcal{M}\setminus\phi}(\hat{g}^j_n(s)) - \min_{i\in\phi}(\hat{g}^i_n(s))\right)\right| \geq \epsilon\right]$$

$$\leq \mathbb{P}\left[\tau_n^{-1}\inf_{s\in\mathcal{F}_c^{-n,\phi}}\left(\min_{j\in\mathcal{M}\setminus\phi}(\hat{g}^j_n(s) - g^j(s)) - \min_{i\in\phi}(\hat{g}^i_n(s) - g^i(s))\right) \leq -\epsilon - \tau_n^{-1}\eta_n\right].$$

Through similar logic to that employed in the proof of Theorem S3, it now follows that the above probability tends to zero as $n \to \infty$. Therefore, for all $\phi \in \mathcal{P}^+(\mathcal{M})$, $W^n_\phi - X^n_\phi \xrightarrow{p} 0$ as $n \to \infty$. By applying Lemma S5, the result of the theorem now follows. □

S4.11 Theorem S9: Convergence of $\max(X_\phi^n) - \max(Y_\phi^n)$

Theorem S9. *Defining X_ϕ^n and Y_ϕ^n as in Section S4.2:*

$$\max_{\phi \in \mathcal{P}^+(\mathcal{M})}(X_\phi^n) - \max_{\phi \in \mathcal{P}^+(\mathcal{M})}(Y_\phi^n) \xrightarrow{p} 0.$$

Proof. By the definitions of X_ϕ^n and Y_ϕ^n it follows that;

$$|X_\phi^n - Y_\phi^n| = \left| \sup_{s \in \mathcal{F}_c^{-n,\phi}} \left| \tau_n^{-1} \min_{i \in \phi}(\hat{g}_n^i(s) - g^i(s)) \right| - \sup_{s \in \partial^\phi \mathcal{F}_c} \left| \tau_n^{-1} \min_{i \in \phi}(\hat{g}_n^i(s) - g^i(s)) \right| \right|$$

$$\leq \omega\bigg(|\tau_n^{-1} \min_{i \in \phi}(\hat{g}_n^i(s) - g^i(s))|, \delta_{-n}^\phi \bigg),$$

where ω is the modulus of continuity (c.f. Lemma S7) and δ_{-n}^ϕ is the Hausdorff distance between $\mathcal{F}_c^{-n,\phi}$ and $\partial^\phi \mathcal{F}_c$. It therefore follows that, for arbitrary $\epsilon > 0$:

$$\mathbb{P}[|X_\phi^n - Y_\phi^n| \geq \epsilon] \leq \mathbb{P}\bigg[\omega\bigg(|\tau_n^{-1} \min_{i \in \phi}(\hat{g}_n^i(s) - g^i(s))|, \delta_{-n}^\phi \bigg) \geq \epsilon \bigg].$$

By applying limits to both sides of the above and noting the result of Lemma S7, it now follows that $X_\phi^n - Y_\phi^n \xrightarrow{p} 0$ if $\delta_{-n}^\phi \rightarrow 0$ as $n \rightarrow \infty$.

However, it may be seen from the definitions of $\mathcal{F}_c^{-n,\phi}$ and $\partial^\phi \mathcal{F}_c$ that the sets $\{\mathcal{F}_c^{-n,\phi}\}_{n \in \mathbb{N}}$ are strictly increasing with $\cup_{n \in \mathbb{N}} \mathcal{F}_c^{-n,\phi} = \partial^\phi \mathcal{F}_c$. From standard properties of the Hausdorff distance, it now follows that $\delta_{-n}^\phi \rightarrow 0$ as $n \rightarrow \infty$. All that remains is to apply Lemma S5 to obtain the result of the theorem.

□

S5 Linear Model Assumptions

In this section, we provide additional assumptions which allow the methods of Section 2.3 to be applied in the linear modelling context of Section 3.1. The assumptions provided in this section were first described by Sommerfeld et al. [2018] and the reader is referred to this canonical text for further detail. To state the assumptions, we must introduce some additional notation.

We denote the $p-$norm and $\infty-$norm of a matrix A as $\|A\|_p = \sup_{\|x\|_p=1} \|Ax\|_p$ and $\|A\|_\infty = \max_i \sum_j |a_{ij}|$, where $\|x\|_p := (\sum_i |x_i|^p)^{\frac{1}{p}}$ is the standard vector $p-$norm and a_{ij} is the $(i,j)^{th}$ element of the matrix A, respectively. For spatial sets $B \subseteq S$, we let $|B|$ denote the Lebesgue measure of B and, for $s, t \in \mathbb{R}^N$ we define the block indexed by $s = (s_1, ..., s_n)$ and $t = (t_1, ..., t_n)$ as $(s,t] = (s_1, t_1] \times \cdots \times (s_N, t_N] \subset \mathbb{R}^N$. For a stochastic process $\delta(s)$ with index set containing $(s,t]$ we now define the increment of $\delta(s)$ across $(s,t]$ as follows:

$$\delta((s,t]) = \sum_{\kappa_1=0,1} \cdots \sum_{\kappa_N=0,1} (-1)^{N-\sum_j \kappa_j} \delta(s_1 + \kappa_1(t_1 - s_1), \cdots, s_N + \kappa_N(t_N - s_N)).$$

For further discussion of the above definition of increment see Bickel and Wichura [1971] and Sommerfeld et al. [2018].

For each study condition $i \in \mathcal{M}$, we now assume that there exists a positive-definite spatial correlation function $\mathfrak{c}^i$: $S \times S \rightarrow [-1, 1]$ defined by the following relationship:

$$\text{cov}\left(\epsilon^i(s), \epsilon^i(t)\right) = \mathfrak{c}^i(s,t)\left(\Sigma^i(s)\Sigma^i(t)\right)^{\frac{1}{2}} \text{ for all } s, t \in S,$$

for some appropriately defined notion of matrix square root. For each $i \in \mathcal{M}$, we also denote $Z^i(s) = \Sigma^i(s)^{-1/2} X^i (X^{i'} \Sigma^i(s)^{-1} X^i)^{-1/2}$. We are now in a position to state the assumptions which allow the theory of Section 2.3 to be applied to the linear modelling context described in Section 3.1 of the main text.

Assumption S1. *We assume that, for all $i \in \mathcal{M}$:*

(a) The noise field ϵ^i has continuous sample paths with probability one. In addition, a centered unit variance Gaussian random field exists which possesses the same spatial correlation function, $\mathfrak{c}^i$, as the noise field ϵ^i and continuous sample paths with probability one.

(b) *The function* $\Sigma^i(s) : S \to \mathbb{R}^{n\times n}$ *is continuous.*

(c) *The decorrelated error field for study condition* i, $\tilde{\epsilon}^i(s) = \Sigma^i(s)^{-\frac{1}{2}}\epsilon^i(s)$, *has independent components.*

(d) *There exists* $\delta > 0$ *and* $K_\delta > 0$ *such that* $\sup_{s\in S}\mathbb{E}\big[|\tilde{\epsilon}^i_j(s)|^{2+\delta}\big] \leq K_\delta$ *(independent of* j *and* n*), and* $\sup_{s\in S}\big(n\|Z^i(s)\|_\infty^{2+\delta}\big) \to 0$ *as* $n \to \infty$.

(e) *There exists* $\gamma \geq 0$ *and* $\beta > 0$ *such that for some constant* $K_{(\beta,\gamma)} > 0$, *it is true for any block* $B \subseteq S$ *that* $\mathbb{E}\big[\|[Z^i(\cdot)\epsilon^i(\cdot)](B)\|_1^{2+\gamma}\big] \leq K_{(\beta,\gamma)}|B|^{1+\beta}$.

(f) *The function* $v^i_n(s) = L^{i'}(X^{i'}\Sigma^i(s)^{-1}X^i)^{-\frac{1}{2}}$ *satisfies* $\tau_n\frac{v^i_n(s)}{\sigma^i(s)} \to v^i(s)$ *uniformly in* s *as* $n \to \infty$ *for some continuous function* $v^i : S \to \mathbb{R}^{1\times p}$.

The above assumptions ensure that the CLT described by Assumption 2.2.1 is satisfied. A full discussion, justification and proof of sufficiency for the above conditions may be found in Sommerfeld et al. [2018]. The additional requirements listed by Assumptions 2.2.2-2.2.3 may be satisfied through placing equivalent assumptions of continuity and differentiability on the estimator and target functions, $L^{i'}\hat{\beta}^i(s)$ and $L^{i'}\beta^i(s)$, respectively.

S6 Comparison to Naive Intersections

In this section, we illustrate why intersecting the single-'study condition' confidence regions obtained using the methods of Sommerfeld et al. [2018] is not a valid approach for obtaining confidence regions for $\mathcal{F}_c$. To do so, we define the naive intersection sets $\hat{\mathcal{I}}_c^{\pm} := \cap_{i\in\mathcal{M}}\hat{\mathcal{A}}_c^{\pm,i}$ where $\{\hat{\mathcal{A}}_c^{\pm,i}\}_{i\in\mathcal{M}}$ are the single-'study condition' confidence regions obtained using the methods of Sommerfeld et al. [2018]. For ease, fix $M = 2$ and $\alpha = 0.05$. By construction, we have that;

$$\lim_{n\to\infty} \mathbb{P}[\hat{\mathcal{A}}_c^{+,1} \subseteq \mathcal{A}_c^1 \subseteq \hat{\mathcal{A}}_c^{-,1}] = 0.95 \quad \text{and} \quad \lim_{n\to\infty} \mathbb{P}[\hat{\mathcal{A}}_c^{+,2} \subseteq \mathcal{A}_c^2 \subseteq \hat{\mathcal{A}}_c^{-,2}] = 0.95. \tag{S18}$$

In other words, if $\{\hat{\mathcal{A}}_c^{\pm,i}\}_{i\in\{1,2\}}$ were generated many times using different datasets, it would be expected that the statements $\hat{\mathcal{A}}_c^{+,1} \subseteq \mathcal{A}_c^1 \subseteq \hat{\mathcal{A}}_c^{-,1}$ and $\hat{\mathcal{A}}_c^{+,2} \subseteq \mathcal{A}_c^2 \subseteq \hat{\mathcal{A}}_c^{-,2}$ would each be violated approximately 1 in 20 times. However, as illustrated by Fig. S13, these violations can occur in a variety of different ways. Consider the following three cases. For every 20 runs of the method;

- **Case 1:** Fig. S13 (a) occurs 18 times, (e) occurs once and (f) occurs once.
- **Case 2:** Fig. S13 (a) occurs 18 times, (e) occurs once and (c) occurs once.
- **Case 3:** Fig. S13 (a) occurs 19 times and (d) occurs once.

In each case, it can be seen that Statement (S18) is satisfied. However, it also follows that;

$$\begin{aligned}
\textbf{Case 1:} &\quad \lim_{n\to\infty} \mathbb{P}[\hat{\mathcal{I}}_c^{+} \subseteq \mathcal{F}_c \subseteq \hat{\mathcal{I}}_c^{-}] = 0.90,\\
\textbf{Case 2:} &\quad \lim_{n\to\infty} \mathbb{P}[\hat{\mathcal{I}}_c^{+} \subseteq \mathcal{F}_c \subseteq \hat{\mathcal{I}}_c^{-}] = 0.95,\\
\textbf{Case 3:} &\quad \lim_{n\to\infty} \mathbb{P}[\hat{\mathcal{I}}_c^{+} \subseteq \mathcal{F}_c \subseteq \hat{\mathcal{I}}_c^{-}] = 1.00.
\end{aligned}$$

We note that, by inflating the noise variance surrounding the boundary violations shown in Fig. S13, datasets can be constructed for which each of the above cases occur. The above logic may be extended to show that, in general, $\mathbb{P}[\hat{\mathcal{I}}_c^{+} \subseteq \mathcal{F}_c \subseteq \hat{\mathcal{I}}_c^{-}]$ is bounded as follows;

$$1 - M\alpha \leq \lim_{n\to\infty} \mathbb{P}[\hat{\mathcal{I}}_c^{+} \subseteq \mathcal{F}_c \subseteq \hat{\mathcal{I}}_c^{-}] \leq 1,$$

but can attain any arbitrary value in $[1 - M\alpha, 1]$ depending on the spatial structure of the noise in the data. In other words, the sets $\hat{\mathcal{I}}_c^{\pm}$ do not serve as $(1-\alpha)$ confidence regions for $\mathcal{F}_c$.

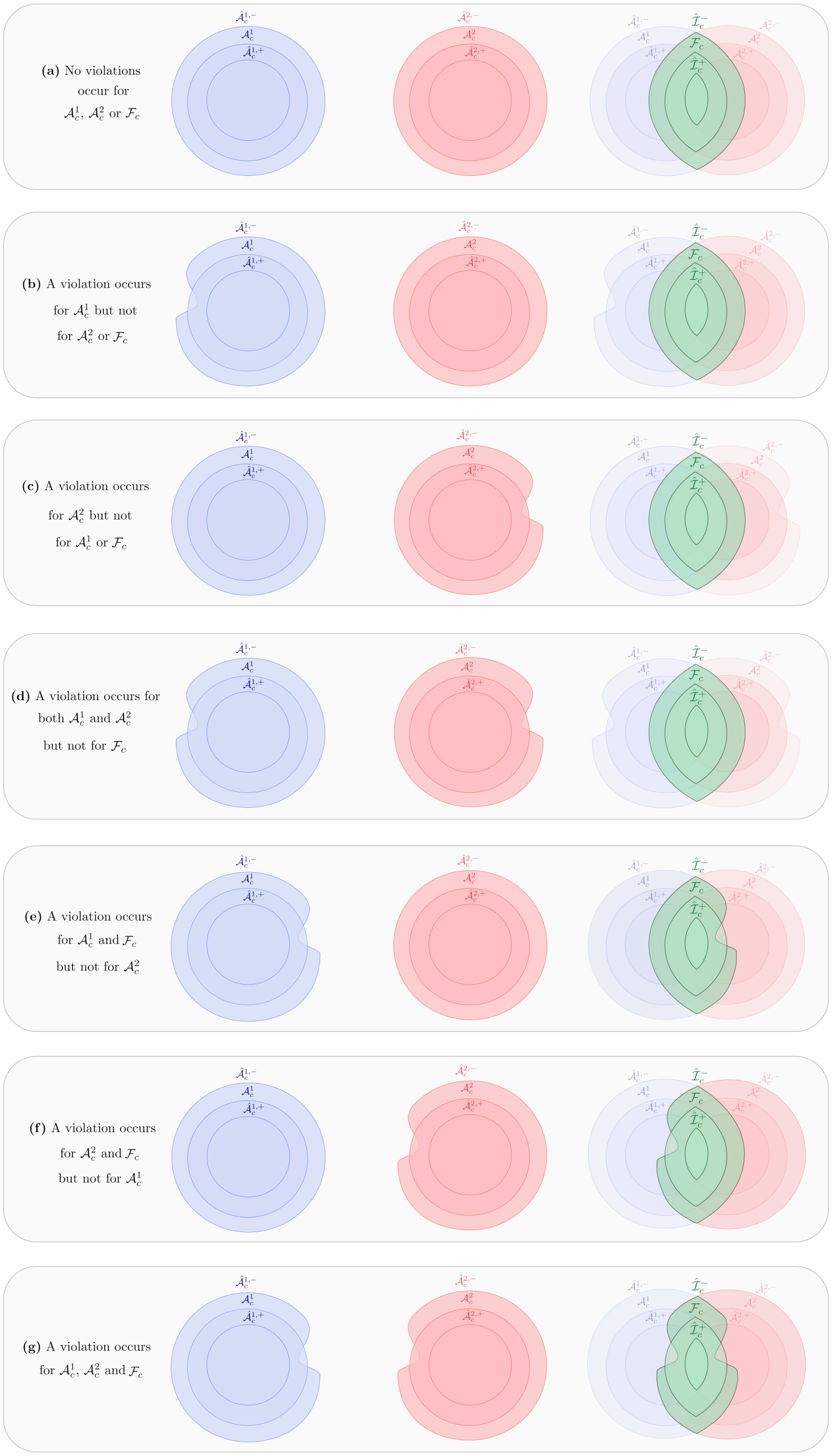

Figure S13: Potential violations of the inclusion statements $\hat{\mathcal{A}}_c^{+,1} \subseteq \mathcal{A}_c^1 \subseteq \hat{\mathcal{A}}_c^{-,1}$, $\hat{\mathcal{A}}_c^{+,2} \subseteq \mathcal{A}_c^2 \subseteq \hat{\mathcal{A}}_c^{-,2}$ and $\hat{\mathcal{I}}_c^{+} \subseteq \mathcal{F}_c \subseteq \hat{\mathcal{I}}_c^{-}$. For brevity, only violations involving the outer sets are illustrated.

References

Max Sommerfeld, Stephan Sain, and Armin Schwartzman. Confidence regions for spatial excursion sets from repeated random field observations, with an application to climate. *Journal of the American Statistical Association*, 113(523):1327–1340, 2018. doi: 10.1080/01621459.2017.1341838. URL `https://doi.org/10.1080/01621459.2017.1341838`. PMID: 31452557.

Davar Khoshnevisan. *Multiparameter Processes: An Introduction to Random Fields*. Springer Monographs in Mathematics. Springer-Verlag New York, 1 edition, 2002. ISBN 9780387954592; 0387954597; 9780387216317; 0387216316.

Gary R. Lawlor. l'hôpital's rule for multivariable functions. *The American Mathematical Monthly*, 127(8):717–725, 2020. doi: 10.1080/00029890.2020.1793635. URL `https://doi.org/10.1080/00029890.2020.1793635`.

P. J. Bickel and M. J. Wichura. Convergence Criteria for Multiparameter Stochastic Processes and Some Applications. *The Annals of Mathematical Statistics*, 42(5):1656 – 1670, 1971. doi: 10.1214/aoms/1177693164. URL `https://doi.org/10.1214/aoms/1177693164`.
